# Grewiifopenes A–K, bioactive clerodane diterpenoids from *Casearia grewiifolia* Vent.

**DOI:** 10.1007/s13659-024-00475-7

**Published:** 2024-09-14

**Authors:** Phanruethai Pailee, Paratchata Batsomboon, Wiriya Yaosanit, Theerawat Thananthaisong, Chulabhorn Mahidol, Poonsakdi Ploypradith, Nanthawan Reuk-ngam, Panita Khlaychan, Supanna Techasakul, Somsak Ruchirawat, Vilailak Prachyawarakorn

**Affiliations:** 1 Laboratory of Natural Products, Medicinal Chemistry and Organic Synthesis, Chulabhorn Research Institute, Bangkok, 10210 Thailand; 2 Department of National Parks, Wildlife and Plant Conservation, Forest Herbarium, Bangkok, 10900 Thailand; 3 Program in Chemical Sciences, Chulabhorn Graduate Institute, Bangkok, 10210 Thailand; 4 Center of Excellence on Environmental Health and Toxicology (EHT), Office of the Permanent Secretary (OPS), Ministry of Higher Education, Science, Research and Innovation (MHESI), Bangkok, 10400 Thailand

**Keywords:** *Casearia grewiifolia* Vent., Salicaceae, Clerodane diterpenoids, Cytotoxic activity, Antibacterial activity

## Abstract

Eleven novel clerodane-type diterpenoids, grewiifopenes A–K (**1**–**4** and **12**–**18**), along with nine known compounds (**5**–**11**, **19**, and **20**) were purified from the dichloromethane extract of the twigs and stems of *Casearia grewiifolia* Vent. (Salicaceae). Their spectroscopic data, including the NMR, HRESIMS, and electronic circular dichroism calculations were employed to completely characterize and elucidate the chemical structures and absolute configurations. The clerodane diterpenoids possessing a 6-OH group and no substitution at C-7 exhibited greater cytotoxic activity than others, with their IC_50_ values ranging from 0.3 to 2.9 μM. Isocaseamembrin E (**7**) exhibited antibacterial activity against *Staphylococcus aureus*, while isocaseamembrin E (**7**), corymbulosin X (**8**), caseargrewiin A (**9**), kurzipene A (**10**), and balanspene F (**11**) exhibited antibacterial activity against *Bacillus cereus*.

**Graphical Abstract:**

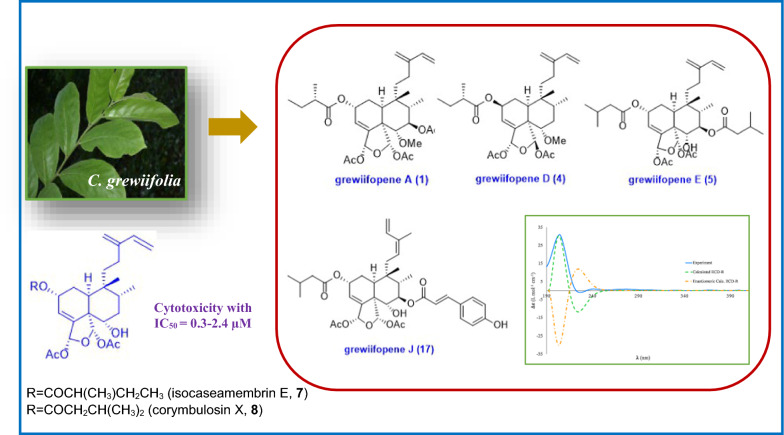

**Supplementary Information:**

The online version contains supplementary material available at 10.1007/s13659-024-00475-7.

## Introduction

*Casearia grewiifolia* Vent. belongs to the Salicaceae family, previously placed in the defunct Flacourtiaceae family, and is widely distributed in the Indo-China to Queensland (Cook). In Thailand, *C. grewiifolia*, known as “Kruaipa”, is a perennial evergreen tree (2–20 m high), growing widely in the north and northeast of Thailand. In Thai folk medicine, the four various parts of plant, root, leaves, seeds and flowers, have been traditionally utilized to treat liver dysfunction, skin diseases, hemorrhoids, as well as antidiarrheal and antipyretic agents, a tonic and febrifuge [1, 2]. Previous chemical studies of this plant revealed that the clerodane-type diterpenes were the main components of *C. grewiifolia*. Most clerodane diterpenes found in the genus *Casearia* are bicyclic diterpenoids with a *cis*-fused decalin ring system and a C-11–C-16 side chain at C-9 and a five-membered ring containing oxygen atom at C-18 and C-19 [2–6]. These compounds have already been reported to exhibit cytotoxic, antimalarial, hypoglycemic, antiulcer, anti-inflammatory, anti-snake venom, antiparasitic, and antimycobacterial activities [3–5, 7–9]. Recently, there has been reported that the grewiifolin C, a clerodane diterpene, downregulated PCSK9 and IDOL mRNA expression [6]. In an attempt to search for structurally intriguing and bioactive clerodane diterpenes, a dichloromethane extract of *C. grewiifolia* has been investigated. In present work, we describe in detail of isolation and structure determination of eleven novel clerodane diterpenoids (**1**–**4** and **12**–**18**) along with nine known compounds (**5**–**11**, **19**, and **20**) and evaluation of their cytotoxicity, antibacterial activity, and aromatase inhibition.

## Results and discussion

### Structure elucidation

**Fig. 1:**
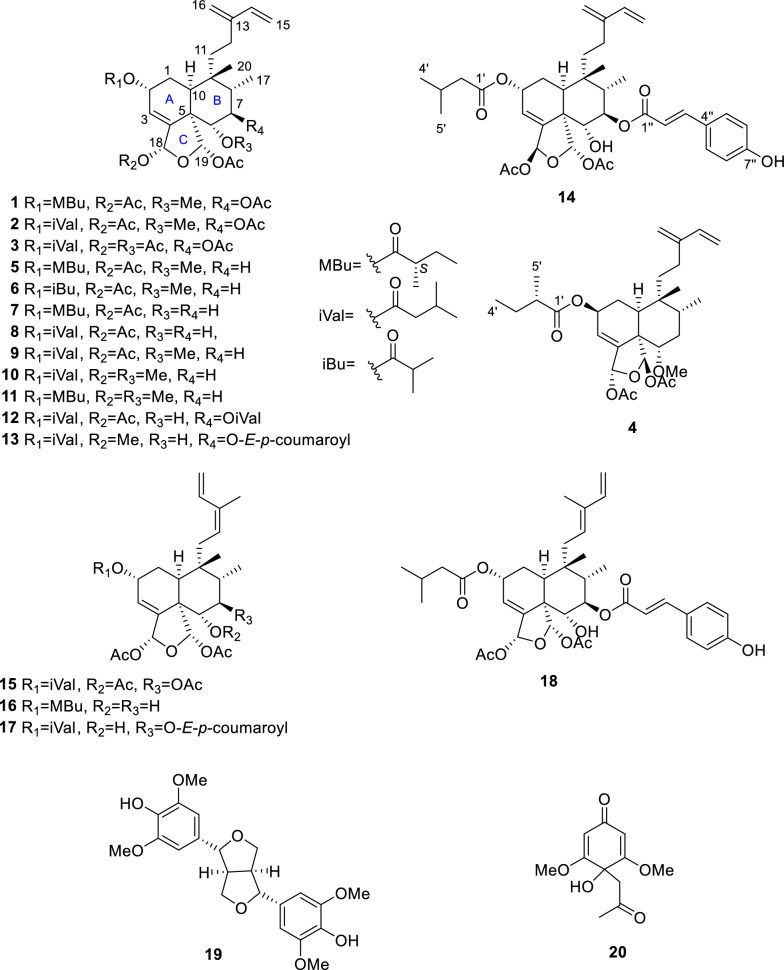
Chemical structures of compounds **1**–**20**

The CH_2_Cl_2_ crude extract of twigs and stems of *C. grewiifolia* was fractionated by chromatographic method, including silica gel and Sephadex LH20 column chromatography, and the obtained fractions were then subjected to purification by preparative TLC and HPLC, leading to the isolation and elucidation of a total of twenty compounds, which included eleven novel clerodane-type diterpenoids (grewiifopenes A–K, **1**–**4** and **12**–**18**), along with nine known compounds (Fig. 1). The known compounds, corymbulosin N (**5**) [10], corymbulosin K (**6**) [10], isocaseamembrin E (**7**) [11], corymbulosin X (**8**) [12], caseargrewiin A (**9**) [3], kurzipene A (**10**) [13], balanspene F (**11**) [14], (−)-syringaresinol (**19**) [15, 16], and 4-acetonyl-3,5-dimethoxy-*p*-quinol (**20**) [17] were identified. The structure elucidation of **1**–**4** and **12**–**18** were identified by using spectroscopic data, including 1D and 2D NMR and HRESIMS data analysis. In addition, the comparisons between the calculated and the experimental electronic circular dichroism (ECD) curves were employed to determine the absolute stereochemistry of those compounds.

**Fig. 2:**
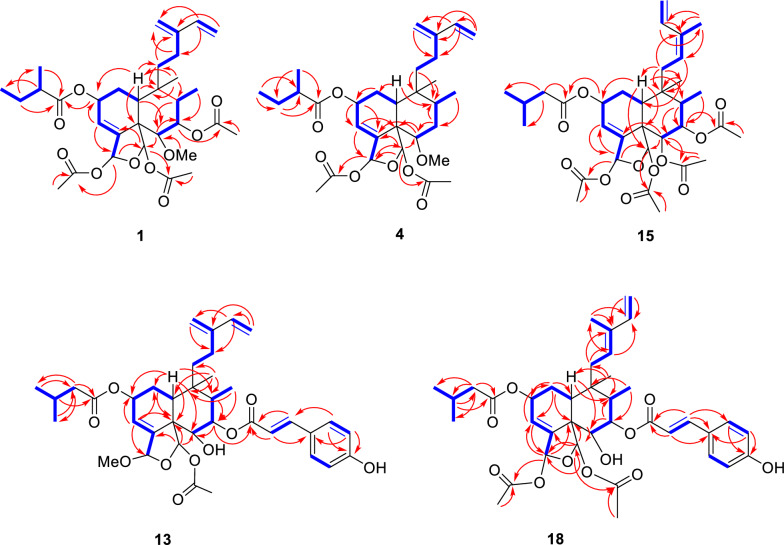
^1^H–^1^H COSY (blue) and HMBC (red) correlations of **1**, **4**, **13**, **15**, and **18**

**Fig. 3:**
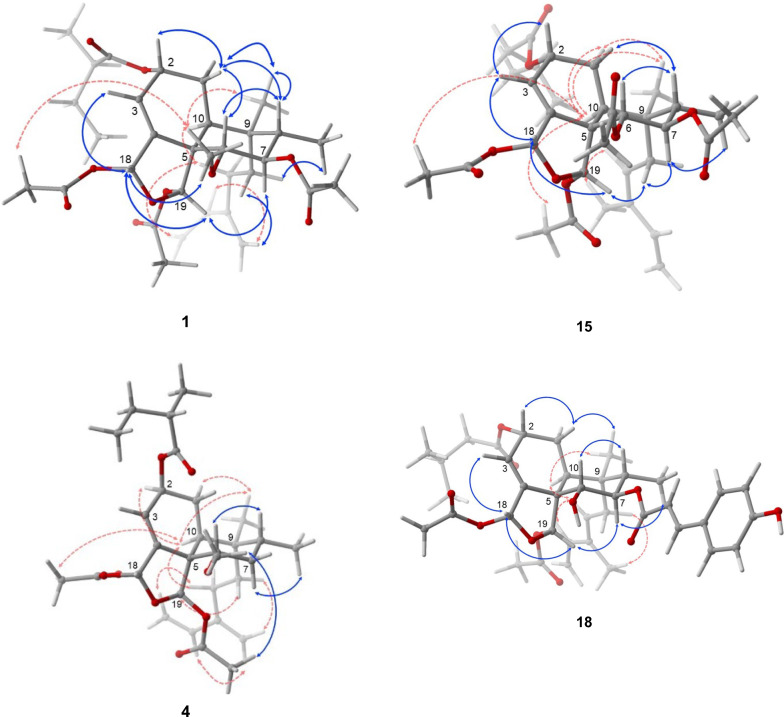
NOESY correlations of **1**, **4**, **15**, and **18**

**Fig. 4:**
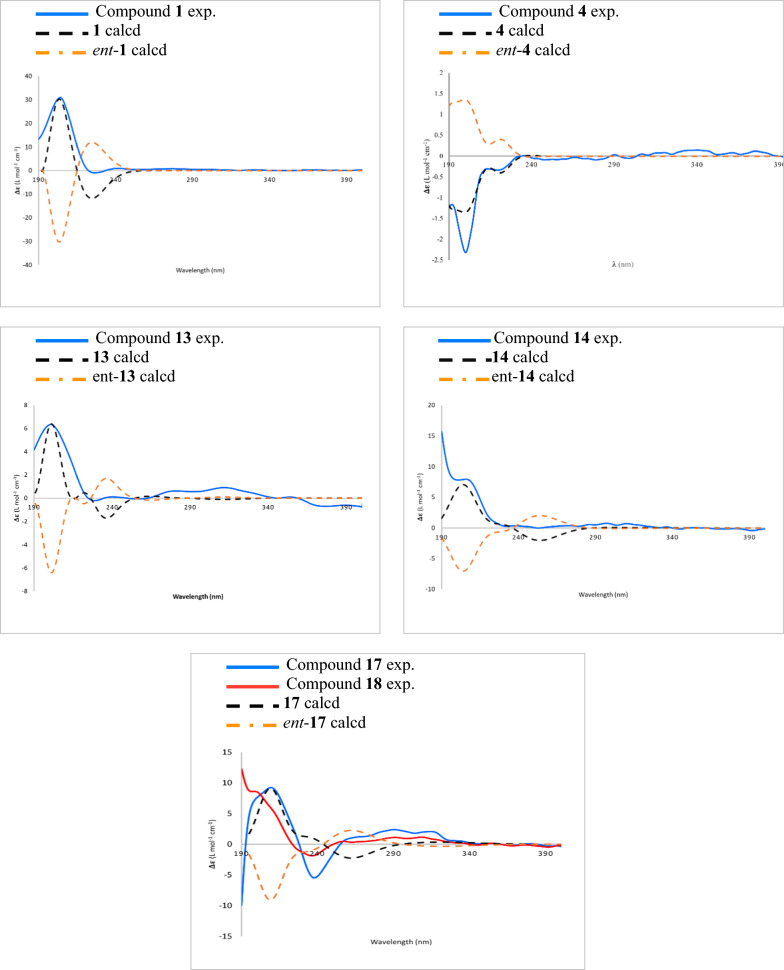
Experimental and calculated ECD spectra of **1**, **4**, **13**, **14**, **17**, and **18**

Grewiifopene A (**1**) was colorless oil and gave a molecular formula of C_32_H_46_O_10_ by HRESIMS analysis at *m/z* 613.2990 [M+Na]^+^ (calcd for C_32_H_46_NaO_10_, 613.2983), corresponding to ten degrees of unsaturation. Absorption band in the IR spectrum at 1729 cm^−1^ suggested the present of the ester carbonyl functionality. In the ^1^H NMR spectrum (Table 1) of **1**, the occurrence of signals for two methyl protons [*δ*_H_ 0.88 (d, *J* = 6.7 Hz, H-17) and 0.96 (s, H-20)], two acetal-acyloxy methine protons [*δ*_H_ 6.66 (br t, *J* = 1.3 Hz, H-18) and 6.47 (s, H-19)], six olefinic protons [*δ*_H_ 6.44 (dd, *J* = 17.7, 10.9 Hz, H-14), 6.00 (br dd, *J* = 4.1, 1.2 Hz, H-3); 5.00 (s, H-16a); 5.08 (s, H-16b); 5.15 (d, *J* = 17.7 Hz, H-15a); and 5.02 (d, *J* = 10.9 Hz, H-15b)], five aliphatic methines (including three oxygenated methines) [*δ*_H_ 2.37 (dd, *J* = 10.0, 7.3 Hz, H-10); 5.48 (br d, *J* = 2.4 Hz, H-2); 3.30 (d, *J* = 10.2 Hz, H-6); 5.19 (t-like, *J* = 10.9 Hz, H-7); and 1.83 (m, H-8)], three sp^2^ methylenes [*δ*_H_ 1.98 (m, H-1); 1.26 and 1.66 (m, H-11); and 2.09 (m, H-12)], three acetoxy methyls [*δ*_H_ 1.86 (s, at C-19); 2.06 (s, at C-18); and 2.11 (s, at C-7)], and a methoxy group [*δ*_H_ 3.42 (s, at C-6] were observed. Its ^13^C NMR spectra (Table 3) together with the help of the DEPT and HSQC data, exhibited six methyls (*δ*_C_ 10.9, 21.0, 21.3, 21.5, 25.9, and 62.4), five methylenes (*δ*_C_ 23.8, 26.9, 28.9, 112.0, and 116.1), nine methines (*δ*_C_ 36.0, 42.4, 65.8, 74.9, 84.5, 95.7, 98.5, 122.5, and 140.5) and seven nonprotonated carbons including three ester carbonyls (*δ*_C_ 169.3, 169.9, and 170.1), two olefinic carbons (*δ* 144.4 and 145.2), and two sp^3^ quaternary carbons (*δ*_C_ 39.0 and 53.7) (Table 3). In addition to the substituent group, a 2-methylbutanoyl side chain was detected from the resonances at* δ*_H_ 2.46 (sext, *J* = 6.9 Hz, H-2′)/*δ*_C_ 41.1;* δ*_H_ 1.57 (m, H-3′a) and 1.68 (m, H-3′b)/*δ*_C_ 27.0;* δ*_H_ 0.97 (t, *J* = 7.4 Hz, H-4′)/*δ*_C_ 11.6;* δ*_H_ 1.18 (d, *J* = 6.9 Hz, H-5′)/*δ*_C_ 16.6, as well as the ester carbonyl at C-1′ at* δ*_C_ 175.9. The data described above indicated that **1** was likely a clerodane diterpene structurally similar to the co-isolated corymbulosin N (**5**), which has been previously found from the bark of *Laetia corymbulosa* [10]. The only difference in the structure was that **1** has an additional acetoxy moiety at C-7 which could be inferred by the HMBC correlations (Fig. 2) of H-7 to the ester carbonyl (*δ*_C_ 169.9) and both Me-17 and H-6 to C-7 (*δ*_C_ 74.9). Five structural fragments were established from the ^1^H–^1^H COSY spectrum of **1** as shown in Fig. 2. The assignment of relative stereochemistry of **1** was initially attempted by using the values of ^1^H–^1^H coupling constants (Table 1) together with a NOESY experiment (Fig. 3). Consideration of the relative configurations of H-6, H-7, and H-8 in ring B were first analyzed to delineate the relationships of other protons throughout the clerodane diterpenoid **1**. The strong 1,3 diaxially NOESY correlation of H-6 and H-8 and the large ^*3*^*J* coupling constants of H-7 (10.9 Hz) appearing as a triplet suggested the *trans* diaxial relationships among H-6, H-7, and H-8. In addition, the NOESY correlations of H-8 and Me-20; Me-20 and H-10; H-10 and H-12; and Me-20 and H-1 indicated *cis*-fused bicyclic six-membered rings, rings A and B, with an α-equatorial and an α-axial orientation for H-10 and CH_2_-11 of the C-11–C-16 conjugated diene moiety, respectively. The acetoxy group located at C-19 was oriented on *α* face, according to the NOESY cross peak between H-19/H-7. In addition, the homoallylic ^5^*J* coupling (*J* = 1.3 Hz) of H-18 and H-2 from the overlapping π-orbitals of the double bond with the 1*s* orbitals of the hydrogen atom also inferred the *α*-orientation for both acetoxy group at C-18 and 2-methylbutyryloxy side chain at C-2 [18–20]; this was also supported by the key correlations in NOESY experiment of 18-OAc/H-10, H-18/H-19, and H-18/6-OMe. Furthermore, the α-orientation of the 2-methylbutyryloxy moiety at C-2 was supported from the characteristic chemical shift at* δ*_C_ 65.8 (C-2); the chemical shift of C-2 with the α-oriented side chain would appear at the higher field (less than 66 ppm) than that of the β-oriented one which would appear at the lower field (greater than 70 ppm) [10, 21]. Additionally, the NOESY spectrums of H-11 and H-16; H-16 and H-14; and H-12 and H-15 suggested that the conjugated diene at C-9 was *s-trans* conformation. ECD comparison of its experimental with the calculated analysis (Fig. 4), thereby allowing the absolute stereochemistry of **1** as shown.

**Table 1:** ^1^H NMR spectroscopic data of compounds **1**–**4**, **15**, and **16** in CDCl_3_ (600 MHz, *J* in Hz)

Position	**1**	**2**	**3**^a^	**3**	**4**	**15**	**16**
1	1.98, m	1.99, m	1.67^b^, m 1.72^c^, m	2.01^b^, m 2.11, m	1.70, m 2.16, m	2.01^b^, m 2.09, dd (13.5, 3.0)	1.90, dd (8.8, 3.7)
2	5.48, br d (2.4)	5.48, br d (2.1)	5.41, s	5.48, br t (1.4)	5.60, m	5.49, br t (4.2)	5.44, br d (2.5)
3	6.00, br dd (4.1, 1.2)	6.02, br d (2.8)	6.03, d (3.6)	6.04, br d (3.7)	5.81, br s	6.05, d (3.7)	6.01, d (3.0)
6	3.30, d (10.2)	3.30, d (10.3)	5.40, d (10.3)	5.20^c^, m	3.51, dd (12.1, 3.9)	5.20, d (10.4)	3.81, dd (12.2, 3.3)
7	5.19, t-like (10.9)	5.19, t-like (10.9)	5.52, t-like t (11.0)	5.20^c^, m	1.48^b^, m 1.91, m	5.14, t-like (10.6)	1.60^b^, m 1.74^c^, m
8	1.83, m	1.83, dq (11.7, 6.7)	1.66^b^, m	1.95, m	1.78, m	1.94, m	1.76^c^, m
10	2.37, dd (10.0, 7.3)	2.35, dd (10.1, 6.9)	2.48, dd (13.6, 3.2)	2.41, dd (13.5, 3.1)	2.36, dd (14.1, 2.8)	2.45, dd (13.5, 3.0)	2.40, m
11	1.26, m; 1.66^b^, m	1.26, m 1.67, m	1.44, td (12.4, 2.3) 1.59, td (13.2, 4.6)	1.28, m 1.70, m	1.22, m 1.48, m	1.68, d (16.1) 2.56, dd (16.1, 9.3)	1.60^b^, m 2.36, m
12	2.09^c^, m	2.08^b^, m	2.02, br dd (13.7, 4.3) 2.14, br t (13.3)	2.12, m	2.07^c^, m	5.25, m	5.28, br d (7.9)
14	6.44, dd (17.7, 10.9)	6.44, dd (17.6, 10.9)	6.30, dd (17.6, 10.8)	6.45, dd (17.6, 10.9)	6.42, dd (17.6, 10.9)	6.64, dd (17.2, 10.8)	6.63, dd (17.3, 10.8)
15	5.02, d (10.9) 5.15, d (17.7)	5.03, d (10.9) 5.17, d (17.2)	4.85, d (10.8) 5.06, d (17.6)	5.05, d (10.9) 5.19, d (17.6)	5.04, d (10.6) 5.21, d (17.6)	5.15, d (10.8) 5.23, d (17.2)	5.11, d (10.8) 5.20, d (17.3)
16	5.00, s 5.08, s	5.00, s 5.08, s	4.81, br s 4.90, s	5.00, s 5.09, s	4.92, s 5.04, s	1.82, s	1.79, s
17	0.88, d (6.7)	0.88, d (6.7)	0.783, d (6.7)	0.91, d (6.7)	0.94, d (7.0)	0.91, d (6.7)	0.93, d (6.7)
18	6.66, br t (1.3)	6.66, br t (1.6)	6.78, br d (1.5)	6.42, br t (1.6)	6.63, t (1.6)	6.42, br t (1.5)	6.74, br t (1.3)
19	6.47, s	6.47, s	7.02, s	6.57, s	6.38, s	6.61, s	6.51, s
20	0.96, s	0.97, s	0.63, s	1.014, s	0.95, s	0.86, s	0.78, s
2′	2.46, sext (6.9)	2.26, dd (14.2, 6.9) 2.29, dd (14.2, 7.2)	1.87, d (7.4)	2.25, dd (14.2, 6.9) 2.28, dd (14.2, 7.1)	2.38, m	2.26, dd (14.2, 6.9) 2.29, dd (14.2, 7.3)	2.48, sext (6.8)
3′	1.57, m 1.68^b^, m	2.13, m	1.98, m	2.12, m	1.50^b^, m 1.72, m	2.13, m	1.56, m 1.70, m
4′	0.97, t (7.4)	1.01, d (6.6)	0.776, d (6.7)	1.00, d (6.4)	0.93, t (7.5)	1.02, d (6.6)	0.97, t (7.4)
5′	1.18, d (6.9)	1.02, d (6.6)	0.80, d (6.7)	1.013, d (5.6)	1.18, d (7.0)	1.01, d (6.6)	1.18, d (6.9)
6-OMe	3.42, s	3.41, s			3.34, s		
6-OAc			1.78, s	2.07, s		2.071, s	
7-OAc	2.11^c^, s	2.11, s	1.73^c^, s	2.02^b^, s		2.01^b^, s	
18-OAc	2.06^c^, s	2.06^b^, s	1.54, s	2.03^b^, s	2.07^c^, s	2.066, s	2.10, s
19-OAc	1.86, s	1.86, s	1.69, s	1.88, s	1.88, s	2.00, s	2.00, s

^a^Recorded in C_6_D_6_

^b,c^Overlapped signals

**Fig. 5:**
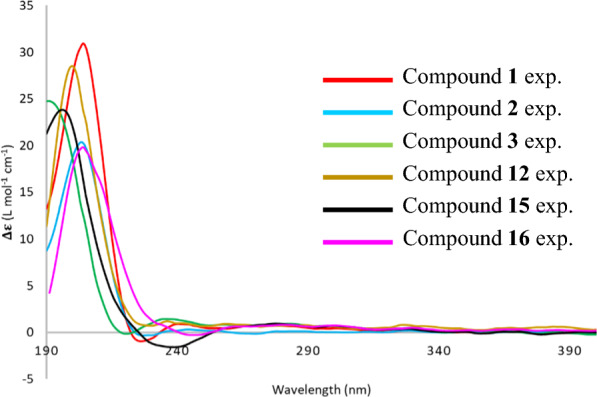
Experimental ECD spectra of **1**–**3**, **12**, **15**, and **16**

Grewiifopene B (**2**) had the same molecular formula, C_32_H_46_O_10_, as that of compound **1**, by HRESIMS at *m/z* 613.2981 [M+Na]^+^ (calcd for C_32_H_46_NaO_10_, 613.2983). Analyses of ^1^H and ^13^C NMR spectroscopic data (Tables 1 and 3) of **2** displayed close similarities to those of **1**, with the only difference assignable to the presence of an isovaleryloxy moiety in **2** instead of 2-methylbutyryloxy side chain in **1**, while the other moieties of **2** appear identical to those of **1**. The characteristic ^1^H NMR resonances of an isovaleryloxy moiety at* δ*_H_ 2.26 (dd, *J* = 14.2, 6.9 Hz, H-2′a), 2.29 (dd, *J* = 14.2, 7.2 Hz, H-2′b), 2.13 (m, H-3′), 1.01 (d, *J* = 6.6 Hz, H-4′), 1.02 (d, *J* = 6.6 Hz, H-5′), along with the ^13^C NMR resonances at* δ*_C_ 172.4 (C-1′), 43.6 (C-2′), 26.1 (C-3′), 22.3 (C-4′), and 22.4 (C-5′) were observed in **2** and the long-range HMBC experiments (Figure S16, Supporting Information) of H-1, H-3, H-10, and H-18 to C-2 (*δ*_C_ 66.0); and H-2 to C-1′ (*δ*_C_ 172.4) suggested that this side chain is attached to C-2. Analyses of the NOESY experiment and the coupling constants in ^1^H NMR of **1** and **2** suggested that their relative configurations were identical (Figure S17, Supporting Information), while their closely related ECD curves of **1** established its absolute configuration (Fig. 5). Therefore, the chemical structure of **2** was purposed as shown.

Grewiifopene C (**3**) was assigned the molecular formula C_33_H_46_O_11_ by HRESIMS at *m/z* 641.2931 [M+Na]^+^ (calcd for C_33_H_46_NaO_11_, 641.2932). Analyses of ^1^H and ^13^C NMR data (Tables 1 and 3) of **3** was similar to those of **2**, except for the different substituent at C-6; the appearance of an acetoxy group in **3** instead of a methoxy group in **2**. This inference was implied by the downfield chemical shift of H-6 from* δ*_H_ 3.30 (d, *J* = 10.3 Hz) in **2** to 5.20 (m, overlapped) in **3**, signals for 6-OAc (*δ*_H_ 2.07/*δ*_C_ 20.9) and ester carbonyl (*δ*_C_ 170.1) as well as the HMBC experiments of H-19, H-7, and 6-OAc to C-6 (*δ*_C_ 73.7) and H-6 to* δ*_C_ 170.1 (Figure S26, Supporting Information). Inspection of ^1^H–^1^H coupling constants and NOESY spectra of **3** and **2** and their matched ECD curves (Fig. 5) conferred the absolute stereochemistry of **3** as shown. However, the coupling constants between H-6 and H-7 of **3** in CDCl_3_ were difficult to measure directly due to signal overlap, but a spectrum obtained from a sample using benzene-*d*_6_ as solvent (Table 1) provided the resolution of these two positions. Therefore, the chemical structure of **3** was purposed as shown.

Grewiifopene D (**4**) has an identical molecular formula to that of the co-occurring corymbulosin N (**5**). The resonance patterns of NMR data of **4** was identical to those of corymbulosin N (**5**) [10], excepted for the different stereochemistry at C-2 and C-19. However, the configuration at C-2 could be deduced by comparing the ^13^C diagnostic shift of C-2 (*δ*_c_ around 66.0 for 2*R*; *δ*_c_ around 70.5 for 2*S*) [10, 21]. Therefore, the *S*-configuration at C-2 of **4** could be inferred from the ^13^C chemical shift at *δ*_C_ 70.5. The observed NOESY correlations of 19-OAc/6-OMe and H-19/H-11 (Fig. 3) implied the relative configurations of 19-OAc as *β*-oriented in **4**. Based on these results, together with ECD comparison of its experimental with the calculated analysis (Fig. 4), its absolute stereochemistry was as shown.

Unfortunately, the stereochemistry at C-2′ of the 2-methybutyryl side chain in grewiifopenes A and D (**1** and **4**) could not be determined by comparing experimental ECD spectra with calculated ECD spectra method because its spectra of the 2′*R* and 2′*S* isomers of both compounds could not reveal the difference of diastereomers. These data are consistent with the previous reports for corymbulosin N (**5**) possessing the 2-methybutyryl side chain moiety [10]. In an effort to further study, corymbulosin N (**5**) was used as authentic compounds because it was available in sufficient quantity by its conversion to the corresponding 2-methylbutyric acid benzyl ester using transesterification by heating in benzyl alcohol in the presence of a catalytic amount of dimethylaminopyridine (DMAP). In addition, the two authentic samples, *S*- and* R*/*S*-2-methylbutyric acid benzyl ester were synthesized from *S*- and *R*/*S*-2-methylbutyric acid, respectively. The ester products, including the desired product from transesterification of **5** and the two benzyl-2-methylbutyrate in *S-* and *R*/*S*-forms, were analyzed with the chiral-phase HPLC on CHIRALPAK® AD-H column (40% CH_3_CN-H_2_O at flow rate 1 mL/min) revealing the* S*-configuration at C-2′ of 2-methylbutyryl side chain in corymbulosin N (**5**) (Fig. S118, Supporting Information). Based on the biogenesis rationale of compounds isolated from the same plant, the absolute configuration at C-2′ of 2-methylbutyryl side chain in grewiifopenes A and D (**1** and **4**), isocaseamembrin E (**7**), balanspene F (**11**), and grewiifopene I (**16**) should be established as *S*-configuration.

From the HRESIMS and NMR data of **12**, its molecular formula was detected to be C_34_H_50_O_10_. The ^1^H and ^13^C NMR spectroscopic data (Tables 1 and 3) showed two sets of signals from the two isovaleryloxy groups. The comparison of the NMR data of compounds **2** and **12** indicated a structural difference by replacing the methoxy group at C-6 and an acetoxy group at C-7 in **2** with the hydroxy group and one of the isovaleryloxy groups in **12**, respectively. This conclusion was in agreement with a series of the correlations of H-6/H-7/H-8/Me-17 in COSY experiment (Fig. S51, supporting information) and the characteristic correlations from H-7 to C-1′′ (*δ*_C_ 173.9); H-10 and H-8 to C-6 (*δ*_C_ 75.4); H-6 to C-19 (*δ*_C_ 97.9); and Me-17 to C-7 (*δ*_C_ 74.8), C-8 (*δ*_C_ 41.8), and C-9 (*δ*_C_ 39.1) in the HMBC experiment (Fig. S53, supporting information). The absorption in the IR spectrum at 3503 cm^−1^ implied the occurrence of the hydroxy moiety in **12**. Based on the NOESY experiments (Fig. S54, supporting information) and ^1^H–^1^H coupling constants suggested that **12** had the same relative configurations as those of **2**. Furthermore, the similarity of their ECD spectra of **12** and **2** (Fig. 5) indicated that the absolute stereochemistry of both **12** and **2** were virtually identical. Thus, compound **12** was elucidated and named as grewiifopene E.

Grewiifopene F (**13**) was colorless oil, and its molecular formula of C_37_H_48_O_10_ was determined by HRESIMS analysis, which exhibited a [M+Na]^+^ at *m/z* 675.3138 (calcd for C_37_H_48_NaO_10_, 675.3140). Comparison of the ^1^H and ^13^C NMR spectra (Tables 2 and 3) of **13** with **12** disclosed that compound **13** possessed a *trans p*-coumaroyl moiety at C-7 and a methoxy moiety at C-18 instead of the isovaleryloxy and acetoxy moieties in **12**, respectively. The ^1^H and ^13^C NMR spectroscopic data (Tables 2 and 3) were consistent with the occurrence of a *trans p*-coumaroyl group [*δ*_H_ 6.27 (d, *J* = 15.9 Hz, H-2″), 7.62 (d, *J* = 15.9 Hz, H-3′′), 6.82 (d, *J* = 8.6 Hz, H-6′′, H-8′′), and 7.39 (d, *J* = 8.6 Hz, H-5′′, H-9′′);* δ*_C_ 167.9 (C-1″), 114.8 (C-2″), 145.39 (C-3″), 127.0 (C-4″), 130.1 (C-5″, C-9″), 115.9 (C-6″, C-8″), 158.0 (C-7″)] and a methoxy signal (*δ*_H_ 3.43/*δ*_C_ 56.3). It was evident from the HMBC experiments (Fig. 2) from H-7, H-2′′, and H-3′′ to carbonyl of ester group at* δ*_C_ 167.9 (C-1″) and Me-17 to C-7 (*δ*_C_ 75.1) that this *trans p*-coumaroyl group was resonated at C-7. Moreover, a methoxy group at C-18 position of **13** was evident by the correlations of H-3, 18-OMe, and H-19 to C-18 (*δ*_C_ 104.5). Analysis of coupling constants in ^1^H NMR and NOESY experiments suggested that **13** had the same relative configurations as those of **12**. Furthermore, ECD comparison of its experimental with the calculated analysis (Fig. 4), and its absolute stereochemistry was assigned as shown.

**Table 2:** ^1^H NMR spectroscopic data of compounds **12**–**14**, **17**, and **18** in CDCl_3_ (600 MHz, *J* in Hz)

Position	**12**	**13**	**14**	**17**	**18**
1	1.97, m	1.98, m	1.99, m	1.93, m	2.01, m
2	5.45 br d (2.2)	5.50, m	5.46, br d (2.5)	5.46, m	5.50, m
3	6.04, br dd (4.1, 1.2)	6.10, br dd (4.2, 1.1)	6.03, br s	5.98, br s	6.07, dd (4.1, 1.3)
6	3.68, d (10.3)	3.75, d (8.2)	3.78, d (10.1)	3.76, d (9.6)	3.80, dd (8.6,6.4)
7	5.04, t-like (11.0)	5.18, t-like (10.9)	5.16, t-like (10.9)	5.14, t-like (10.7)	5.15, t-like (10.9)
8	1.87^a^, m	1.94, m	1.94, m	1.90, m	1.97^a^, m
10	2.38, dd (10.5, 6.8)	2.37, dd (11.0, 6.0)	2.40, t-like (8.0)	2.42, dd (11.4, 5.7)	2.46^b^, m
11	1.25, m 1.69, m	1.30, m 1.71, m	1.28, m 1.72, m	1.68, d (16.2) 2.56, dd (16.2, 9.2)	1.79, br d (17.5) 2.46^b^, m
12	2.10, m	2.10^a^, m	2.11, m	5.25, br d (8.8)	5.40, br d (5.3)
14	6.44, dd (17.6, 10.8)	6.45, dd (17.5, 10.9)	6.45, dd (17.5, 10.9)	6.63, dd (17.2, 10.7)	6.32, dd (17.2, 11.0)
15	5.05, d (10.8) 5.18, d (17.6)	5.04, d (10.9) 5.17, d (17.5)	5.05, d (10.9) 5.19, d (17.5)	5.14, d (10.7) 5.21, d (17.2)	5.00, d (11.0) 5.16, d (17.2)
16	4.97, s 5.08, s	5.00, s 5.09, s	4.99, s 5.09, s	1.81, s	1.72, s
17	0.92, d (6.7)	0.95, d (6.7)	0.96, d (6.7)	0.95, d (6.7)	0.99, d (6.7)
18	6.73, t (1.4)	5.49, t (1.5)	6.74, s	6.71, t (1.6)	6.76, t (1.6)
19	6.54, s	6.57, s	6.59, s	6.64, s	6.66, s
20	0.99, s	0.99, s	1.00, s	0.82, s	0.91, s
2′	2.26^b^, m	2.24, dd (14.4, 6.9) 2.27, dd (14.4, 7.3)	2.26, dd (14.1, 6.9) 2.29, dd (14.1, 7.2)	2.29, m	2.30, dd (14.2, 6.7) 2.33, dd (14.2, 7.2)
3′	2.13^c^, m	2.12^a^, m	2.14, m	2.14, m	2.18, m
4′	1.01^d^, d (6.6)	1.002^d^, d (6.6)	1.023^d^, d (6.6)	1.03^d^, d (6.6)	1.06, d (6.6)
5′	0.98^e^, d (6.7)	1.004^d^, d (6.7)	1.015^d^, d (6.6)	1.02^d^, d (6.7)	1.05, d (6.6)
2′′	2.26^b^, m	6.27, d (15.9)	6.30, d (15.9)	6.28, d (15.9)	6.33, d (15.9)
3′′	2.13^c^, m	7.62, d (15.9)	7.66, d (15.9)	7.62, d (15.9)	7.69, d (15.9)
4′′	1.02^d^, d (6.6)				
5′′ (9′′)	0.98^e^, d (6.7)	7.39, d (8.6)	7.41, d (8.6)	7.39, d (8.6)	7.45, d (8.6)
6′′ (8′′)		6.82, d (8.6)	6.84, d (8.6)	6.85, d (8.6)	6.87, d (8.6)
18-OMe		3.43, s			
18-OAc	2.05, s		2.06, s	2.10, s	2.11, s
19-OAc	1.88^a^, s	1.90, s	1.90, s	2.00, s	1.97^a^, s

^a^^,b,c^Overlapped signals

^d,e^Signals may be interchanged in each column

**Table 3:** ^13^C NMR spectroscopic data of compounds **1**–**4** and **12**–**18** in CDCl_3_ (150 MHz)

Position	**1**	**2**	**3**^a^	**3**	**4**	**12**	**13**	**14**	**15**	**16**	**17**	**18**
1	26.9	26.9	27.3	26.7	26.6	26.8	27.1	26.9	26.6	26.7	26.8	26.8
2	65.8	66.0	66.4	65.8	70.5	66.0	66.0	66.1	65.8	66.1	66.1	66.0
3	122.5	122.4	124.3	124.1	123.8	122.2	122.1	122.1	124.0	121.9	122.0	122.1
4	145.2	145.2	144.2	143.1	145.2	144.6	145.42^b^	144.6^b^	143.0	145.3	144.4	144.5
5	53.7	53.6	53.7	52.8	53.1	53.9	53.8	53.8	52.6	53.6	53.5	53.5
6	84.5	84.6	74.4	73.7	83.1	75.4	75.7	75.4	73.7	72.9	75.2^b^	75.3^f^
7	74.9	74.9	73.4	72.8	31.3	74.8	75.1	75.1	72.9	37.4	75.3^b^	75.3^f^
8	42.4	42.4	41.8	41.6	37.0	41.8	42.0	42.0	41.0	36.9	41.3	41.3
9	39.0	39.0	39.4	38.9	38.2	39.1	39.1	39.1	39.1	37.8	39.3	39.2
10	36.0	36.1	37.5	36.7	41.2	35.8	35.8	35.9	36.9	36.7	36.1	36.2
11	28.9	29.0	29.4	28.9	27.5	29.1	29.2	29.1	30.0	29.1	30.1	31.5
12	23.8	23.8	24.5	23.8	23.8	23.7	23.7	23.8	125.4	126.5	125.6	128.0
13	144.4	144.5	144.7	144.4	145.1	144.5	144.6	144.5^b^	134.1	133.6	134.0	136.2
14	140.5	140.5	141.2	140.3	140.3	140.4	140.4	140.4	133.2	133.3	133.3	141.0
15	112.0	112.1	112.3	112.2	112.5	112.2	112.3	112.2	114.9	114.4	114.8	111.5
16	116.1	116.1	117.0	116.0	115.5	115.9	115.8	116.0	20.4	20.4	20.4	12.1
17	10.9	10.9	11.4	11.0	15.9	11.2	11.2	11.2	10.9	15.6	11.1	11.1
18	95.7	95.7	95.3	94.5	95.7	95.5	104.5	95.6	94.7	95.7	95.8	95.7
19	98.5	98.5	98.8	98.1	98.0	97.9	97.7	98.0	97.5	97.2	97.4	97.2
20	25.9	25.8	25.8	25.7	25.6	25.7	25.8	25.7	25.4	25.0	25.3	25.4
1′	175.9	172.4	172.0	172.2	176.5	172.4	172.7	172.5	172.3	175.9	172.6	172.5
2′	41.1	43.6	43.6	43.6	41.2	43.5^b^	43.7	43.7	43.6	41.1	43.6	43.6
3′	27.0	26.1	26.4	26.1	26.8	25.8^c^	26.0	26.2	26.1	27.1	26.1	26.2
4′	11.6	22.3	22.58	22.3	11.7	22.32^d^	22.4^c^	22.3^c^	22.3	11.6	22.36^c^	22.36^b^
5′	16.6	22.4	22.64	22.4	16.6	22.40^e^	22.5^c^	22.4^c^	22.4	16.6	22.39^c^	22.41^b^
1″						173.9	167.9	168.0			168.0	167.9
2″						43.6^b^	114.8	114.6			114.3	114.7
3″						26.2^c^	145.39^b^	145.6			145.9	145.6
4″						22.37^d^	127.0	127.0			126.7	127.1
5″ (9″)						22.45^e^	130.1	130.2			130.3	130.2
6″ (8″)							115.9	116.0			116.0	115.9
7″							158.0	158.0			158.3	157.9
6-OMe	62.4	62.4			57.5							
6-OAc			170.3/20.8	170.1^b^/20.9					170.1/21.0^b^			
7-OAc	169.9/21.0	169.9/21.0	169.9^f^/20.6	170.0^b^/20.6					169.9/20.6			
18-OMe							56.3					
18-OAc	170.1/21.3	170.1/21.3	169.9^f^/20.9	169.7/21.1	170.2/21.4	169.9/21.1		170.1/21.2	169.8/21.1^b^	170.1/21.2	170.3/21.2	170.1/21.2
19-OAc	169.3/21.5	169.4/21.5	168.8/21.7	169.3/21.4	169.9/21.7	169.3/21.3	170.0/21.5	169.4/21.4	168.8/21.3	169.2/21.3	168.9/21.3	169.1/21.5

^a^Recorded in C_6_D_6_

^b,c,d,e^Signals may be interchanged in each column

^f^Overlapped signals

From the HRESIMS and NMR spectra of **14**, its molecular formula was determined to be C_38_H_48_O_11_. Analyses of the ^1^H and ^13^C NMR data (Tables 1 and 3) of **14** were closely similar to those of **13**, excepted that a methoxy group at C-18 was substituted with an acetoxy group in **14**. This evidence was verified by the downfield shift of H-18 from* δ*_H_ 5.49 (t,* J* = 1.5 Hz) in **13** to 6.74 (s) in **14**. NOESY experiments (Fig. S74, supporting information) and ^1^H–^1^H coupling constants of **13** and **14** indicated the identical relative stereochemistry of **13** and **14**, except for the H-18 position, which resonated as a singlet due to the disappearance of homoallylic ^5^*J* coupling between H-2 and H-18 and no cross peak between H-18 and H-19 in the NOESY spectrum. This, in turn, would suggest the *α*-orientation for H-18; the other configuration of **14** was assigned to be the same as **13** based on the NOESY experiments and ^1^H–^1^H coupling constants. Consequently, the absolute stereochemistry of **14** was finally deduced as shown by the ECD calculation method (Fig. 4). Therefore, the structure of **14** (grewiifopene G) was assigned.

Grewiifopene H (**15**) was assigned the molecular formula C_33_H_46_O_11_ by HRESIMS and ^1^H and ^13^C NMR and DEPT experiments. The chemical structure of **15** was nearly identical to that of **3**, except that the resonances for 3-methylenepent-1-ene unit at C-9 in **3** were replaced by resonances for a 3-methylpenta-1,3-diene moiety in **15**. The above assignment was further supported by the ^1^H NMR spectrum (Table 1), which showed some characteristic signals two double bonds [including a terminal monosubstituted olefin (*δ*_H_ 6.64 (dd, *J* = 17.2, 10.8 Hz, H-14), 5.15 (d, *J* = 10.8 Hz, H-15a) 5.23 (d, *J* = 17.2 Hz, H-15b) and one trisubstituted olefin (*δ*_H_ 5.25 (m, H-12)], an olefinic methyl group [*δ*_H_ 1.82 (s, Me-16)], and nonequivalent methylene protons [*δ*_H_ 1.68 (d, *J* = 16.1 Hz, H-11a and 2.56 (dd, *J* = 16.1, 9.3 Hz, H-11b)]. Further HMBC experiments (Fig. 2) from H-11 to C-9 (*δ*_C_ 39.1), C-10 (*δ*_C_ 36.9), Me-20 (*δ*_C_ 25.4), C-13 (*δ*_C_ 134.1); Me-16 to C-12 (*δ*_C_ 125.4), C-13, and C-14 (*δ*_C_ 133.2); H-14, H-15, and Me-16 to C-13 confirmed the 3-methylpenta-1,3-diene at C-9 in **15**. The trisubstituted double bond at C12/C-13 for compound **15** was assigned with the *Z* geometry due to the NOESY cross peak between H-12 and Me-16 (Fig. 3). Moreover, the *Z-*configuration was confirmed by comparing the ^13^C NMR chemical shift at* δ*_C_ 20.4 for 16-Me in compound **15** with the available diagnostic data for *Z*-(*δ*_C_ 20.3) and *E*-(*δ*_C_ 12.0) double bonds [18, 19, 21]. With the similar ECD curves (Fig. 5), NOESY experiments (Fig. 3), and ^1^H–^1^H coupling constants, compound **15** was deduced to possess the same absolute configurations as those of **3**.

Grewiifopene I (**16**) was assigned the molecular formula C_29_H_42_O_8_, by HRESIMS at *m*/*z* 541.2782, [M+Na]^+^. The IR absorptions at 3456 cm^−1^ for the hydroxy and 1749, and 1729 cm^−1^ for ester carbonyl functionalities were detected. Analyses of the ^1^H and ^13^C NMR spectra (Tables 1 and 3) of **16** showed resonances similar to those of the co-occurring isocaseamembrin E (**7**) [11], except for the structural differences in the C-11–C-16 side chain moiety at C-9, which was identified as (*Z*)-3-methylpenta-1,3-diene. The relative stereochemistry of **16** was virtually similar to that of isocaseamembrin E (**7**) by analyzing their coupling constants in ^1^H NMR and NOESY correlations (Fig. S94, supporting information). The comparison of the ECD curves of compound **16** with those of structural analogs **1**, **2**, **3**, **12**, and **15** (Fig. 5) penultimately assigned its absolute stereochemistry as shown.

Grewiifopene J (**17**) was isolated as a colorless oil. Its HRESIMS data gave a molecular formula of C_38_H_48_O_11_. Analyses of its ^1^H and ^13^C NMR spectra (Tables 2 and 3) of **17** displayed the same skeleton as **12**, with the structural differences at C-9 and C-7. The NMR data exhibited resonances for (*Z*)-3-methylpenta-1,3-diene moiety at C-9 and *trans p*-coumaroyloxy group at C-7 in **17** instead of the 3-methylenepent-1-ene side chain and isovaleryloxy group, respectively, in **12**. On the basis of the NOESY experiments (Fig. S104, supporting information) and ^1^H–^1^H coupling constants, compound **17** had the same relative stereochemistry as those of **12**. The absolute stereochemistry of **17** was established as shown by using ECD comparison of its experimental with the calculated analysis.

Grewiifopene K (**18**) had the same molecular formula of C_38_H_48_O_11_ as **17** based on a sodium ion adduct peak in the HRESIMS spectrum, indicating that **18** is isomeric with **17**. The closely matched ^1^H and ^13^C NMR spectra (Tables 2 and 3) suggested that **18** possessed the same planar structure as **17**, but they have differences in the geometry of the C-12/C-13 tri-substituted alkene. The NOESY cross peaks (Fig. 3) between H-12 and H-14; H-11 and Me-16 and the characteristic ^13^C chemical shift of the olefinic methyl moiety (Me-16;* δ*_C_ 12.1) indicated its *E-*configuration [21]. Both the NOESY experiments (Fig. S114, supporting information) and ^1^H–^1^H coupling constants as well as ECD curves (Fig. 4) suggested that **18** had the same absolute configurations as those of **17**.

### Cytotoxicity, antibacterial activities, and inhibition of aromatase of some isolated compounds

The in vitro cytotoxicity assay toward a panel of human cancer cell lines of several clerodane diterpenoids has been well investigated [3–5, 7, 10, 21, 22]. Thus, the isolates in adequate amounts were tested against MOLT-3 (acute lymphoblastic leukemia), HuCCA-1 (cholangiocarcinoma), HepG2 (hepatocellular carcinoma), A549 (lung adenocarcinoma), HeLa (cervical carcinoma), and MDA-MB-231 (triple negative breast cancer) cancer cell lines using the MTT and XTT methods. Doxorubicin and etoposide were used as positive controls. The cytotoxicity results as shown in Table 4 revealed that the clerodane diterpenoids **7**, **8**, and **16**, with a free hydroxy moiety at C-6 and no substituent at C-7, exhibited strong cytotoxicity with IC_50_ values in the range of 0.3 to 2.9 µM and exhibited a greater cytotoxic activity than those with either the methoxy or acetoxy groups at C-6. These data are consistent with the previous reports that the hydroxy substituted at C-6 was very important for enhancing cytotoxic activity [7, 23]. Cytotoxicity toward A549 cell line of compounds **7** and **16** (both with the IC_50_ = 0.3 μM) and toward MDA-MB-231 cell line of compounds **7**, **8**, and **16**–**18** (IC_50_ = 2.0–2.2 μM) was virtually comparable to that of a standard, etoposide. Furthermore, compounds **8** and **17** were two-fold more efficacious than etoposide against HuCCA-1 cell line. Interestingly, selective cytotoxicity toward MOLT-3 (IC_50_ = 3.7 µM) was observed for **13**, while the other cell lines tested (HuCCA-1, A549, HeLa, and MDA-MB-231) were found to exhibit only weak activities. When evaluated against HepG2, the IC_50_ values of the tested clerodane diterpenoids, except for the compounds **2**, **13**, and **15**, ranged from 1.9 to 14.3 μM; compound **7** (IC_50_ = 1.9 μM) was the most cytotoxic. The tested clerodane diterpenoids, except for **18** and **19**, exhibited moderate cytotoxicity toward MOLT-3 cell line with IC_50_ values ranging from 1.0 to 5.0 µM. Structural differences in the six-carbon conjugated diene side chain, including (*E*)/(*Z*)-3-methylpenta-1,3-diene and 3-methylpenta-1,3-diene at C-9 in compounds **14**, **17**, and **18** did not significantly affect cytotoxicity. Moreover, the replacement of an acetoxy group at C-18 with a methoxy group in compounds **13** and **10** rendered them less active. (−)-Syringaresinol (**19**), a known lignan, was inactive against all cell lines, even at 50 µg/mL. 4-Acetonyl-3,5-dimethoxy-*p*-quinol (**20**) exhibited weak cytotoxicity against only MOLT-3 (IC_50_ = 10.0 μM) and MDA-MB-231 (IC_50_ = 24.4 μM) cell lines.

**Table 4:** Cytotoxicity of compounds **2**, **3**, and **5**–**20**

Compound	Cell lines (IC_50_, μM); values are expressed as mean ± s.d. (n = 3)					
	MOLT-3	HuCCA-1	A549	HeLa	MDA-MB-231	HepG2
**2**	2.7 ± 0.4	8.7 ± 1.1	7.8 ± 0.5	8.8 ± 0.9	9.3 ± 0.4	37.1 ± 4.1
**3**	2.7 ± 0.3	3.9 ± 0.2	5.1 ± 1.1	5.3 ± 0.1	8.4 ± 0.1	13.2 ± 0.5
**5**	3.4 ± 0.4	5.3 ± 0.1	5.0 ± 0.9	5.6 ± 0.3	8.7 ± 0.2	10.0 ± 0.5
**6**	3.2 ± 0.2	5.6 ± 1.2	5.8 ± 0.8	7.3 ± 0.3	10.2 ± 0.6	11.8 ± 1.1
**7**	1.4 ± 0.2	0.7 ± 0.1	0.3 ± 0.02	0.9 ± 0.1	2.0 ± 0.2	1.9 ± 0.4
**8**	1.2 ± 0.2	0.4 ± 0.1	0.6 ± 0.1	1.8 ± 0.2	2.2 ± 0.1	2.4 ± 0.1
**9**	3.0 ± 0.2	5.2 ± 0.4	5.0 ± 0.5	5.2 ± 0.1	8.1 ± 0.4	9.5 ± 0.8
**10**	5.0 ± 1.4	7.5 ± 0.2	8.2 ± 0.8	10.7 ± 0.6	10.4 ± 0.2	13.0 ± 1.0
**11**	5.0 ± 1.7	3.3 ± 0.4	3.0 ± 0.4	10.3 ± 0.5	11.4 ± 0.5	14.3 ± 1.5
**12**	1.7 ± 0.4	4.4 ± 0.4	3.6 ± 0.04	3.8 ± 0.6	7.6 ± 0.9	8.9 ± 0.3
**13**	3.7 ± 1.0	41.4 ± 6.1	30.6 ± 3.9	I^a^	37.0 ± 5.1	44.2 ± 4.4
**14**	1.3 ± 0.5	1.8 ± 0.2	2.6 ± 0.1	2.4 ± 0.2	2.0 ± 0.1	7.3 ± 0.6
**15**	3.1 ± 0.4	6.6 ± 0.6	9.2 ± 1.2	8.8 ± 0.6	9.1 ± 0.3	38.5 ± 2.8
**16**	1.2 ± 0.2	0.7 ± 0.02	0.8 ± 0.01	0.8 ± 0.1	2.0 ± 0.1	2.9 ± 0.0
**17**	1.0 ± 0.1	0.4 ± 0.03	2.4 ± 0.2	2.8 ± 0.03	2.0 ± 0.1	7.1 ± 0.2
**18**	ND^b^	1.6 ± 0.2	2.2 ± 0.2	6.1 ± 0.3	2.5 ± 0.1	8.9 ± 1.2
**19**	75.3 ± 4.9	I	I	I	I	I
**20**	10.0 ± 3.5	111.0 ± 2.2	125.0 ± 4.7	131.7 ± 3.8	24.4 ± 1.2	121.7 ± 11.3
Doxorubicin^d^	0.01 ± 0.002	0.83 ± 0.07	0.38 ± 0.02	0.32 ± 0.01	2.09 ± 0.10	0.52 ± 0.10
Etoposide^d^	0.022 ± 0.004					33.50 ± 2.55

^a^Inactive for cytotoxicity with < 50% inhibition at a concentration of 50 µg/mL

^b^ND not determined

^d^Etoposide and doxorubicin are used as positive controls

In addition, the isolates were also evaluated for their antibacterial activity towards three strains including *Bacillus cereus* (ATCC 11778), *Staphylococcus aureus* (ATCC 6538) and *Staphylococcus epidermidis* (ATCC 12228) using penicillin G and gentamicin as the antibiotic controls (Table 5). However, due to insufficient materials for testing, only compounds **2**, **3**, **7**–**11**, **15**, **19**, and **20** were subjected to evaluation. Most of the tested compounds, except for compound **7**, did not show significant activity towards *S. aureus* and *S. epidermidis*. Compound **7** exhibited antibacterial activity against only *S. aureus* with the MIC and MBC values of 25 (50) µg/mL. Compounds **7**–**11** exhibited antibacterial activity toward *B. cereus* with MIC and MBC values of 6.25 (> 200), 25 (50), 100 (100), 12.5 (12.5), and 12.5 (25.0) μg/mL, respectively. Finally, the inhibitory activity of aromatase by using a CYP19 screening kit was investigated. The aromatase (also called estrogen synthase) is responsible for converting the non-aromatic ring A of androgens to estrogens, which are related to the proliferation of breast cancer cells. However, almost all the tested compounds (**3**, **5**, **7**, **9**, **11**, **12**, **19**, and **20**) did not show activity at the concentration up to 12.15 µM (Table S11, supporting information).

**Table 5:** Antimicrobial activity of compounds **2**, **3**, **7**–**11**, **15**, **19**, and **20** (MIC (MBC), μg/mL)

Compound	*S. aureus* (ATCC 6538)		*S. epidermidis* (ATCC 12228)		*B. cereus* (ATCC 11778)	
	MIC	MBC	MIC	MBC	MIC	MBC
**2**	> 200	> 200	> 200	> 200	100	> 200
**3**	> 200	> 200	> 200	> 200	> 200	> 200
**7**	25	50	100	> 200	6.25	> 200
**8**	> 200	> 200	> 200	> 200	25	50
**9**	> 200	> 200	> 200	> 200	100	100
**10**	> 200	> 200	> 200	> 200	12.5	12.5
**11**	> 200	> 200	> 200	> 200	12.5	25
**15**	> 200	> 200	> 200	> 200	> 200	> 200
**19**	> 200	> 200	> 200	> 200	> 200	> 200
**20**	> 200	> 200	> 200	> 200	> 200	> 200
Penicillin G^a^	0.00076	0.003	0.195	1.56	3.125	3.125
Gentamicin^a^	0.024	0.024	0.024	0.024	0.048	0.048

^a^Penicillin G and gentamicin are used as positive controls

## Conclusions

In summary, a total of twenty isolates, including eleven novel clerodane diterpenoids, grewiifopenes A–K, as well as nine known compounds were isolated from the dichloromethane extract of *C. grewiifolia*. Their structures were determined by extensive spectroscopic analysis. Meanwhile, the absolute stereochemistry was established by comparing the experimental and calculated ECD data. A biological evaluation of some isolated compounds revealed that all tested clerodane diterpenoids possessed cytotoxic activities against a panel of cancer cell lines. On the other hand, only compound **7** exhibited antibacterial activity against *S. aureus*, while compounds **7**–**11** exhibited antibacterial activity against *B. cereus*. None of the tested compounds inhibited the aromatase. To the best of our knowledge, this is the first report on the antibacterial activity and inhibition of aromatase of clerodane diterpenoids from *C. grewiifolia*.

## Experimental

### General experimental procedures

A JASCO P-1020 digital polarimeter measured at 590 nm (Na lamp D line) was used to measure the specific optical rotation values. A JASCO J-815 spectropolarimeter was utilized to provide the ECD spectra. A Perkin–Elmer Spectrum One spectrometer [attenuated total reflectance (ATR)] was used to obtain FT-IR spectra. A Bruker AVANCE 600 spectrometers was used to record 1D and 2D NMR spectra. A Bruker MicroTOFLC spectrometer was used to record the ESITOFMS (positive mode) data. The stationary phases for column chromatography (CC) are silica gel 60 (Merck, 70–200 mesh ASTM), Sephadex LH-20 gel (Ambersham Biosciences), and silica gel 60 RP-18 (Merck, 40–63 µm). Preparative TLC was performed with silica gel 60 PF_254_. TLC plate detection was carried out on silica gel precoated aluminum-backed plate (silica gel 60 F_254_, Merck). A VertiSep™ UPS RP-C18 column (21.2 × 250 mm) on Waters 600 Delta HPLC instrument connected to a Water photodiode array detector was used for purification with the eluent flow rate at 12 mL/min.

### Plant materials

The twigs and stems of *Casearia grewiifolia* Vent. (Salicaceae) were collected from the Sai Yok National Park, Kanchanaburi Province, Thailand (14.2495, 99.0270) in December 2020. One of the authors (T.T.) authenticated the plant, and a voucher specimen (SY16) has been deposited at the Forest Herbarium, Department of National Parks, Wildlife and Plant Conservation, Thailand.

### Extraction and isolation

Air dried twigs and stems powder of *C. grewiifolia* (8.9 kg) were macerated two times with CH_2_Cl_2_ at ambient temperature, and the resulting solvent was concentrated under vacuum to afford 50.0 g of the CH_2_Cl_2_ extract. A 48 g of CH_2_Cl_2_ extract was developed to column chromatography (CC) with silica gel with the mobile phase of hexane–acetone (a gradient from 100:0 to 0:100) and acetone-MeOH (a gradient from 100:0 to 0:100) provide sixteen fractions (A1–A16). The Sephadex LH-20 CC was then applied for fraction A6 (13.6 g) using CH_2_Cl_2_–MeOH in a ratio of 1:1 as eluent to give five fractions (B1–B5). Fraction B3 (2.7 g), was fractionated by silica gel CC with the mobile phase of hexane–EtOAc (a gradient from 100:0 to 0:100) providing seven fractions (C1–C7). A gradient elution of hexane and acetone was applied to silica gel CC for further separated fraction C5 (1.5 g) to provide eleven fractions (D1–D11). Using HPLC approach for purification of fraction D3 (69.0 mg) and eluting with a CH_3_CN–H_2_O (70:30 to 100:0 for 180 min) gave compounds **10** (2.0 mg) and **11** (2.6 mg). Compound** 4** (2.0 mg) was afforded from the purification of fraction D5 (1.5 g) by HPLC, using an isocratic system of CH_3_CN in H_2_O (73:27) as eluent. Compounds **6** (1.3 mg), **9** (25.6 mg), and **5** (28.5 mg) were obtained from fraction D6 (782.5 mg) by the RP 18 HPLC column using an isocratic system of CH_3_CN–H_2_O (72:27) as mobile phase. Fraction B4 (3.4 g) was chromatographed by CC with gel filtration, Sephadex LH-20, eluting with the mobile phase of CH_2_Cl_2_–MeOH in ratio of 1:1., then followed by RP 18 HPLC column, eluting with an isocratic system of CH_3_CN–H_2_O (65:35) to give additional yields of compounds **11** (5.7 mg), **9** (21.5 mg), and** 5** (42.6 mg). A 1.6 g of fraction A8 was subjected by CC with a gel filtration, Sephadex LH-20, eluting with CH_2_Cl_2_–MeOH in a ratio of 1:1 and then purified by silica gel CC with a gradient of MeOH in CH_2_Cl_2_ as eluent to afford sixteen fractions (E1–E16). After purifying fraction E10 (15.9 mg) with RP 18 HPLC column, using a mobile phase of MeOH–H_2_O (a gradient from 60:40 to 80:20 in 140 min), compounds** 3** (19.5 mg) and **15** (8.5 mg) were obtained. The eluent of CH_2_Cl_2_–MeOH (1:1) was applied on Sephadex LH-20 CC for the fractionations of fraction E12 (231.9 mg) to give three fractions (F1–F3). Subsequently, fraction F2 (139.9 mg) was applied on preparative TLC plates, eluting with the mobile phase of hexane:acetone:EtOAc in a ratio of 8:1:1 yielding the three bands (G1–G3). Compound **12** (17.5 mg) was also obtained from band G1, while band G2 (47.4 mg) was subjected to RP 18 HPLC column using a mixture of CH_3_CN in H_2_O (a gradient from 70:30 to 80:20 for 60 min) as eluent to give compounds** 2** (6.4 mg) and **1** (1.8 mg). Compounds **8** (11.0 mg), **7** (29.5 mg), and **16** (6.5 mg) was obtained from fraction E13 (181.2 mg), purifying by RP 18 HPLC column with a gradient elution of CH_3_CN in H_2_O (60:40 to 80:20 for 90 min) at flow rate of 10 mL/min. The Sephadex LH-20 CC with eluent of CH_2_Cl_2_–MeOH (1:1) and silica gel CC with a gradient elution of MeOH in CH_2_Cl_2_ were used for fractionation of fraction A12 (5.9 g), yielding twelve fractions (H1–H12). Fraction H10 (424.4 mg) was applied on preparative TLC plates, eluting with a mixture of hexane:acetone:MeOH in the ratio of 70:30:1 to provide three bands (I1–I3). Band I2 (152.4 mg) was further purified with HPLC with a gradient elution of CH_3_CN in H_2_O (65:35 to 70:30 for 140 min) to afforded compounds **18** (3.0 mg) and **14** (2.5 mg). Compound **17** (8.2 mg) was received from band I3 (198.5 mg) by HPLC, eluting with an isocratic system of CH_3_CN–H_2_O (65:35). Purification of fraction H11 (709.3 mg) with RP 18 HPLC column which was eluted with an isocratic system of CH_3_CN in H_2_O (80:20) afford compounds **14** (20.0 mg) and **13** (6.0 mg). The eluent, a mixture of CH_2_Cl_2_ and MeOH (1:1), was applied to Sephadex LH-20 CC for the purification of fraction A13 (3.06 g) provided four fractions (J1–J4). Further purification of fraction J3 (1.1 g) by silica gel CC with a gradient elution of MeOH in CH_2_Cl_2_ provided seven fractions (K1–K7) were obtained. Compounds **19** (23.5 mg) and **20** (21.0 mg) were purified from fractions K4 (52.0 mg) and K6 (35.0 mg), respectively, were applied on preparative TLC plates, eluting with MeOH:CH_2_Cl_2_ in the ratio of 1:99 and 2:98 respectively.

### Compound characterization

#### Grewiifopene A (1)

Optically active colorless oil; [*α*]_D_^25^ + 29.6 (*c* 0.30, CHCl_3_); ECD (*c* 0.06 μM, acetonitrile) λ_max_ (Δ*ε*): 204 (+ 30.94), 226 (− 0.98), 236 (+ 0.45); IR (ATR) *ν*_max_ 2970, 2933, 1729, 1456, 1373, 1226, 1182, 1138, 1098, 1074, 1019, 951, 897, 733 cm^−1^; HRESIMS (positive mode) *m/z* 613.2990 (calcd for C_32_H_46_NaO_10_ [M+Na]^+^, 613.2983).

#### Grewiifopene B (2)

Optically active colorless oil; [*α*]_D_^25^ + 52.0 (*c* 0.67, CHCl_3_); ECD (*c* 0.06 μM, acetonitrile) λ_max_ (Δ*ε*): 203 (+ 20.34), 223 (+ 0.24), 228 (− 0.29), 243 (+ 0.32); IR (ATR) *ν*_max_ 2959, 2931, 1733, 1457, 1372, 1226, 1184, 1096, 1074, 1019, 998, 979, 950, 897, 734 cm^−1^; HRESIMS (positive mode) *m/z* 613.2981 (calcd for C_32_H_46_NaO_10_ [M+Na]^+^, 613.2983).

#### Grewiifopene C (3)

Optically active colorless oil; [*α*]_D_^25^ + 58.7 (*c* 2.30, CHCl_3_); ECD (*c* 0.06 μM, acetonitrile) λ_max_ (Δ*ε*): 191 (+ 24.76), 227 (+ 0.60), 237 (+ 1.42); IR (ATR) *ν*_max_ 2960, 2930, 1744, 1371, 1217, 1167, 1100, 1071, 1023, 999, 946, 917, 900, 734 cm^−1^; HRESIMS (positive mode) *m/z* 641.2931 (calcd for C_33_H_46_NaO_11_ [M+Na]^+^, 641.2932).

#### Grewiifopene D (4)

Optically active colorless oil; [*α*]_D_^25^ − 108.3 (*c* 0.07, CHCl_3_); ECD (*c* 0.28 μM, acetonitrile) λ_max_ (Δ*ε*): 200 (− 2.33), 220 (− 0.34); IR (ATR) *ν*_max_ 2965, 2930, 1755, 1731, 1454, 1374, 1225, 1179, 1151, 1100, 1076, 1024, 995, 936, 898 cm^−1^; HRESIMS (positive mode) *m/z* 555.2932 (calcd for C_30_H_44_NaO_8_ [M+Na]^+^, 555.2928).

#### Grewiifopene E (12)

Optically active colorless oil; [*α*]_D_^25^ + 66.2 (*c* 1.72, CHCl_3_); ECD (*c* 0.05 μM, acetonitrile) λ_max_ (Δ*ε*): 200 (+ 28.49), 229 (+ 0.70), 236 (+ 1.17), 238 (+ 1.05), 250 (+ 0.62); IR (ATR) *ν*_max_ 3503, 2959, 2927, 1733, 1465, 1457, 1371, 1291, 1226, 1188, 1167, 1093, 1023, 996, 956, 902, 735 cm^−1^; HRESIMS (positive mode) *m/z* 641.3303 (calcd for C_34_H_50_NaO_10_ [M+Na]^+^, 641.3296).

#### Grewiifopene F (13)

Optically active colorless oil; [*α*]_D_^25^ + 26.8 (*c* 0.41, CHCl_3_); ECD (*c* 0.06 μM, acetonitrile) λ_max_ (Δ*ε*): 200 (+ 6.29), 229 (− 0.22), 279 (+ 0.61), 312 (+ 0.90); IR (ATR) *ν*_max_ 3388, 2957, 2925, 2854, 1724, 1632, 1604, 1515, 1446, 1372, 1264, 1168, 1097, 1044, 1006, 991, 948, 900, 833, 731 cm^−1^; HRESIMS (positive mode) *m/z* 675.3138 (calcd for C_37_H_48_NaO_10_ [M+Na]^+^, 675.3140).

#### Grewiifopene G (14)

Optically active colorless oil; [*α*]_D_^25^ + 54.1 (*c* 0.39, CHCl_3_); ECD (*c* 0.07 μM, acetonitrile) λ_max_ (Δ*ε*): 190 (+ 15.69), 201 (+ 7.82), 206 (+ 7.93), 231 (+ 0.31), 299 (+ 0.72), 340 (+ 0.02); IR (ATR) *ν*_max_ 3406, 2960, 2928, 1711, 1632, 1604, 1515, 1443, 1373, 1227, 1168, 1094, 1024, 996, 903, 833, 735 cm^−1^; HRESIMS (positive mode) *m/z* 703.3098 (calcd for C_38_H_48_NaO_11_ [M+Na]^+^, 703.3089).

#### Grewiifopene H (15)

Optically active colorless oil; [*α*]_D_^25^ + 58.7 (*c* 0.98, CHCl_3_); ECD (*c* 0.06 μM, acetonitrile) λ_max_ (Δ*ε*): 196 (+ 23.82), 239 (− 1.61), 259 (+ 0.47); IR (ATR) *ν*_max_ 2963, 2930, 1748, 1434, 1370, 1216, 1105, 1071, 1060, 999, 982, 962, 931, 917 cm^−1^; HRESIMS (positive mode) *m/z* 641.2943 (calcd for C_33_H_46_NaO_11_ [M+Na]^+^, 641.2932).

#### Grewiifopene I (16)

Optically active colorless oil; [*α*]_D_^25^ + 40.7 (*c* 0.62, CHCl_3_); ECD (*c* 0.07 μM, acetonitrile) λ_max_ (Δ*ε*): 204 (+ 19.80), 246 (− 0.30), 256 (+ 0.26); IR (ATR) *ν*_max_ 3456, 2965, 2930, 2878, 1749, 1729, 1461, 1373, 1227, 1177, 1150, 1099, 1074, 1023, 1002, 959, 946, 925, 890, 735 cm^−1^; HRESIMS (positive mode) *m/z* 541.2782 (calcd for C_29_H_42_NaO_8_ [M+Na]^+^, 541.2772).

#### Grewiifopene J (17)

Optically active colorless oil; [*α*]_D_^25^ + 116.7 (*c* 0.46, CHCl_3_); ECD (*c* 0.06 μM, acetonitrile) λ_max_ (Δ*ε*): 209 (+ 9.20), 238 (− 5.48), 290 (+ 2.36); IR (ATR) *ν*_max_ 3407, 2962, 2929, 1727, 1632, 1604, 1515, 1441, 1371, 1329, 1226, 1168, 1093, 1024, 998, 981, 960, 918, 891, 833, 734 cm^−1^; HRESIMS (positive mode) *m/z* 703.3090 (calcd for C_38_H_48_NaO_11_ [M+Na]^+^, 703.3089).

#### Grewiifopene K (18)

Optically active colorless oil; [*α*]_D_^25^ + 74.0 (*c* 0.29, CHCl_3_); ECD (*c* 0.09 μM, acetonitrile) λ_max_ (Δ*ε*): 198 (+ 8.60), 223 (+ 0.10), 236 (− 1.91); IR (ATR) *ν*_max_ 3374, 2958, 2926, 1728, 1632, 1604, 1515, 1448, 1372, 1227, 1161, 1095, 1025, 998, 894, 834, 736 cm^−1^; HRESIMS (positive mode) *m/z* 703.3090 (calcd for C_38_H_48_NaO_11_ [M+Na]^+^, 703.3089).

### Preparation of 2-methylbutyric acid benzyl esters

A solution of benzyl alcohol (31.8 mg, 0.3 mmol) in CH_2_Cl_2_ 1 mL was added to *R*/*S*-2-methylbutanoic acid (20 mg, 0.2 mmol), 1-ethyl-3-(3-dimethylaminopropyl)carbodiimide (EDC) (57 mg, 0.3 mmol), and dimethylaminopyridine (DMAP) (1 mg, 0.008 mmol). The reaction mixture was stirred at room temperature for 3 h, then evaporated and purified by PTLC with 5% EtOAc-hexnae to afford *R*/*S*-2-methylbutyric acid benzyl esters (32 mg, 85%): colorless oil; ^1^H NMR (400 MHz, CDCl_3_) 7.37–7.29 (m, H-2–H-6), 5.12 (s, H_2_-7), 2.45 (sext, *J* = 7.0 Hz, H-2′), 1.70 (m, *J* = 7.4 Hz H-3′a), 1.49 (m, H-3′b), 1.16 (d, *J* = 7.0 Hz, Me-5′), 0.89 (t, *J* = 7.5 Hz, Me-4′); ^13^C NMR (100 MHz, CDCl_3_): 176.6 (C-1′), 136.3 (C-1), 128.5 (C-2, C-6), 128.07 (C-3, C-5), 128.1 (C-4), 66.0 (C-7), 41.1 (C-2′), 26.8 (C-3′), 16.6 (C-5′), 11.6 (C-4′) (Figs. S119 and S120, Supporting Information). The corresponding *S*-2-methylbutyric acid benzyl ester (30 mg, 85%) was obtained by the same procedure using (*S*)-2-methylbutanoic acid.

### Transesterification of compound 5

Corymbulosin N (**5**) (28.1 mg, 0.053 mmol), benzyl alcohol (0.5 mL, 4.8 mmol), and 4-dimethylaminopyridine (0.65 mg, 0.053 mmol) was heated to 120 °C in seal tube and stirred for 48 h. The reaction was allowed to cool to room temperature and then was purified with column chromatography with 5% EtOAc in hexane as eluent to remove a large amount of benzyl alcohol. The resulting was analyzed by chiral HPLC.

### ECD computational methods

Spartan’ 20 with MMFF94 molecular mechanics model (within 5 kcal/mol energy window) was used for a conformational search and Guassian 16 Rev. C.01 program was used for all DFT calculations [24, 25]. Further optimization of the obtained low-energy conformers was calculated using wB97XD/cc-PVDZ level of theory with IEFPCM of methanol solvent model. All optimized conformers were confirmed to be a true minimum of electronic potential energy by means of the vibrational frequency calculation at the same level without any detection of imaginary frequencies. Each conformer with the over 2% population was subjected to the ECD calculations, according to Boltzmann distribution law base on Gibbs free energies. Using TD-DFT calculations at the CAM-B3LYP/def2-SVP level of theory and the application of the IEFPCM of methanol solvent model [26] were computed the simulated ECD spectra of **1**, **4**, **8**, and **15**. Thirty excited states of each conformer were calculated and the resulting ECD curves were developed using SpecDis with Boltzmann average all conformers and overlapping Gaussian function with an exponential half-width (σ = 0.35) [27, 28]. For the related enantiomers of **1**, **4**, **8**, and **15**, their theoretical ECD spectra derived from the direct inversion of their simulated ECD spectra.

### Cytotoxicity assays

The MTT (3-(4,5-dimethylthiazol-2-yl)-2,5-diphenyltetrazolium bromide) assay [29] was employed for the cytotoxic evaluation against five human cancer cell lines, including HepG2, A549, MDA-MB-231, HeLa, and HuCCA-1, while the XTT (2,3-bis-(2-methoxy-4-nitro-5-sulphenyl)-(2*H*)-tetrazolium-5-carboxanilide) method [30] was used for MOLT-3 cancer cell. Doxorubicin and etoposide were used as positive controls (Table 4).

### Antibacterial susceptibility testing

#### Tested microorganisms

The tested strains, *Staphylococcus aureus* (*S. aureus*) ATCC6538, *Staphylococcus epidermidis* (*S. epidermidis*) ATCC12228, and *Bacillus cereus* (*B. cereus*) ATCC11778 were obtained from the American Type Culture Collection (ATCC).

#### Inoculum preparation

All tested species were grown on Mueller Hinton Agar (MHA) and incubated at 37 °C for 18–24 h. The bacterial colonies suspension in sterile normal saline was diluted until they reached a 1 × 10^6^ CFC/mL concentration, which was then compared to the McFarland scale. This step provided a standard bacterial suspension (1 × 10^6^ CFU/mL), which was used for the following assays.

#### Determination of minimum inhibitory concentration (MIC) and minimum bactericidal concentration (MBC)

The broth microdilution technique was used for the MIC and MBC determination, which was expressed according to Clinical and Laboratory Standards Institute (CLSI) standard M07 guideline [31]. The compounds were prepared as a stock solution by dissolving in dimethyl sulfoxide (DMSO) with the concentration ranging from 0.195 to 200 µg/mL. DMSO and penicillin G or gentamicin were used as normal and standard drug control. The minimum inhibitory concentration (MIC) refers to the lowest antibacterial compound concentration at which no visual growth was observed, while the complete death of bacteria compared to the initial bacterial inoculum refers to the MBC (minimum bactericidal concentration). Three replicates were performed for each experiment.

### Aromatase (CYP19) inhibition assay

The aromatase assay used a method previously designed by Stresser and co-workers [32]. Letrozole, a positive control, exhibited an IC_50_ value of 1.4 ± 0.3 nM for CYP19 inhibition.

## Supplementary Information

Additional file 1. The NMR, HRESIMS, IR, ECD spectra, chiral HPLC analysis of 2-methylbutyric acid benzyl ester derived from transesterification of **5** and synthetic samples, ECD calculation details of new compounds and the aromatase inhibition data of **3**, **5**, **7**, **9**, **11**, **12**, **19**, and **20**.

## Data Availability

All relevant data supporting the results are provide in the manuscript and its Additional files.
