# Supplementary material for: Grewiifopenes A–K, bioactive clerodane diterpenoids from Casearia grewiifolia Vent
Source: Nat Prod Bioprospect. 2024 Sep 14;14(1):54. doi: 10.1007/s13659-024-00475-7 (PMC11401813; doi:10.1007/s13659-024-00475-7)
Supplement: Supplementary file 1 — Additional file 1. The NMR, HRESIMS, IR, ECD spectra, chiral HPLC analysis of 2-methylbutyric acid benzyl ester derived from transesterification of 5 and synthetic samples, ECD calculation details of new compounds and the aromatase inhibition data of 3, 5, 7, 9, 11, 12, 19, and 20. [file 13659_2024_475_MOESM1_ESM.pdf]

Supporting Information

Grewiifopenes A-K, bioactive clerodane diterpenoids from *Casearia grewiifolia* Vent.

Phanruethai Pailee [1], Paratchata Batsomboon [1], Wiriya Yaosanit [1], Theerawat Thananthaisong [2], Chulabhorn Mahidol [1,3], Poonsakdi Ploypradith [1,3,4], Nanthawan Rek-ngam [1], Panita Khlaychan [1], Supanna Techasakul [1], Somsak Ruchirawat [1,3,4], and Vilailak Prachyawarakorn [1,*]

[1] Laboratory of Natural Products, Medicinal Chemistry and Organic Synthesis, Chulabhorn Research Institute, 54 Kamphaeng Phet 6 Road, Laksi, Bangkok 10210, Thailand

[2] Forest Herbarium, Department of National Parks, Wildlife and Plant Conservation, 61 Phahonyothin Road, Chatuchak, Bangkok 10900, Thailand

[3] Program in Chemical Sciences, Chulabhorn Graduate Institute, 54 Kamphaeng Phet 6 Road, Laksi, Bangkok 10210, Thailand

[4] Center of Excellence on Environmental Health and Toxicology (EHT), Office of the Permanent Secretary (OPS), Ministry of Higher Education, Science, Research and Innovation (MHESI), Bangkok 10400, Thailand

* Corresponding author vilailak@cri.or.th (V. Prachyawarakorn); Tel.: +66-2-553-8555 (ext. 3070)

Contents

Page

Figure S1. ^{1}H-NMR spectrum (600 MHz, $CDCl_3$) of compound **1** 7

Figure S2. ^{13}C-NMR spectrum (150 MHz, $CDCl_3$) of compound **1** 7

Figure S3. DEPT spectrum (150 MHz, $CDCl_3$) of compound **1** 8

Figure S4. ^{1}H-^{1}H COSY spectrum (600 MHz, $CDCl_3$) of compound **1** 8

Figure S5. HSQC spectrum (600 MHz, $CDCl_3$) of compound **1** 9

Figure S6. HMBC spectrum (600 MHz, $CDCl_3$) of compound **1** 9

Figure S7. NOESY spectrum (600 MHz, $CDCl_3$) of compound **1** 10

Figure S8. CD spectrum of compound **1** 11

Figure S9. IR spectrum of compound **1** 12

Figure S10. ESITOFMS spectrum of compound **1** 13

Figure S11. ^{1}H-NMR spectrum (600 MHz, $CDCl_3$) of compound **2** 14

Figure S12. ^{13}C-NMR spectrum (150 MHz, $CDCl_3$) of compound **2** 14

Figure S13. DEPT spectrum (150 MHz, $CDCl_3$) of compound **2** 15

Figure S14. ^{1}H-^{1}H COSY spectrum (600 MHz, $CDCl_3$) of compound **2** 15

Figure S15. HSQC spectrum (600 MHz, $CDCl_3$) of compound **2** 16

Figure S16. HMBC spectrum (600 MHz, $CDCl_3$) of compound **2** 16

Figure S17. NOESY spectrum (600 MHz, $CDCl_3$) of compound **2** 17

Figure S18. CD spectrum of compound **2** 18

Figure S19. IR spectrum of compound **2** 19

Figure S20. ESITOFMS spectrum of compound **2** 20

Figure S21. ^{1}H-NMR spectrum (600 MHz, $CDCl_3$) of compound **3** 21

Figure S22. ^{13}C-NMR spectrum (150 MHz, $CDCl_3$) of compound **3** 21

Figure S23. DEPT spectrum (150 MHz, $CDCl_3$) of compound **3** 22

Figure S24. ^{1}H-^{1}H COSY spectrum (600 MHz, $CDCl_3$) of compound **3** 22

Figure S25. HSQC spectrum (600 MHz, $CDCl_3$) of compound **3** 23

Figure S26. HMBC spectrum (600 MHz, $CDCl_3$) of compound **3** 23

Figure S27. NOESY spectrum (600 MHz, $CDCl_3$) of compound **3** 24

Figure S28. CD spectrum of compound **3** 25

Figure S29. IR spectrum of compound **3** 26

Figure S30. ESITOFMS spectrum of compound **3** 26

Figure S31. ^{1}H-NMR spectrum (600 MHz, C_6D_6) of compound **3** 27

Figure S32. ^{13}C-NMR spectrum (150 MHz, C_6D_6) of compound **3** 27
Figure S33. DEPT spectrum (150 MHz, C_6D_6) of compound **3** 28
Figure S34. ^{1}H-^{1}H COSY spectrum (600 MHz, C_6D_6) of compound **3** 28
Figure S35. HSQC spectrum (600 MHz, C_6D_6) of compound **3** 29
Figure S36. HMBC spectrum (600 MHz, C_6D_6) of compound **3** 29
Figure S37. NOESY spectrum (600 MHz, C_6D_6) of compound **3** 30
Figure S38. ^{1}H-NMR spectrum (600 MHz, $CDCl_3$) of compound **4** 31
Figure S39. ^{13}C-NMR spectrum (150 MHz, $CDCl_3$) of compound **4** 31
Figure S40. DEPT spectrum (150 MHz, $CDCl_3$) of compound **4** 32
Figure S41. ^{1}H-^{1}H COSY spectrum (600 MHz, $CDCl_3$) of compound **4** 32
Figure S42. HSQC spectrum (600 MHz, $CDCl_3$) of compound **4** 33
Figure S43. HMBC spectrum (600 MHz, $CDCl_3$) of compound **4** 33
Figure S44. NOESY spectrum (600 MHz, $CDCl_3$) of compound **4** 34
Figure S45. CD spectrum of compound **4** 35
Figure S46. IR spectrum of compound **4** 36
Figure S47. ESITOFMS spectrum of compound **4** 36
Figure S48. ^{1}H-NMR spectrum (600 MHz, $CDCl_3$) of compound **12** 37
Figure S49. ^{13}C-NMR spectrum (150 MHz, $CDCl_3$) of compound **12** 37
Figure S50. DEPT spectrum (150 MHz, $CDCl_3$) of compound **12** 38
Figure S51. ^{1}H-^{1}H COSY spectrum (600 MHz, $CDCl_3$) of compound **12** 38
Figure S52. HSQC spectrum (600 MHz, $CDCl_3$) of compound **12** 39
Figure S53. HMBC spectrum (600 MHz, $CDCl_3$) of compound **12** 39
Figure S54. NOESY spectrum (600 MHz, $CDCl_3$) of compound **12** 40
Figure S55. CD spectrum of compound **12** 41
Figure S56. IR spectrum of compound **12** 42
Figure S57. ESITOFMS spectrum of compound **12** 42
Figure S58. ^{1}H-NMR spectrum (600 MHz, $CDCl_3$) of compound **13** 43
Figure S59. ^{13}C-NMR spectrum (150 MHz, $CDCl_3$) of compound **13** 43
Figure S60. DEPT spectrum (150 MHz, $CDCl_3$) of compound **13** 44
Figure S61. ^{1}H-^{1}H COSY spectrum (600 MHz, $CDCl_3$) of compound **13** 44
Figure S62. HSQC spectrum (600 MHz, $CDCl_3$) of compound **13** 45
Figure S63. HMBC spectrum (600 MHz, $CDCl_3$) of compound **13** 45
Figure S64. NOESY spectrum (600 MHz, $CDCl_3$) of compound **13** 46

Figure S65. CD spectrum of compound **13** 47
Figure S66. IR spectrum of compound **13** 48
Figure S67. ESITOFMS spectrum of compound **13** 48
Figure S68. ^{1}H-NMR spectrum (600 MHz, $CDCl_3$) of compound **14** 49
Figure S69. ^{13}C-NMR spectrum (150 MHz, $CDCl_3$) of compound **14** 49
Figure S70. DEPT spectrum (150 MHz, $CDCl_3$) of compound **14** 50
Figure S71. ^{1}H-^{1}H COSY spectrum (600 MHz, $CDCl_3$) of compound **14** 50
Figure S72. HSQC spectrum (600 MHz, $CDCl_3$) of compound **14** 51
Figure S73. HMBC spectrum (600 MHz, $CDCl_3$) of compound **14** 51
Figure S74. NOESY spectrum (600 MHz, $CDCl_3$) of compound **14** 52
Figure S75. CD spectrum of compound **14** 53
Figure S76. IR spectrum of compound **14** 54
Figure S77. ESITOFMS spectrum of compound **14** 54
Figure S78. ^{1}H-NMR spectrum (600 MHz, $CDCl_3$) of compound **15** 55
Figure S79. ^{13}C-NMR spectrum (150 MHz, $CDCl_3$) of compound **15** 55
Figure S80. DEPT spectrum (150 MHz, $CDCl_3$) of compound **15** 56
Figure S81. ^{1}H-^{1}H COSY spectrum (600 MHz, $CDCl_3$) of compound **15** 57
Figure S82. HSQC spectrum (600 MHz, $CDCl_3$) of compound **15** 57
Figure S83. HMBC spectrum (600 MHz, $CDCl_3$) of compound **15** 58
Figure S84. NOESY spectrum (600 MHz, $CDCl_3$) of compound **15** 59
Figure S85. CD spectrum of compound **15** 59
Figure S86. IR spectrum of compound **15** 60
Figure S87. ESITOFMS spectrum of compound **15** 61
Figure S88. ^{1}H-NMR spectrum (600 MHz, $CDCl_3$) of compound **16** 62
Figure S89. ^{13}C-NMR spectrum (150 MHz, $CDCl_3$) of compound **16** 62
Figure S90. DEPT spectrum (150 MHz, $CDCl_3$) of compound **16** 63
Figure S91. ^{1}H-^{1}H COSY spectrum (600 MHz, $CDCl_3$) of compound **16** 63
Figure S92. HSQC spectrum (600 MHz, $CDCl_3$) of compound **16** 64
Figure S93. HMBC spectrum (600 MHz, $CDCl_3$) of compound **16** 64
Figure S94. NOESY spectrum (600 MHz, $CDCl_3$) of compound **16** 65
Figure S95. CD spectrum of compound **16** 66
Figure S96. IR spectrum of compound **16** 67
Figure S97. ESITOFMS spectrum of compound **16** 68

Figure S98. ^{1}H-NMR spectrum (600 MHz, $CDCl_3$) of compound **17** 69

Figure S99. ^{13}C-NMR spectrum (150 MHz, $CDCl_3$) of compound **17** 69

Figure S100. DEPT spectrum (150 MHz, $CDCl_3$) of compound **17** 70

Figure S101. ^{1}H-^{1}H COSY spectrum (600 MHz, $CDCl_3$) of compound **17** 70

Figure S102. HSQC spectrum (600 MHz, $CDCl_3$) of compound **17** 71

Figure S103. HMBC spectrum (600 MHz, $CDCl_3$) of compound **17** 71

Figure S104. NOESY spectrum (600 MHz, $CDCl_3$) of compound **17** 72

Figure S105. CD spectrum of compound **17** 73

Figure S106. IR spectrum of compound **17** 74

Figure S107. ESITOFMS spectrum of compound **17** 75

Figure S108. ^{1}H-NMR spectrum (600 MHz, $CDCl_3$) of compound **18** 76

Figure S109. ^{13}C-NMR spectrum (150 MHz, $CDCl_3$) of compound **18** 76

Figure S110. DEPT spectrum (150 MHz, $CDCl_3$) of compound **18** 77

Figure S111. ^{1}H-^{1}H COSY spectrum (600 MHz, $CDCl_3$) of compound **18** 77

Figure S112. HSQC spectrum (600 MHz, $CDCl_3$) of compound **18** 78

Figure S113. HMBC spectrum (600 MHz, $CDCl_3$) of compound **18** 78

Figure S114. NOESY spectrum (600 MHz, $CDCl_3$) of compound **18** 79

Figure S115. CD spectrum of compound **18** 80

Figure S116. IR spectrum of compound **18** 81

Figure S117. ESITOFMS spectrum of compound **18** 81

Figure S118. Chiral HPLC analysis of 2-methylbutyric acid benzyl ester derived from transesterification of **5** and synthetic samples 82

Figure S119. ^{1}H-NMR spectrum (400 MHz, $CDCl_3$) of *R*/*S*-2methylbutyric acid benzyl ester 83

Figure S120. 13C-NMR spectrum (100 MHz, $CDCl_3$) of *R*/*S*-2methylbutyric acid benzyl ester 83

Table S1. Conformational analysis of Compound **1** 84

Table S2. Conformational analysis of Compound **4** 84

Table S3. Conformational analysis of Compound **13** 84

Table S4. Conformational analysis of Compound **14** 84

Table S5. Conformational analysis of Compound **17** 85

Table S6. Coordinates of Compound **1** 85

Table S7. Coordinates of Compound **4** 88

Table S8. Coordinates of Compound **13** 96

Table S9. Coordinates of Compound **14** 100

Table S10. Coordinates of Compound **17** 105

Table S11. Aromatase inhibition data of compounds at a concentration of 12.5 µM from the CH_2Cl_2 extract of *C. grewiifolia* 107

Compound **1**

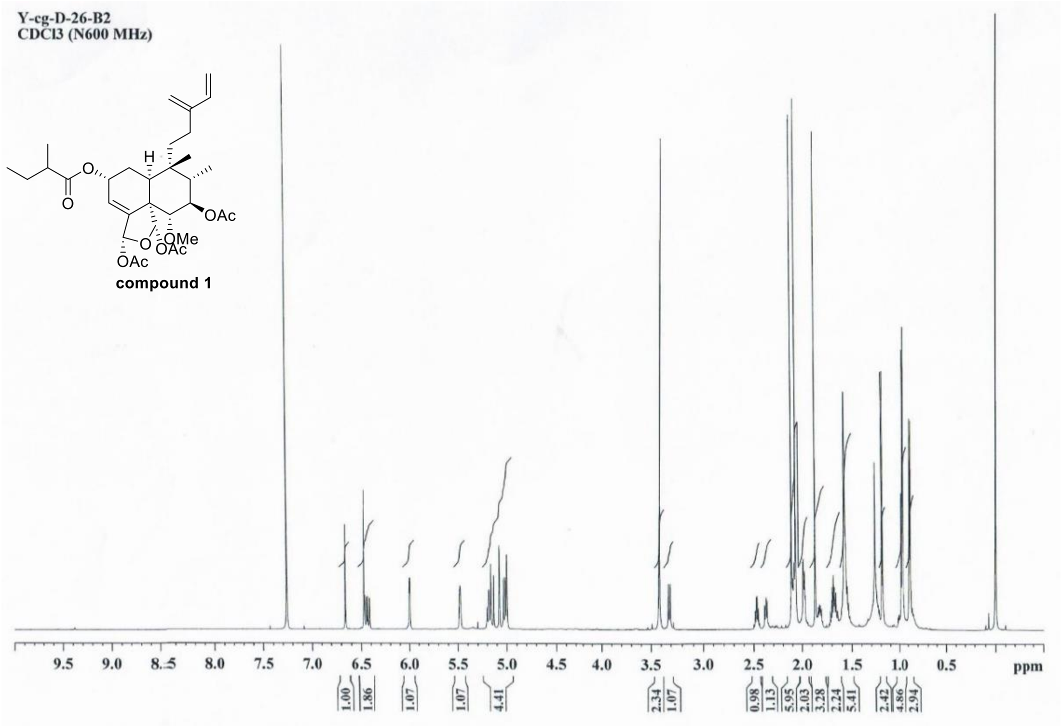

Figure S1. ^{1}H-NMR spectrum (600 MHz, $CDCl_3$) of compound **1**

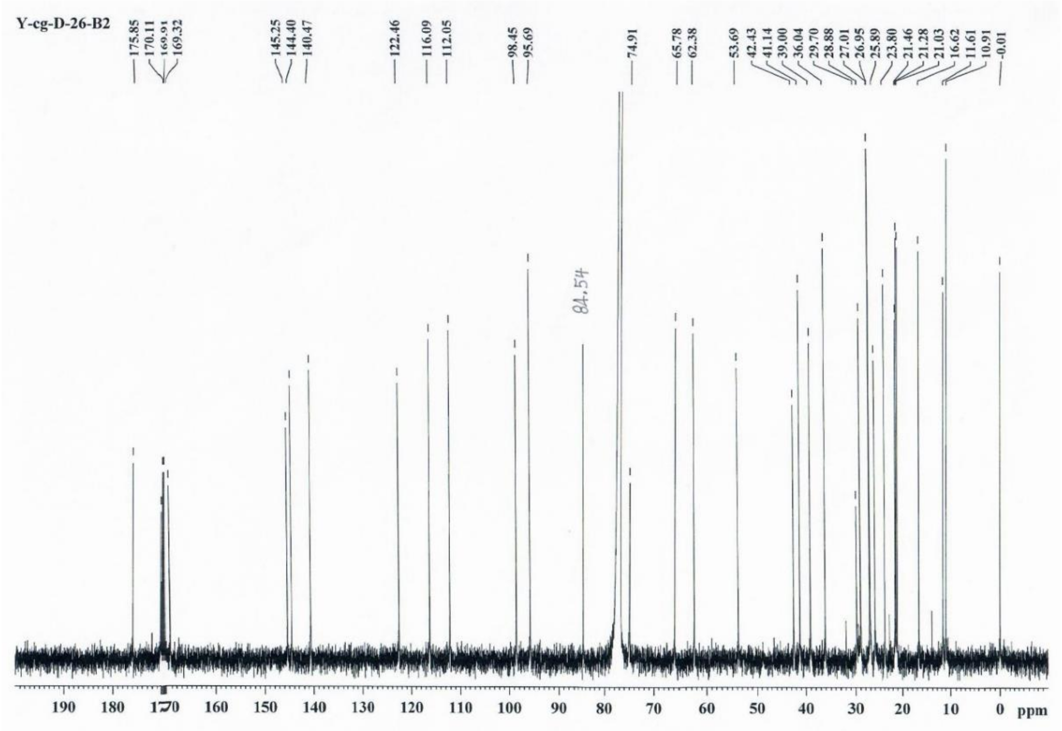

Figure S2. ^{13}C-NMR spectrum (150 MHz, $CDCl_3$) of compound **1**

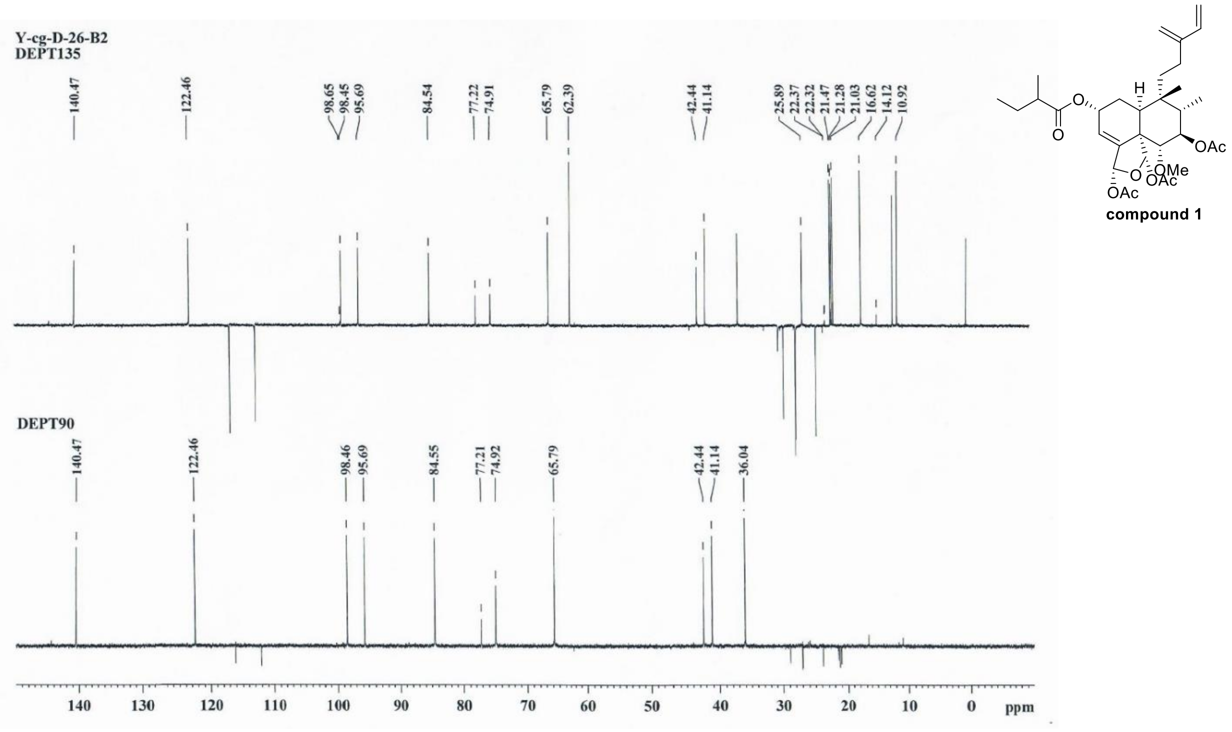

Figure S3. DEPT spectrum (600 MHz, $CDCl_3$) of compound **1**

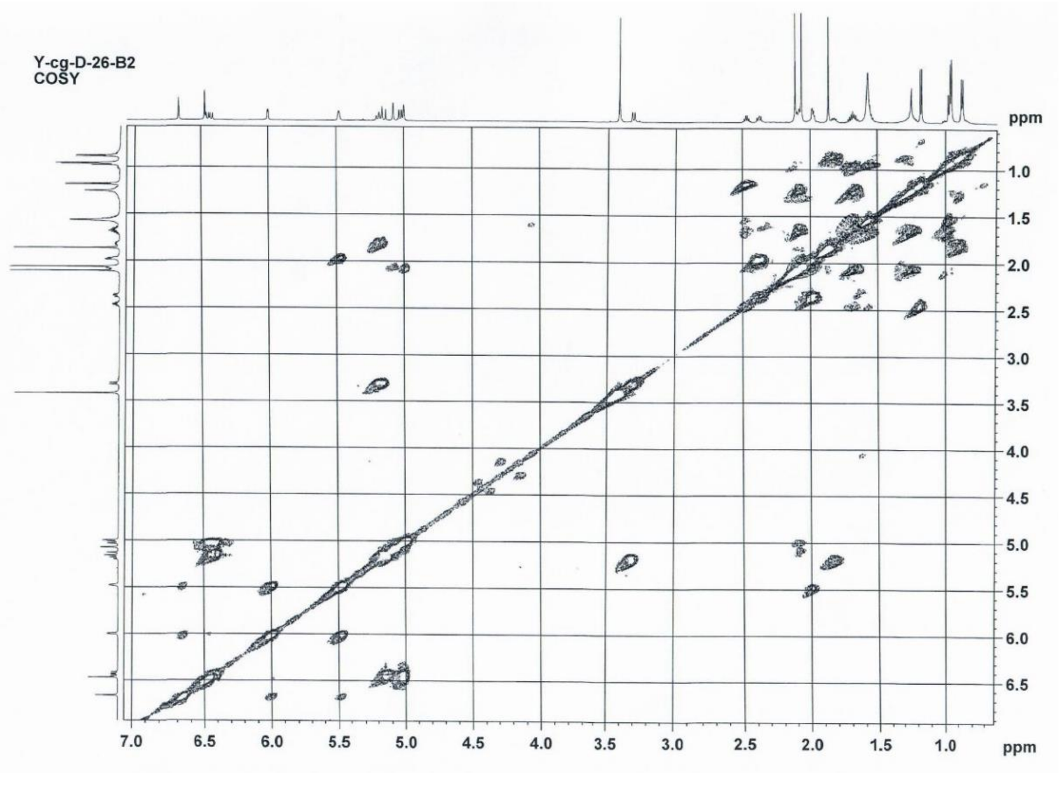

Figure S4. ^{1}H-^{1}H COSY spectrum (600 MHz, $CDCl_3$) of compound **1**

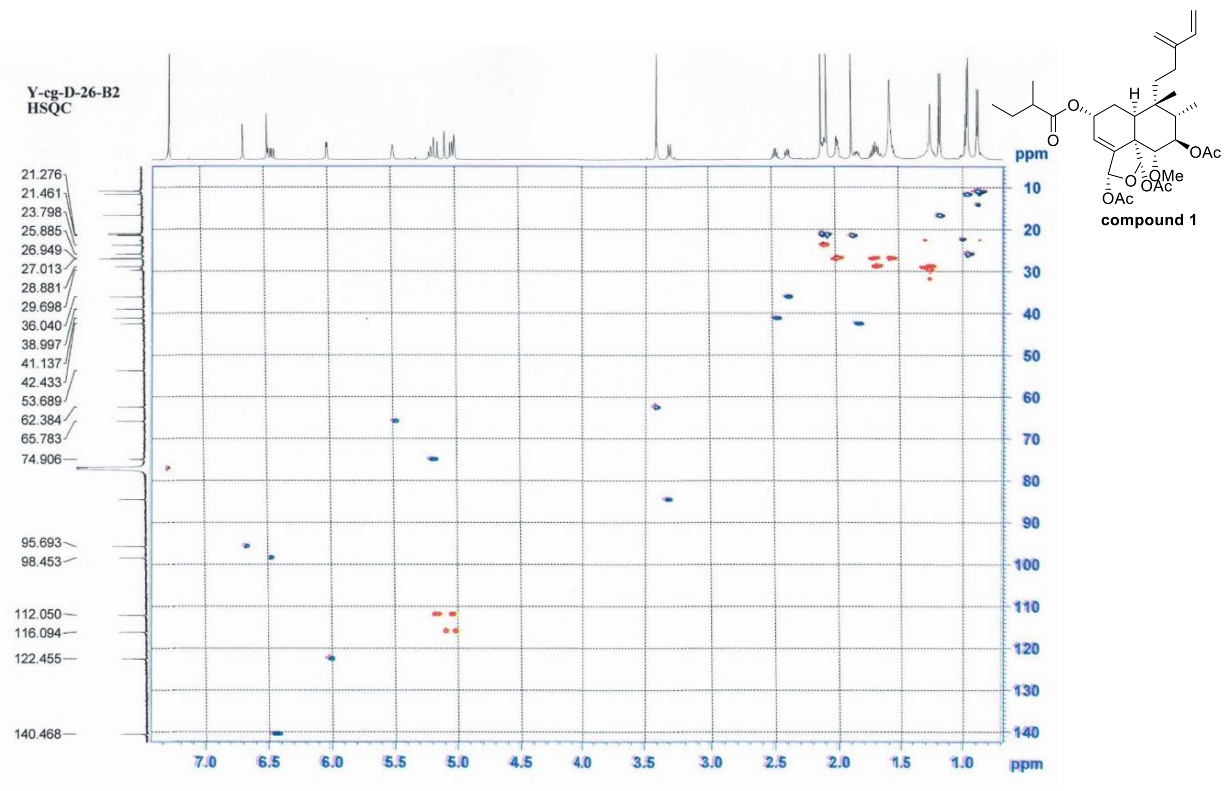

Figure S5. HSQC spectrum (600 MHz, $CDCl_3$) of compound **1**

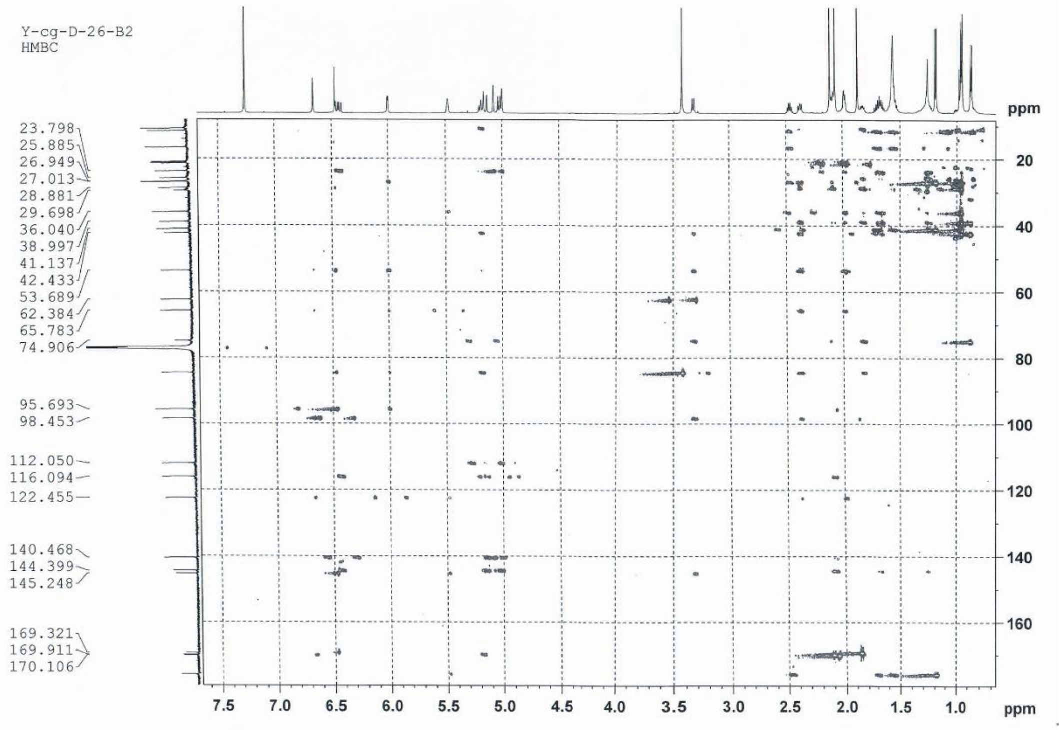

Figure S6. HMBC spectrum (600 MHz, $CDCl_3$) of compound **1**

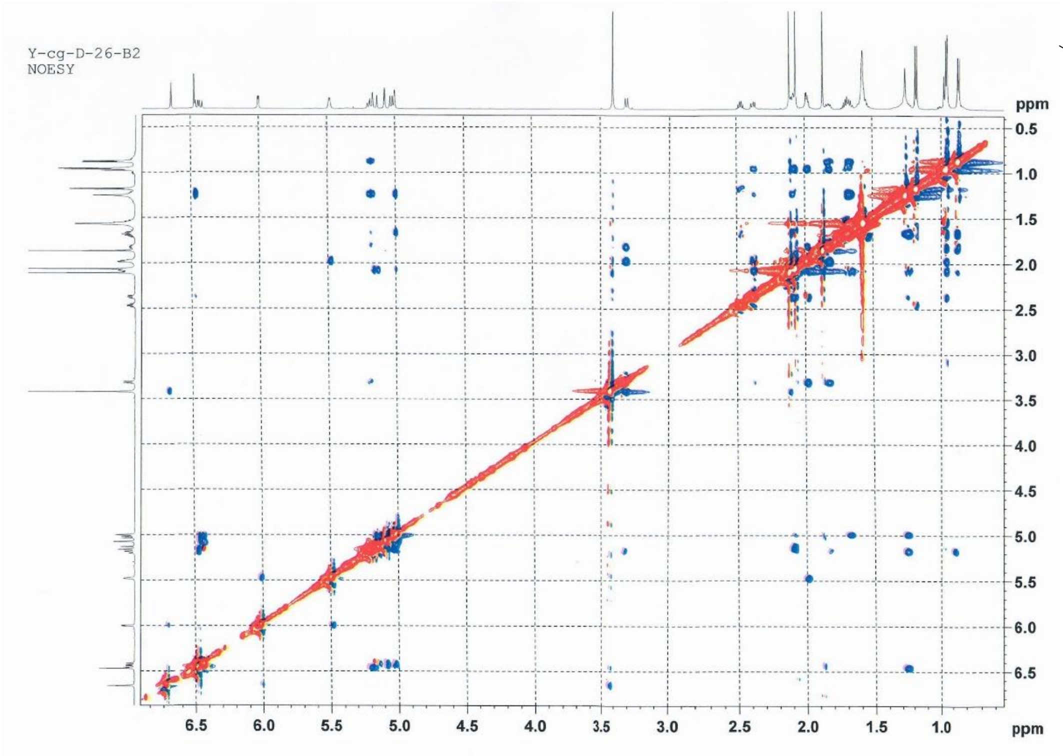

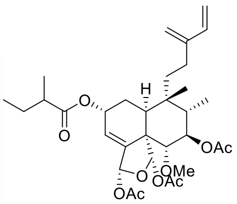

compound 1

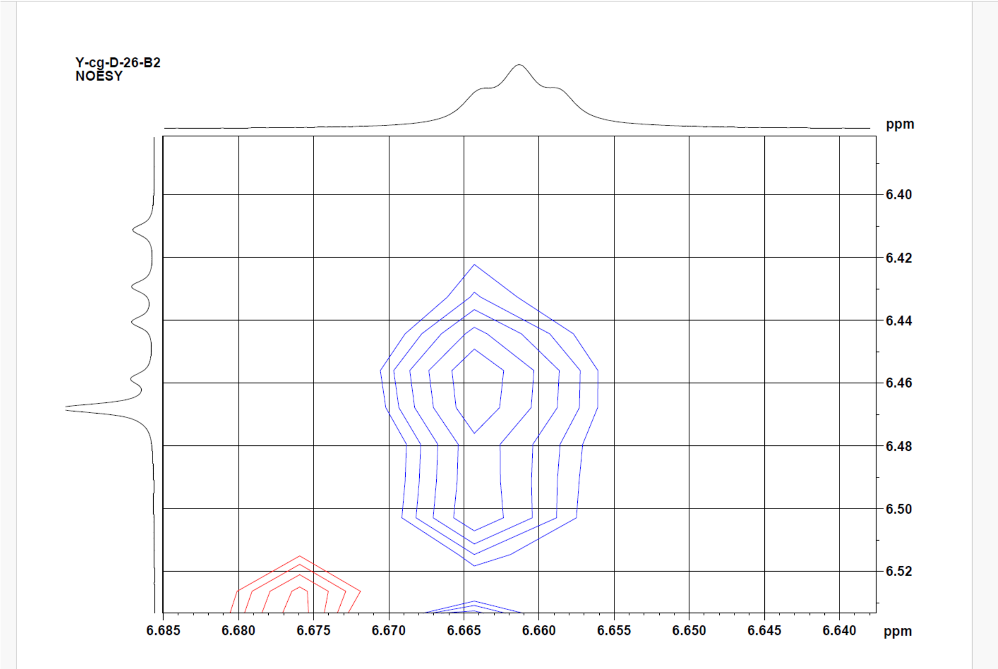

Figure S7. NOESY spectrum (600 MHz, $CDCl_3$) of compound **1**

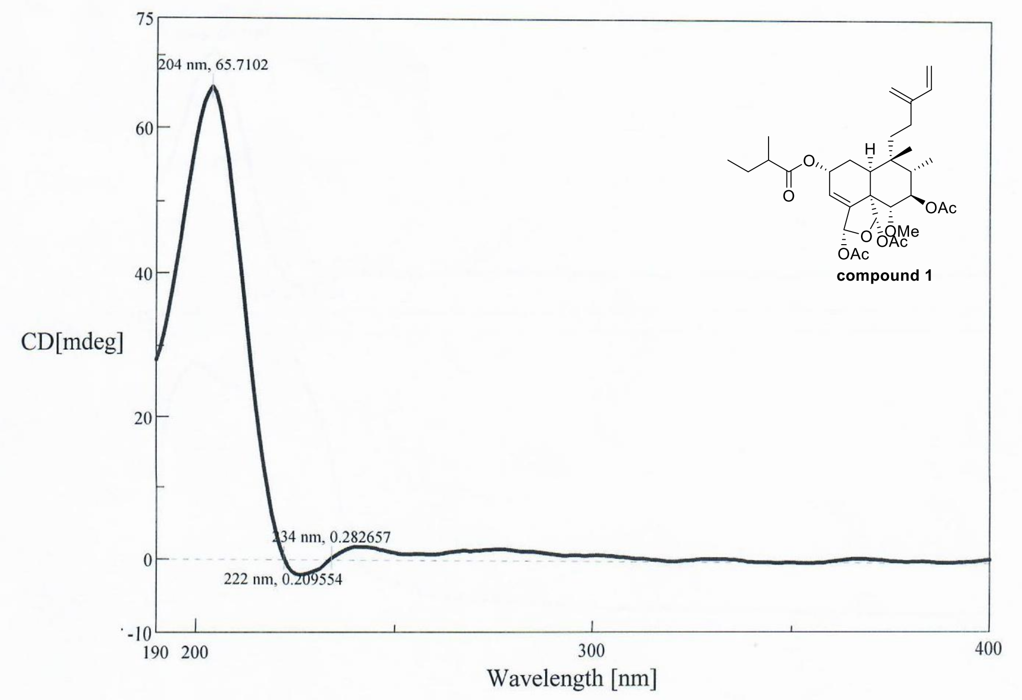

Date	8/5/2022 4:14PM
File name	Y-cg-D-26-B2 original dilution_2
Model	J-815
Serial No.	A024561168
Band width	1 nm
Response	1 sec
Sensitivity	Standard
Measurement range	400 - 190 nm
Data pitch	1nm
Scanning speed	200 nm/min
Accumulation	2
Cell Length	1 cm
Solvent	ACN
Temperature	25 C
Sample name	Y-CG-D-26-B2
Operator	Vilailak
Comment	2.3 mg/2ml--200ul/5800ul

Figure S8. CD spectrum of compound **1**

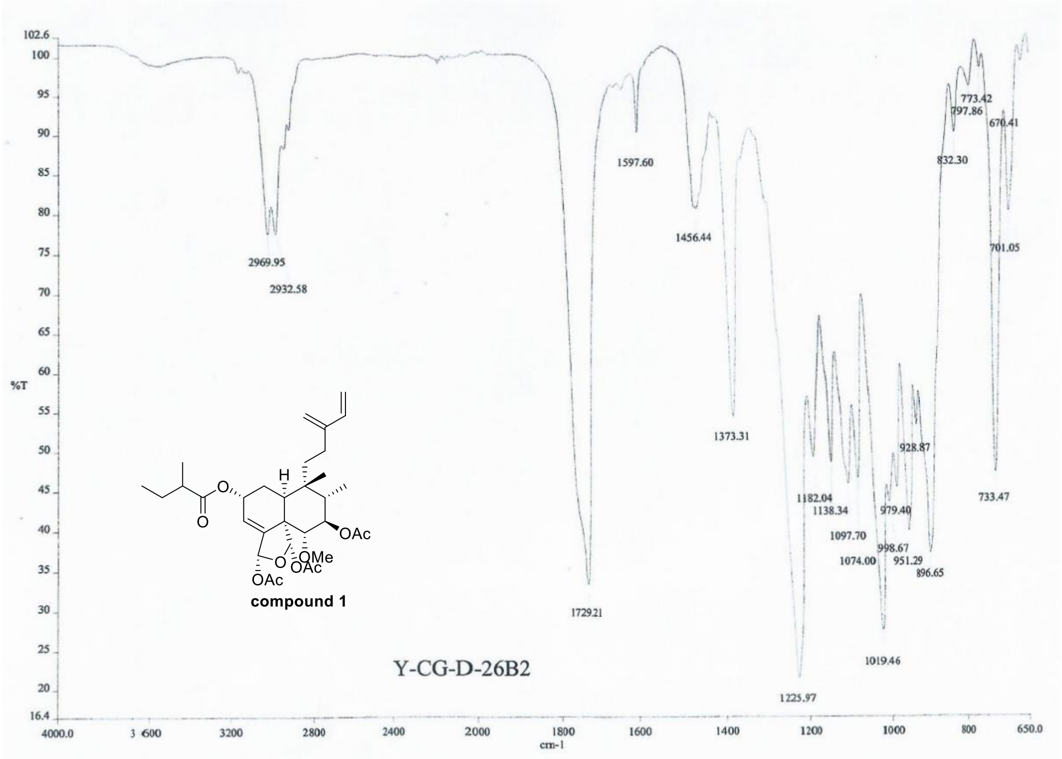

Figure S9. IR spectrum of compound **1**

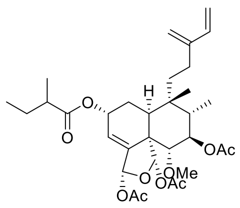

compound 1

Mass Spectrum SmartFormula Report

Analysis Info

Analysis Name	D:\Data\CRI\QCR10913 Vilailak Y-CG-D26B2 E+.d	Acquisition Date	1/21/2022 12:09:52 PM
Method	Nitirat esi pos low may2021-2.m	Operator	BDAL@DE
Sample Name	ESIpos	Instrument	compact 8255754.20094
Comment			

Acquisition Parameter

Source Type	ESI	Ion Polarity	Positive	Set Nebulizer	0.6 Bar
Focus	Not active	Set Capillary	3000 V	Set Dry Heater	180 °C
Scan Begin	150 m/z	Set End Plate Offset	-500 V	Set Dry Gas	5.8 l/min
Scan End	750 m/z	Set Charging Voltage	2000 V	Set Divert Valve	Source
		Set Corona	0 nA	Set APCI Heater	0 °C

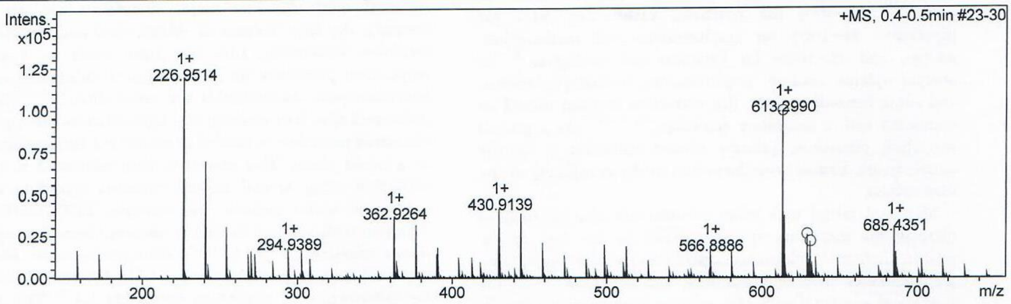

Meas. m/z	#	Ion Formula	m/z	err [ppm]	Mean err [ppm]	rdb	N-Rule	e⁻ Conf	mSigma	Std I	Std Mean m/z	Std I VarNorm	Std m/z Diff	Std Comb Dev
613.299045	1	C32H46NaO10	613.298318	-1.2	0.9	9.5	ok	even	2.3	3.7	n.a.	n.a.	n.a.	n.a.
	2	C29H45N2O12	613.296701	-3.8	-2.0	8.5	ok	even	11.7	19.0	n.a.	n.a.	n.a.	n.a.
	3	C34H45O10	613.300724	2.7	4.8	12.5	ok	even	12.2	17.5	n.a.	n.a.	n.a.	n.a.
629.273724	1	C35H42NaO9	629.272104	-2.6	1319.7	14.5	ok	even	47.6	89.0	n.a.	n.a.	n.a.	n.a.
	2	C37H41O9	629.274509	1.2	1319.4	17.5	ok	even	55.1	101.9	n.a.	n.a.	n.a.	n.a.
	3	C32H46KO10	629.272256	-2.3	2047.2	9.5	ok	even	71.3	142.0	n.a.	n.a.	n.a.	n.a.
	4	C36H43KN2NaO4	629.275210	2.4	2032.5	15.5	ok	even	75.9	153.3	n.a.	n.a.	n.a.	n.a.
631.306603	1	C32H48NaO11	631.308883	3.6	775.1	8.5	ok	even	8.5	13.6	n.a.	n.a.	n.a.	n.a.
	2	C29H47N2O13	631.307266	1.1	777.3	7.5	ok	even	12.4	16.9	n.a.	n.a.	n.a.	n.a.
	3	C40H45KN2NaO	631.306116	-0.8	1510.2	18.5	ok	even	60.4	111.6	n.a.	n.a.	n.a.	n.a.

Figure S10. ESITOFMS spectrum of compound **1**

Compound **2**

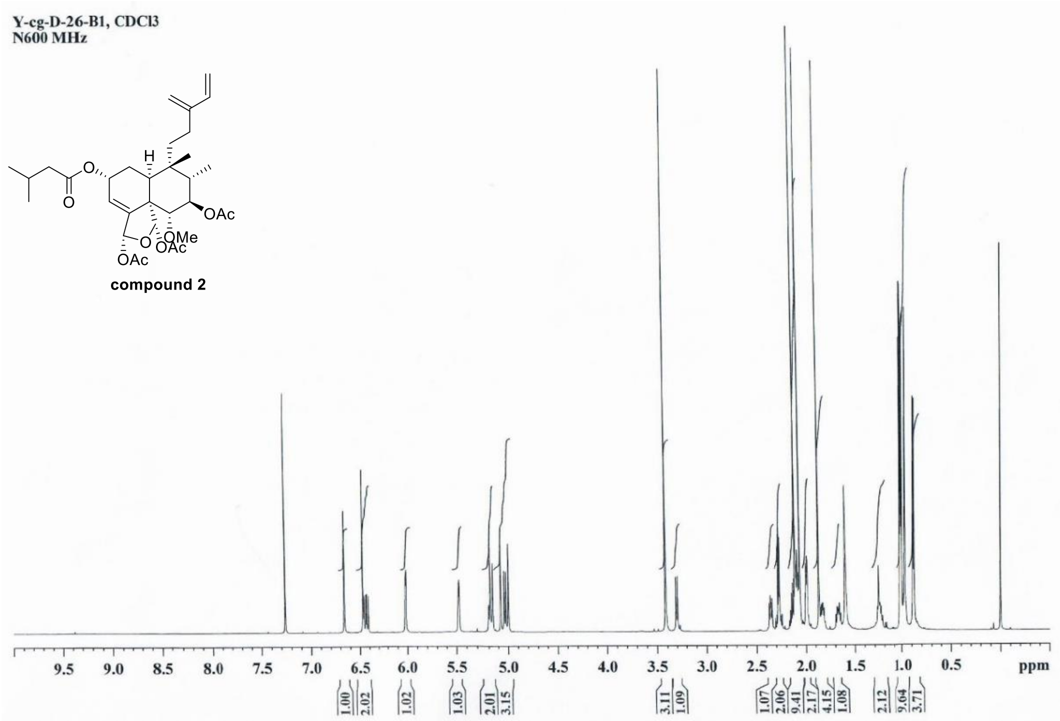

Figure S11. ^{1}H-NMR spectrum (600 MHz, $CDCl_3$) of compound **2**

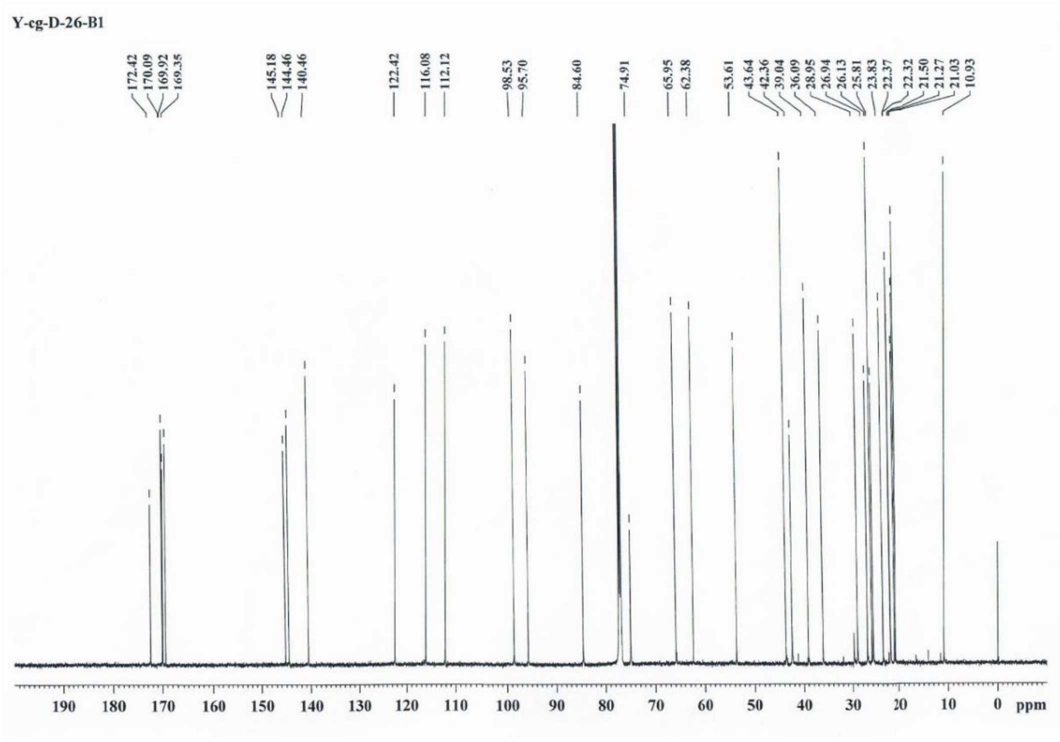

Figure S12. ^{13}C-NMR spectrum (150 MHz, $CDCl_3$) of compound **2**

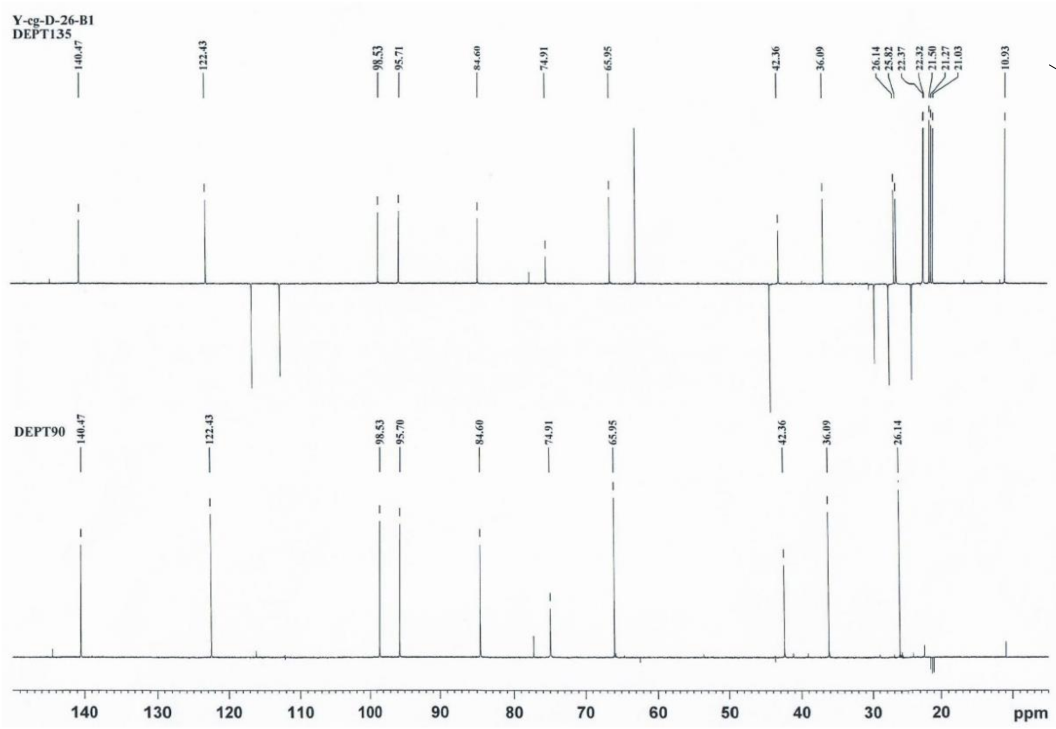

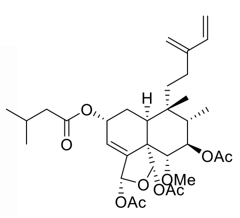

compound 2

Figure S13. DEPT spectrum (600 MHz, $CDCl_3$) of compound **2**

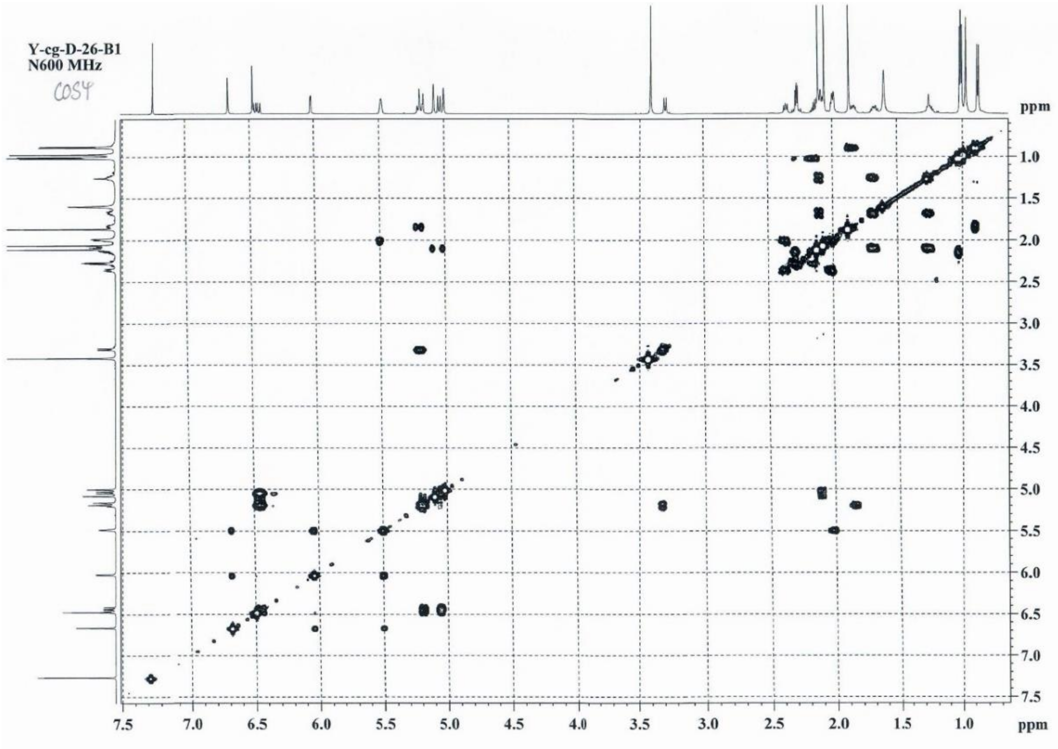

Figure S14. ^{1}H-^{1}H COSY spectrum (600 MHz, $CDCl_3$) of compound **2**

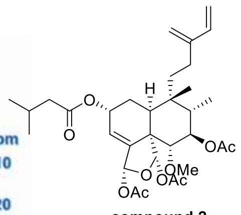

compound 2

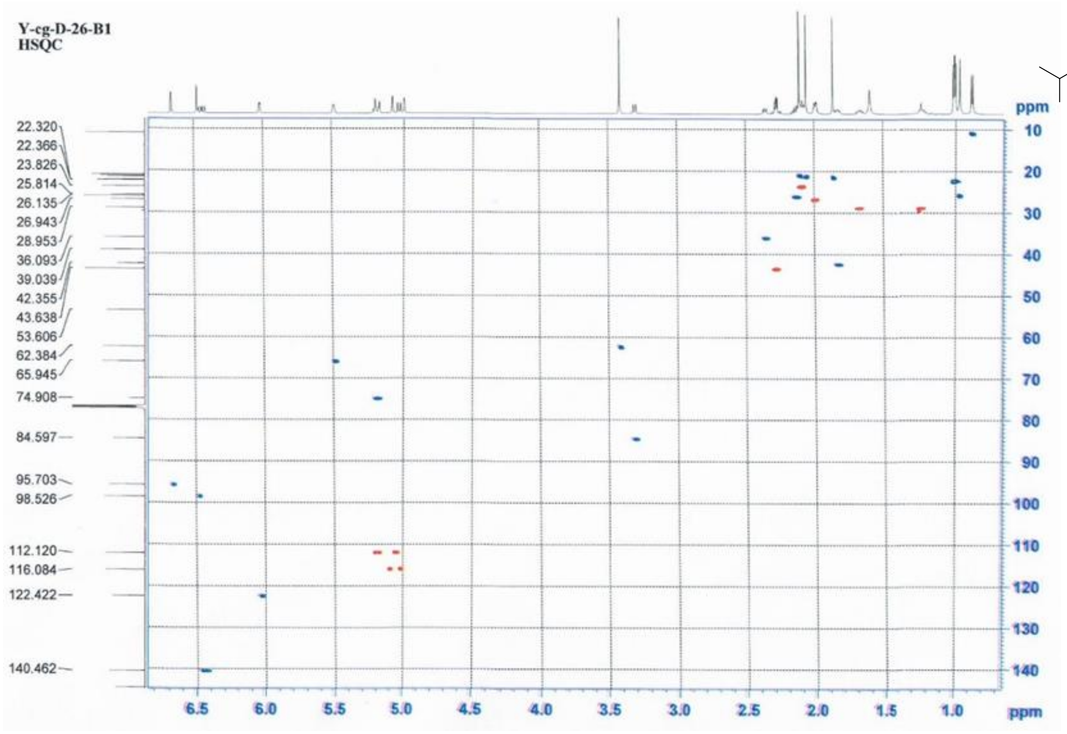

Figure S15. HSQC spectrum (600 MHz, $CDCl_3$) of compound **2**

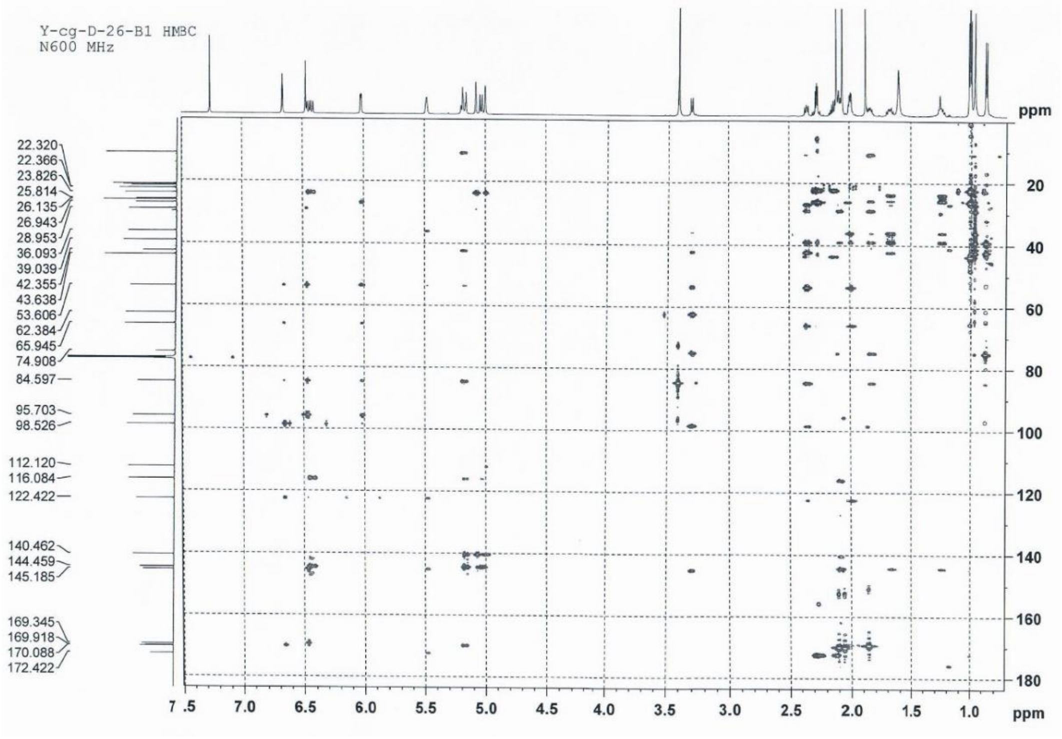

Figure S16. HMBC spectrum (600 MHz, $CDCl_3$) of compound **2**

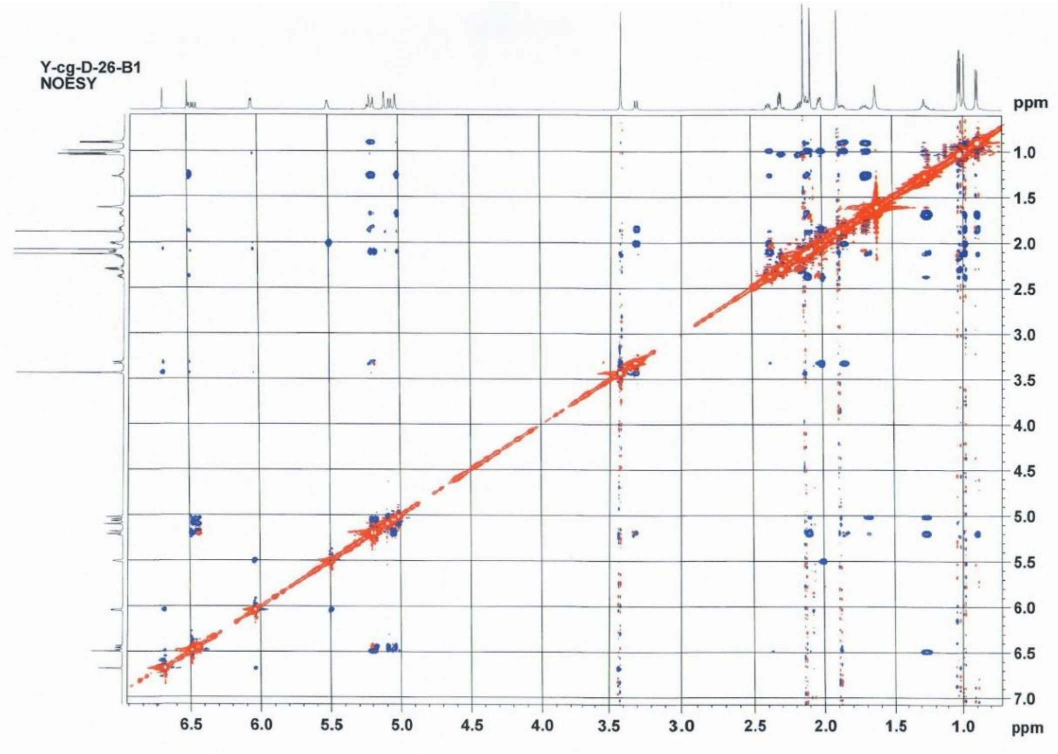

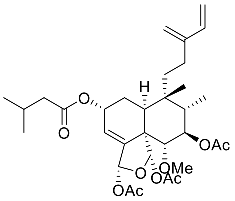

compound 2

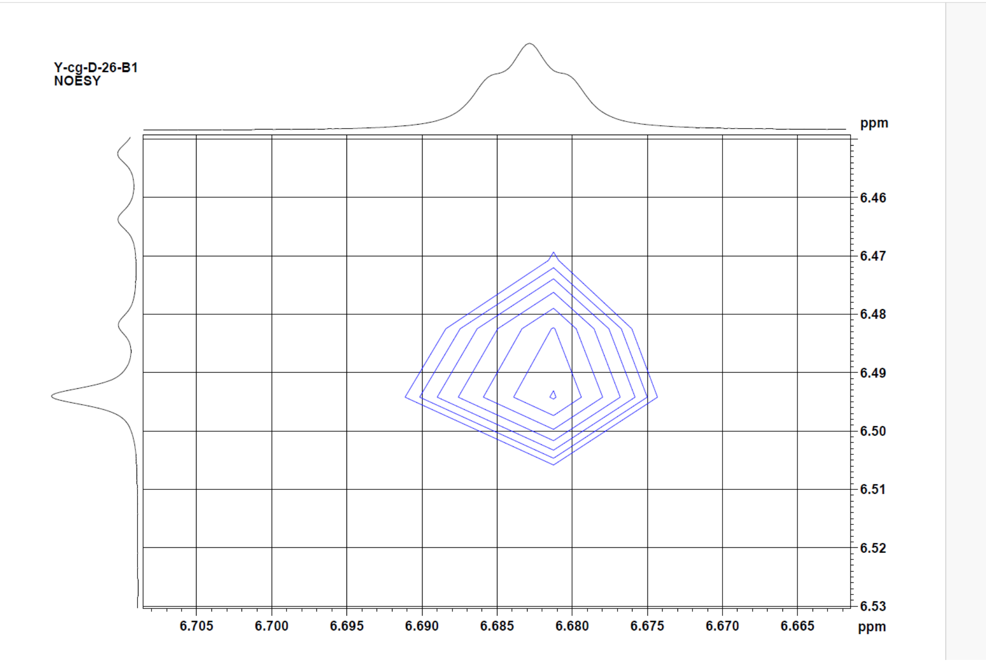

Figure S17. NOESY spectrum (600 MHz, $CDCl_3$) of compound **2**

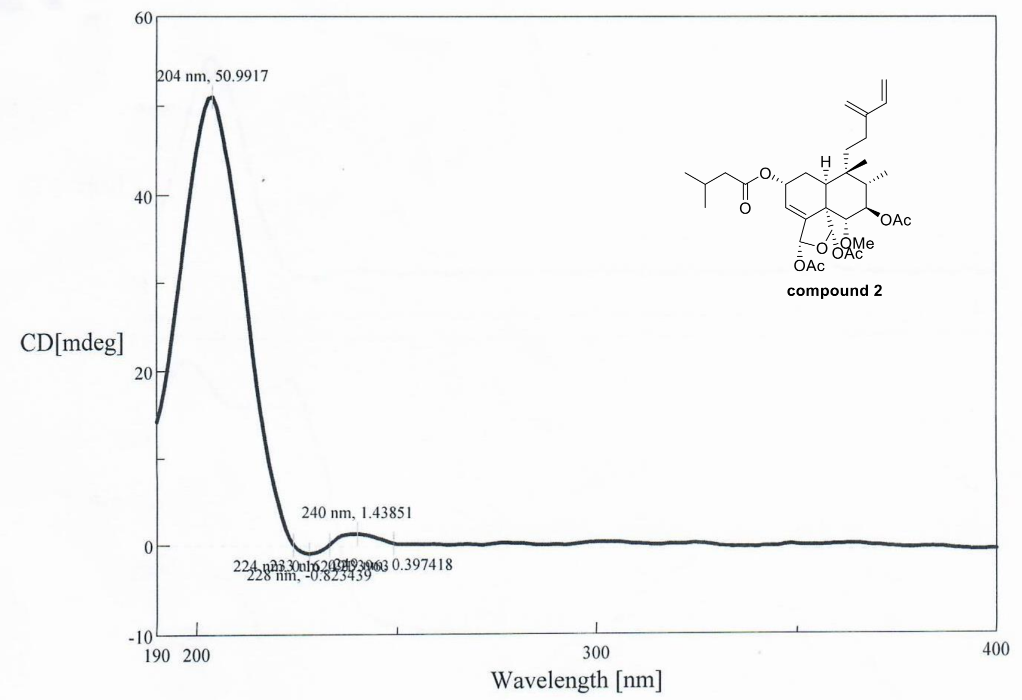

Date	8/4/2022 4:22PM
File name	Y-cg-D-26-B1_2
Model	J-815
Serial No.	A024561168
Band width	1 nm
Response	1 sec
Sensitivity	Standard
Measurement range	400 - 190 nm
Data pitch	1nm
Scanning speed	200 nm/min
Accumulation	3
Cell Length	1 cm
Solvent	ACN
Temperature	24.9 C
Sample name	Y-CG-D-26-B1
Operator	Vilailak
Comment	1.9 mg/2ml--200um/3800um

Figure S18. CD spectrum of compound **2**

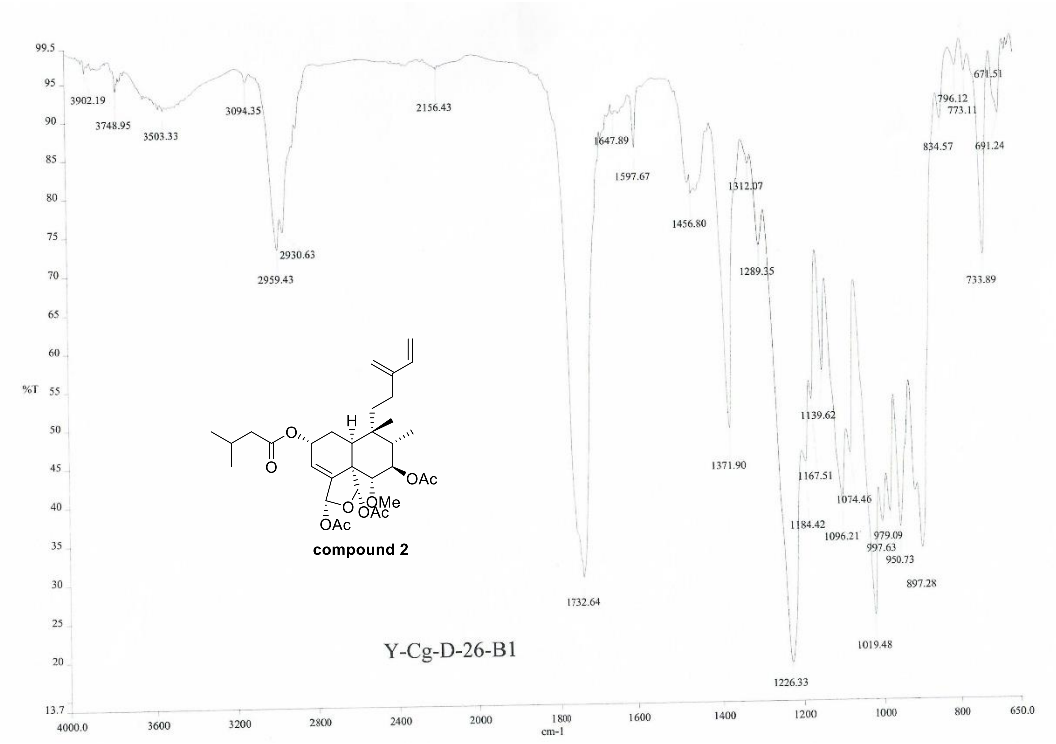

Figure S19. IR spectrum of compound **2**

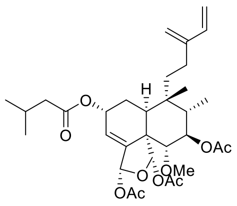

compound 2

Mass Spectrum SmartFormula Report

Analysis Info

Analysis Name	D:\Data\CRI\QCR10912 Vilailak Y-CG-D26B1 E+.d	Acquisition Date	1/21/2022 12:08:27 PM
Method	Nitirat esi pos low may2021-2.m	Operator	BDAL@DE
Sample Name	ESIpos	Instrument	compact 8255754.20094
Comment			

Acquisition Parameter

Source Type	ESI	Ion Polarity	Positive	Set Nebulizer	0.6 Bar
Focus	Not active	Set Capillary	3000 V	Set Dry Heater	180 °C
Scan Begin	150 m/z	Set End Plate Offset	-500 V	Set Dry Gas	5.8 l/min
Scan End	700 m/z	Set Charging Voltage	2000 V	Set Divert Valve	Source
		Set Corona	0 nA	Set APCI Heater	0 °C

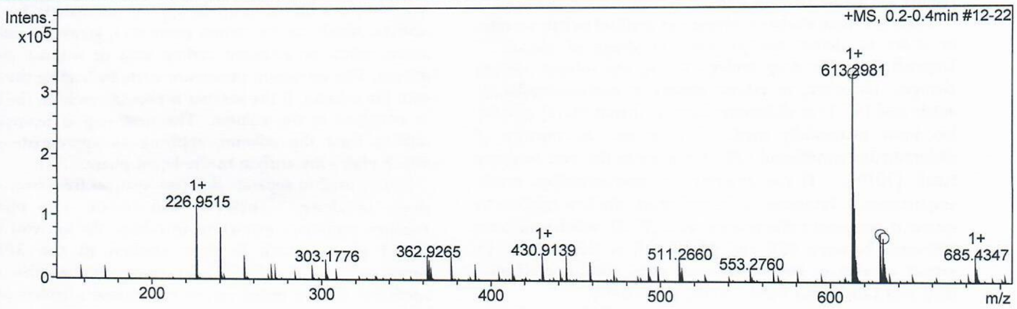

Meas. m/z	#	Ion Formula	m/z	err [ppm]	Mean err [ppm]	rdb	N-Rule	e⁻ Conf	mSigma	Std I	Std Mean m/z	Std I VarNorm	Std m/z Diff	Std Comb Dev
613.298137	1	C32H46NaO10	613.298318	0.3	3.2	9.5	ok	even	4.0	7.3	n.a.	n.a.	n.a.	n.a.
	2	C29H45N2O12	613.296701	-2.3	0.3	8.5	ok	even	13.1	22.2	n.a.	n.a.	n.a.	n.a.
629.273286	1	C35H42NaO9	629.272104	-1.9	1320.1	14.5	ok	even	50.0	95.0	n.a.	n.a.	n.a.	n.a.
	2	C37H41O9	629.274509	1.9	1319.8	17.5	ok	even	58.7	109.0	n.a.	n.a.	n.a.	n.a.
	3	C32H46KO10	629.272256	-1.6	2047.5	9.5	ok	even	71.3	145.6	n.a.	n.a.	n.a.	n.a.
	4	C36H43KN2NaO4	629.275210	3.1	2032.8	15.5	ok	even	77.8	159.1	n.a.	n.a.	n.a.	n.a.
631.305903	1	C32H48NaO11	631.308883	4.7	776.1	8.5	ok	even	8.7	15.9	n.a.	n.a.	n.a.	n.a.
	2	C29H47N2O13	631.307266	2.2	778.3	7.5	ok	even	10.7	14.6	n.a.	n.a.	n.a.	n.a.
	3	C36H43N2O8	631.301393	-7.1	757.0	16.5	ok	even	30.9	48.7	n.a.	n.a.	n.a.	n.a.
	4	C39H44NaO6	631.303010	-4.6	754.5	17.5	ok	even	43.2	65.3	n.a.	n.a.	n.a.	n.a.

Figure S20. ESITOFMS spectrum of compound **2**

Compound **3**

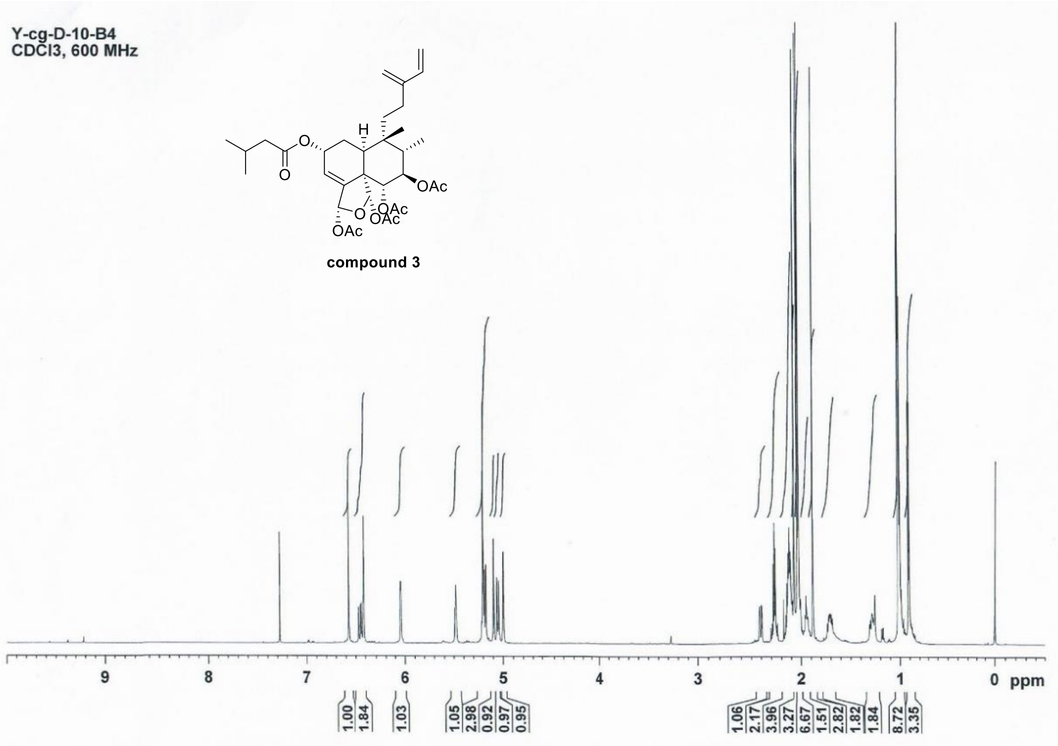

Figure S21. ^{1}H-NMR spectrum (600 MHz, $CDCl_3$) of compound **3**

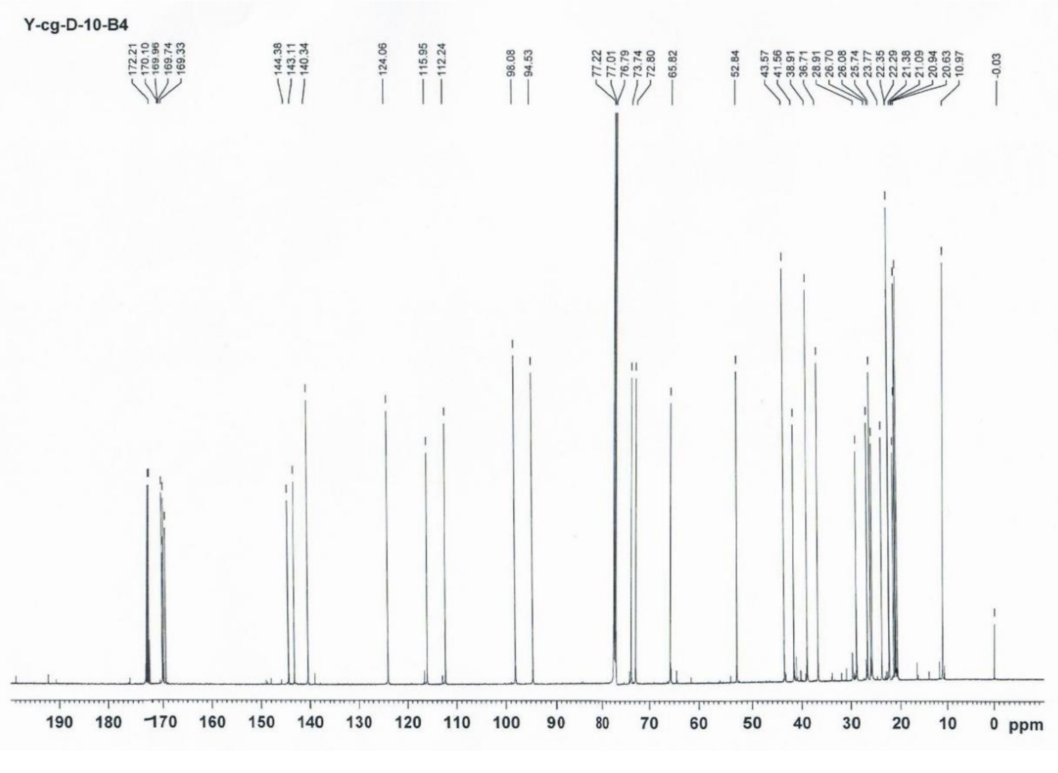

Figure S22. ^{13}C-NMR spectrum (150 MHz, $CDCl_3$) of compound **3**

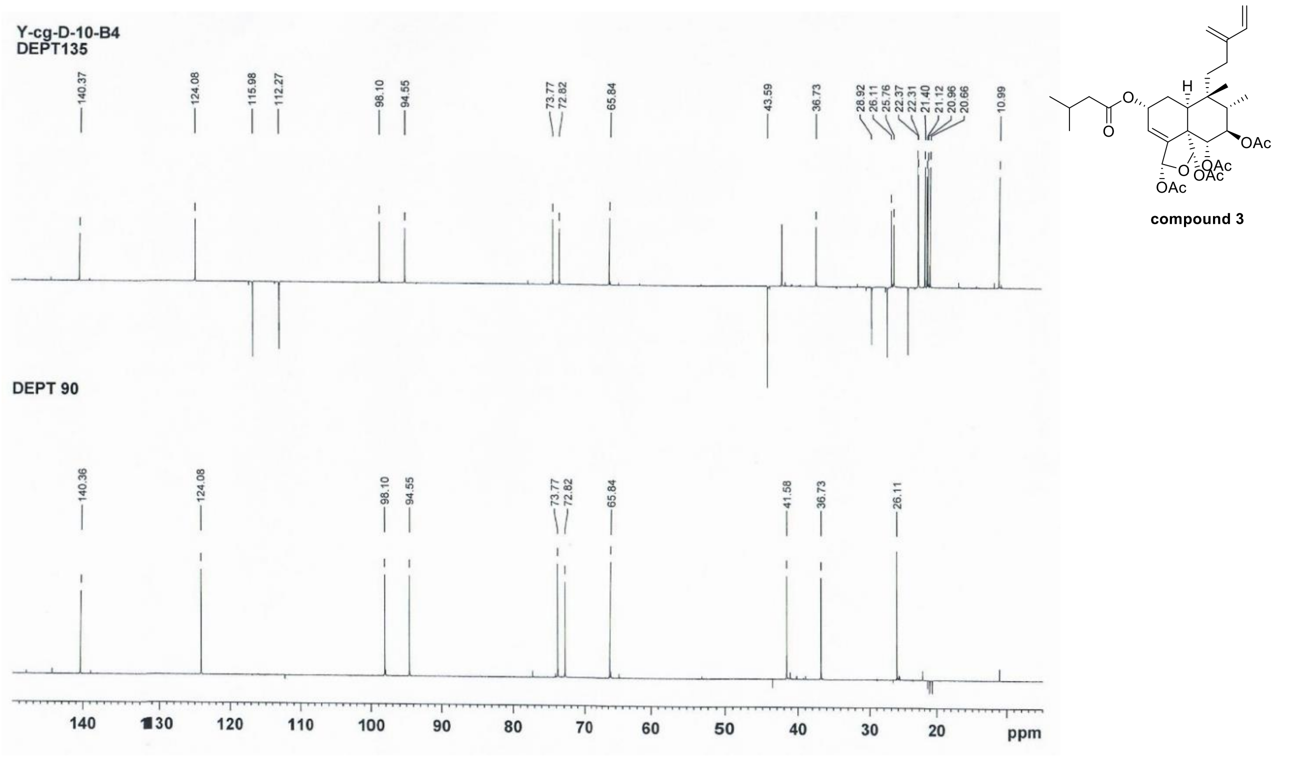

Figure S23. DEPT spectrum (600 MHz, $CDCl_3$) of compound **3**

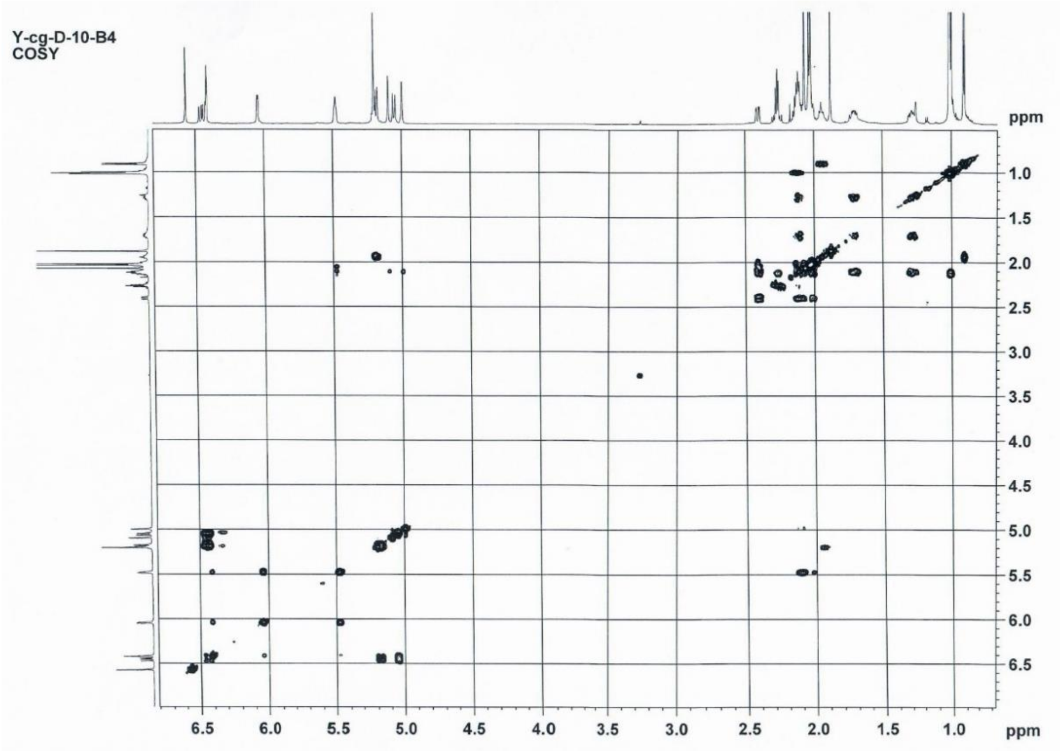

Figure S24. ^1H-^1H COSY spectrum (600 MHz, $CDCl_3$) of compound **3**

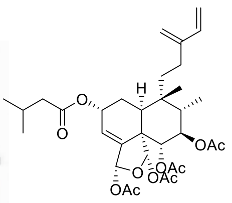

compound 3

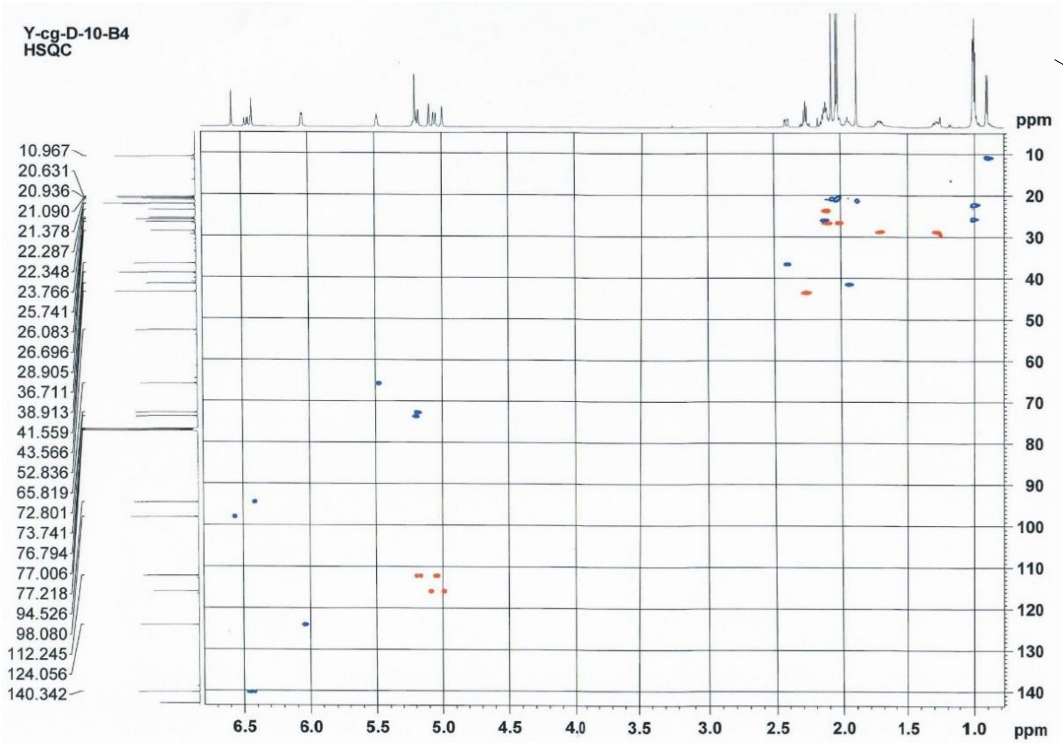

Figure S25. HSQC spectrum (600 MHz, $CDCl_3$) of compound **3**

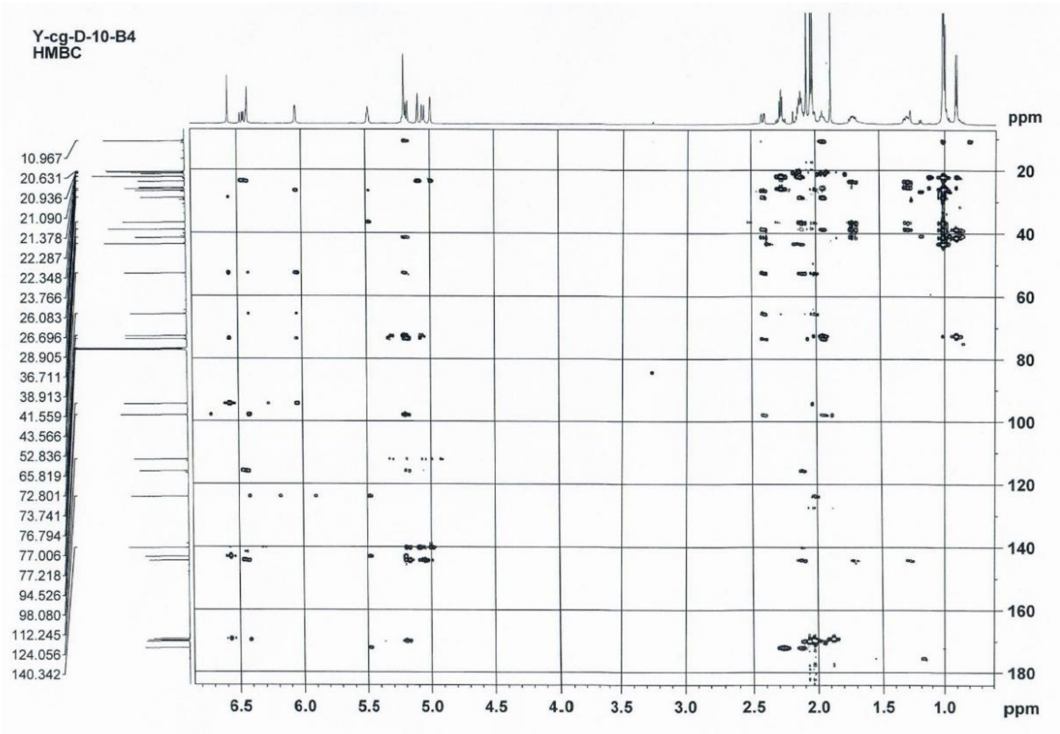

Figure S26. HMBC spectrum (600 MHz, $CDCl_3$) of compound **3**

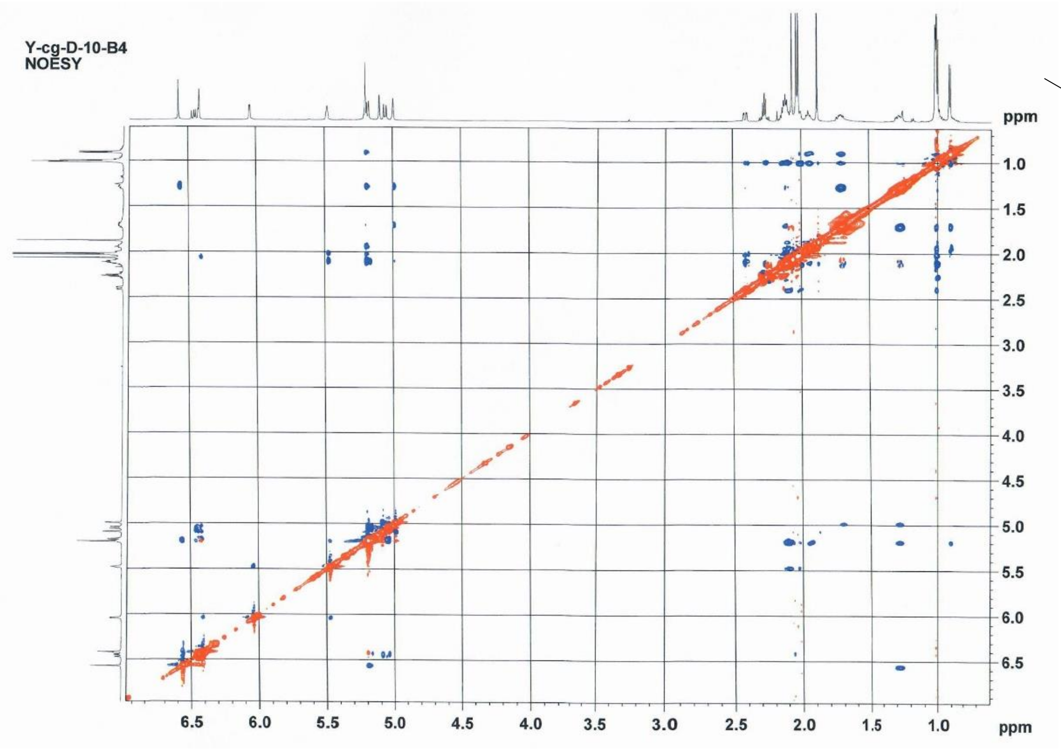

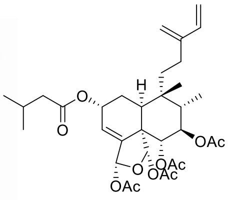

compound 3

Figure S27. NOESY spectrum (600 MHz, $CDCl_3$) of compound **3**

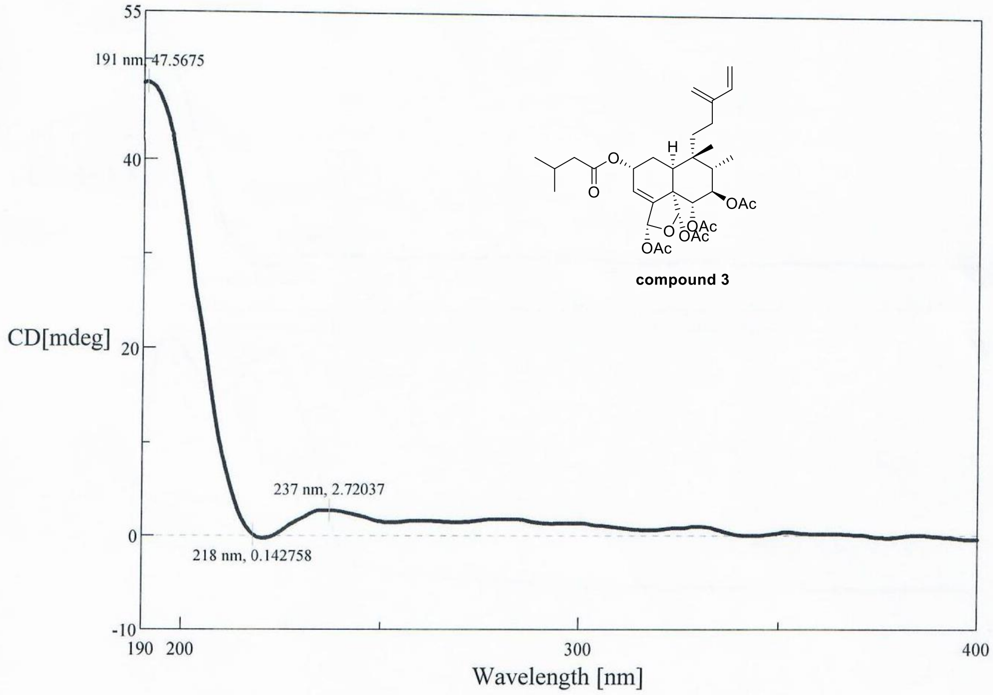

Date	8/5/2022 3:33PM
File name	Y-cg-D-10-B4 original dilution_2
Model	J-815
Serial No.	A024561168
Band width	1 nm
Response	1 sec
Sensitivity	Standard
Measurement range	400 - 190 nm
Data pitch	1nm
Scanning speed	200 nm/min
Accumulation	2
Cell Length	1 cm
Solvent	ACN
Temperature	25.1 C
Sample name	Y-CG-D-10-B4
Operator	Vilailak
Comment	1.9 mg/2ml--150ul/3850ul

Figure S28. CD spectrum of compound **3**

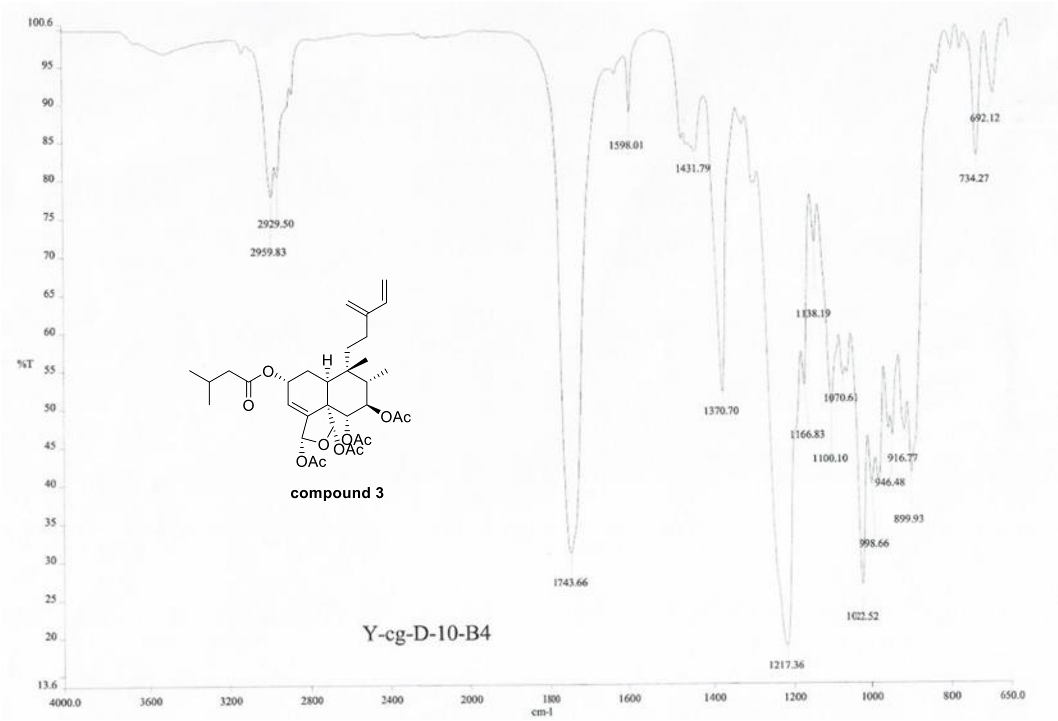

Figure S29. IR spectrum of compound **3**

Mass Spectrum SmartFormula Report

Analysis Info

Analysis Name	D:\Data\CRI\QCR09467 Vialilak Y-cgD-10B4 E+.d	Acquisition Date	7/12/2021 12:59:03 PM
Method	Nitirat esi pos low may2021.m	Operator	BDAL@DE
Sample Name	ESIpos	Instrument	compact 8255754.20094
Comment			

Acquisition Parameter

Source Type	ESI	Ion Polarity	Positive	Set Nebulizer	1.5 Bar
Focus	Not active	Set Capillary	3000 V	Set Dry Heater	36 °C
Scan Begin	100 m/z	Set End Plate Offset	-200 V	Set Dry Gas	4.0 l/min
Scan End	1000 m/z	Set Charging Voltage	2000 V	Set Divert Valve	Source
		Set Corona	0 nA	Set APCI Heater	0 °C

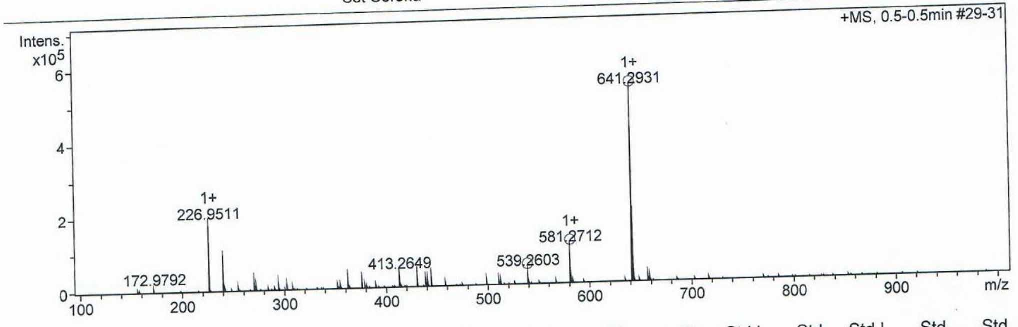

Meas. m/z	#	Ion Formula	m/z	err [ppm]	Mean err [ppm]	rdb	N-Rule	e⁻ Conf	mSigma	Std I	Std Mean m/z	Std I VarNorm	Std m/z Diff	Std Comb Dev
539.260340	1	C29H40NaO8	539.261539	2.2	912.8	9.5	ok	even	9.5	13.9	n.a.	n.a.	n.a.	n.a.
	2	C31H39O8	539.263945	6.7	912.1	12.5	ok	even	17.3	27.4	n.a.	n.a.	n.a.	n.a.
	3	C38H35O3	539.258071	-4.2	886.6	21.5	ok	even	53.6	80.5	n.a.	n.a.	n.a.	n.a.
581.271176	1	C31H42NaO9	581.272104	1.6	-0.6	10.5	ok	even	18.2	28.3	n.a.	n.a.	n.a.	n.a.
	2	C33H41O9	581.274509	5.7	3.6	13.5	ok	even	23.4	31.3	n.a.	n.a.	n.a.	n.a.
	3	C40H37O4	581.268636	-4.4	-6.2	22.5	ok	even	57.1	74.7	n.a.	n.a.	n.a.	n.a.
* 641.293072	1	C33H46NaO11	641.293233	0.3	1.8	10.5	ok	even	4.1	6.4	n.a.	n.a.	n.a.	n.a.
	2	C35H45O11	641.295639	4.0	5.5	13.5	ok	even	15.5	23.7	n.a.	n.a.	n.a.	n.a.
	3	C42H41O6	641.289765	-5.2	-3.4	22.5	ok	even	53.5	74.8	n.a.	n.a.	n.a.	n.a.

Figure S30. ESITOFMS spectrum of compound **3**

Compound **3** in C_6D_6

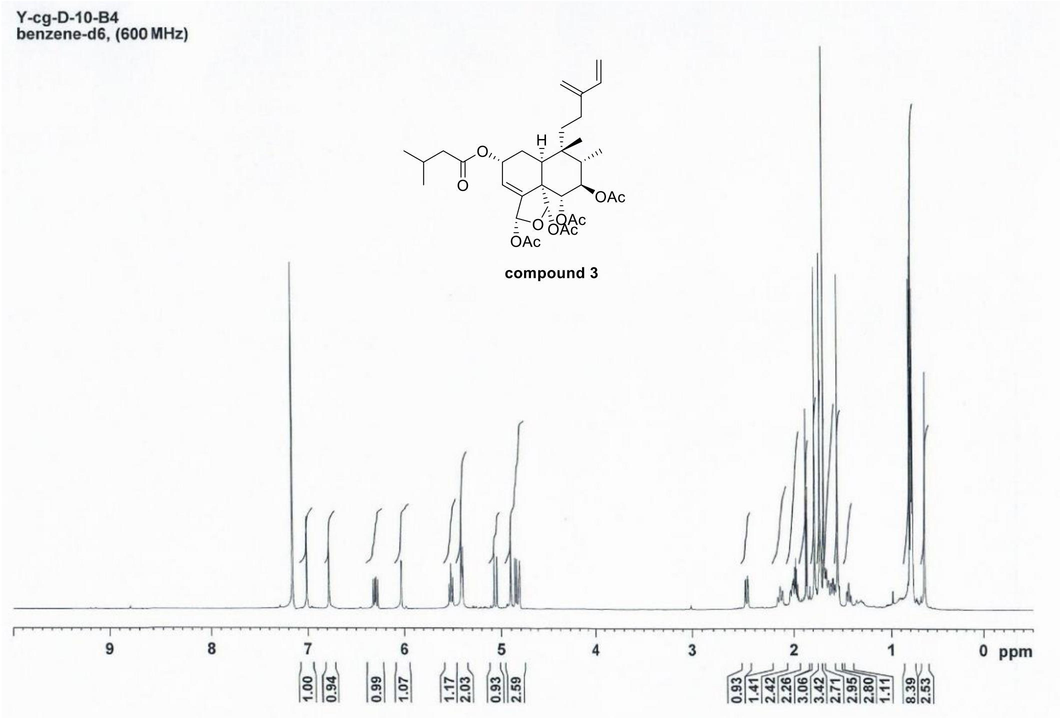

Figure S31. ^{1}H-NMR spectrum (600 MHz, C_6D_6) of compound **3**

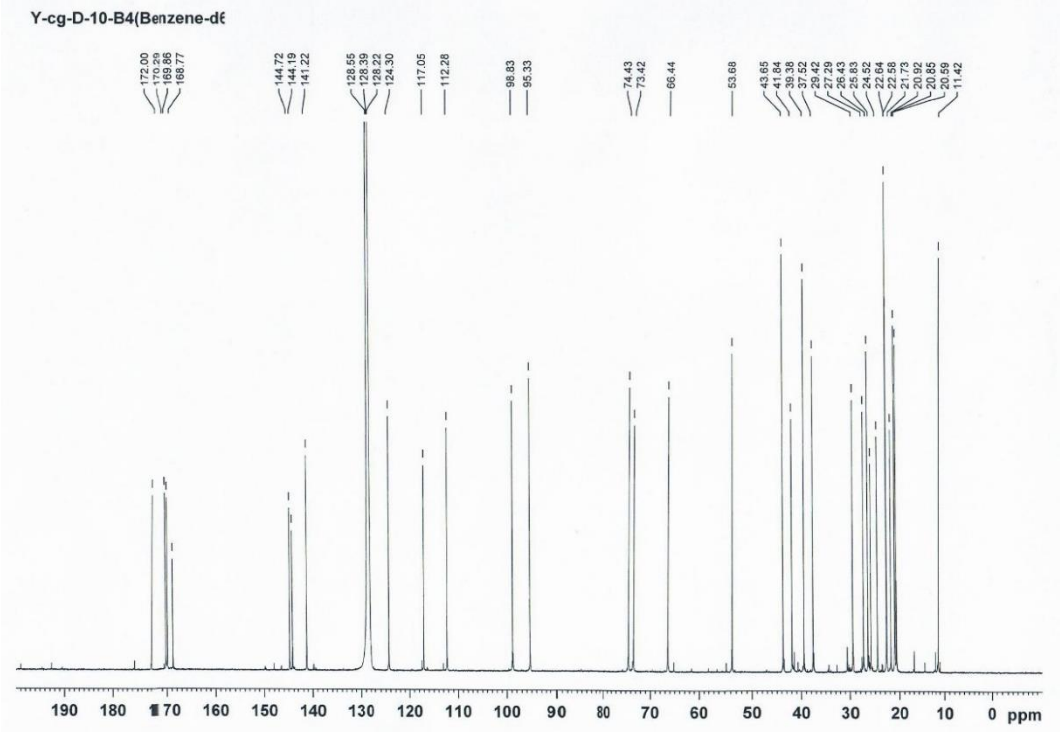

Figure S32. ^{13}C-NMR spectrum (150 MHz, C_6D_6) of compound **3**

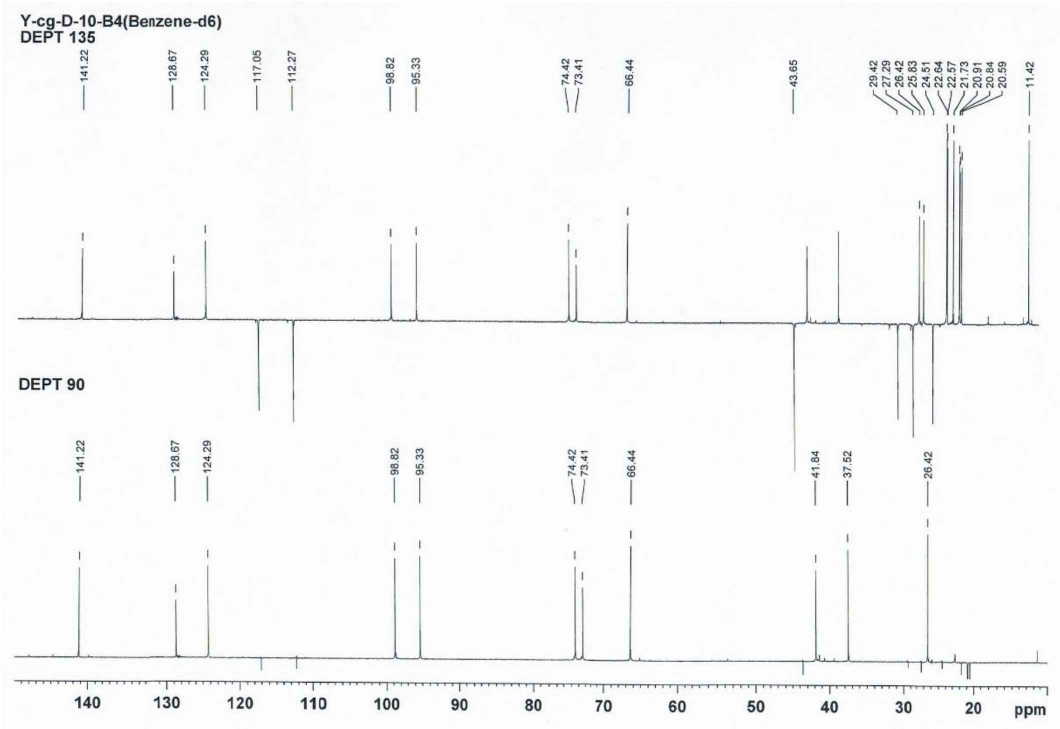

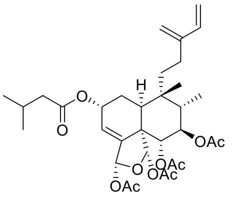

compound 3

Figure S33. DEPT spectrum (600 MHz, C_6D_6) of compound **3**

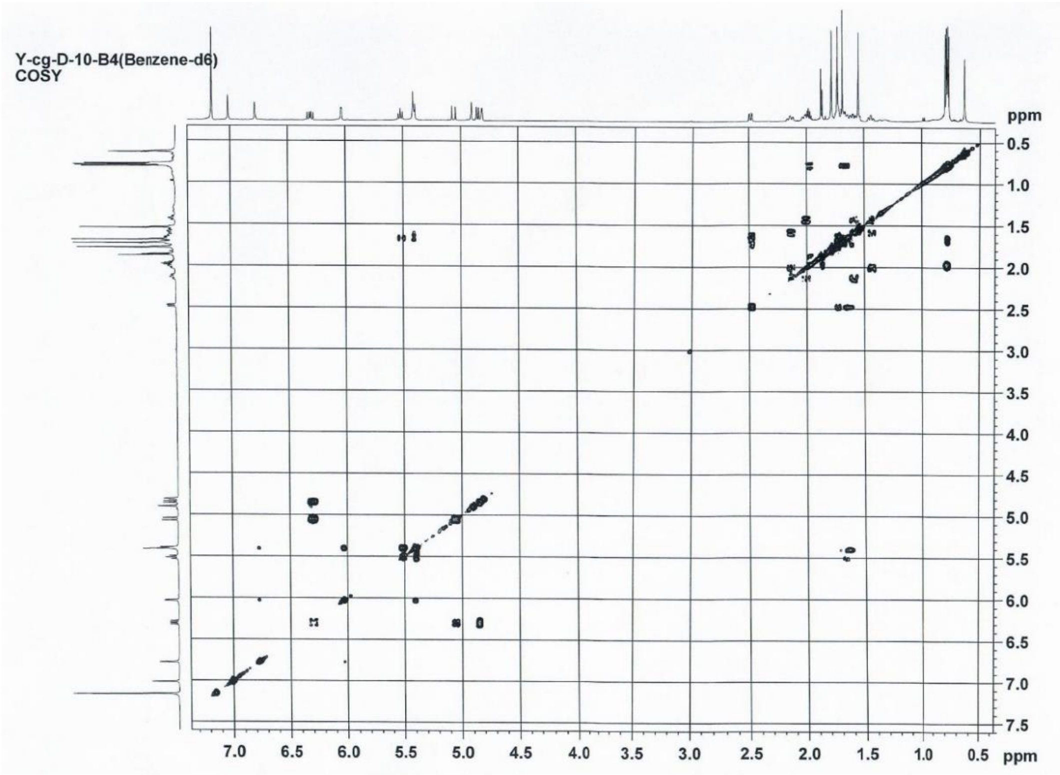

Figure S34. ^{1}H-^{1}H COSY spectrum (600 MHz, C_6D_6) of compound **3**

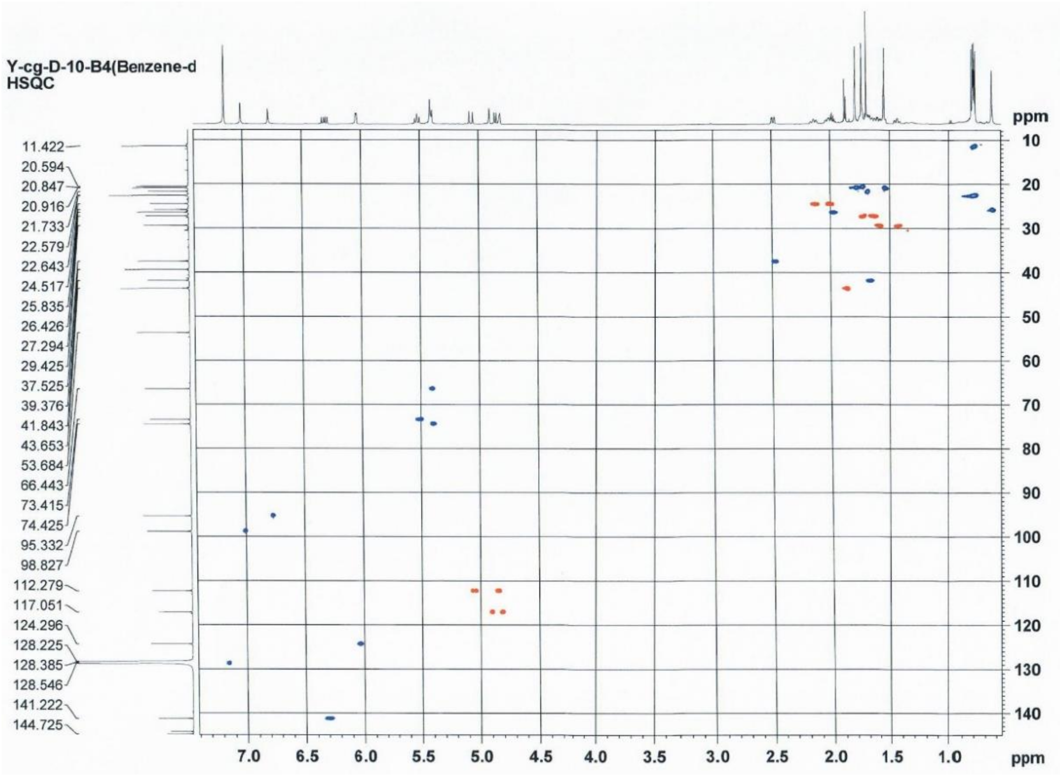

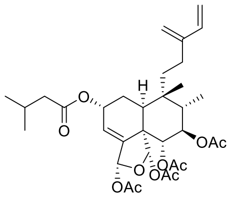

compound 3

Figure S35. HSQC spectrum (600 MHz, C_6D_6) of compound **3**

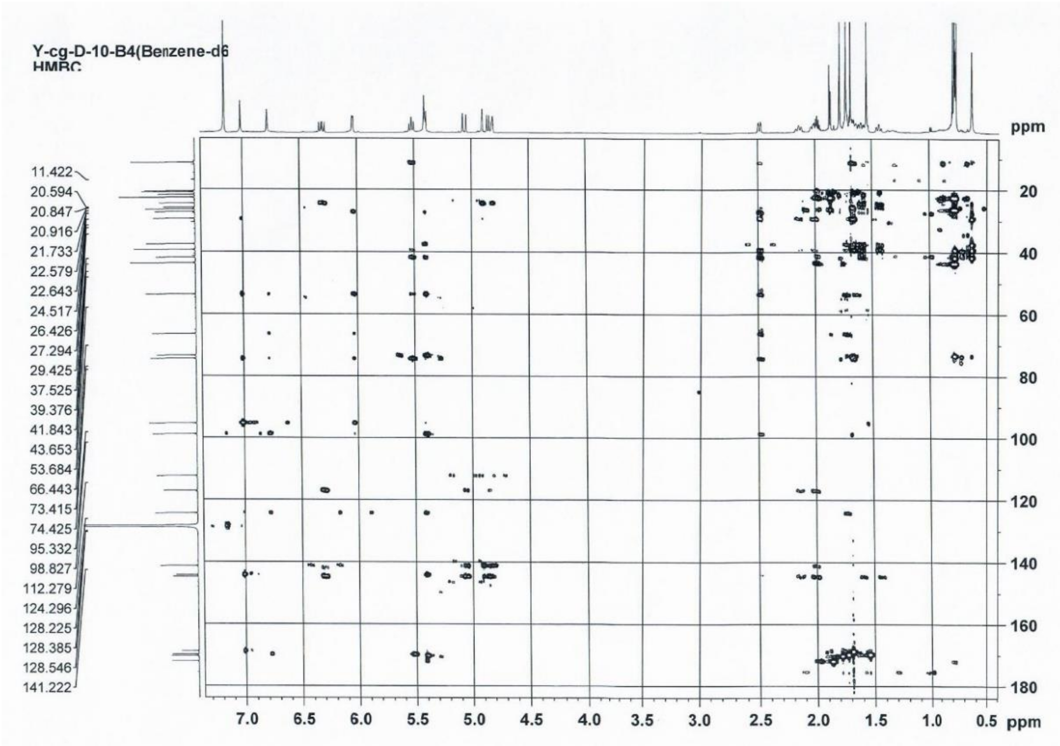

Figure S36. HMBC spectrum (600 MHz, C_6D_6) of compound **3**

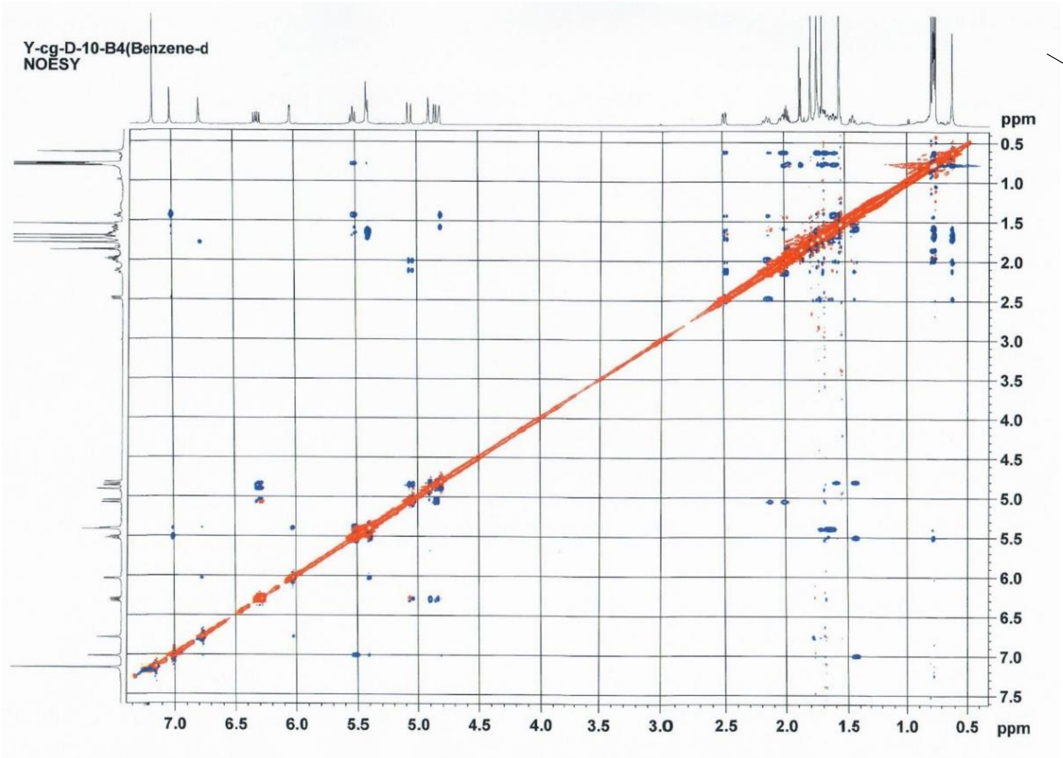

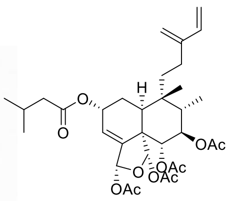

compound 3

Figure S37. NOESY spectrum (600 MHz, C_6D_6) of compound **3**

Compound **4**

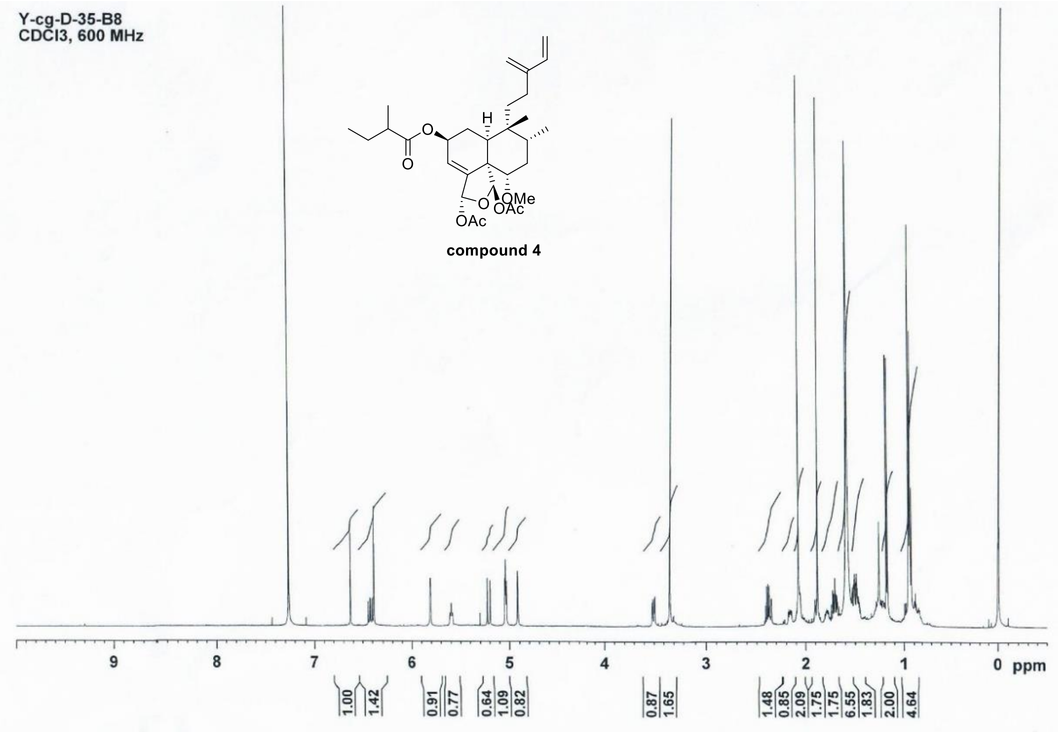

Figure S38. ^{1}H-NMR spectrum (600 MHz, $CDCl_3$) of compound **4**

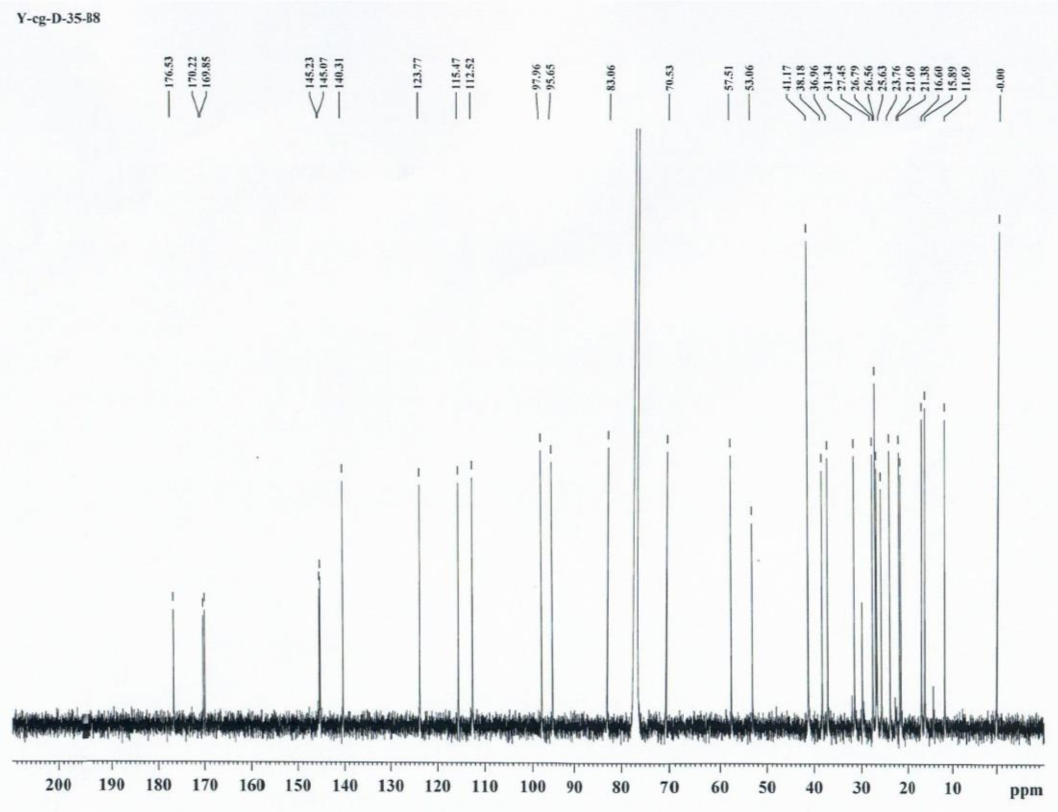

Figure S39. ^{13}C-NMR spectrum (150 MHz, $CDCl_3$) of compound **4**

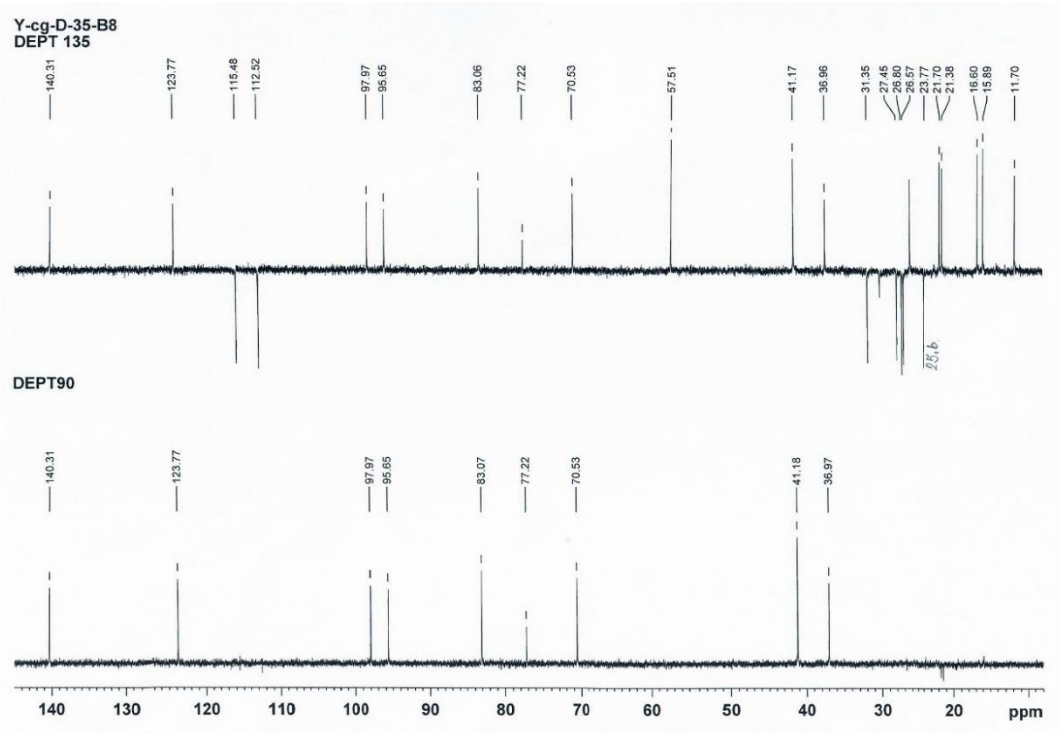

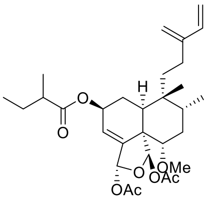

compound 4

Figure S40. DEPT spectrum (600 MHz, $CDCl_3$) of compound **4**

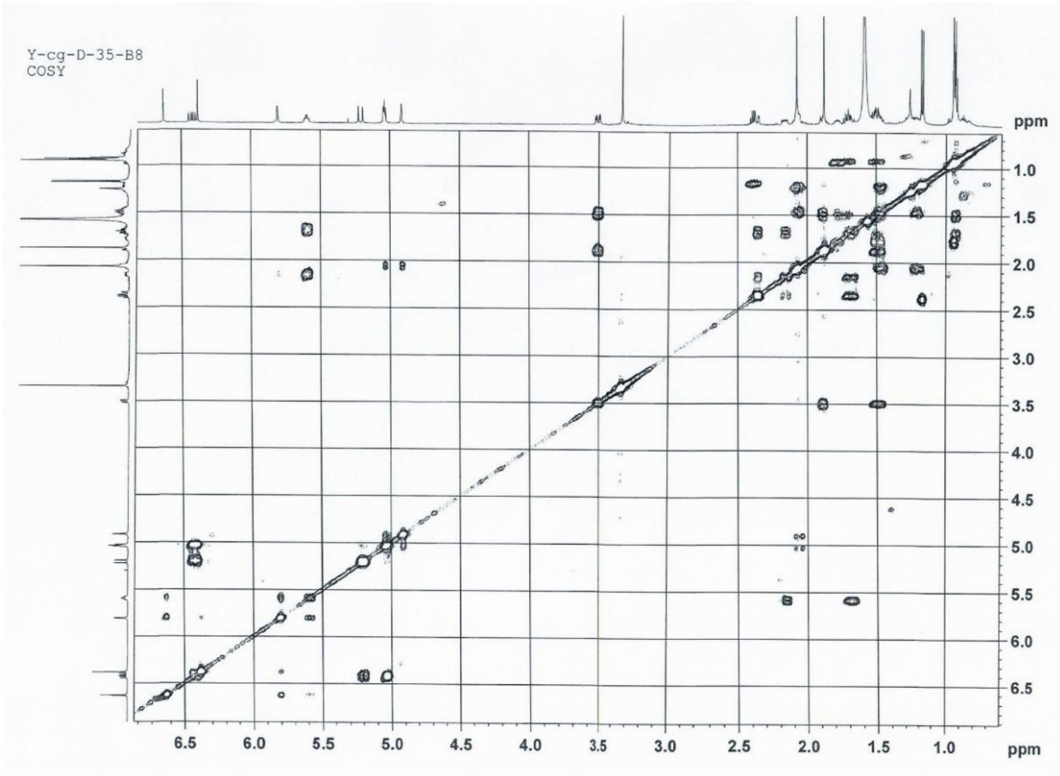

Figure S41. ^{1}H-^{1}H COSY spectrum (600 MHz, $CDCl_3$) of compound **4**

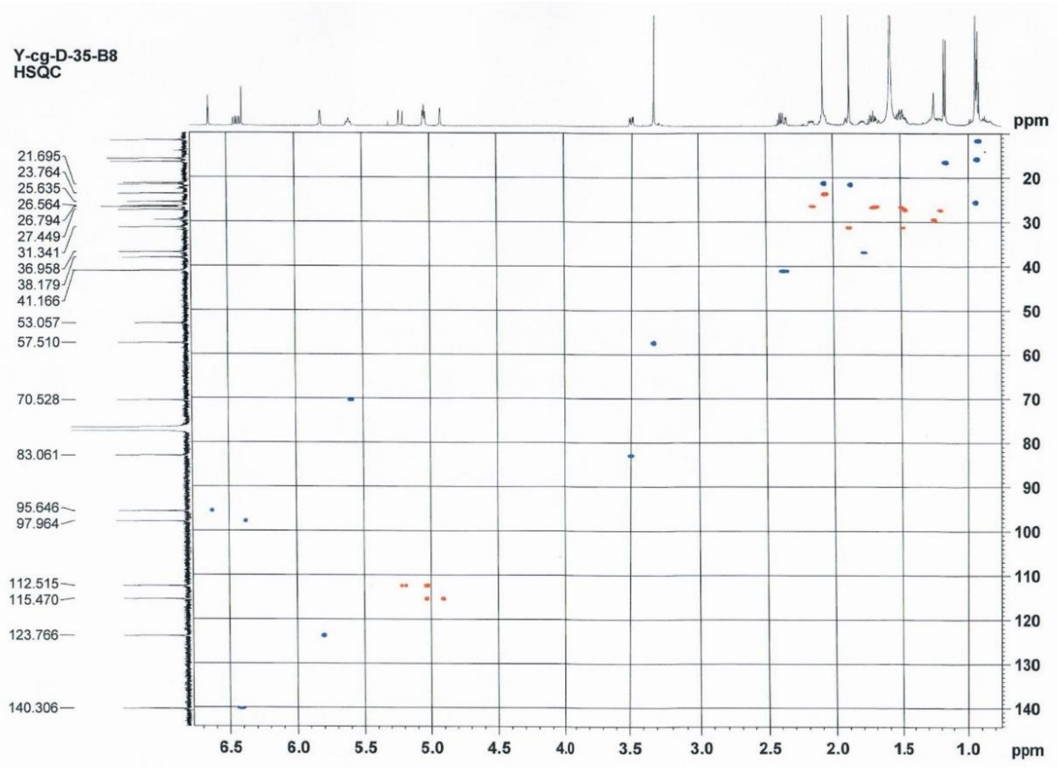

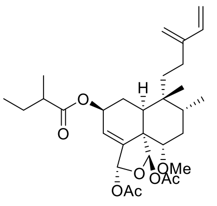

compound 4

Figure S42. HSQC spectrum (600 MHz, $CDCl_3$) of compound **4**

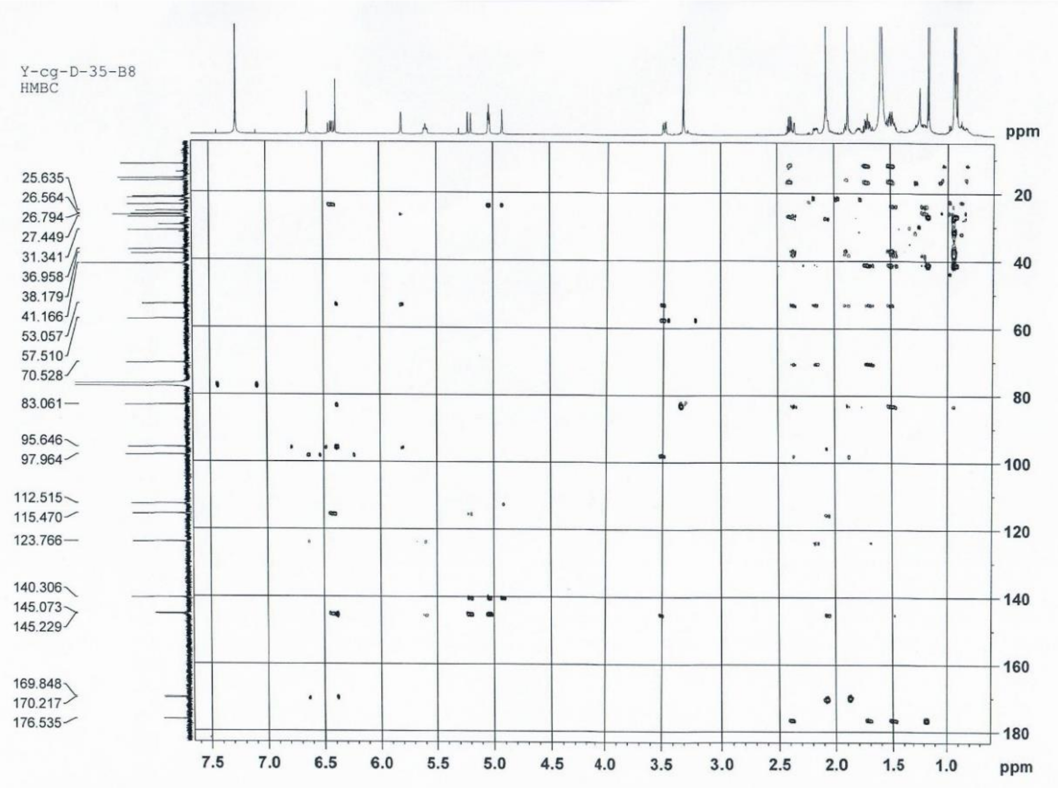

Figure S43. HMBC spectrum (600 MHz, $CDCl_3$) of compound **4**

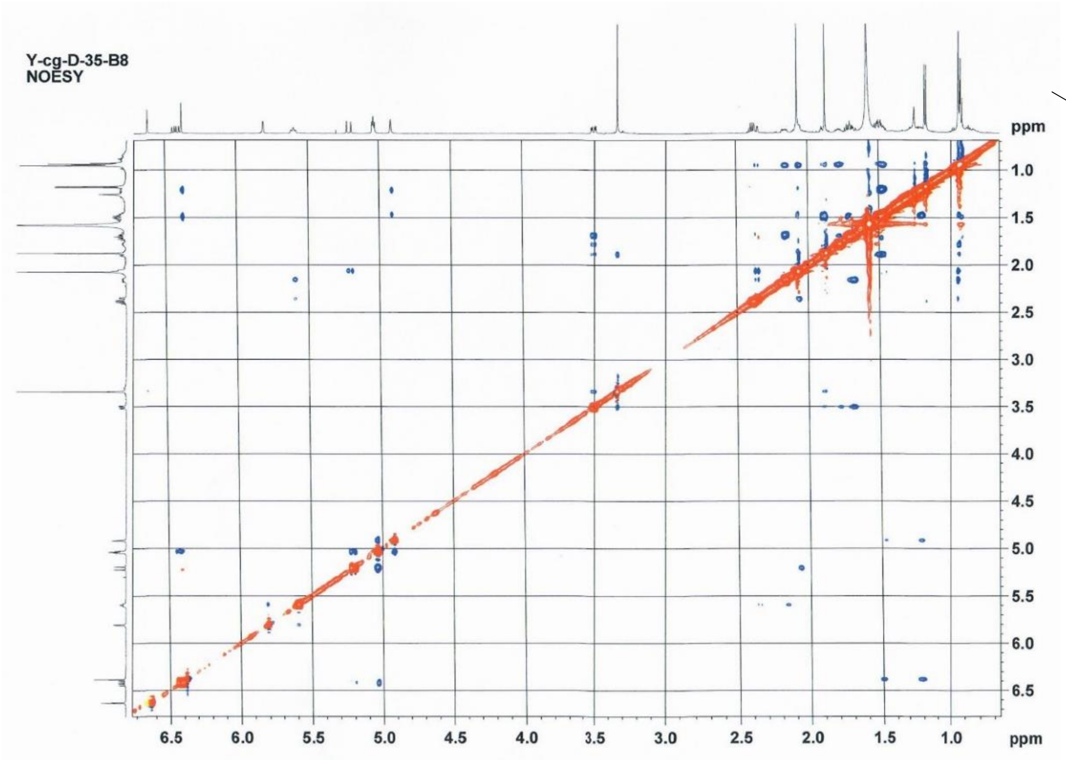

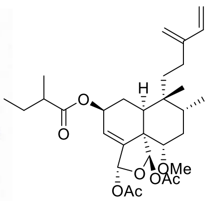

compound 4

Figure S44. NOESY spectrum (600 MHz, $CDCl_3$) of compound **4**

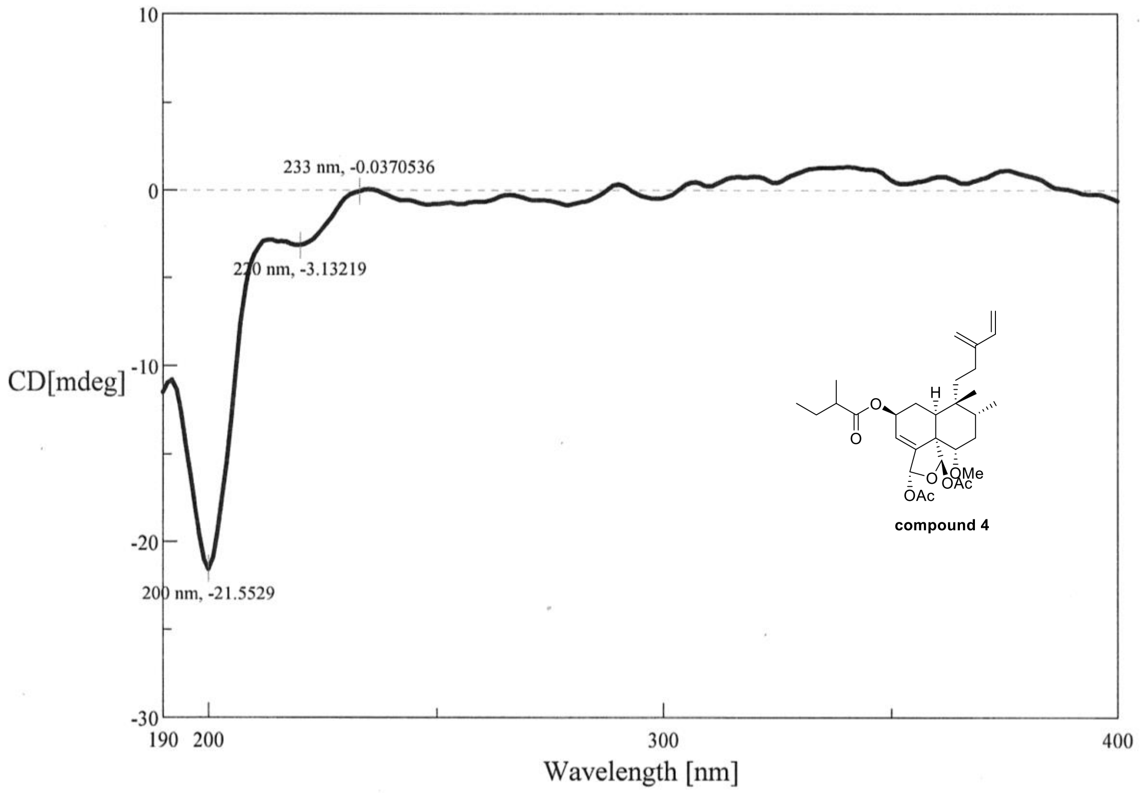

Date	5/8/2024 0:40PM
File name	Memory#20
Model	J-815
Serial No.	A024561168
Band width	1 nm
Response	1 sec
Sensitivity	Standard
Measurement range	400 - 190 nm
Data pitch	1nm
Scanning speed	200 nm/min
Accumulation	2
Cell Length	1 cm
Solvent	ACN
Temperature	25 C
Sample name	Y-cg-D-35-B8
Operator	vilailak
Comment	1.5 mg in 2 mL-- --400 um-1.6 ml

Figure S45. CD spectrum of compound **4**

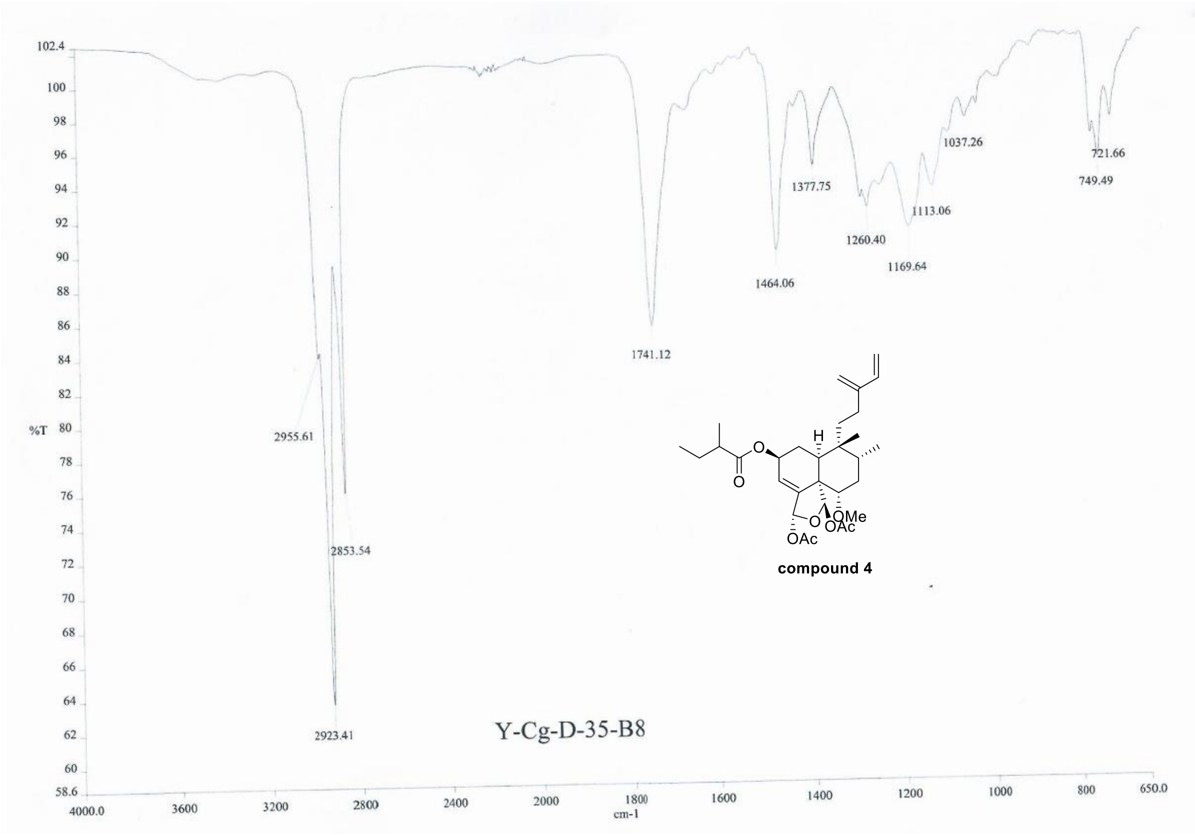

Figure S46. IR spectrum of compound **4**

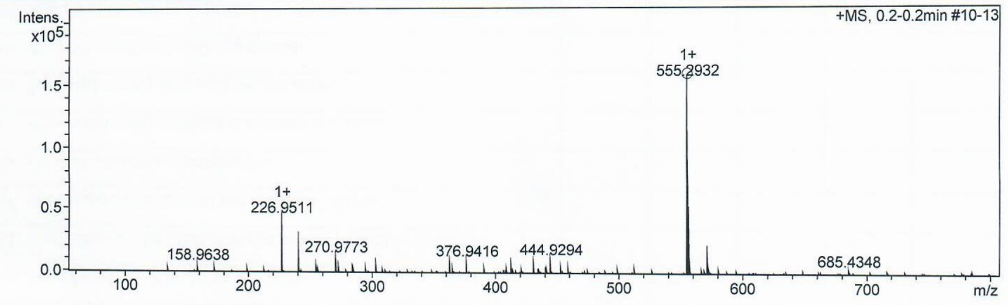

Mass Spectrum SmartFormula Report

Analysis Info

Acquisition Date 6/28/2022 11:15:14 AM

Analysis Name D:\Data\CRI\QCR11985 Vialailak Y-CGD-35BB E+.d
Method Nitirat esi pos low 2022-1.m
Sample Name ESIpos
Comment

Operator BDAL@DE
Instrument compact 8255754.20094

Acquisition Parameter

Source Type	ESI	Ion Polarity	Positive	Set Nebulizer	0.5 Bar
Focus	Not active	Set Capillary	3000 V	Set Dry Heater	155 °C
Scan Begin	60 m/z	Set End Plate Offset	-500 V	Set Dry Gas	5.5 l/min
Scan End	800 m/z	Set Charging Voltage	2000 V	Set Divert Valve	Source
		Set Corona	0 nA	Set APCI Heater	0 °C

Meas. m/z	#	Ion Formula	m/z	err [ppm]	Mean err [ppm]	rdb	N-Rule	e⁻ Conf	mSigma	Std I	Std Mean m/z	Std I VarNorm	Std m/z Diff	Std Comb Dev
555.293157	1	C30H44NaO8	555.292839	-0.6	1.2	8.5	ok	even	2.3	3.2	n.a.	n.a.	n.a.	n.a.
	2	C27H43N2O10	555.291222	-3.5	-2.1	7.5	ok	even	10.7	17.7	n.a.	n.a.	n.a.	n.a.
	3	C32H43O8	555.295245	3.8	5.5	11.5	ok	even	13.0	19.9	n.a.	n.a.	n.a.	n.a.

Figure S47. ESITOFMS spectrum of compound **4**

Compound **12**

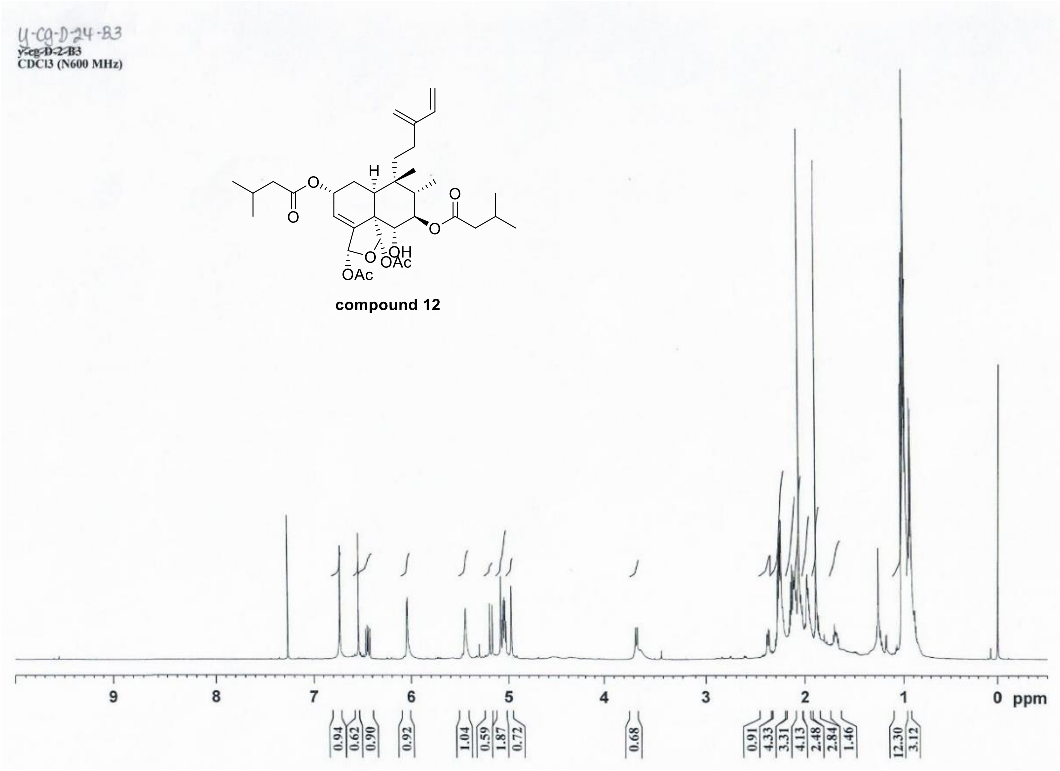

Figure S48. ^{1}H-NMR spectrum (600 MHz, $CDCl_3$) of compound **12**

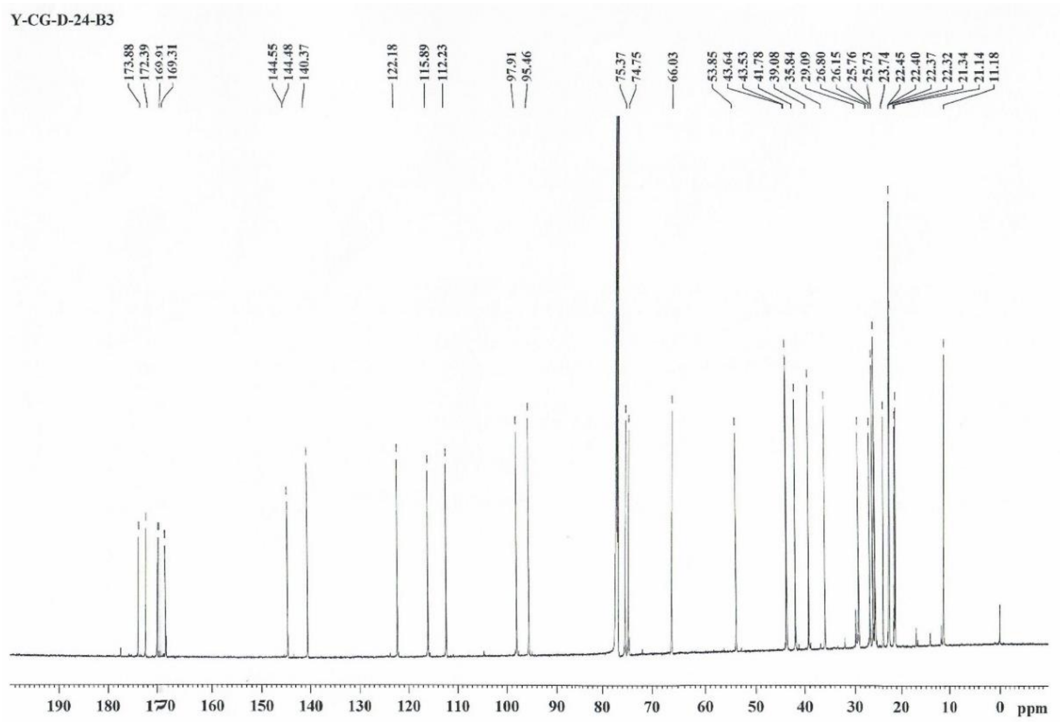

Figure S49. ^{13}C-NMR spectrum (150 MHz, $CDCl_3$) of compound **12**

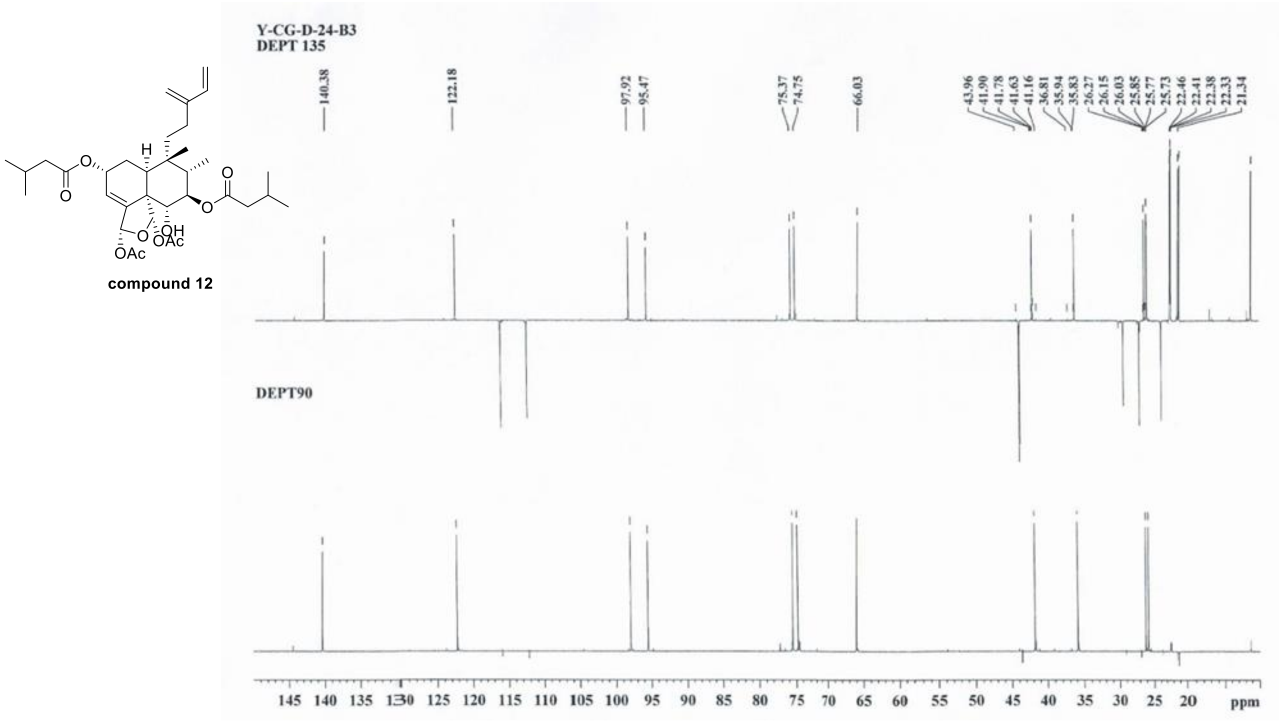

Figure S50. DEPT spectrum (600 MHz, $CDCl_3$) of compound **12**

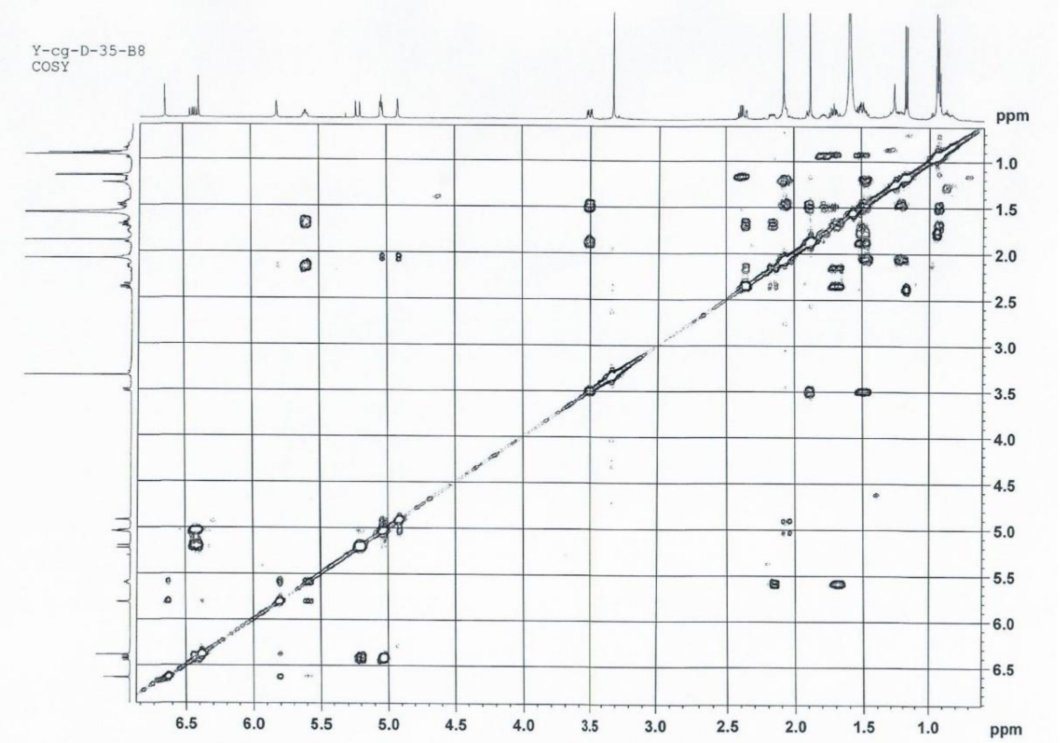

Figure S51. ^{1}H-^{1}H COSY spectrum (600 MHz, $CDCl_3$) of compound **12**

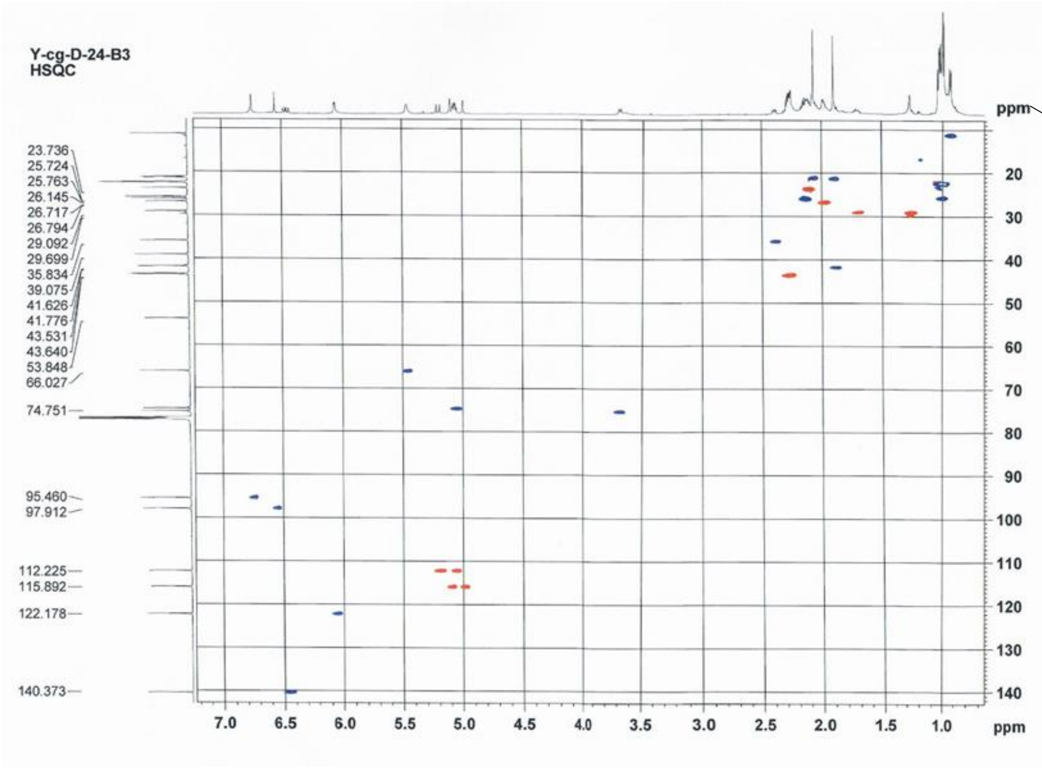

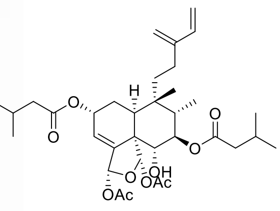

compound 12

Figure S52. HSQC spectrum (600 MHz, $CDCl_3$) of compound **12**

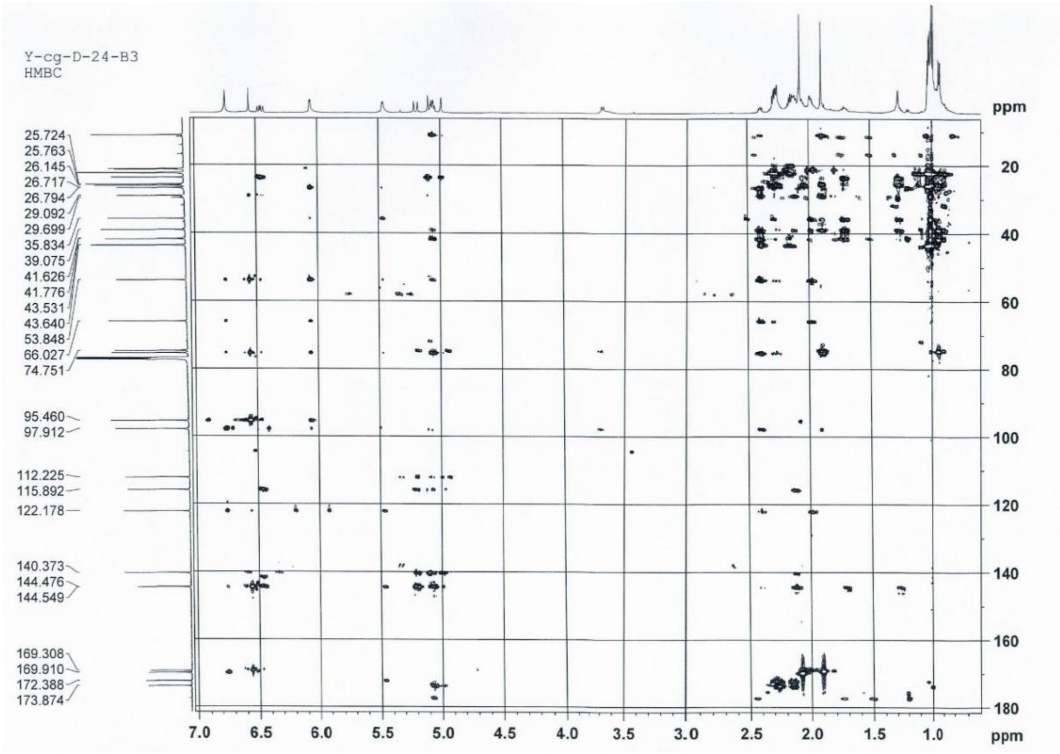

Figure S53. HMBC spectrum (600 MHz, $CDCl_3$) of compound **12**

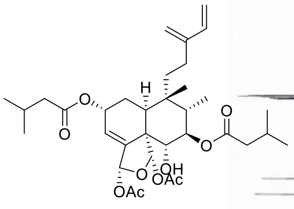

compound 12

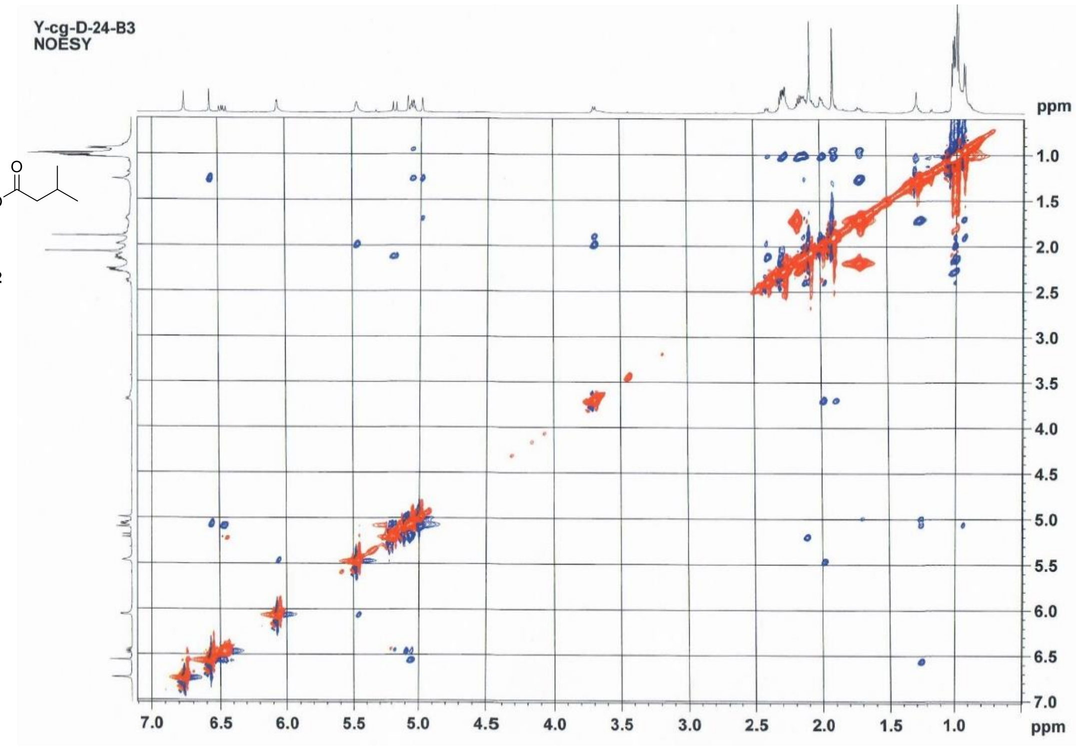

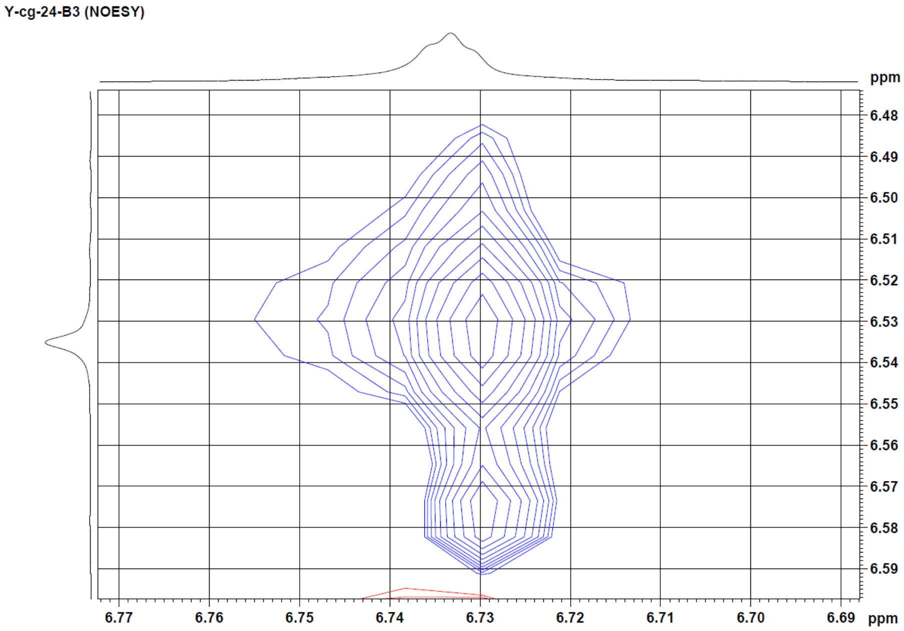

Figure S54. NOESY spectrum (600 MHz, $CDCl_3$) of compound **12**

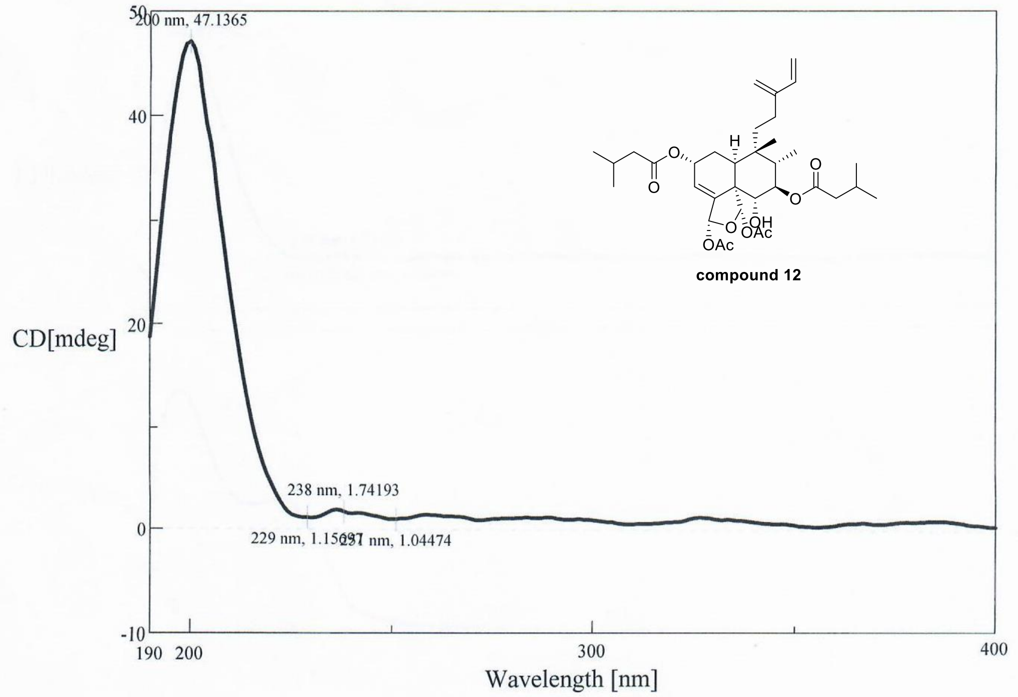

Date	8/4/2022 6:02PM
File name	Y-cg-D-24-B3_original high concentration_2
Model	J-815
Serial No.	A024561168
Band width	1 nm
Response	1 sec
Sensitivity	Standard
Measurement range	400 - 190 nm
Data pitch	1nm
Scanning speed	200 nm/min
Accumulation	1
Cell Length	1 cm
Solvent	ACN
Temperature	25 C
Sample name	Y-CG-D-24-B3 higher concentration
Operator	Vilailak
Comment	1 mg/2ml--250um/3750um

Figure S55. CD spectrum of compound **12**

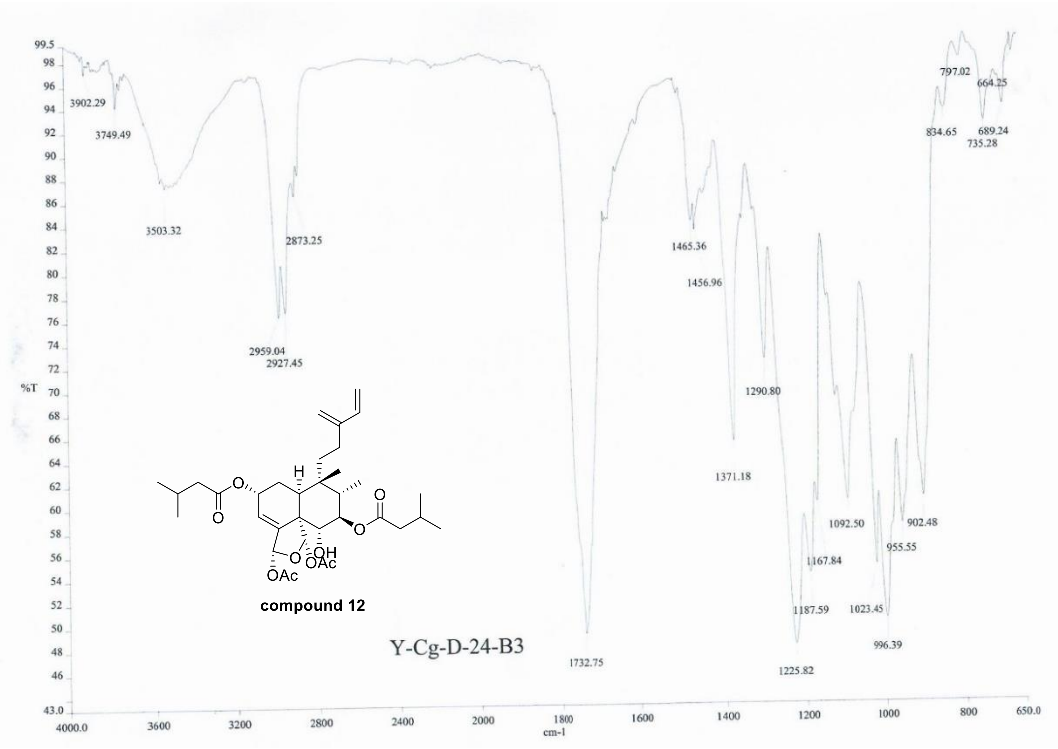

Figure S56. IR spectrum of compound **12**

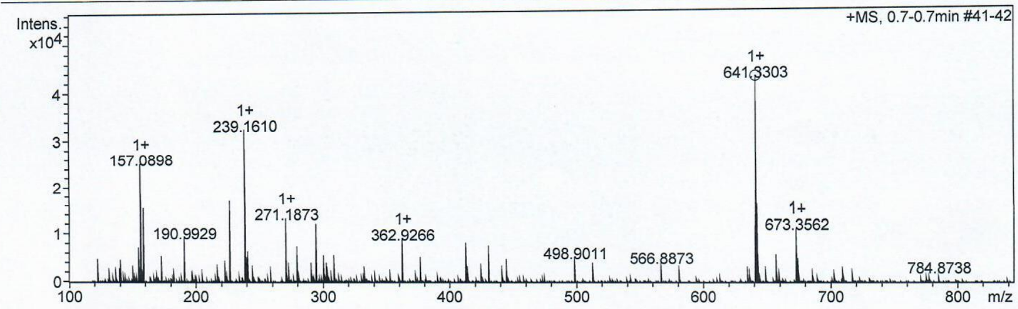

Mass Spectrum SmartFormula Report

Analysis Info

Analysis Name C:\Users\Admin\Desktop\Results from MTof\MTCRI26035 Vilailak Y-cgD-24B3 E+.d
Method Nitirat ESIpos 2021.m
Sample Name ESIpos
Comment

Acquisition Date 3/25/2022 12:51:45 AM
Operator Administrator
Instrument micrOTOF

Acquisition Parameter

Source Type	ESI	Ion Polarity	Positive	Set Nebulizer	0.5 Bar
Focus	Not active			Set Dry Heater	150 °C
Scan Begin	100 m/z	Set Capillary	3000 V	Set Dry Gas	5.0 l/min
Scan End	800 m/z	Set End Plate Offset	-500 V	Set Divert Valve	Source

Meas. m/z	#	Ion Formula	m/z	err [ppm]	Mean err [ppm]	rdb	N-Rule	e⁻ Conf	mSigma	Std I	Std Mean m/z	Std I VarNorm	Std m/z Diff	Std Comb Dev
641.330292	1	C34H50NaO10	641.329619	-1.0	3.0	9.5	ok	even	13.6	22.6	n.a.	n.a.	n.a.	n.a.
	2	C36H49O10	641.332024	2.7	6.8	12.5	ok	even	15.6	20.5	n.a.	n.a.	n.a.	n.a.
	3	C31H49N2O12	641.328001	-3.6	0.3	8.5	ok	even	18.9	33.2	n.a.	n.a.	n.a.	n.a.
	4	C43H45O5	641.326151	-6.5	-2.2	21.5	ok	even	49.3	61.5	n.a.	n.a.	n.a.	n.a.

Figure S57. ESITOFMS spectrum of compound **12**

Compound **13**

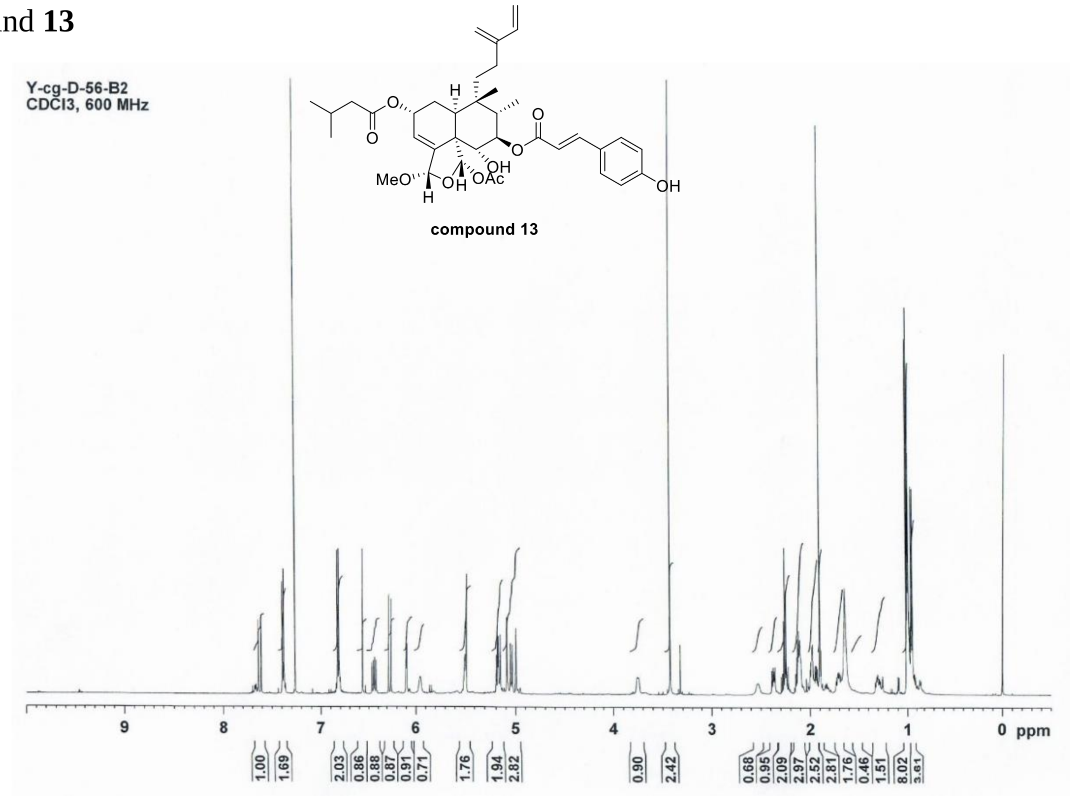

Figure S58. ^{1}H-NMR spectrum (600 MHz, $CDCl_3$) of compound **13**

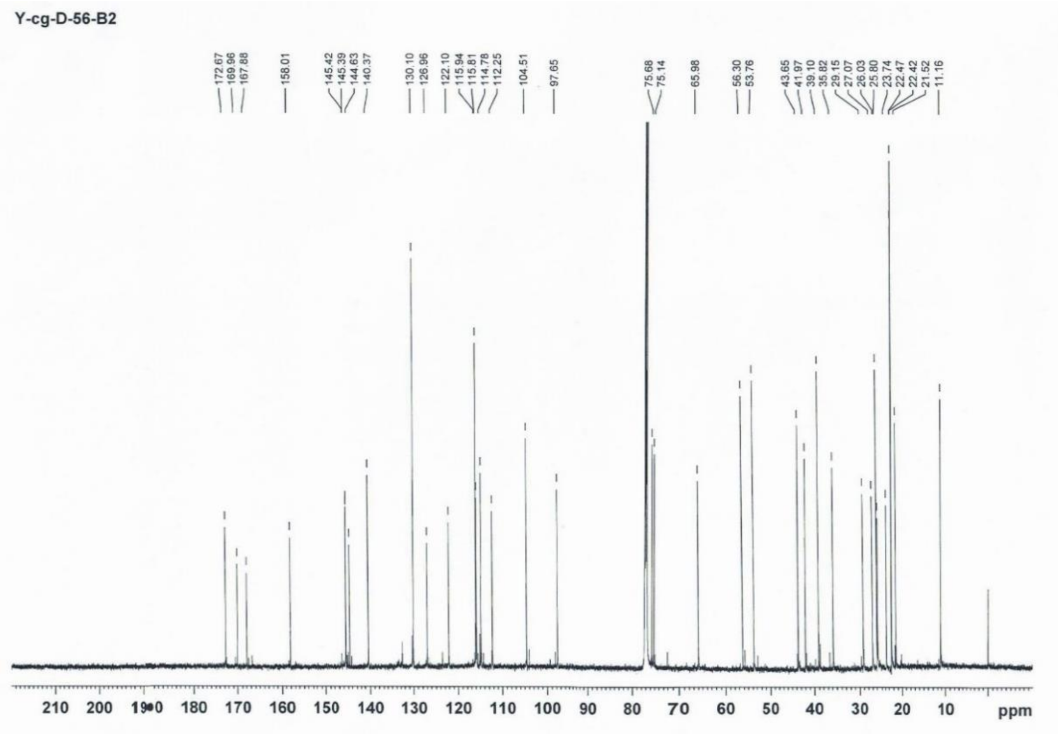

Figure S59. ^{13}C-NMR spectrum (150 MHz, $CDCl_3$) of compound **13**

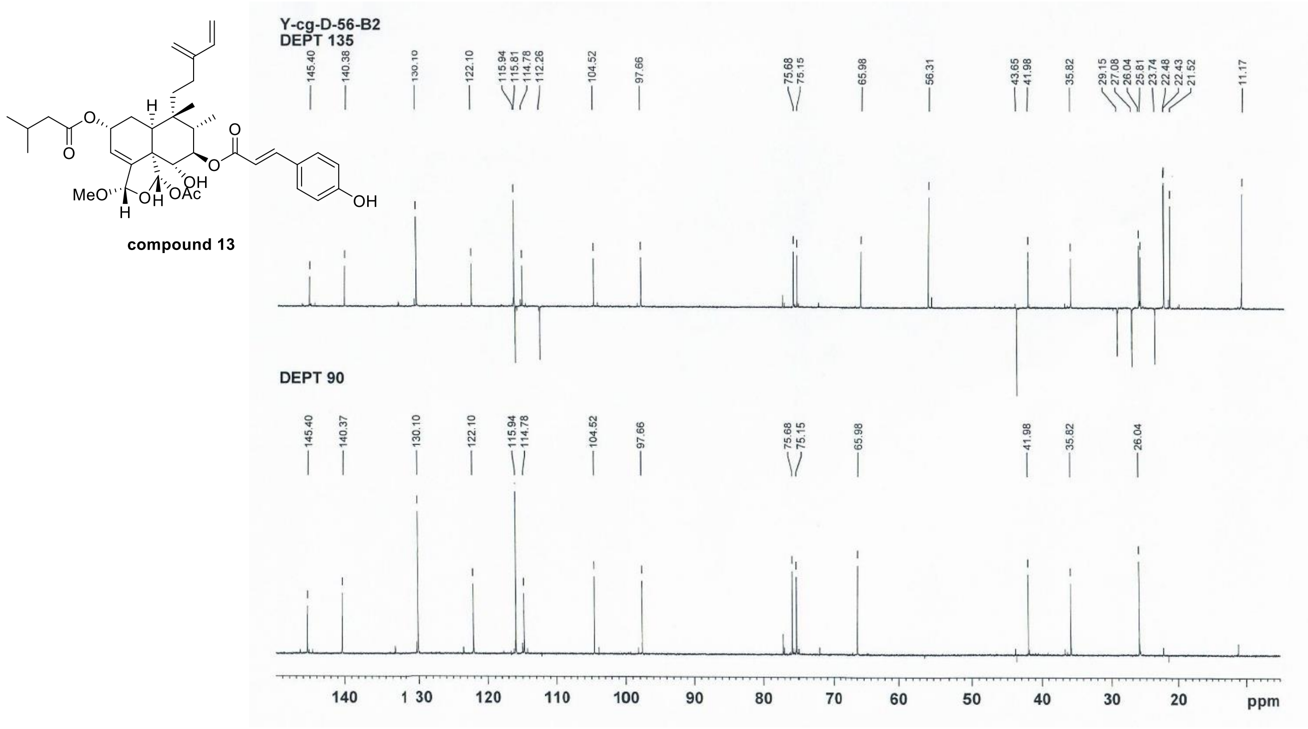

Figure S60. DEPT spectrum (600 MHz, $CDCl_3$) of compound **13**

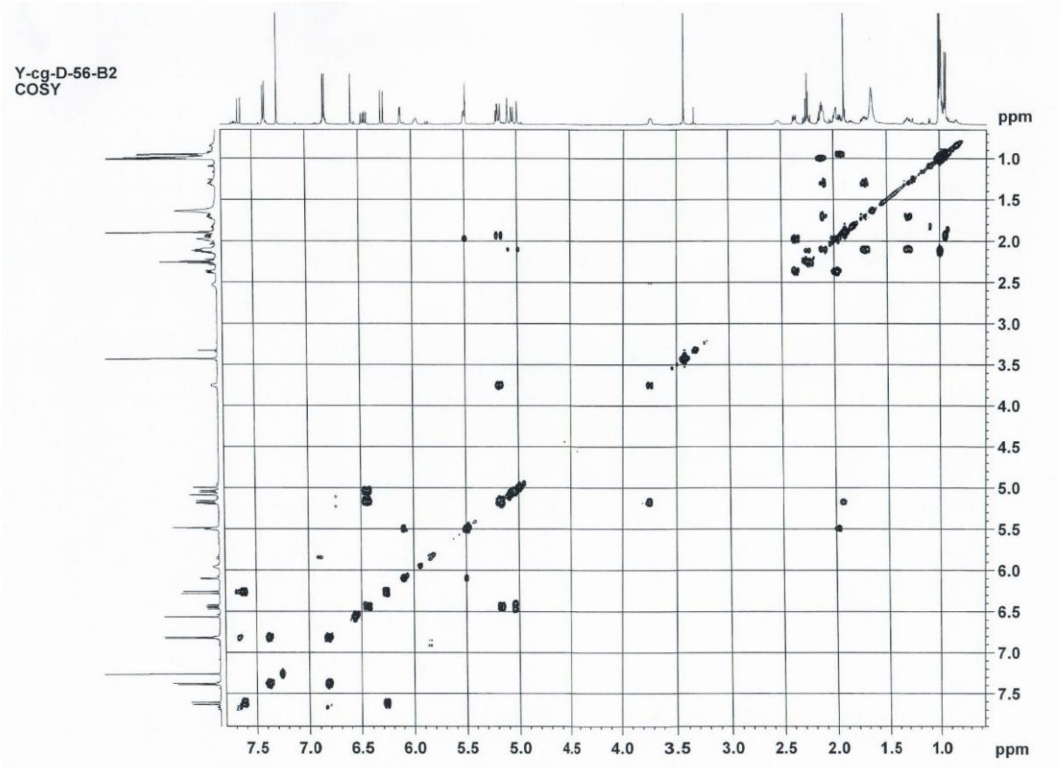

Figure S61. ^1H-^1H COSY spectrum (600 MHz, $CDCl_3$) of compound **13**

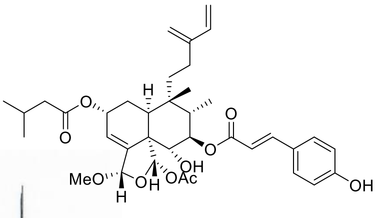

compound 13

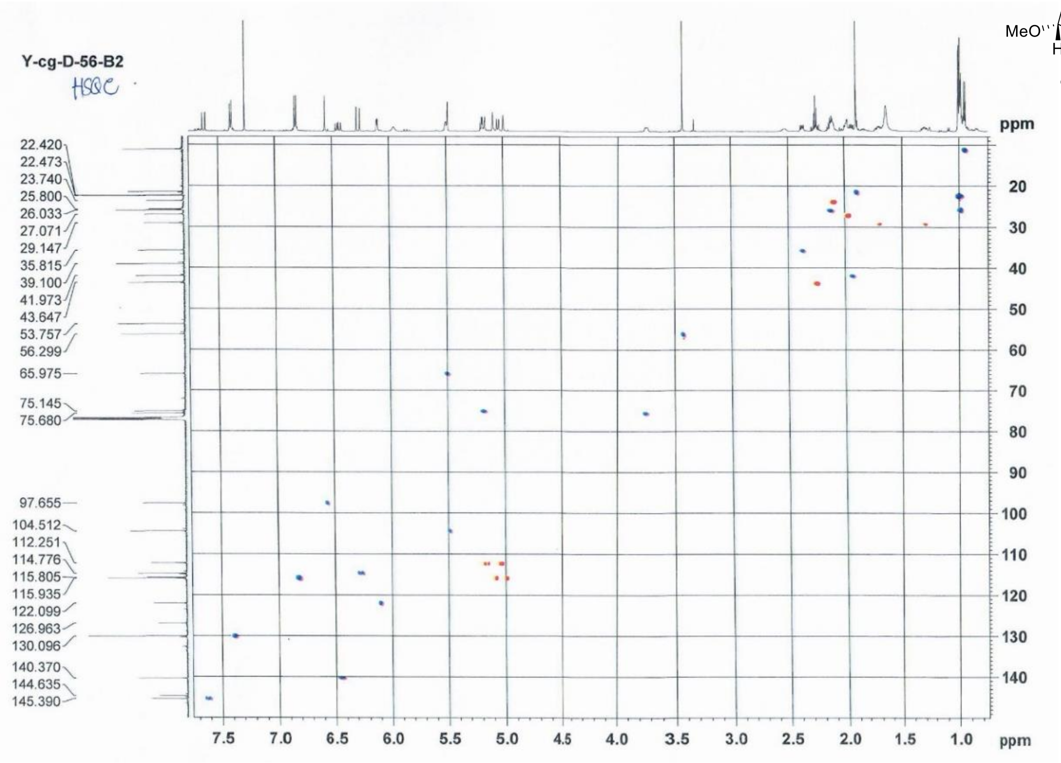

Figure S62. HSQC spectrum (600 MHz, $CDCl_3$) of compound **13**

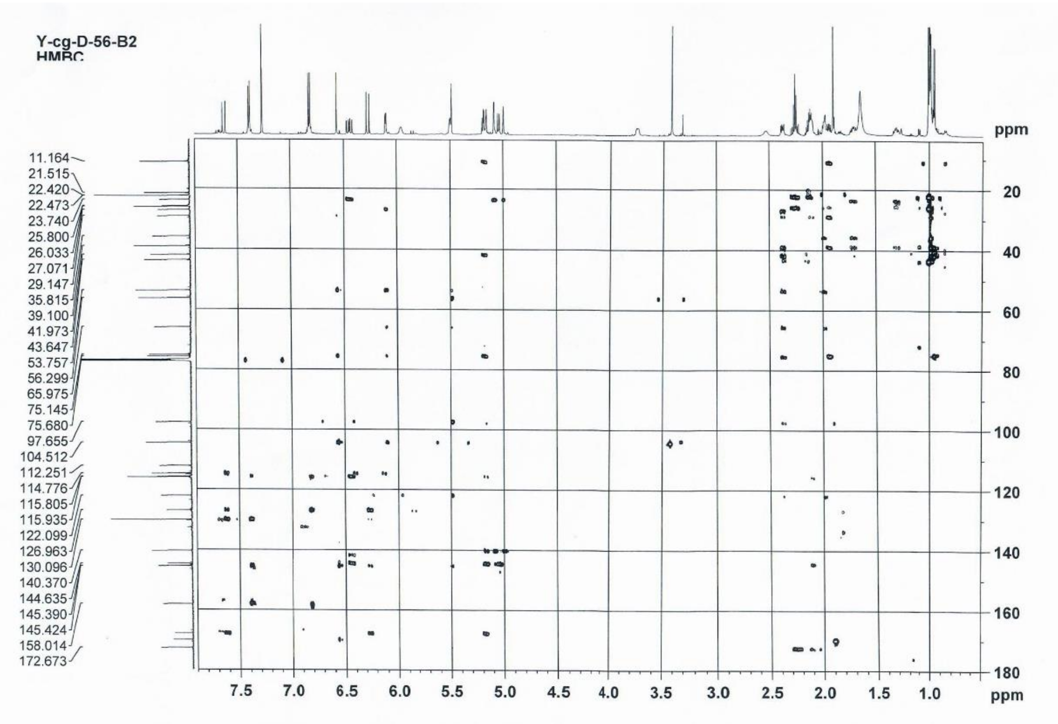

Figure S63. HMBC spectrum (600 MHz, $CDCl_3$) of compound **13**

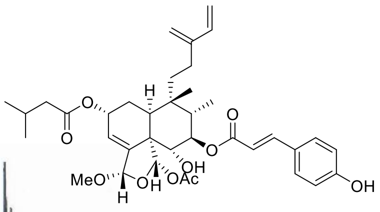

compound **13**

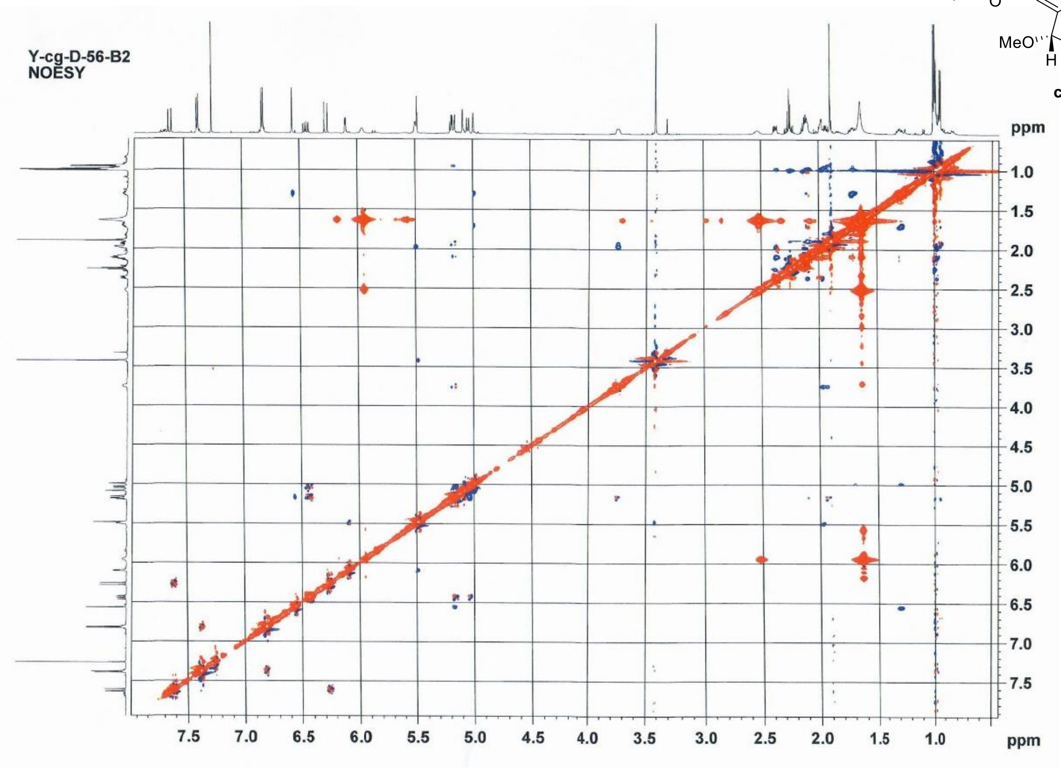

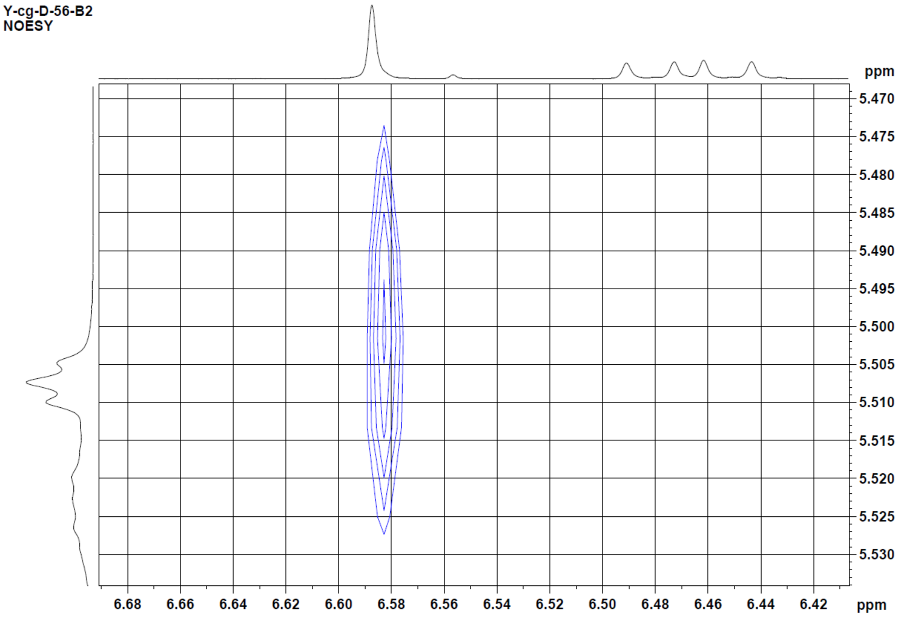

Figure S64. NOESY spectrum (600 MHz, $CDCl_3$) of compound **13**

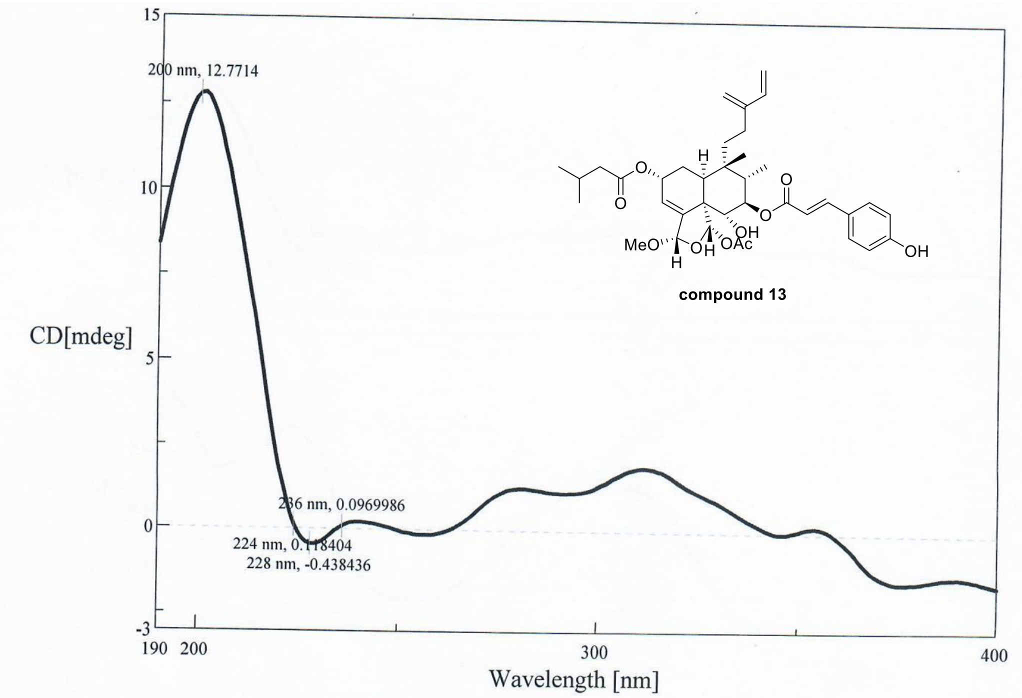

Date	5/11/2023 4:12PM
File name	Memory#24
Model	J-815
Serial No.	A024561168
Band width	1 nm
Response	1 sec
Sensitivity	Standard
Measurement range	400 - 190 nm
Data pitch	1nm
Scanning speed	200 nm/min
Accumulation	2
Cell Length	1 cm
Solvent	ACN
Temperature	Room Temperature
Sample name	Y-cg-D-56-B2
Operator	vilaialk
Comment	1 mg in 2 ml ACN_400um/4600 ml

Figure S65. CD spectrum of compound **13**

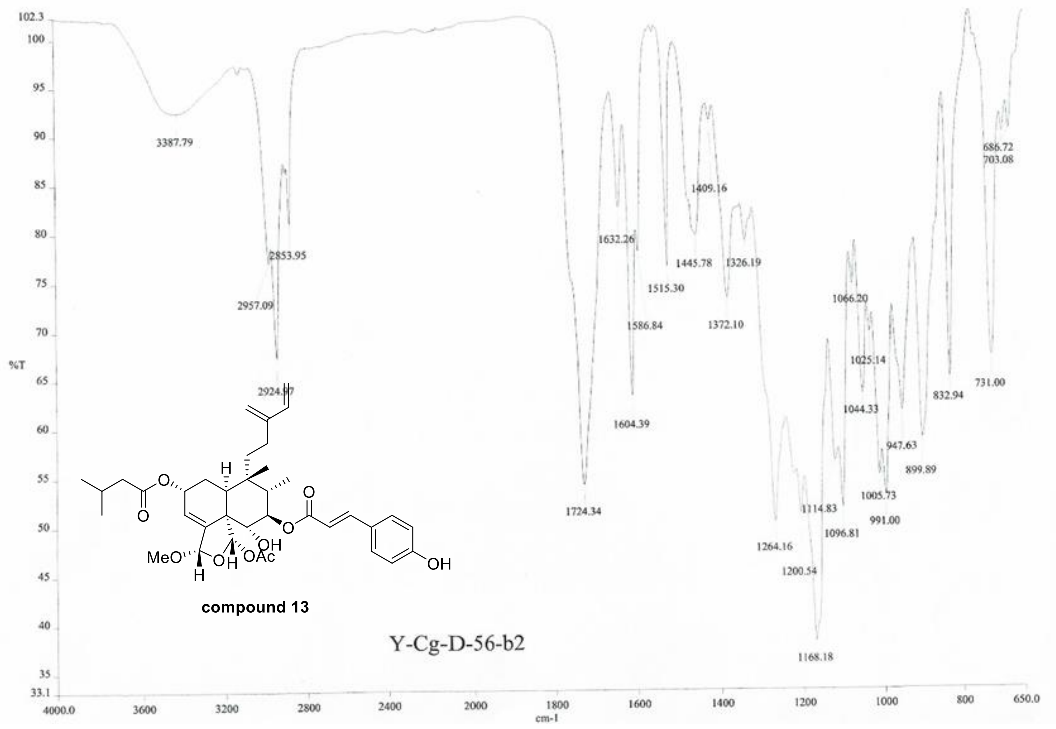

Figure S66. IR spectrum of compound **13**

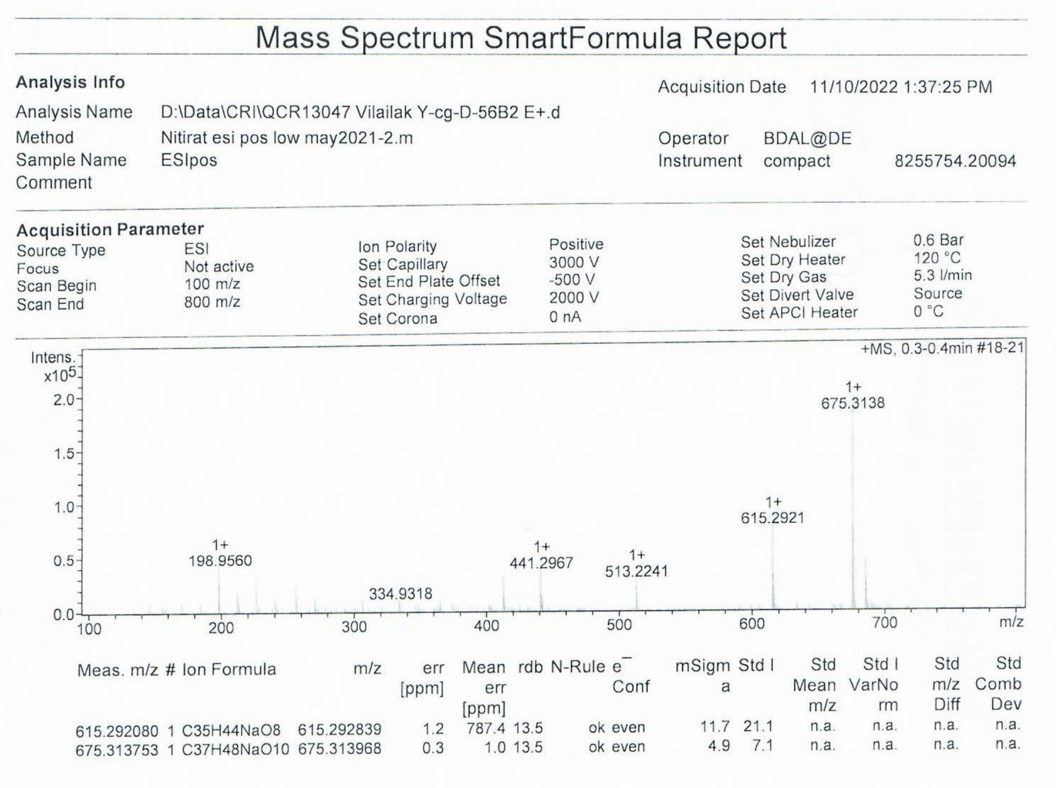

Mass Spectrum SmartFormula Report

Analysis Info

Analysis Name D:\Data\CRI\QCR13047 Vilailak Y-cg-D-56B2 E+.d
Method Nitirat esi pos low may2021-2.m
Sample Name ESIpos
Comment

Acquisition Date 11/10/2022 1:37:25 PM
Operator BDAL@DE
Instrument compact 8255754.20094

Acquisition Parameter

Source Type	ESI	Ion Polarity	Positive	Set Nebulizer	0.6 Bar
Focus	Not active	Set Capillary	3000 V	Set Dry Heater	120 °C
Scan Begin	100 m/z	Set End Plate Offset	-500 V	Set Dry Gas	5.3 l/min
Scan End	800 m/z	Set Charging Voltage	2000 V	Set Divert Valve	Source
		Set Corona	0 nA	Set APCI Heater	0 °C

Meas. m/z	#	Ion Formula	m/z	err [ppm]	Mean err [ppm]	rdb	N-Rule	e⁻ Conf	mSigma	Std I	Std Mean m/z	Std I VarNorm	Std m/z Diff	Std Comb Dev
615.292080	1	C35H44NaO8	615.292839	1.2	787.4	13.5	ok	even	11.7	21.1	n.a.	n.a.	n.a.	n.a.
675.313753	1	C37H48NaO10	675.313968	0.3	1.0	13.5	ok	even	4.9	7.1	n.a.	n.a.	n.a.	n.a.

Figure S67. ESITOFMS spectrum of compound **13**

Compound **14**

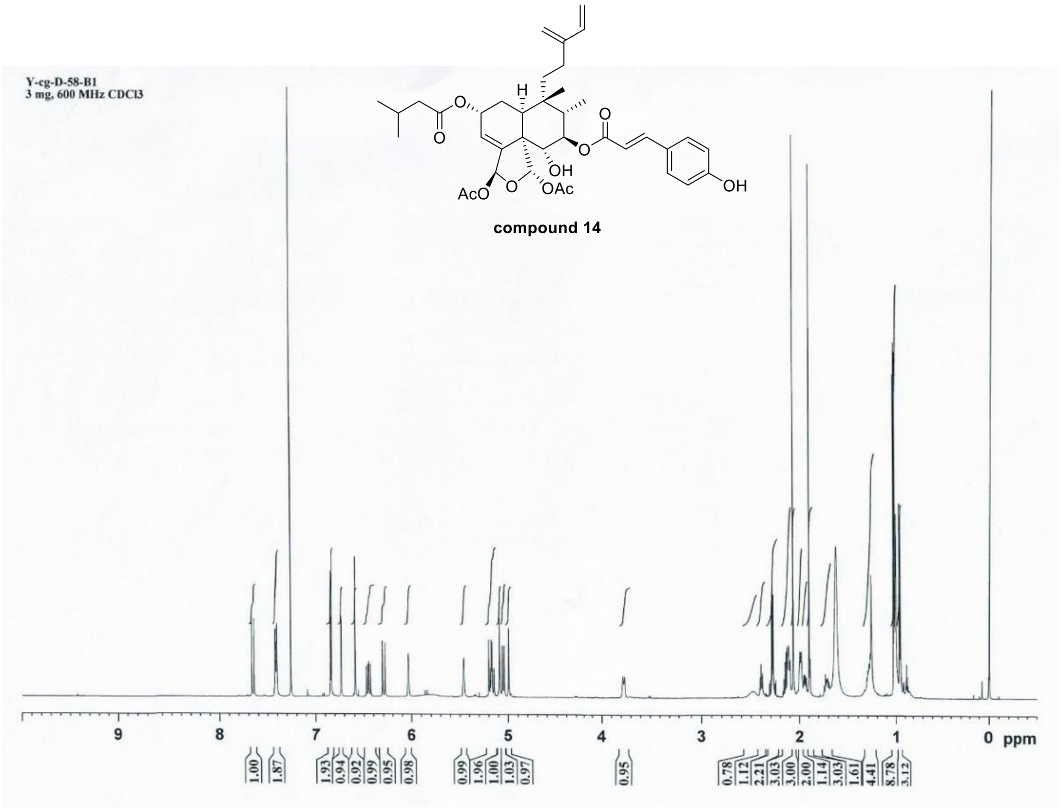

Figure S68. ^{1}H-NMR spectrum (600 MHz, $CDCl_3$) of compound **14**

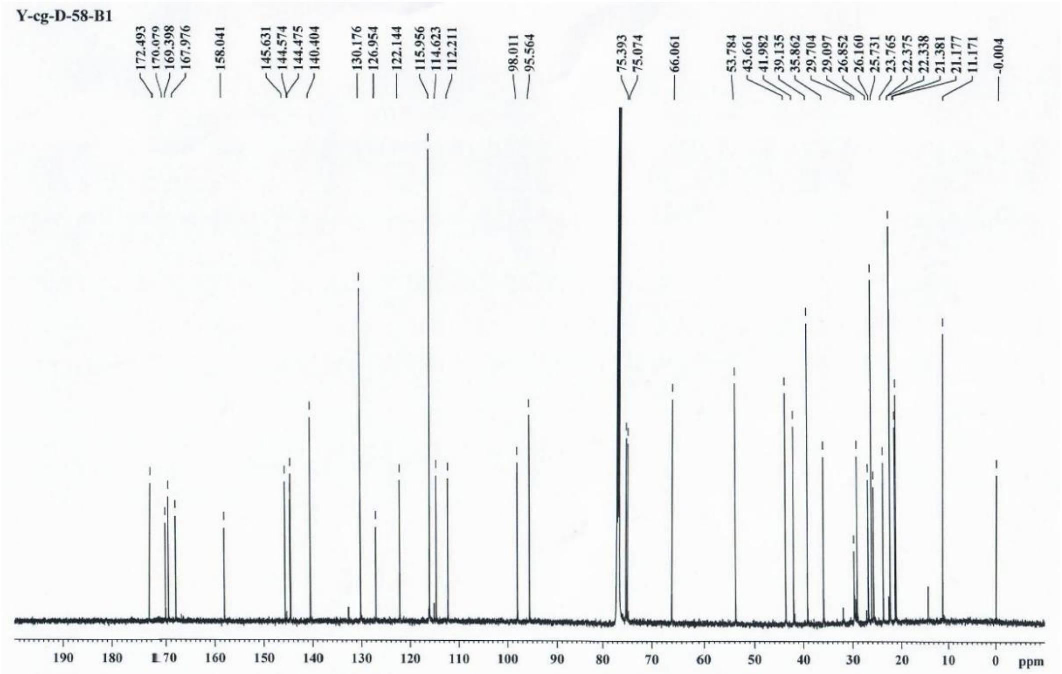

Figure S69. ^{13}C-NMR spectrum (150 MHz, $CDCl_3$) of compound **14**

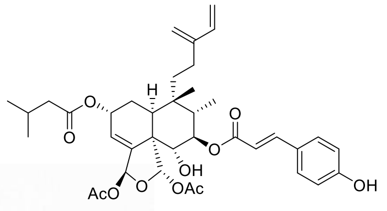

compound 14

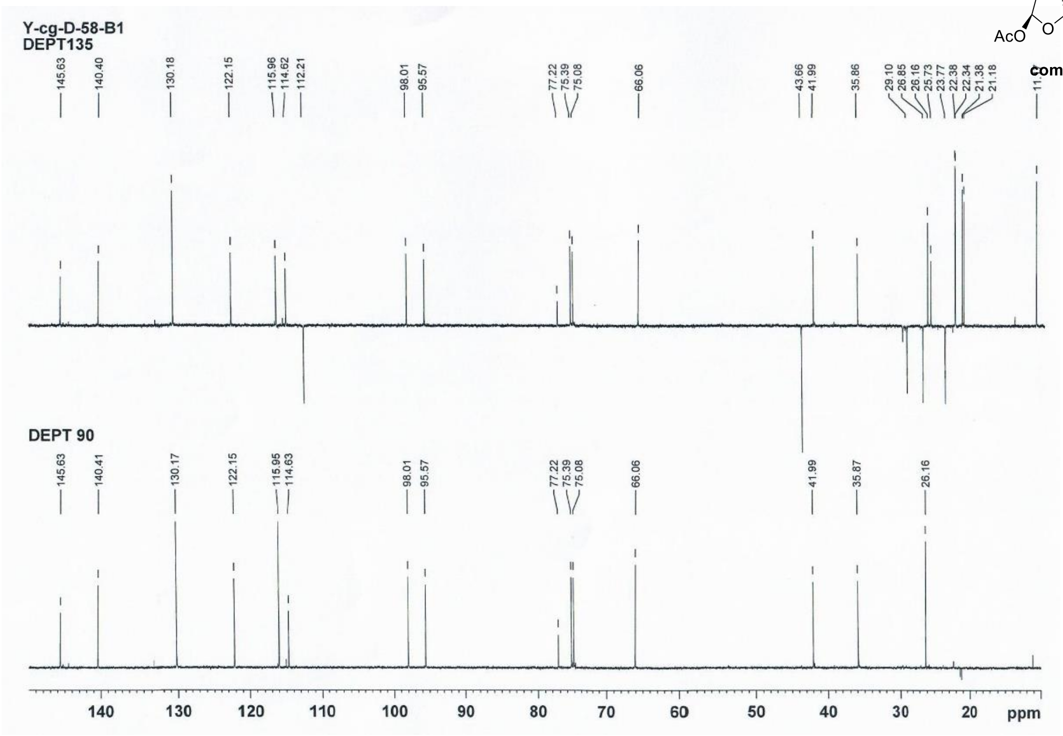

Figure S70. DEPT spectrum (600 MHz, $CDCl_3$) of compound **14**

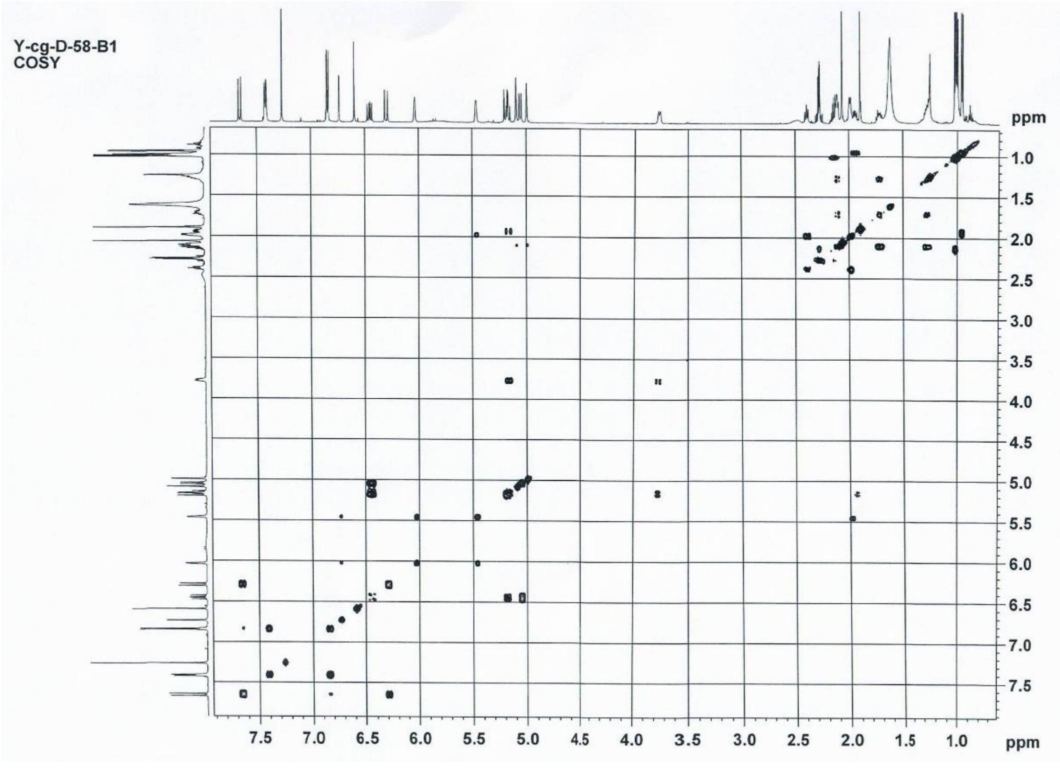

Figure S71. ^1H-^1H COSY spectrum (600 MHz, $CDCl_3$) of compound **14**

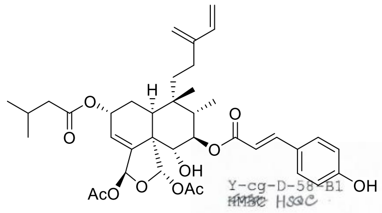

compound 14

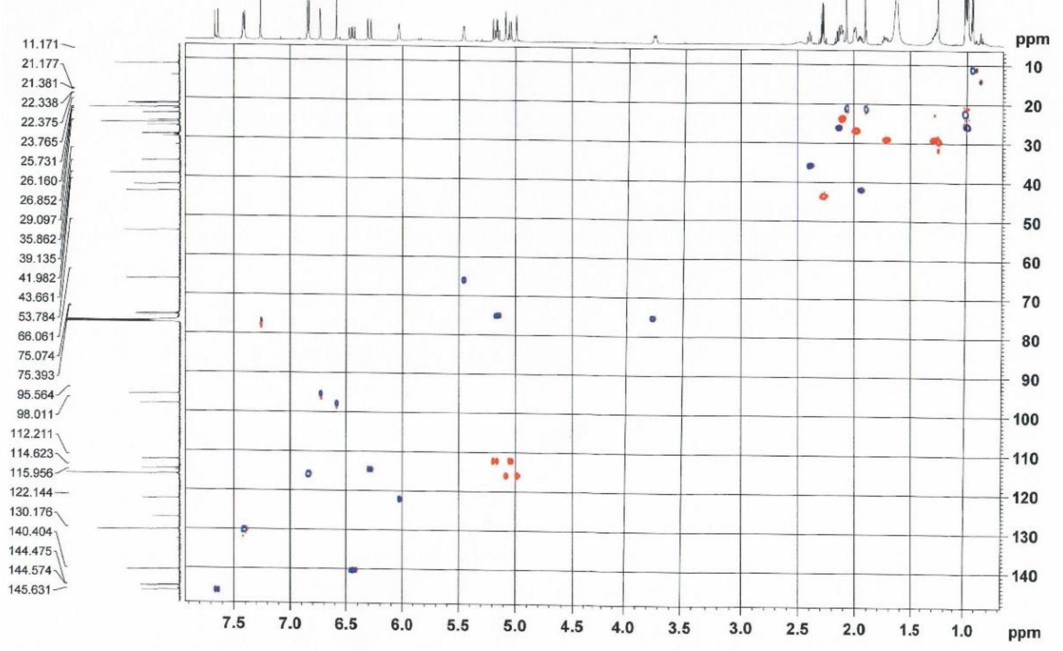

Figure S72. HSQC spectrum (600 MHz, $CDCl_3$) of compound **14**

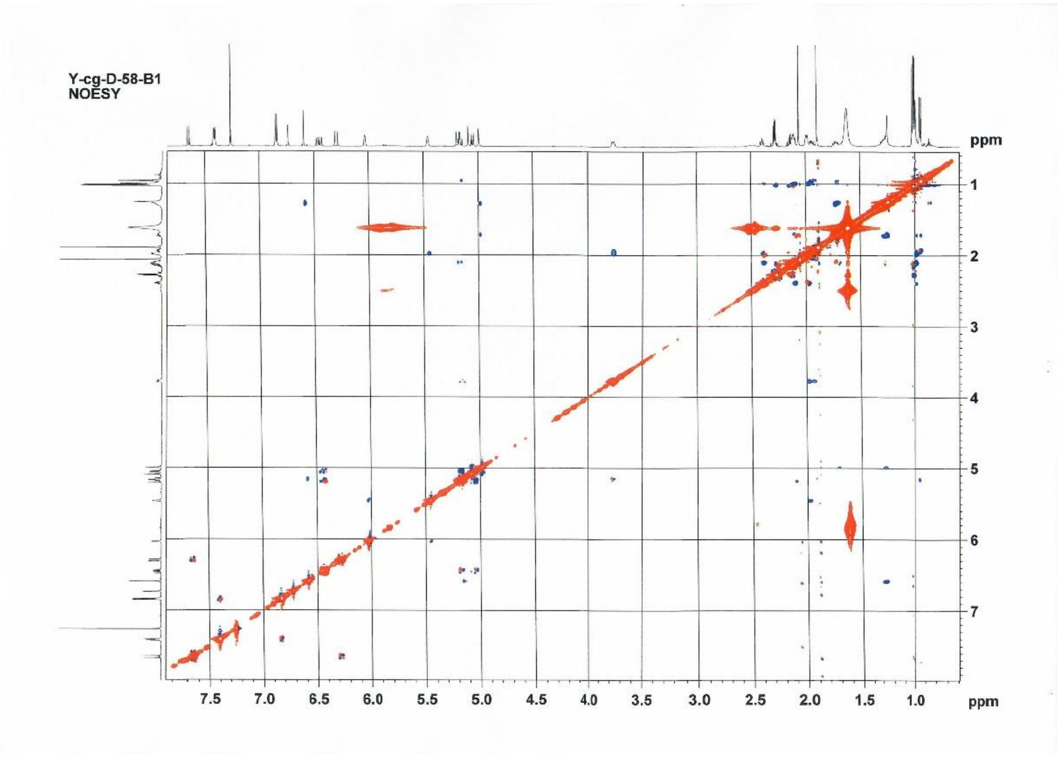

Figure S73. HMBC spectrum (600 MHz, $CDCl_3$) of compound **14**

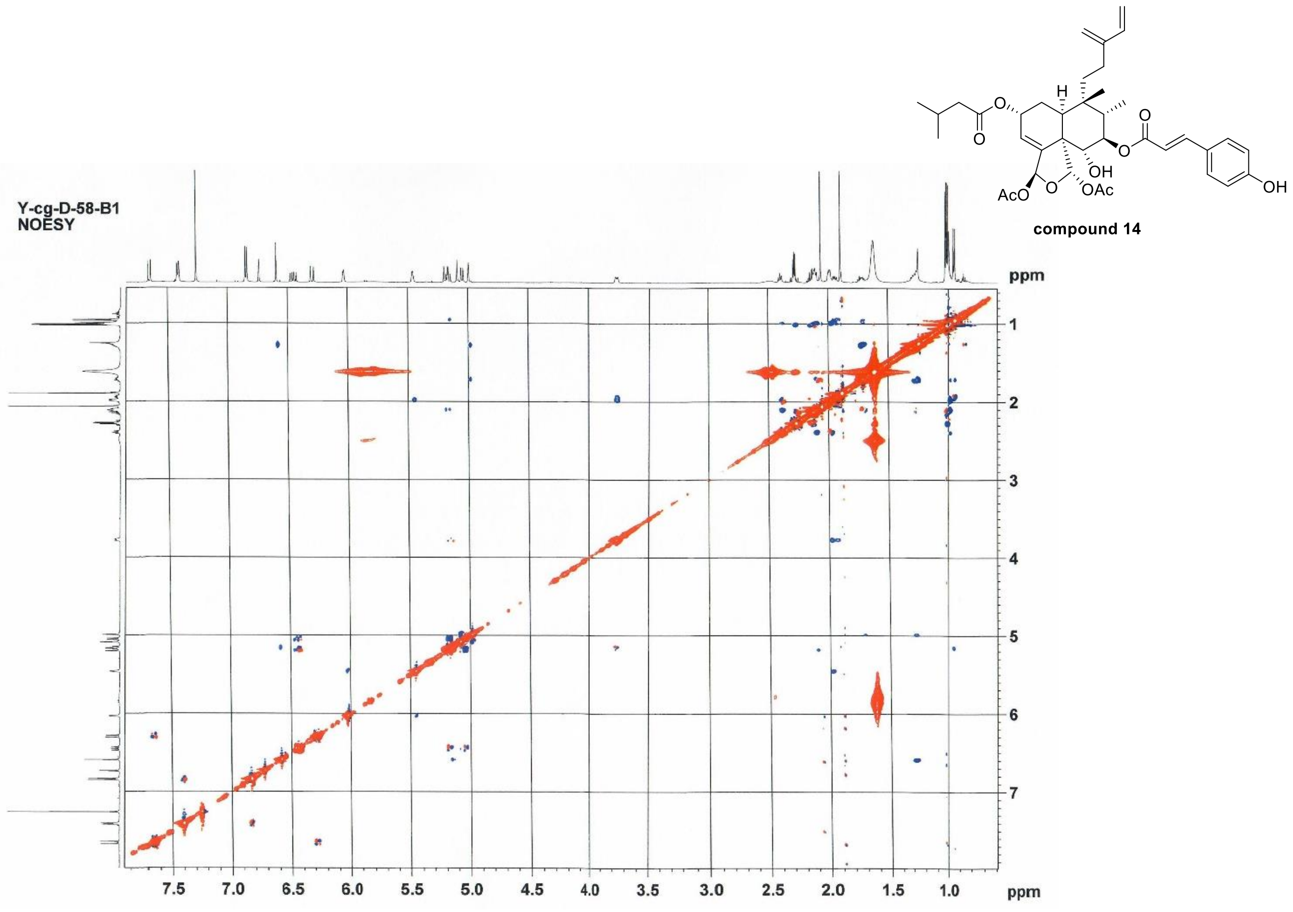

Figure S74. NOESY spectrum (600 MHz, $CDCl_3$) of compound **14**

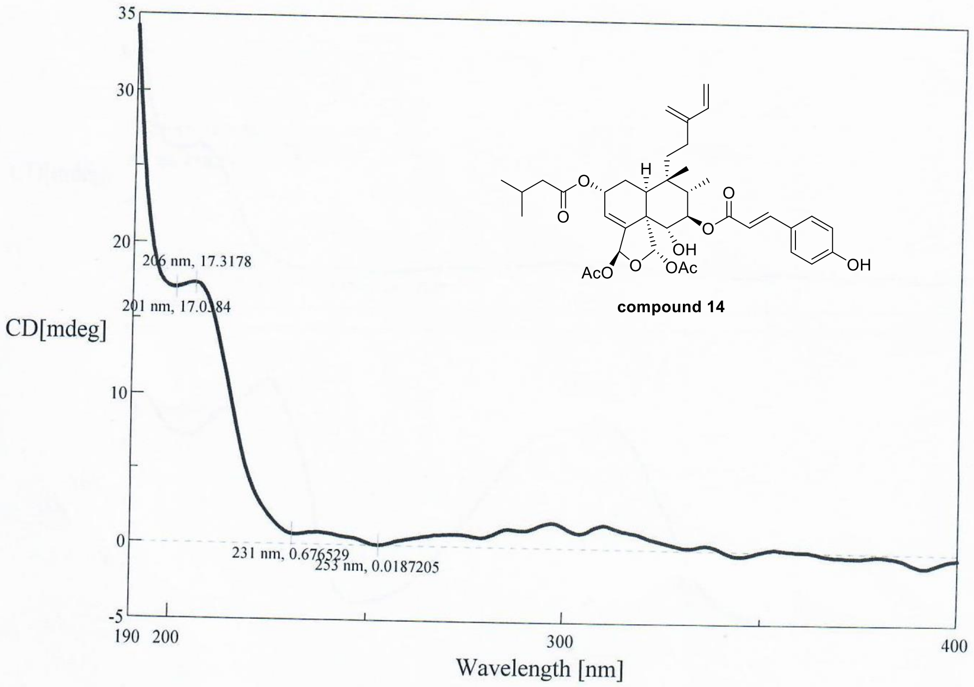

Date	11/29/2022 7:07PM
File name	Y-cg-D-58-B1 150u (process 1)
Model	J-815
Serial No.	A024561168
Band width	1 nm
Response	1 sec
Sensitivity	Standard
Measurement range	400 - 190 nm
Data pitch	1nm
Scanning speed	200 nm/min
Accumulation	2
Temperature	24.9 C
Sample name	Y-cg-D-58-B1 150u
Operator	Vilailak
Comment	3.6 mg in 3 ml ACN 150u/3850u

Figure S75. CD spectrum of compound **14**

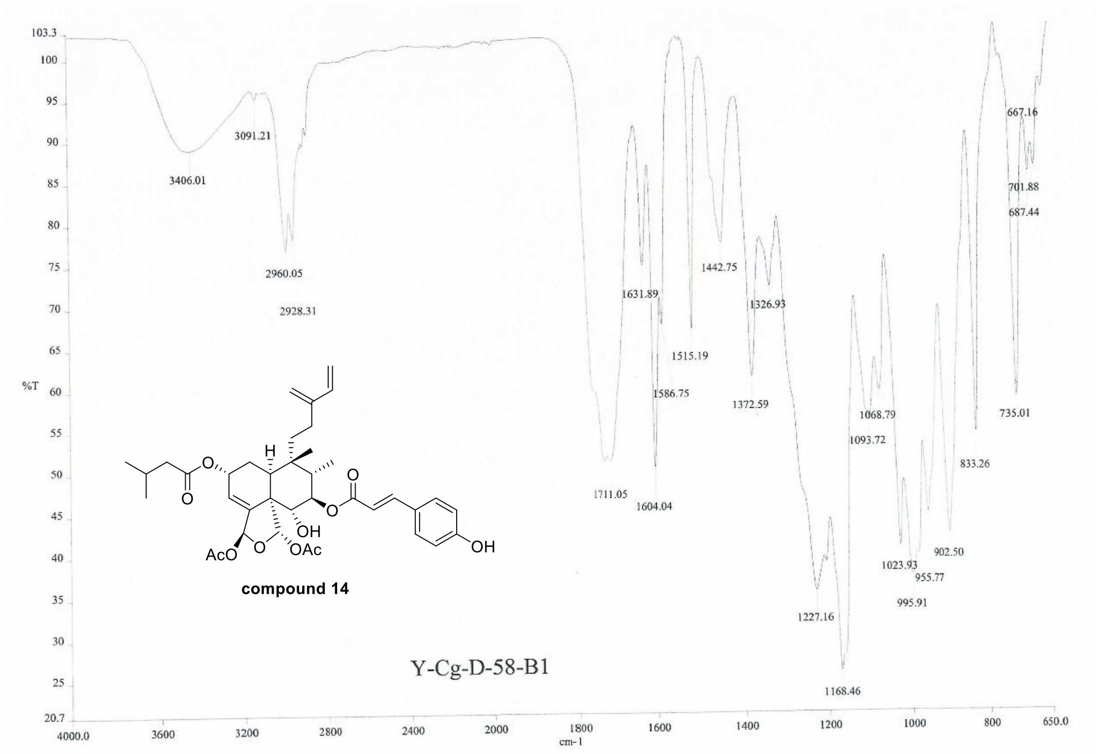

Figure S76. IR spectrum of compound **14**

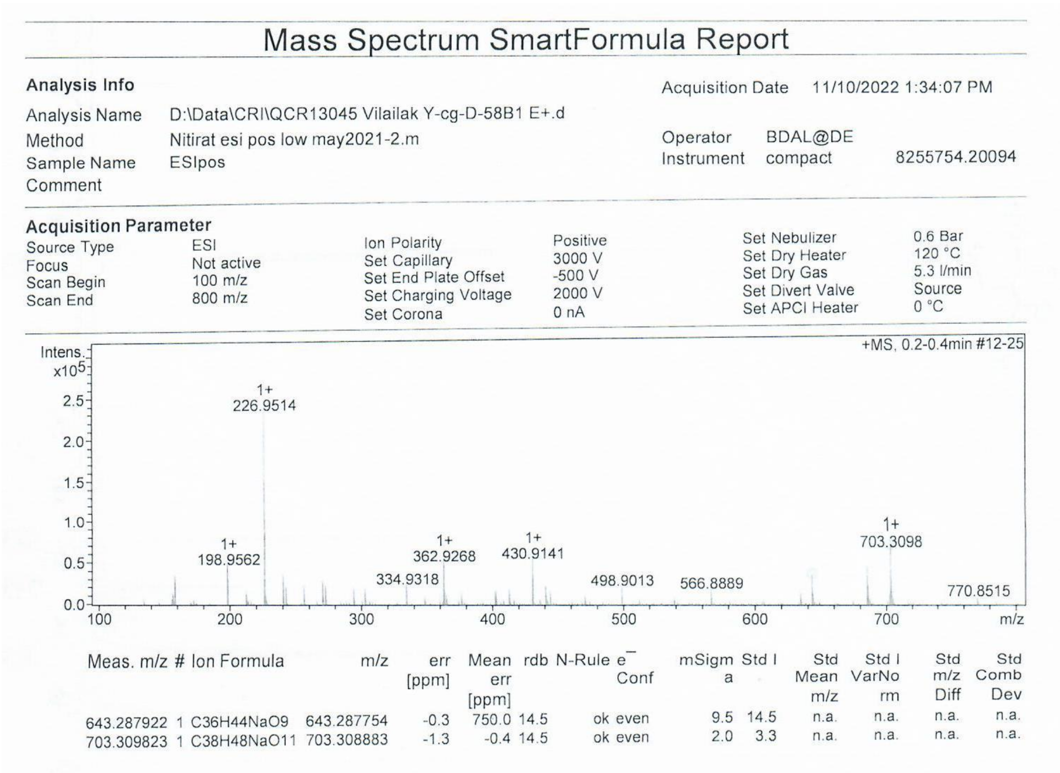

Figure S77. ESITOFMS spectrum of compound **14**

Compound **15**

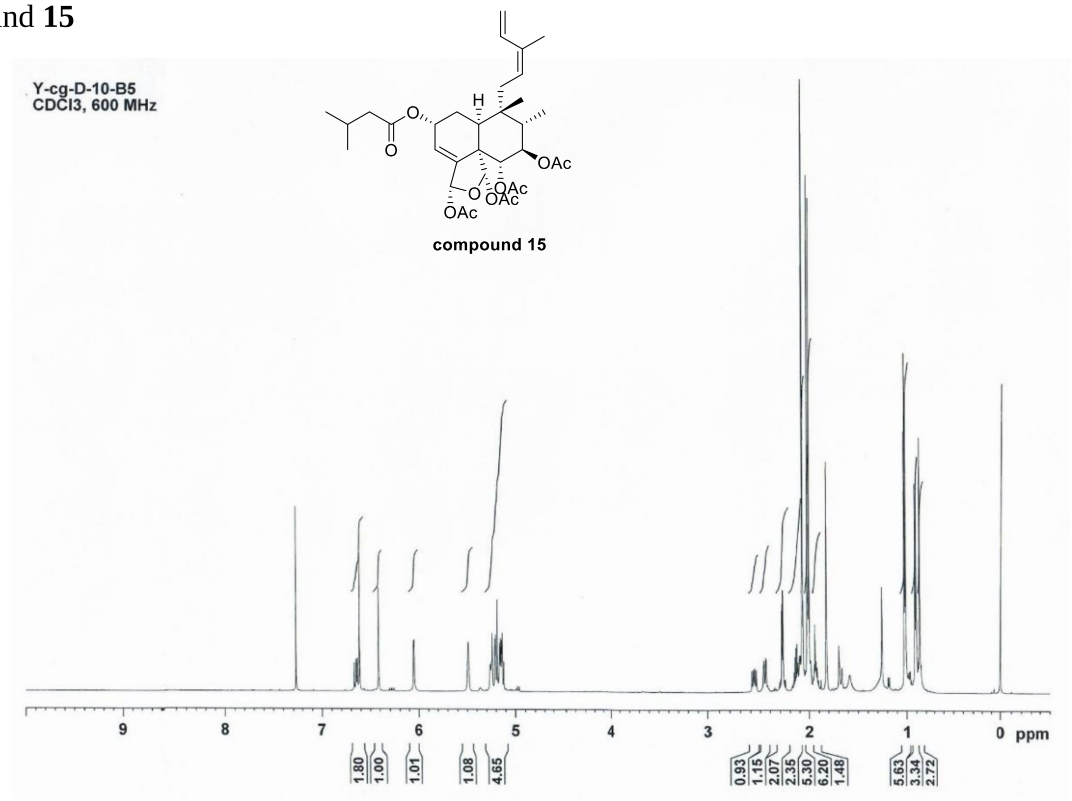

Figure S78. ^{1}H-NMR spectrum (600 MHz, $CDCl_3$) of compound **15**

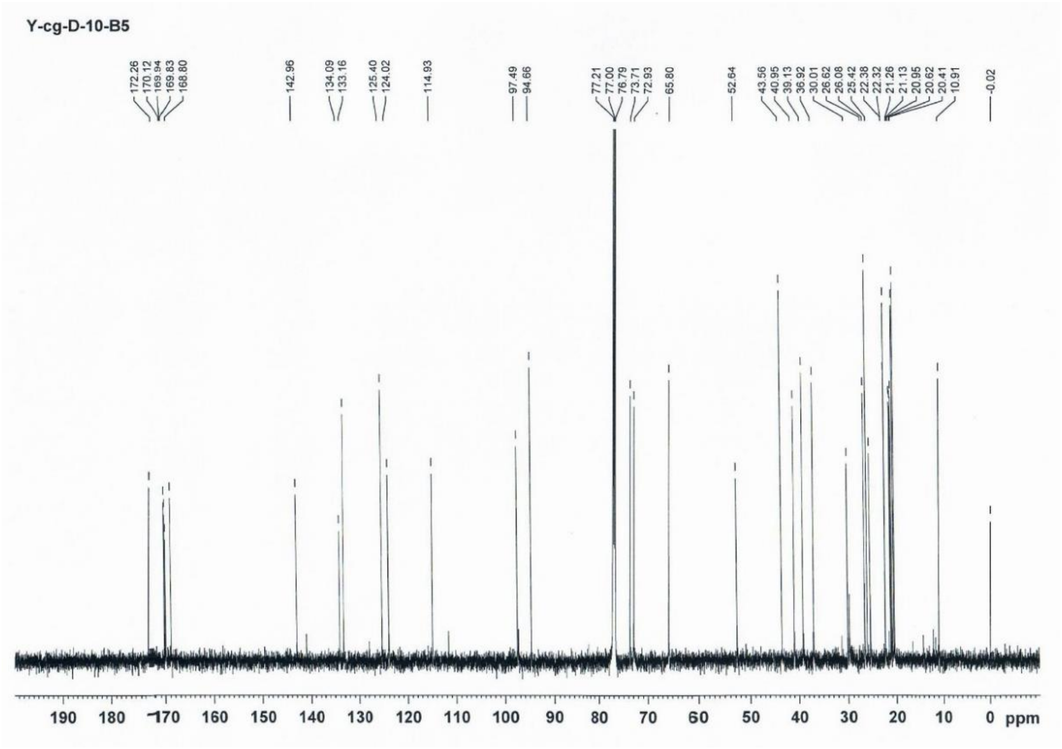

Figure S79. ^{13}C-NMR spectrum (150 MHz, $CDCl_3$) of compound **15**

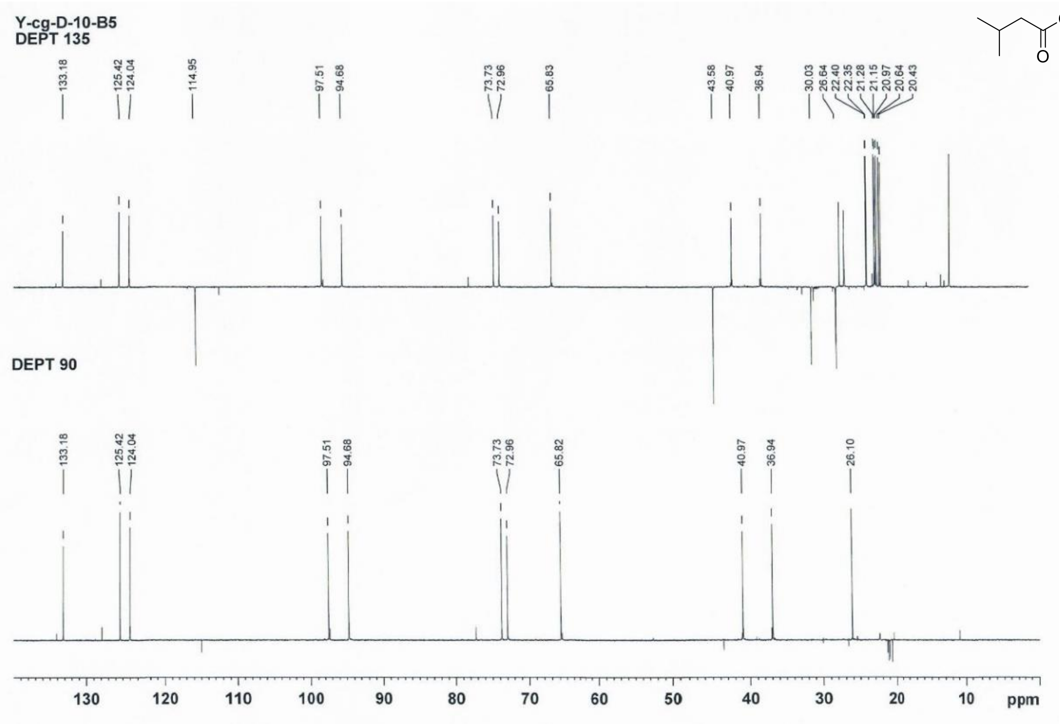

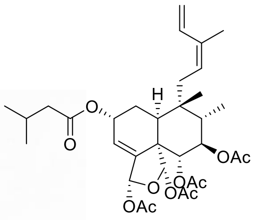

compound 15

Figure S80. DEPT spectrum (600 MHz, $CDCl_3$) of compound **15**

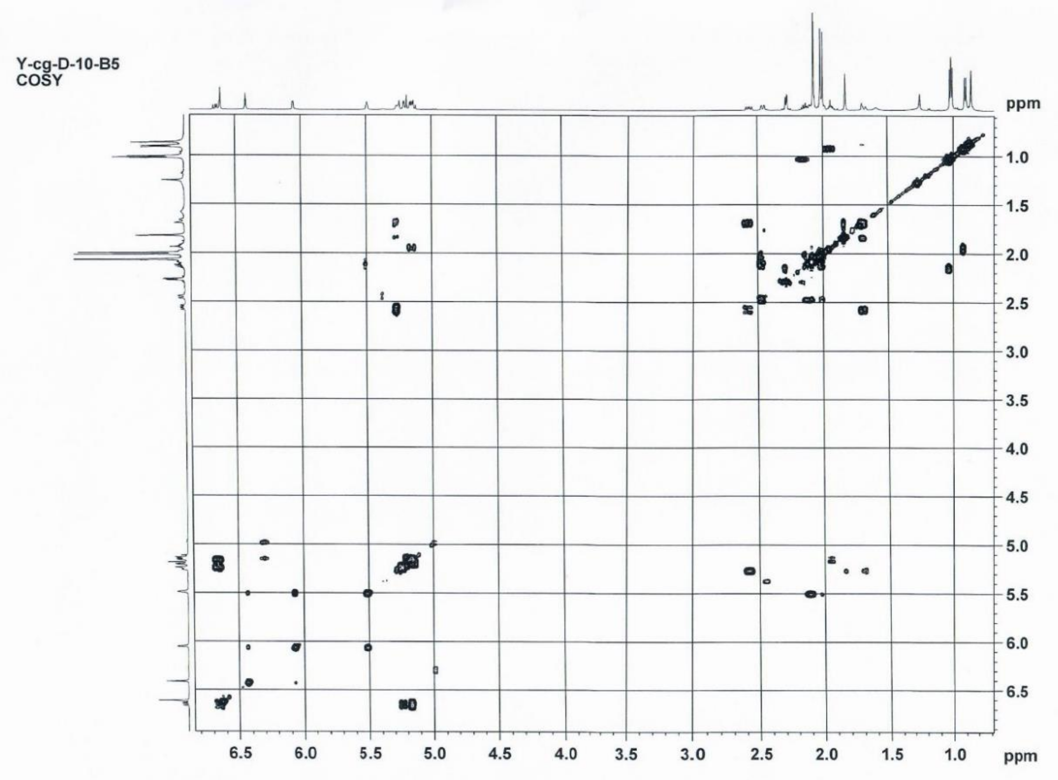

Figure S81. ^{1}H-^{1}H COSY spectrum (600 MHz, $CDCl_3$) of compound **15**

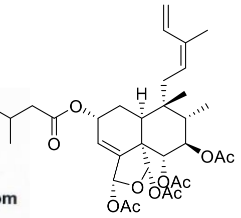

compound **15**

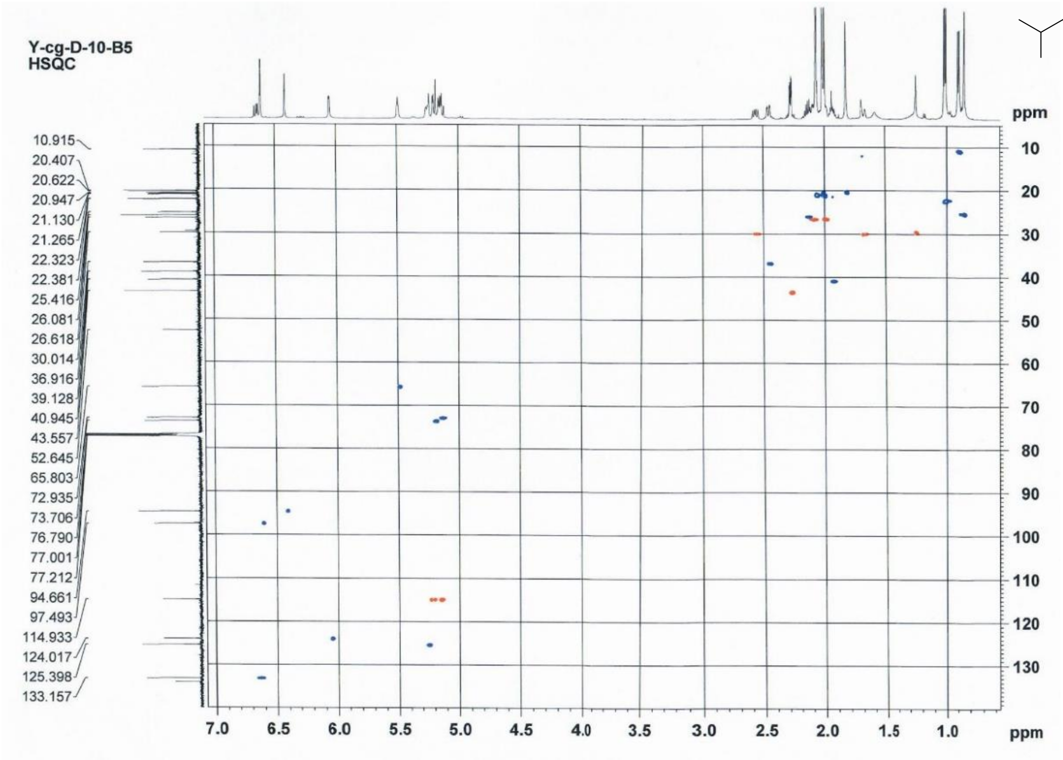

Figure S82. HSQC spectrum (600 MHz, $CDCl_3$) of compound **15**

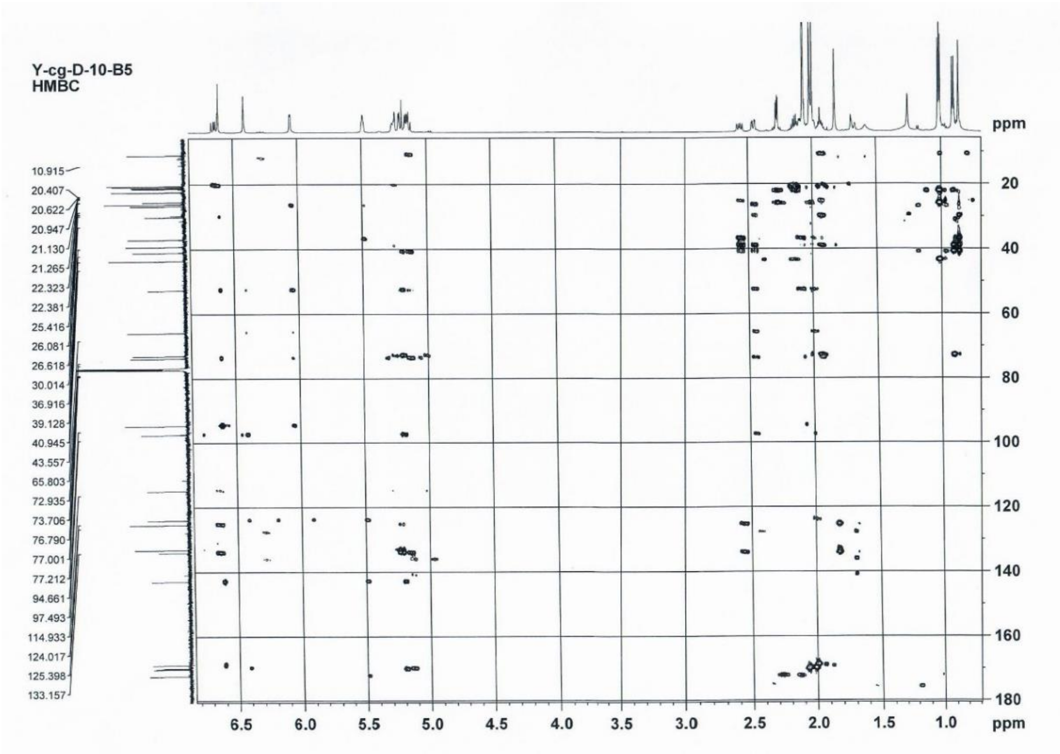

Figure S83. HMBC spectrum (600 MHz, $CDCl_3$) of compound **15**

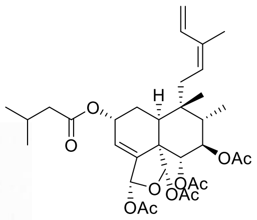

compound 15

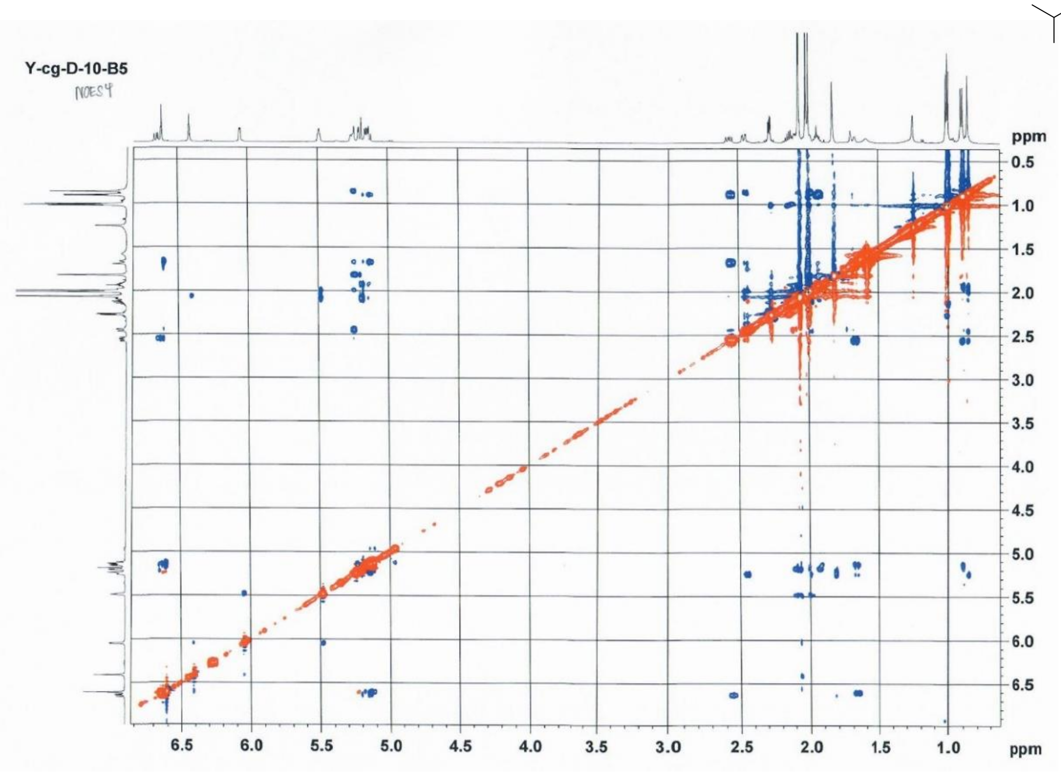

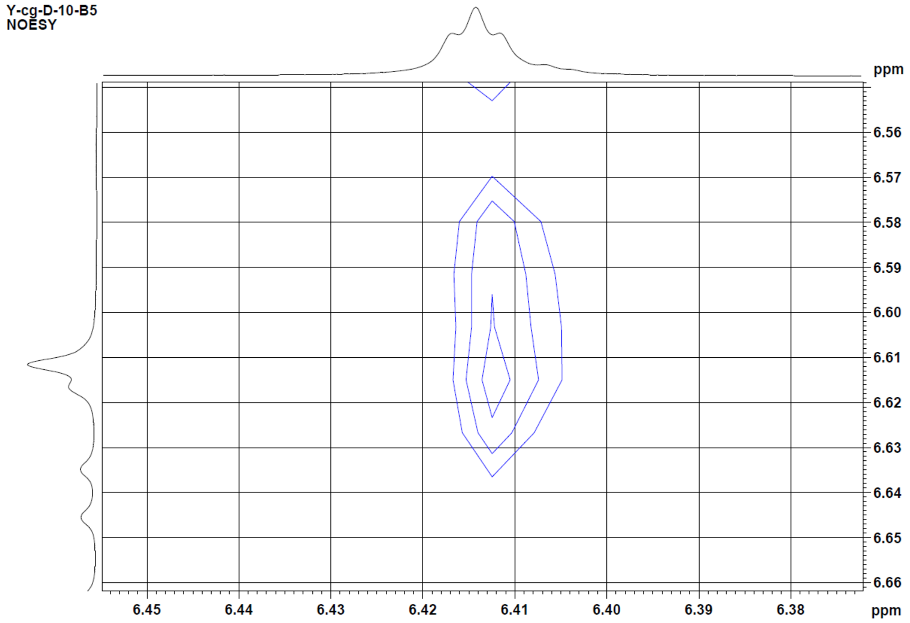

Figure S84. NOESY spectrum (600 MHz, $CDCl_3$) of compound **15**

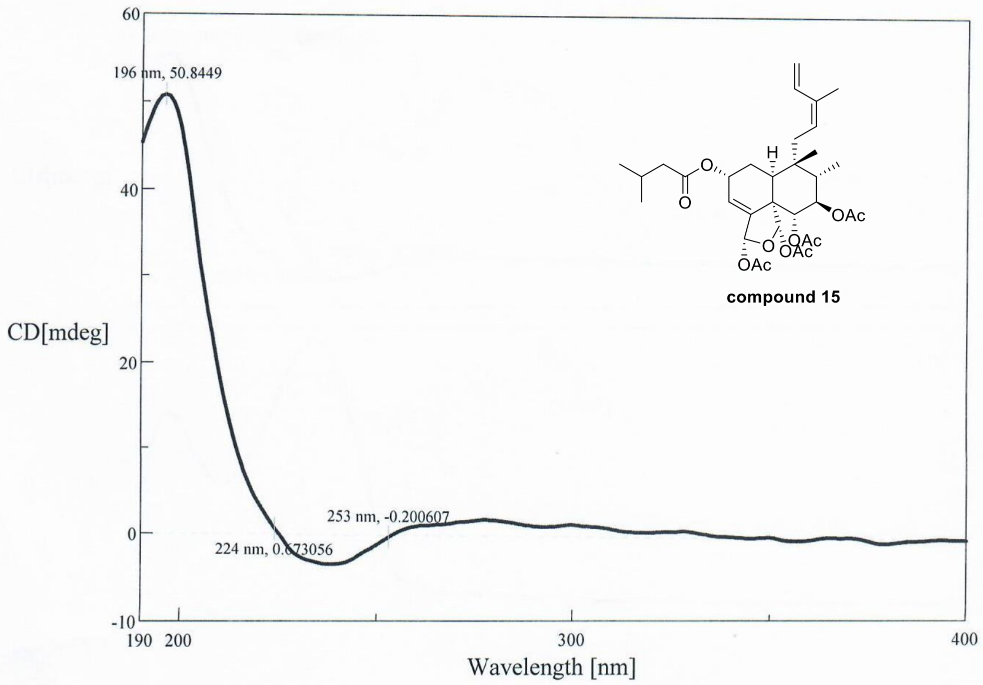

Date	8/5/2022 3:42PM
File name	Y-cg-D-10-B5 original_2
Model	J-815
Serial No.	A024561168
Band width	1 nm
Response	1 sec
Sensitivity	Standard
Measurement range	400 - 190 nm
Data pitch	1nm
Scanning speed	200 nm/min
Accumulation	2
Cell Length	1 cm
Solvent	ACN
Temperature	24.9 C
Sample name	Y-CG-D-10-B5
Operator	Vilailak
Comment	1.6 mg/2ml--200ul/3800ul

Figure S85. CD spectrum of compound **15**

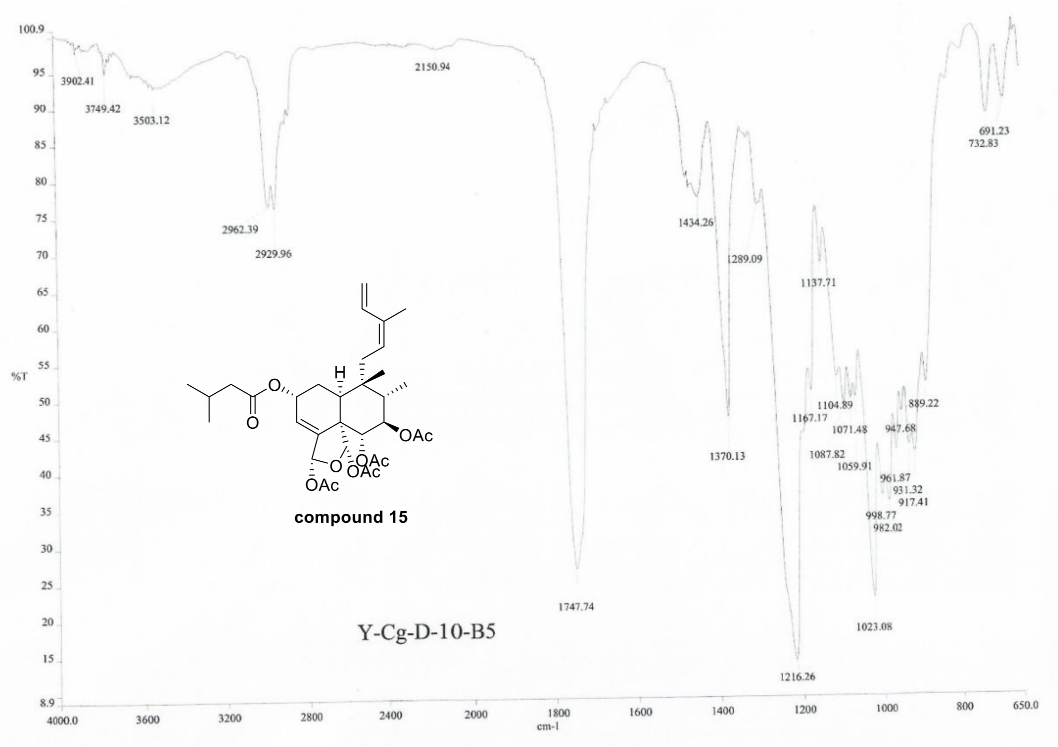

Figure S86. IR spectrum of compound **15**

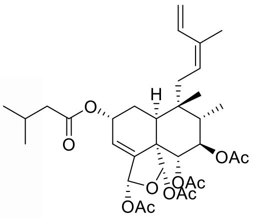

compound 15

Mass Spectrum SmartFormula Report

Analysis Info

Analysis Name D:\Data\CRI\QCR09481 Vilailak Y-CGD-10B5 E+.d
Method Nitirat esi pos low may2021.m
Sample Name ESIpos
Comment

Acquisition Date 7/15/2021 3:32:17 PM
Operator BDAL@DE
Instrument compact 8255754.20094

Acquisition Parameter

Source Type	ESI	Ion Polarity	Positive	Set Nebulizer	0.5 Bar		
Focus	Not active	Set Capillary	3000 V	Set Dry Heater	139 °C		
Scan Begin	100 m/z	Set End Plate Offset	-200 V	Set Dry Gas	4.5 l/min		
Scan End	1000 m/z	Set Charging Voltage	2000 V	Set Divert Valve	Source		
		Set Corona	0 nA	Set APCI Heater	0 °C		

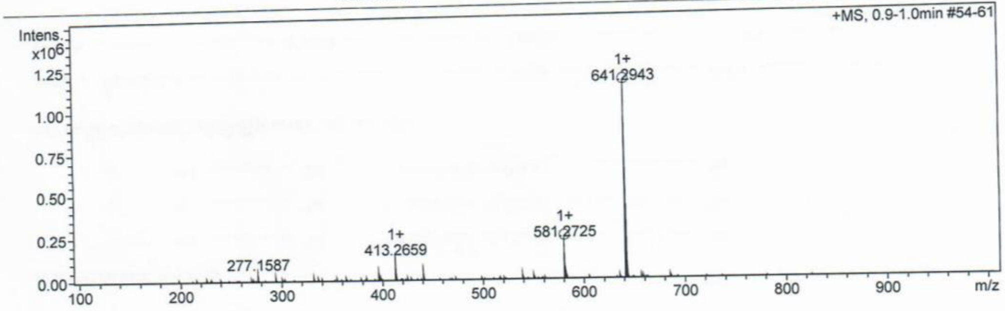

Meas. m/z	#	Ion Formula	m/z	err [ppm]	Mean err [ppm]	rdb	N-Rule	e⁻ Conf	mSigma	Std I	Std Mean m/z	Std I VarNorm	Std m/z Diff	Std Comb Dev
581.272479	1	C31H42NaO9	581.272104	-0.6	-0.2	10.5	ok	even	5.7	7.7	n.a.	n.a.	n.a.	n.a.
	2	C28H41N2O11	581.270487	-3.4	-3.3	9.5	ok	even	7.7	13.6	n.a.	n.a.	n.a.	n.a.
	3	C33H41O9	581.274509	3.5	4.0	13.5	ok	even	16.3	24.0	n.a.	n.a.	n.a.	n.a.
641.294270	1	C33H46NaO11	641.293233	-1.6	-0.3	10.5	ok	even	1.9	3.2	n.a.	n.a.	n.a.	n.a.
	2	C30H45N2O13	641.291616	-4.1	-3.1	9.5	ok	even	10.8	15.6	n.a.	n.a.	n.a.	n.a.
	3	C35H45O11	641.295639	2.1	3.4	13.5	ok	even	13.3	20.5	n.a.	n.a.	n.a.	n.a.

Figure S87. ESITOFMS spectrum of compound **15**

Compound **16**

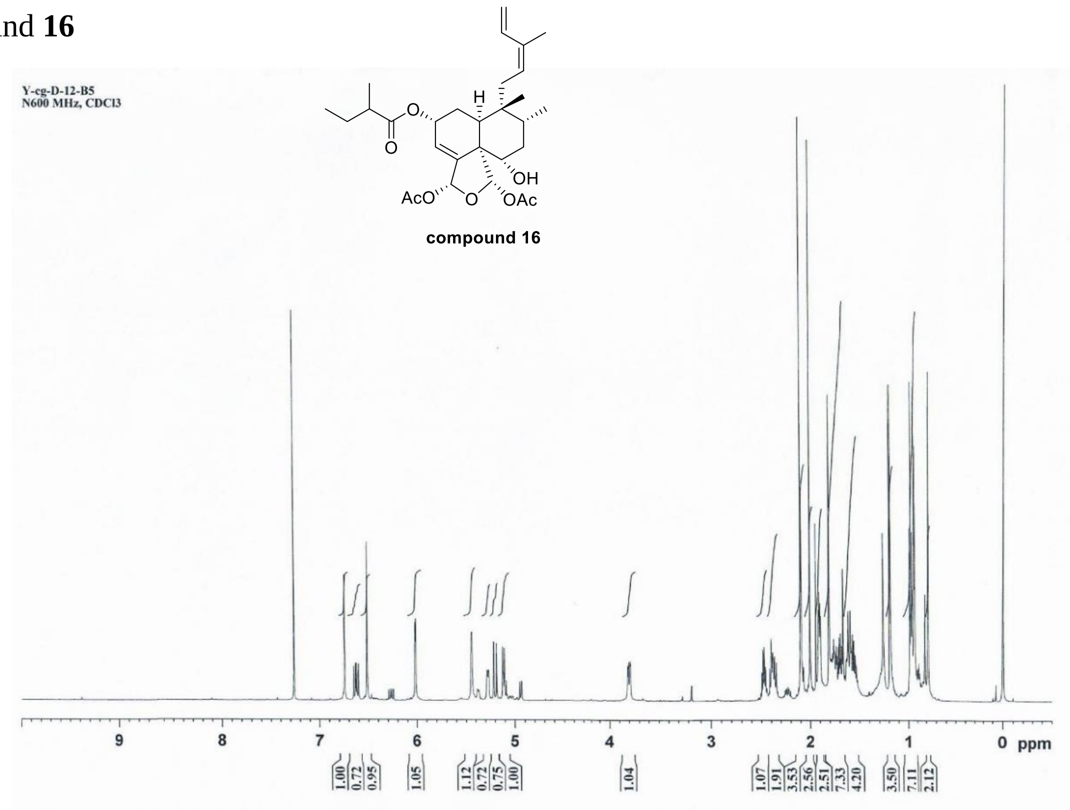

Figure S88. ^{1}H-NMR spectrum (600 MHz, $CDCl_3$) of compound **16**

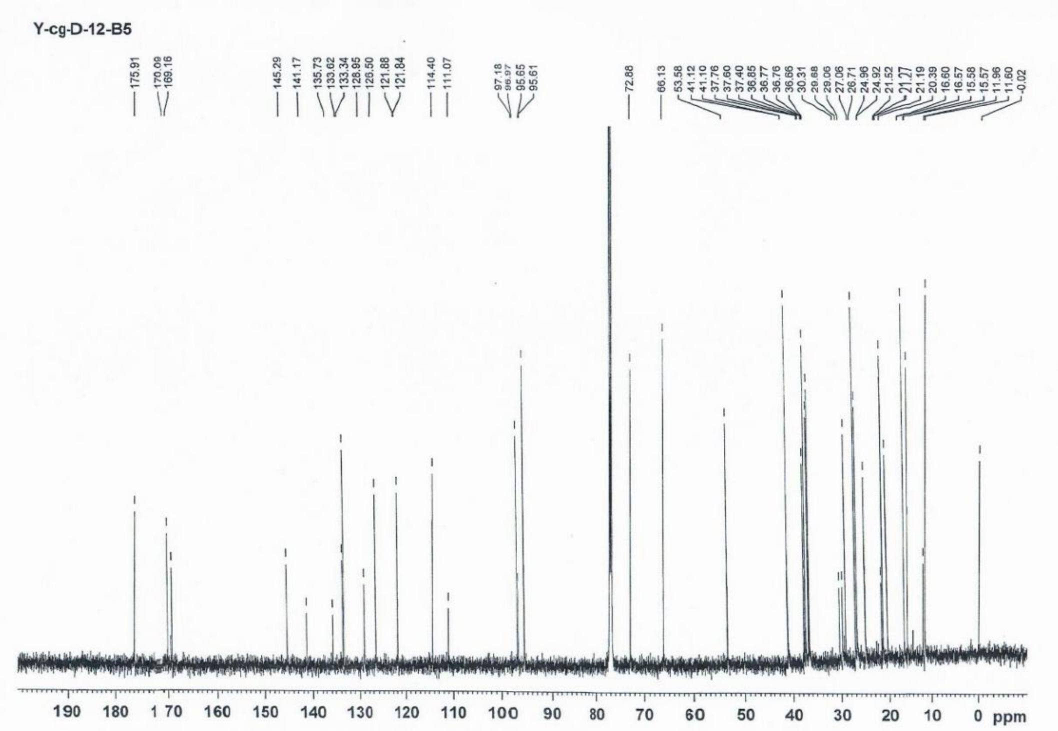

Figure S89. ^{13}C-NMR spectrum (150 MHz, $CDCl_3$) of compound **16**

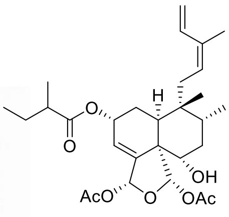

compound **16**

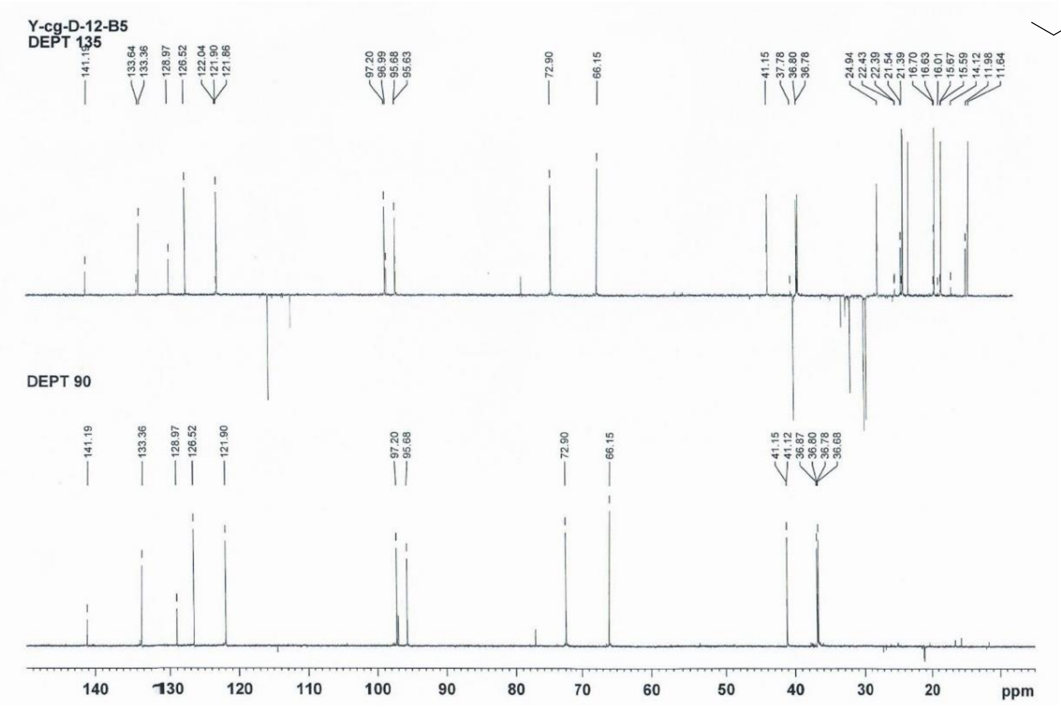

Figure S90. DEPT spectrum (600 MHz, $CDCl_3$) of compound **16**

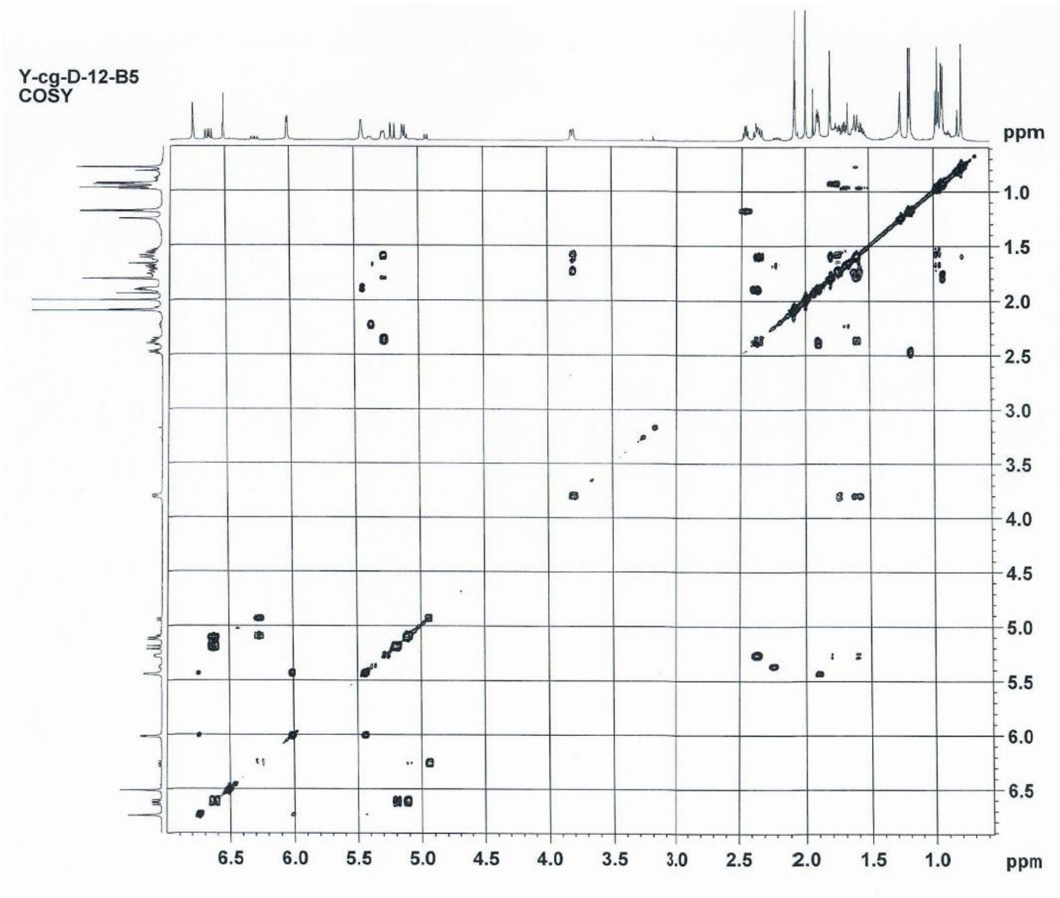

Figure S91. ^{1}H-^{1}H COSY spectrum (600 MHz, $CDCl_3$) of compound **16**

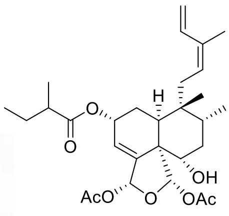

compound 16

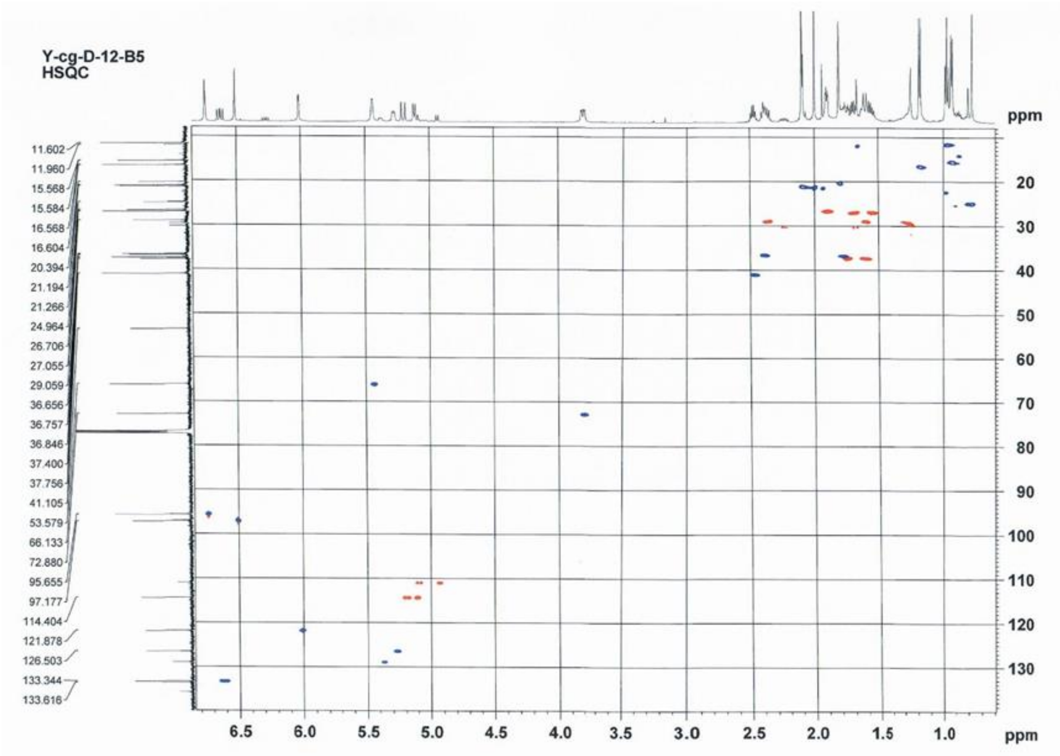

Figure S92. HSQC spectrum (600 MHz, $CDCl_3$) of compound **16**

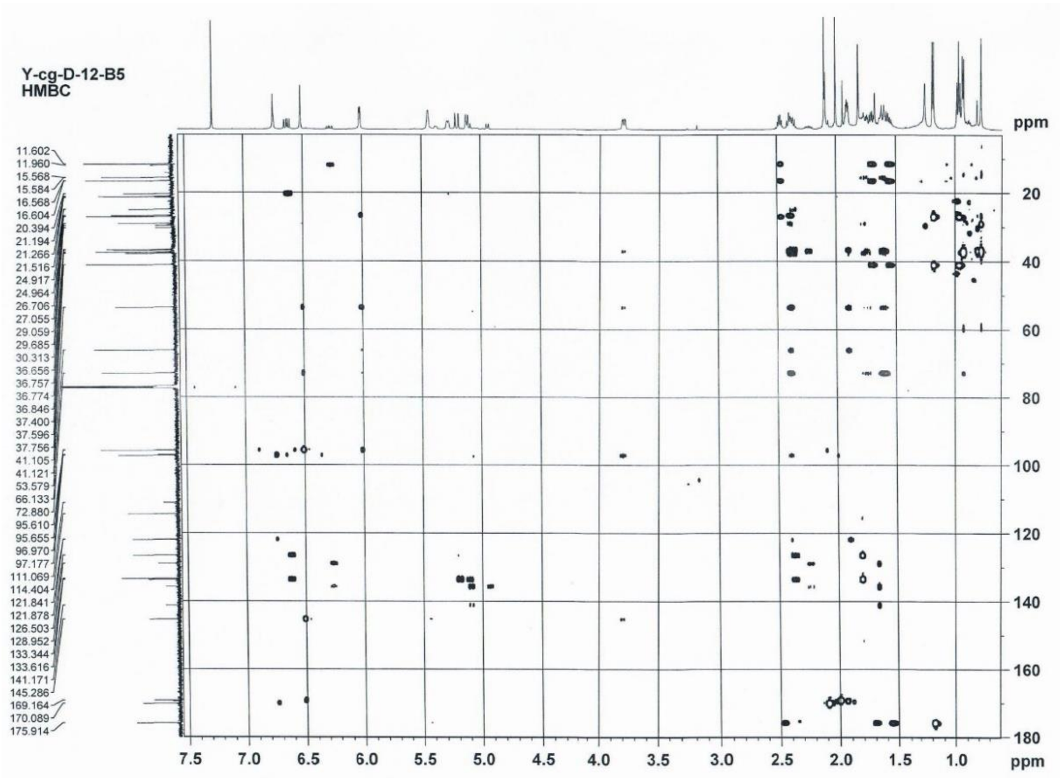

Figure S93. HMBC spectrum (600 MHz, $CDCl_3$) of compound **16**

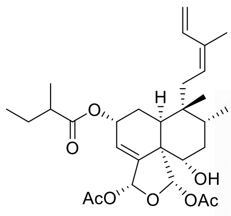

compound 16

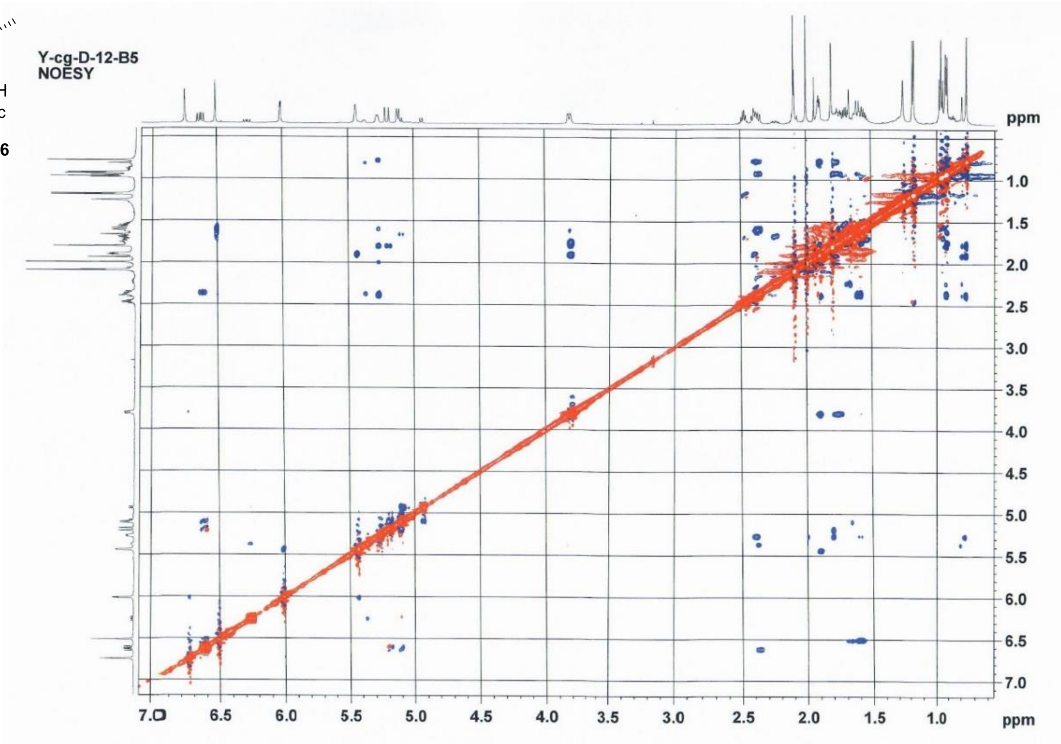

Figure S94. NOESY spectrum (600 MHz, $CDCl_3$) of compound **16**

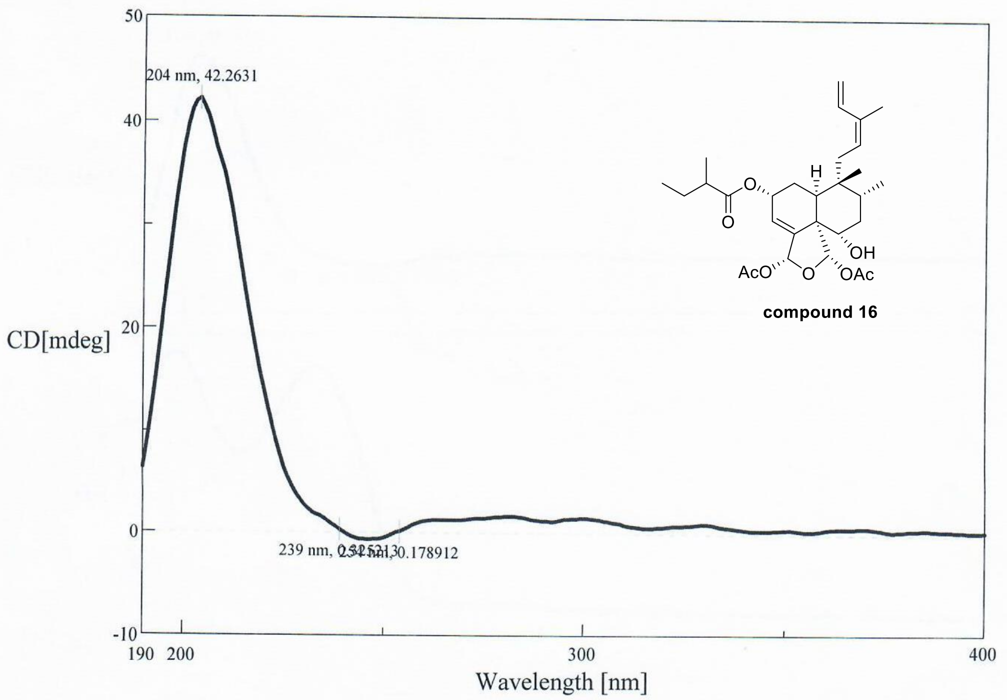

Date	8/5/2022 4:34PM
File name	Y-cg-D-12-B5 original_4
Model	J-815
Serial No.	A024561168
Band width	1 nm
Response	1 sec
Sensitivity	Standard
Measurement range	400 - 190 nm
Data pitch	1nm
Scanning speed	200 nm/min
Accumulation	2
Cell Length	1 cm
Solvent	ACN
Temperature	25 C
Sample name	Y-CG-D-12-B5
Operator	Vilailak
Comment	1.1 mg/2ml--250ul/3750ul

Figure S95. CD spectrum of compound **16**

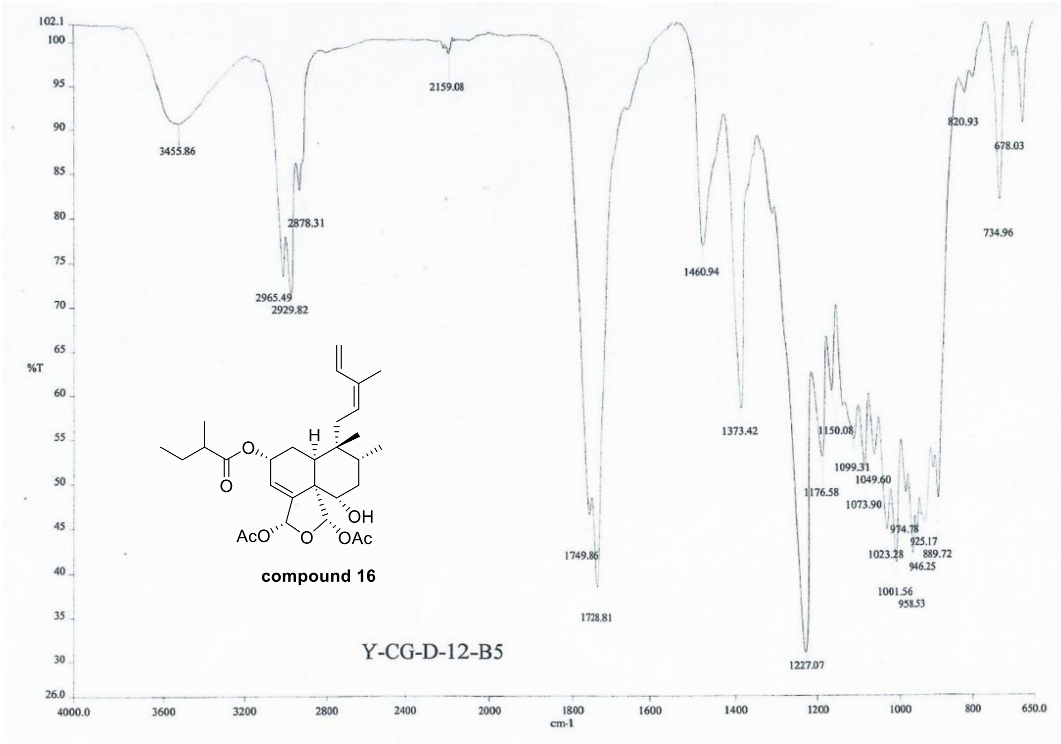

Figure S96. IR spectrum of compound **16**

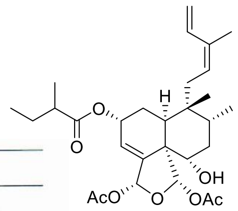

compound 16

Mass Spectrum SmartFormula Report

Analysis Info

Analysis Name D:\Data\CRI\QCR11647 Vilailak Y-cg-D12-B5 E+.d
Method Nitirat esi pos low 2022-1.m
Sample Name ESIpos
Comment

Acquisition Date 6/2/2022 12:29:33 PM
Operator BDAL@DE
Instrument compact 8255754.20094

Acquisition Parameter

Source Type	ESI	Ion Polarity	Positive	Set Nebulizer	0.5 Bar
Focus	Not active	Set Capillary	3000 V	Set Dry Heater	150 °C
Scan Begin	100 m/z	Set End Plate Offset	-500 V	Set Dry Gas	4.5 l/min
Scan End	700 m/z	Set Charging Voltage	2000 V	Set Divert Valve	Source
		Set Corona	0 nA	Set APCI Heater	0 °C

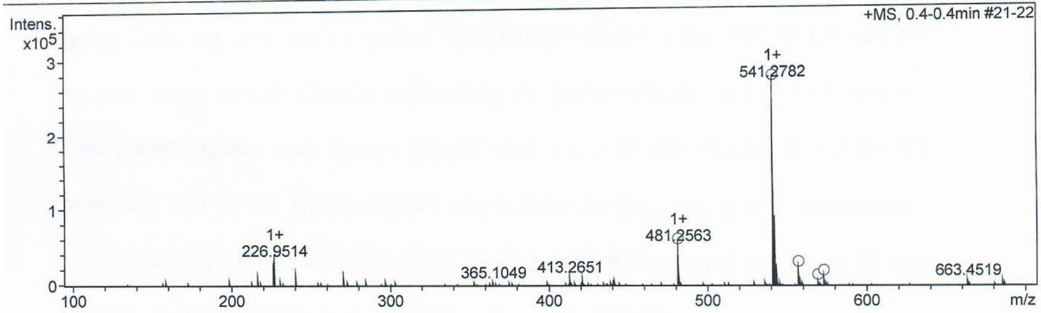

Meas. m/z	#	Ion Formula	m/z	err [ppm]	Mean err [ppm]	rdb	N-Rule	e⁻ Conf	mSigma	Std I	Std Mean m/z	Std I VarNorm	Std m/z Diff	Std Comb Dev
481.256256	1	C24H37N2O8	481.254443	-3.8	1028.2	7.5	ok	even	9.0	14.5	n.a.	n.a.	n.a.	n.a.
	2	C27H38NaO6	481.256060	-0.4	1025.3	8.5	ok	even	9.2	14.0	n.a.	n.a.	n.a.	n.a.
	3	C29H37O6	481.258465	4.6	1024.6	11.5	ok	even	18.2	30.0	n.a.	n.a.	n.a.	n.a.
541.278239	1	C29H42NaO8	541.277189	-1.9	1544.9	8.5	ok	even	33.8	63.4	n.a.	n.a.	n.a.	n.a.
	2	C26H41N2O10	541.275572	-4.9	1549.9	7.5	ok	even	35.6	60.3	n.a.	n.a.	n.a.	n.a.
	3	C31H41O8	541.279595	2.5	1544.0	11.5	ok	even	37.9	74.3	n.a.	n.a.	n.a.	n.a.
557.265022	1	C31H43KNaO5	557.263977	-1.9	883.6	9.5	ok	even	19.6	26.6	n.a.	n.a.	n.a.	n.a.
	2	C33H42KO5	557.266383	2.4	884.1	12.5	ok	even	19.8	30.6	n.a.	n.a.	n.a.	n.a.
	3	C28H42KN2O7	557.262360	-4.8	885.0	8.5	ok	even	25.8	33.4	n.a.	n.a.	n.a.	n.a.
	4	C31H38N2NaO6	557.262208	-5.0	1490.6	13.5	ok	even	36.2	66.9	n.a.	n.a.	n.a.	n.a.
	5	C33H37N2O6	557.264613	-0.7	1489.9	16.5	ok	even	41.0	78.0	n.a.	n.a.	n.a.	n.a.
	6	C36H38NaO4	557.266230	2.2	1485.4	17.5	ok	even	47.5	89.5	n.a.	n.a.	n.a.	n.a.
	7	C24H42N2NaO11	557.268081	5.5	1516.8	4.5	ok	even	48.9	71.6	n.a.	n.a.	n.a.	n.a.
569.379865	1	C33H54NaO6	569.381260	2.5	1461.1	6.5	ok	even	44.3	69.8	n.a.	n.a.	n.a.	n.a.
	2	C35H53O6	569.383666	6.7	1460.5	9.5	ok	even	44.3	74.9	n.a.	n.a.	n.a.	n.a.
	3	C30H53N2O8	569.379643	-0.4	1465.6	5.5	ok	even	49.6	72.6	n.a.	n.a.	n.a.	n.a.
	4	C28H54N2NaO8	569.377237	-4.6	1466.5	2.5	ok	even	55.1	77.2	n.a.	n.a.	n.a.	n.a.
	5	C42H49O	569.377793	-3.6	1436.9	18.5	ok	even	59.6	103.5	n.a.	n.a.	n.a.	n.a.
573.265400	1	C31H41O10	573.269424	7.0	855.5	11.5	ok	even	79.3	116.3	n.a.	n.a.	n.a.	n.a.
	2	C29H42NaO10	573.267018	2.8	856.1	8.5	ok	even	82.7	125.7	n.a.	n.a.	n.a.	n.a.
	3	C26H41N2O12	573.265401	0.0	858.4	7.5	ok	even	85.9	134.8	n.a.	n.a.	n.a.	n.a.
	4	C24H42N2NaO12	573.262995	-4.2	858.9	4.5	ok	even	91.6	148.0	n.a.	n.a.	n.a.	n.a.

Figure S97. ESITOFMS spectrum of compound **16**

Compound **17**

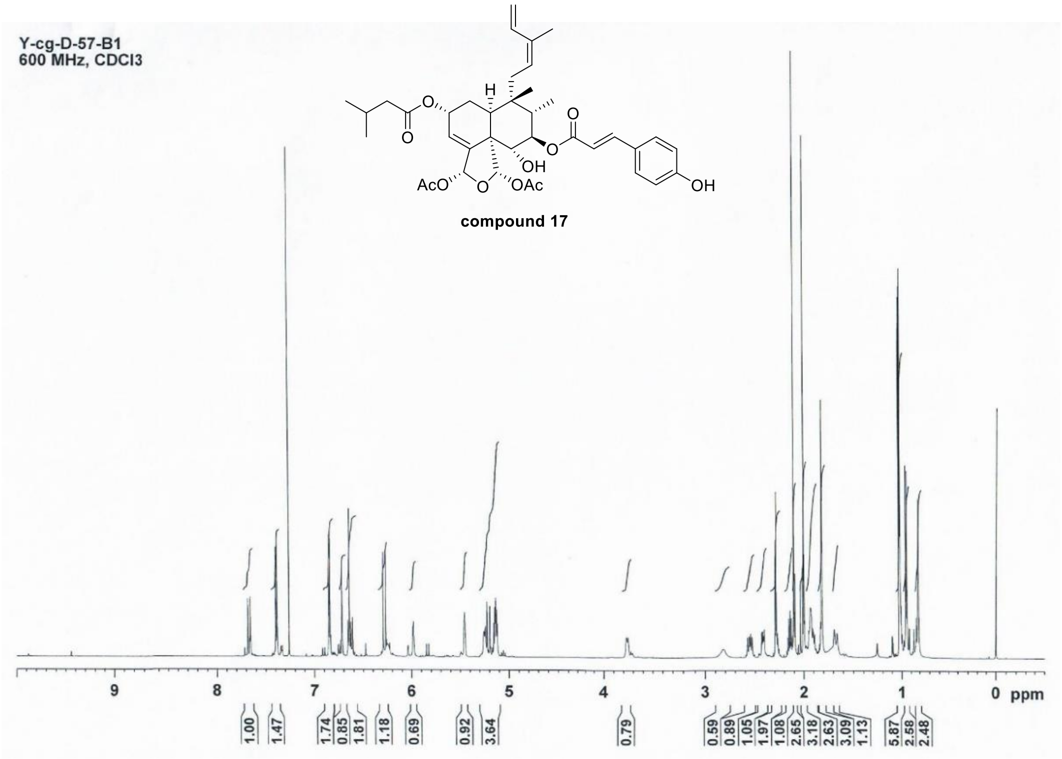

Figure S98. ^{1}H-NMR spectrum (600 MHz, $CDCl_3$) of compound **17**

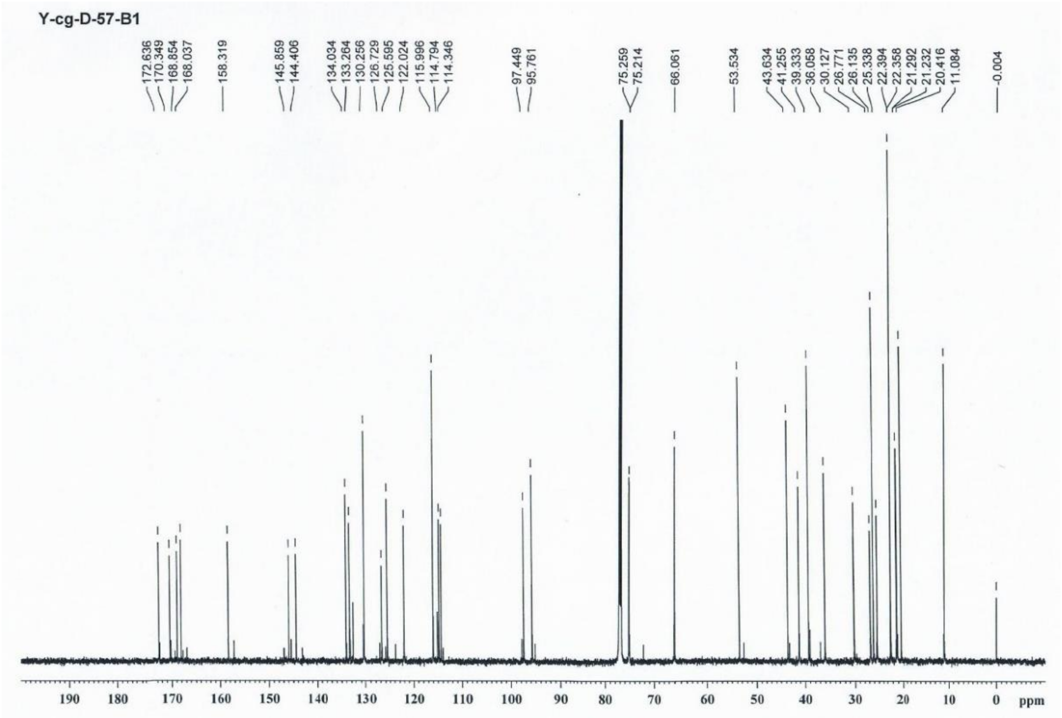

Figure S99. ^{13}C-NMR spectrum (150 MHz, $CDCl_3$) of compound **17**

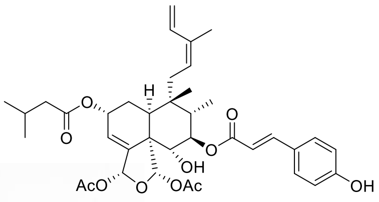

compound 17

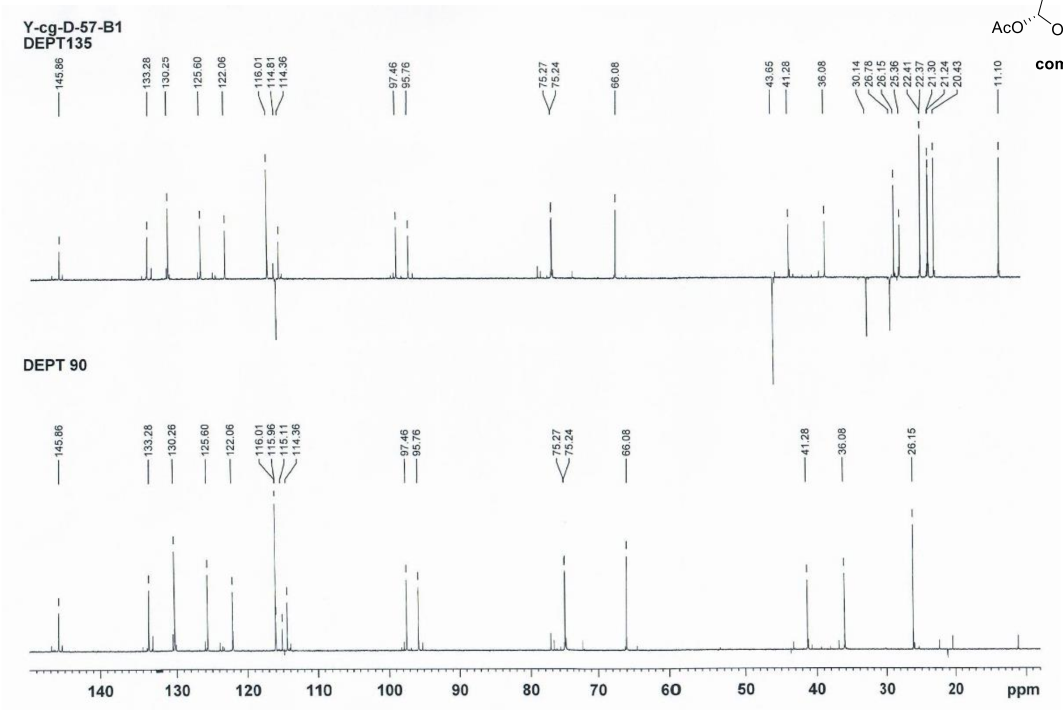

Figure S100. DEPT spectrum (600 MHz, $CDCl_3$) of compound **17**

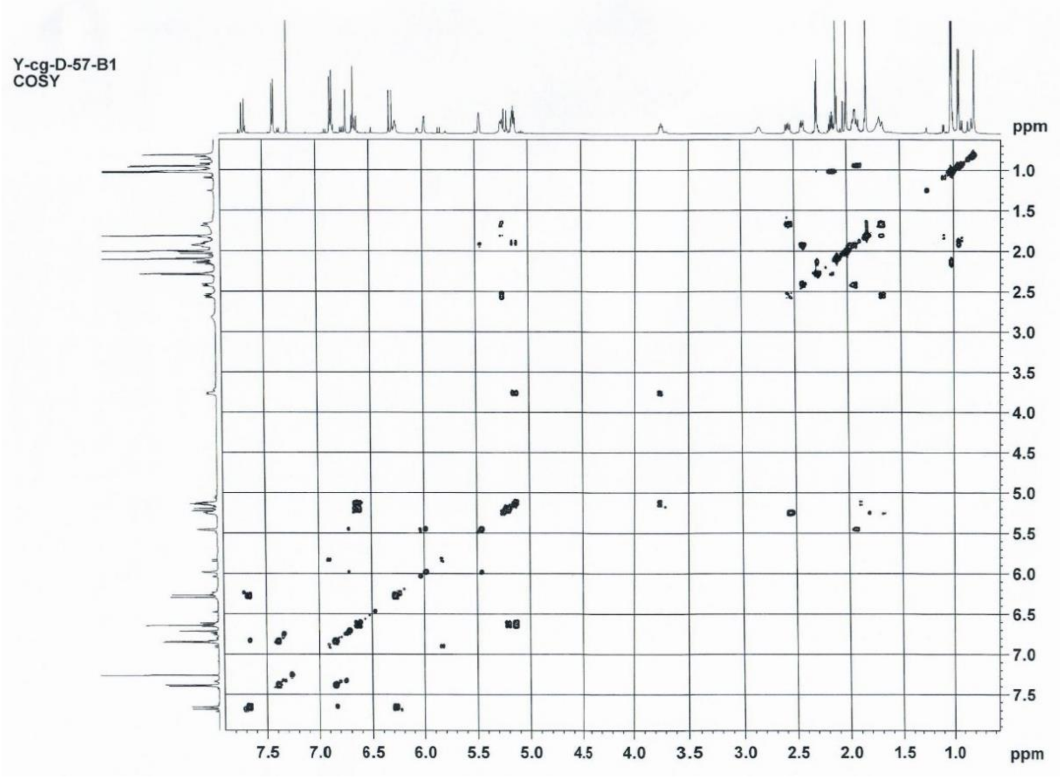

Figure S101. ^{1}H-^{1}H COSY spectrum (600 MHz, $CDCl_3$) of compound **17**

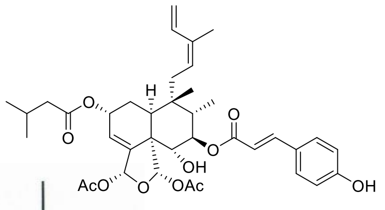

compound 17

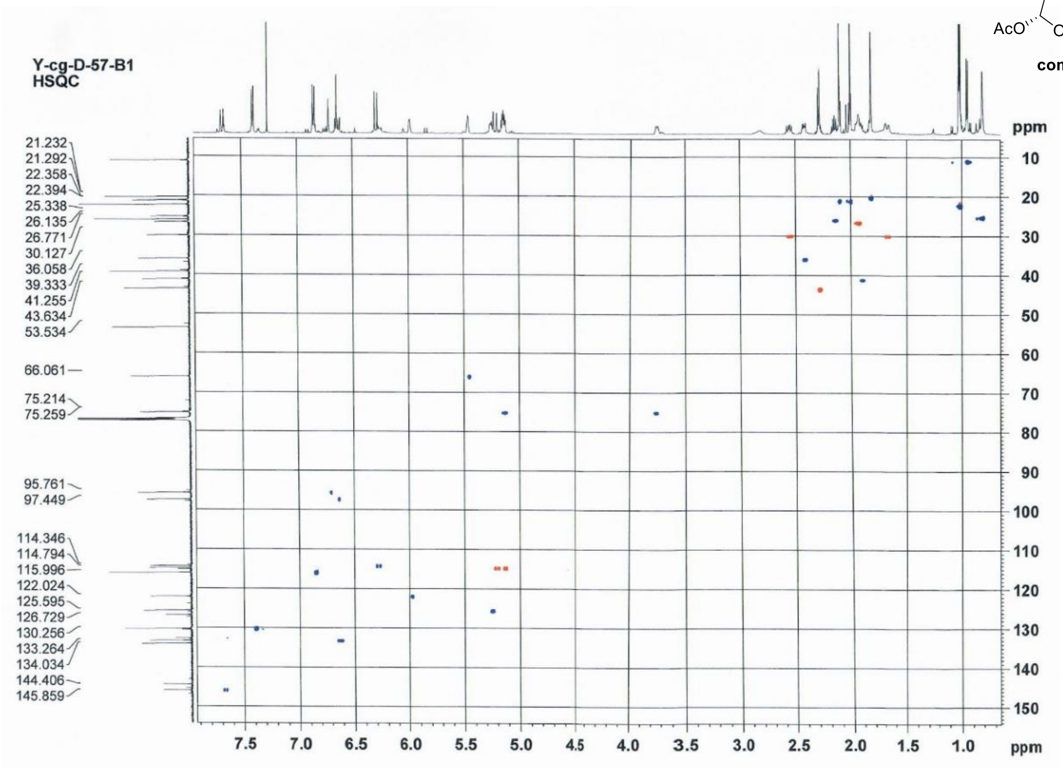

Figure S102. HSQC spectrum (600 MHz, $CDCl_3$) of compound **17**

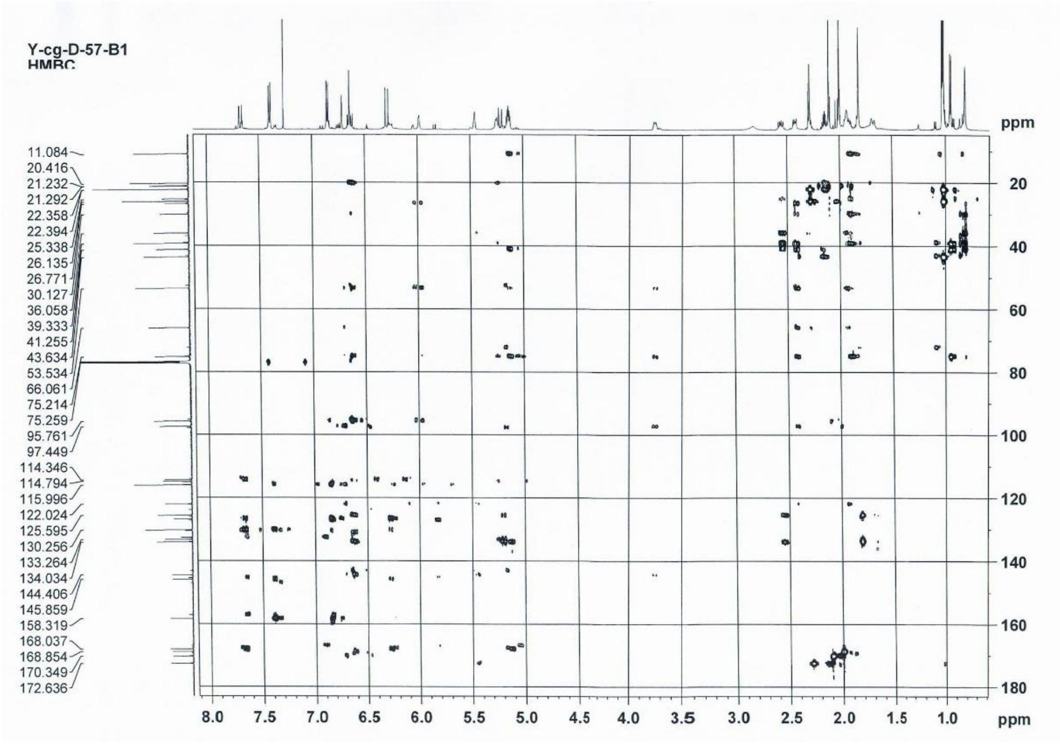

Figure S103. HMBC spectrum (600 MHz, $CDCl_3$) of compound **17**

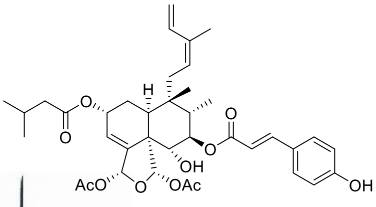

compound **17**

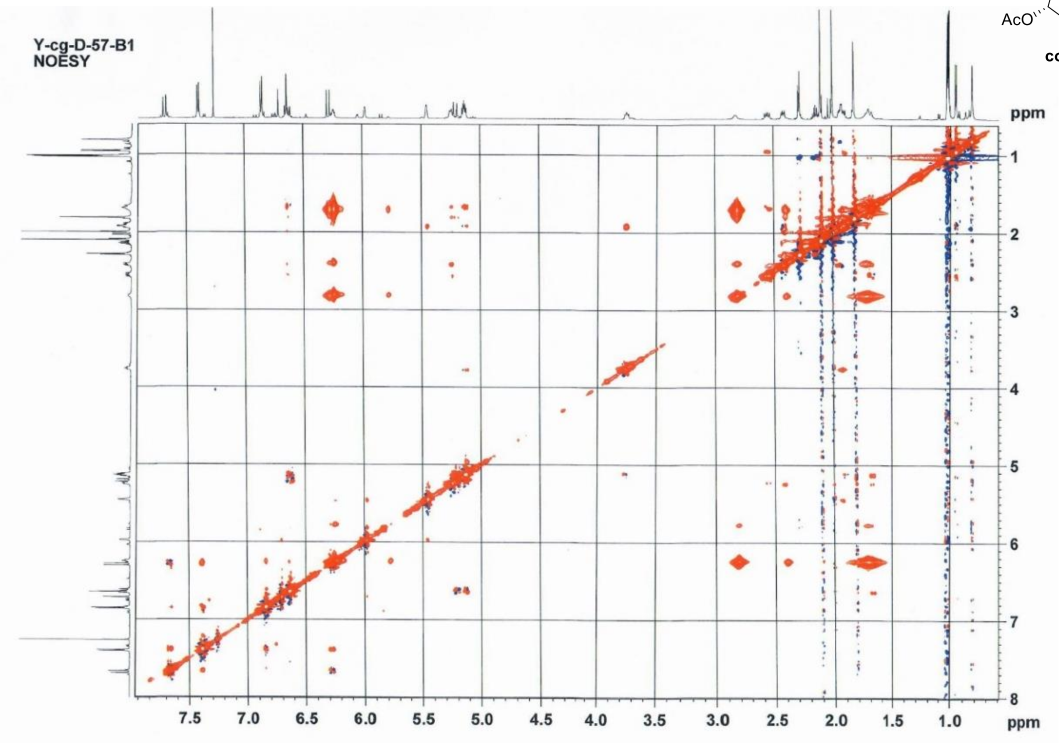

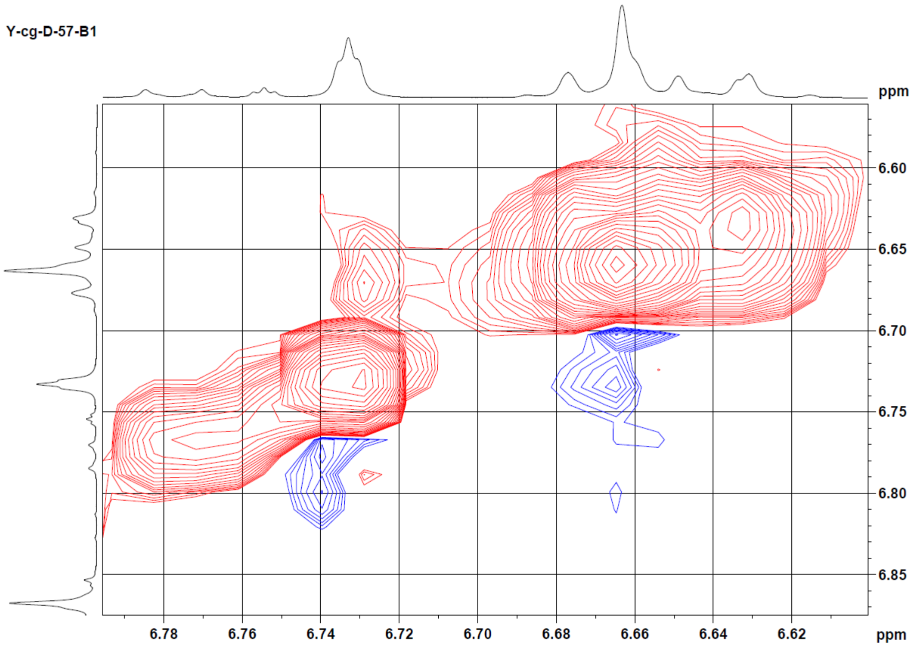

Figure S104. NOESY spectrum (600 MHz, $CDCl_3$) of compound **17**

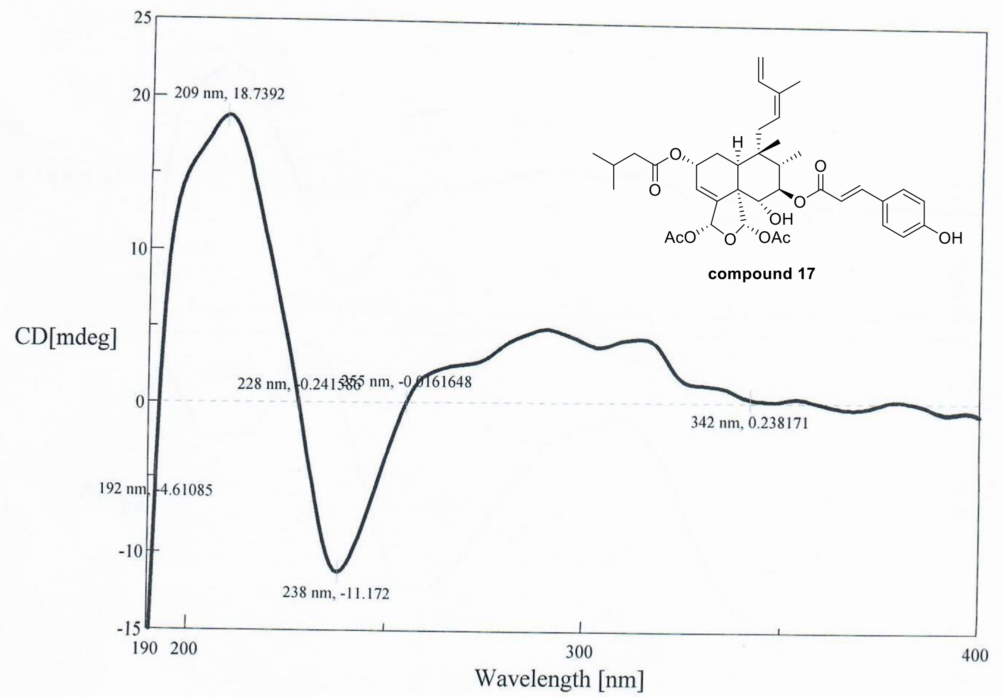

Date	11/29/2022 6:39PM
File name	Y-cg-D-57-B1 80u (process 1)
Model	J-815
Serial No.	A024561168
Band width	1 nm
Response	1 sec
Sensitivity	Standard
Measurement range	400 - 190 nm
Data pitch	1nm
Scanning speed	200 nm/min
Accumulation	2
Temperature	24.9 C
Sample name	Y-cg-D-57-B1 80u
Operator	Vilailak
Comment	4.2 mg in 2 ml ACN 80u/3920u

Figure S105. CD spectrum of compound **17**

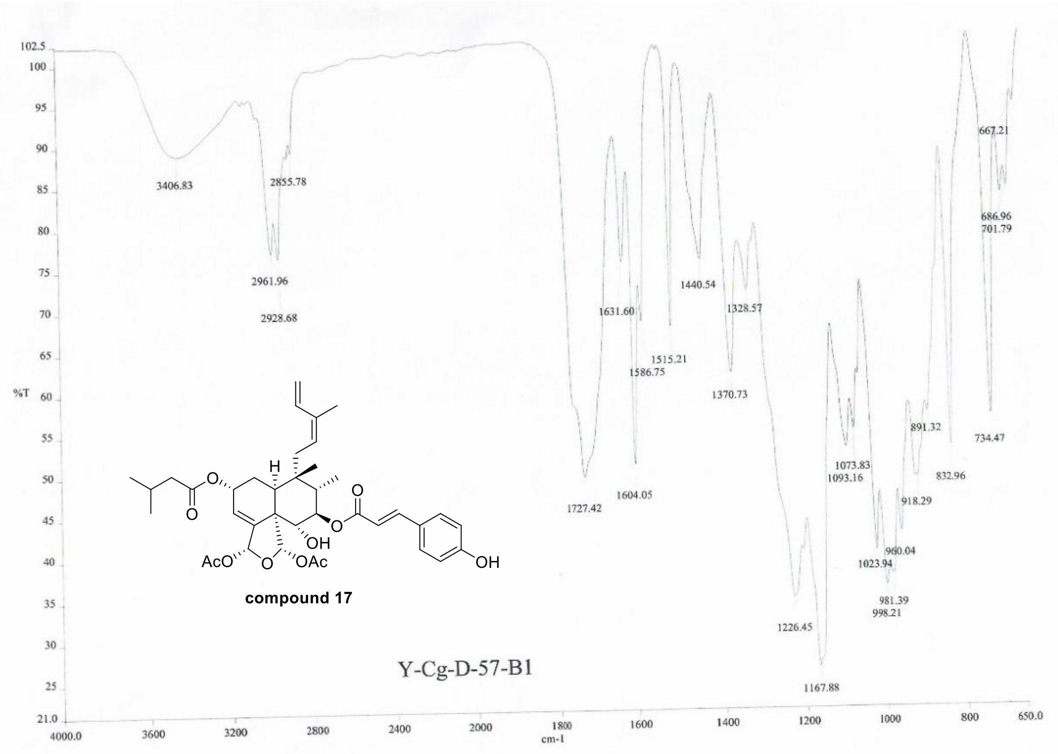

Figure S106. IR spectrum of compound **17**

Mass Spectrum SmartFormula Report

Analysis Info

Analysis Name	D:\Data\CRI\QCR13046 Vilailak Y-cg-D-57B1 E+.d	Acquisition Date	11/10/2022 1:35:41 PM
Method	Nitirat esi pos low may2021-2.m	Operator	BDAL@DE
Sample Name	ESIpos	Instrument	compact 8255754.20094
Comment			

Acquisition Parameter

Source Type	ESI	Ion Polarity	Positive	Set Nebulizer	0.6 Bar
Focus	Not active	Set Capillary	3000 V	Set Dry Heater	120 °C
Scan Begin	100 m/z	Set End Plate Offset	-500 V	Set Dry Gas	5.3 l/min
Scan End	800 m/z	Set Charging Voltage	2000 V	Set Divert Valve	Source
		Set Corona	0 nA	Set APCI Heater	0 °C

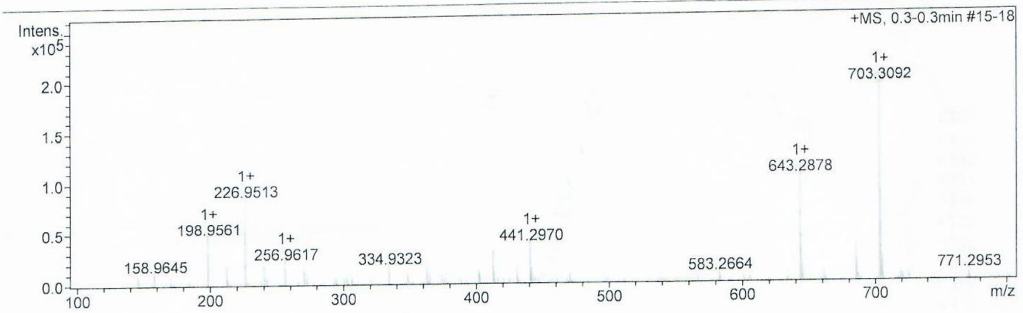

Meas. m/z	#	Ion Formula	m/z	err [ppm]	Mean err [ppm]	rdb	N-Rule	e⁻ Conf	mSigma	Std I	Std Mean m/z	Std I VarNorm	Std m/z Diff	Std Comb Dev
643.287800	1	C36H44NaO9	643.287754	-0.1	0.9	14.5	ok	even	1.1	1.7	n.a.	n.a.	n.a.	n.a.
703.309214	1	C38H48NaO11	703.308883	-0.5	0.2	14.5	ok	even	5.0	6.5	n.a.	n.a.	n.a.	n.a.

Figure S107. ESITOFMS spectrum of compound **17**

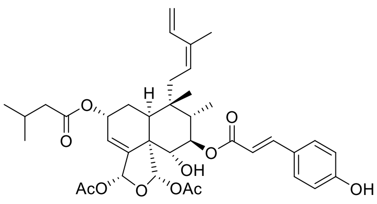

compound 17

Compound **18**

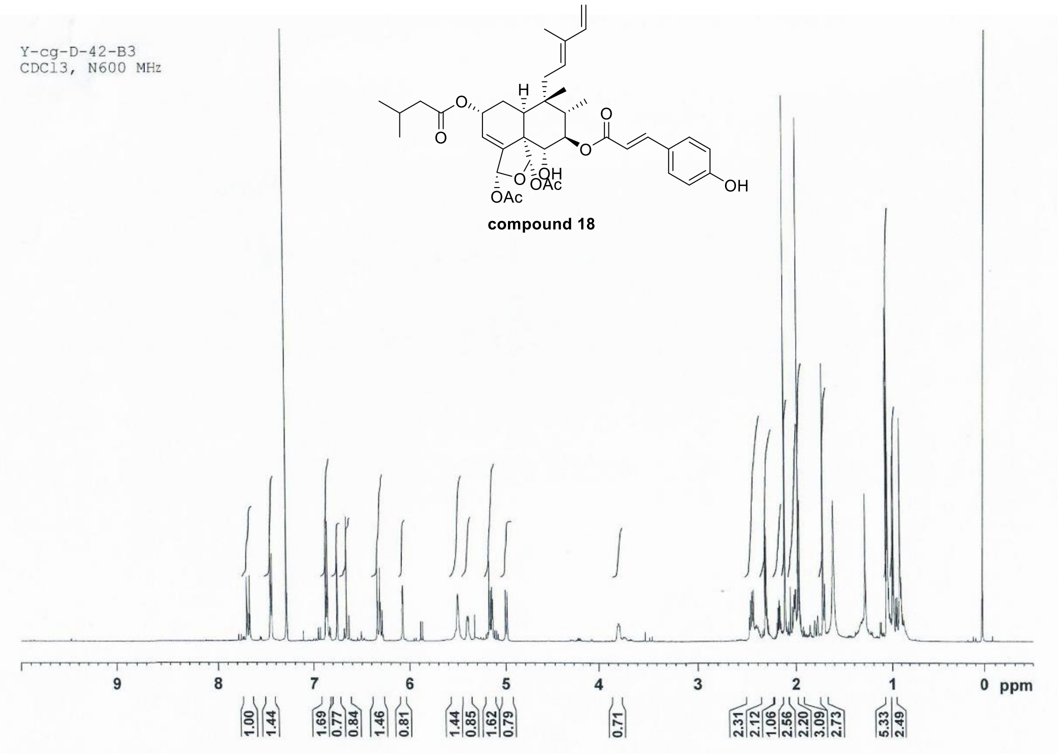

Figure S108. ^{1}H-NMR spectrum (600 MHz, $CDCl_3$) of compound **18**

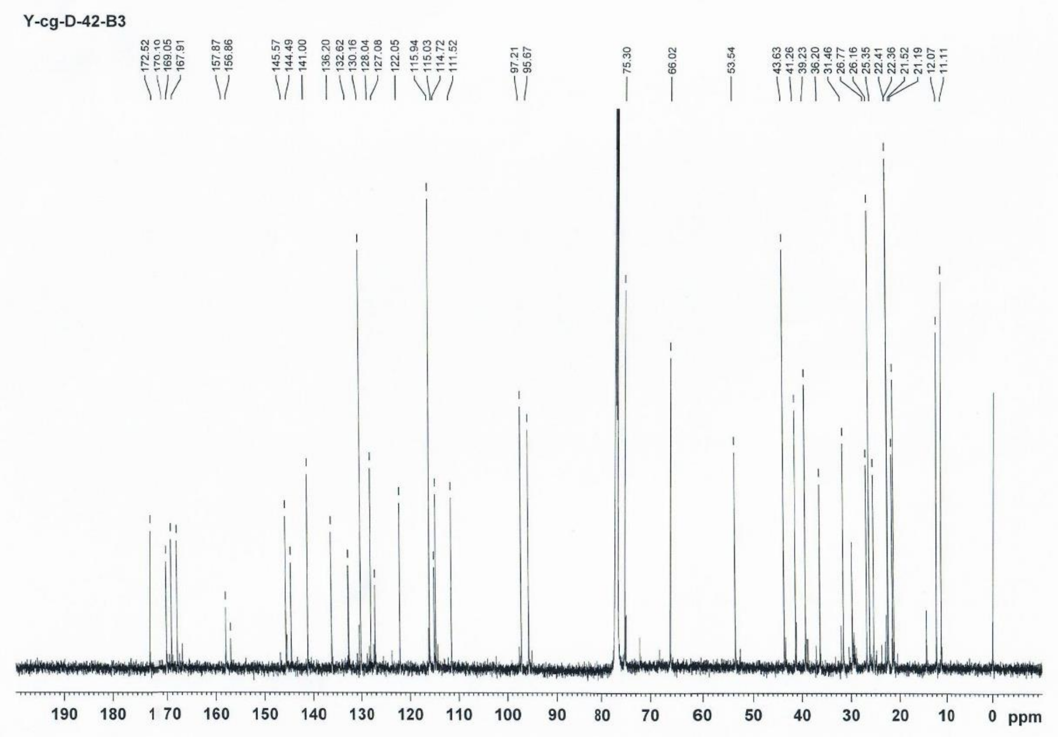

Figure S109. ^{13}C-NMR spectrum (150 MHz, $CDCl_3$) of compound **18**

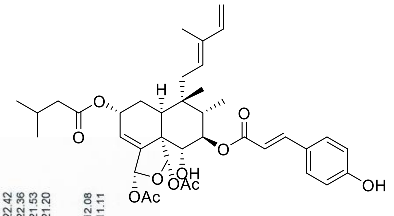

compound 18

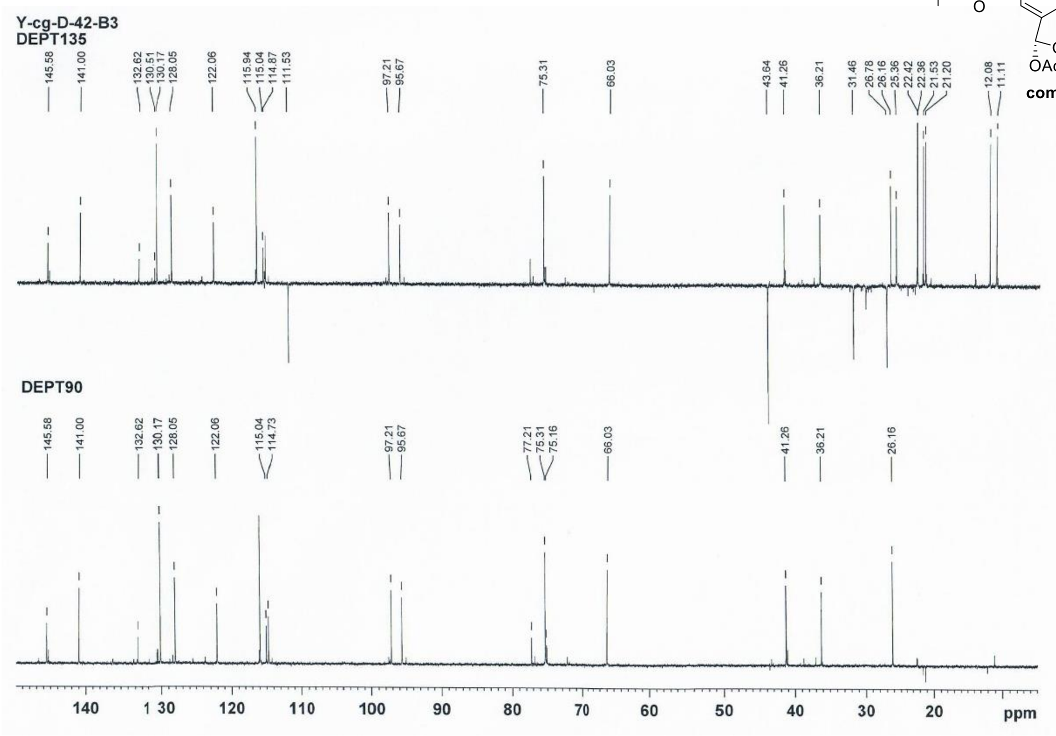

Figure S110. DEPT spectrum (600 MHz, $CDCl_3$) of compound **18**

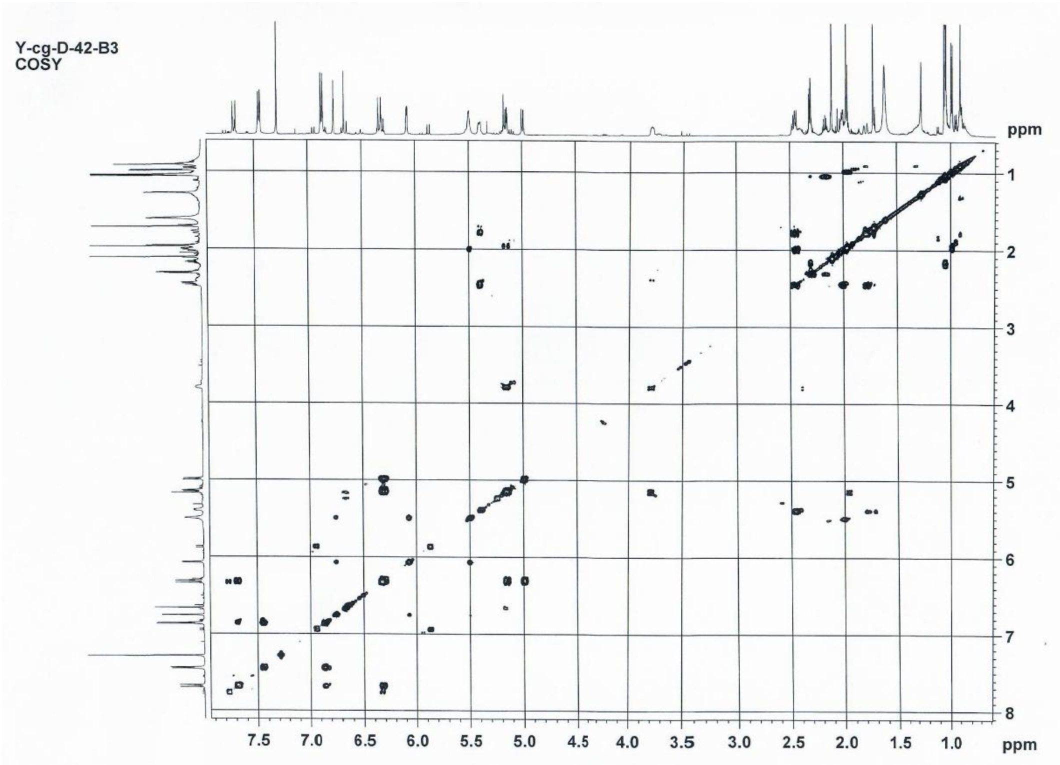

Figure S111. ^1H-^1H COSY spectrum (600 MHz, $CDCl_3$) of compound **18**

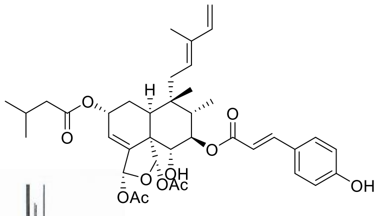

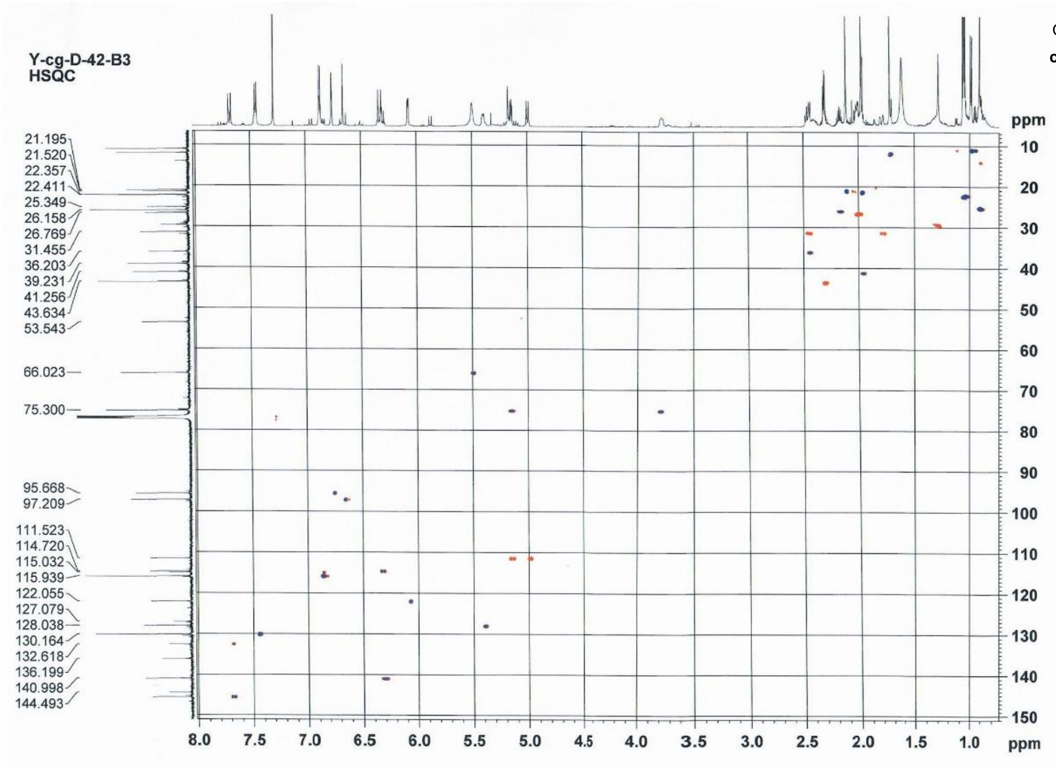

Figure S112. HSQC spectrum (600 MHz, $CDCl_3$) of compound **18**

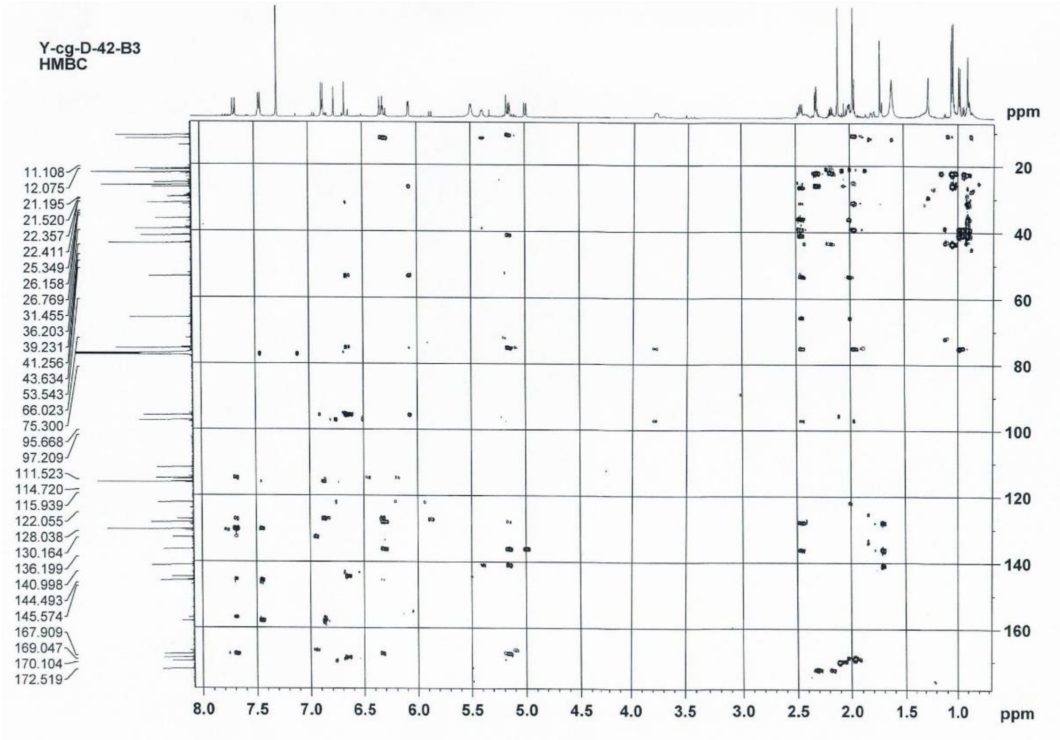

Figure S113. HMBC spectrum (600 MHz, $CDCl_3$) of compound **18**

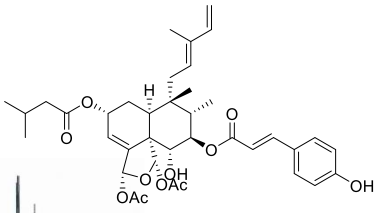

compound 18

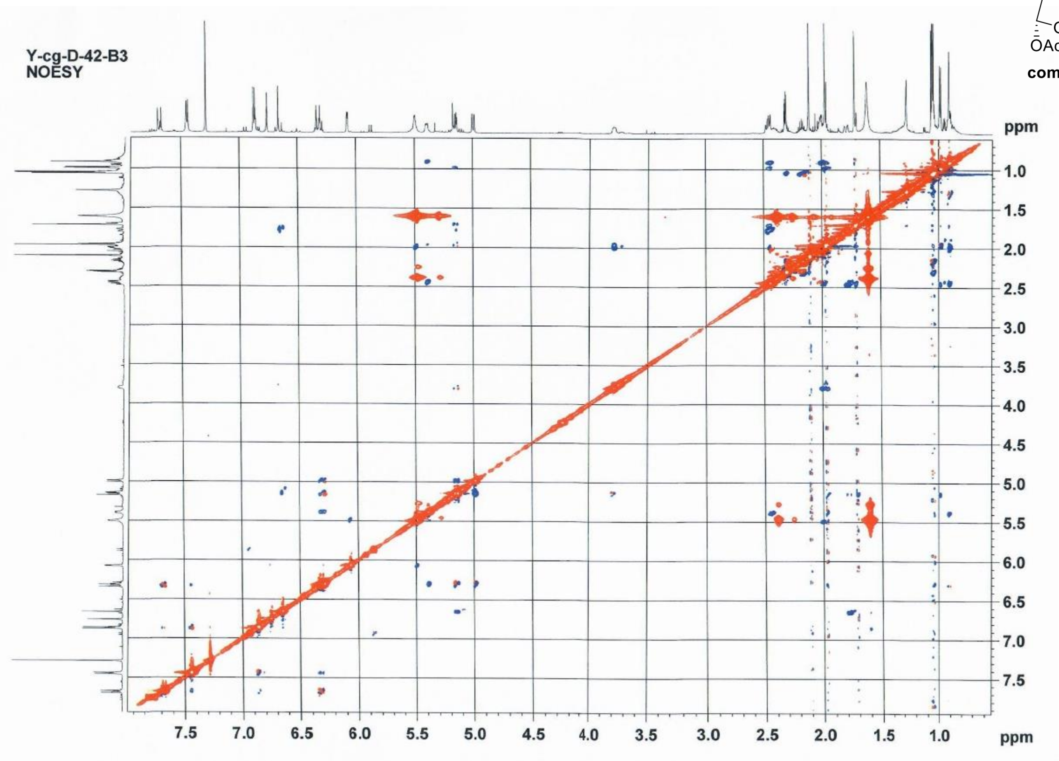

Figure S114. NOESY spectrum (600 MHz, $CDCl_3$) of compound **18**

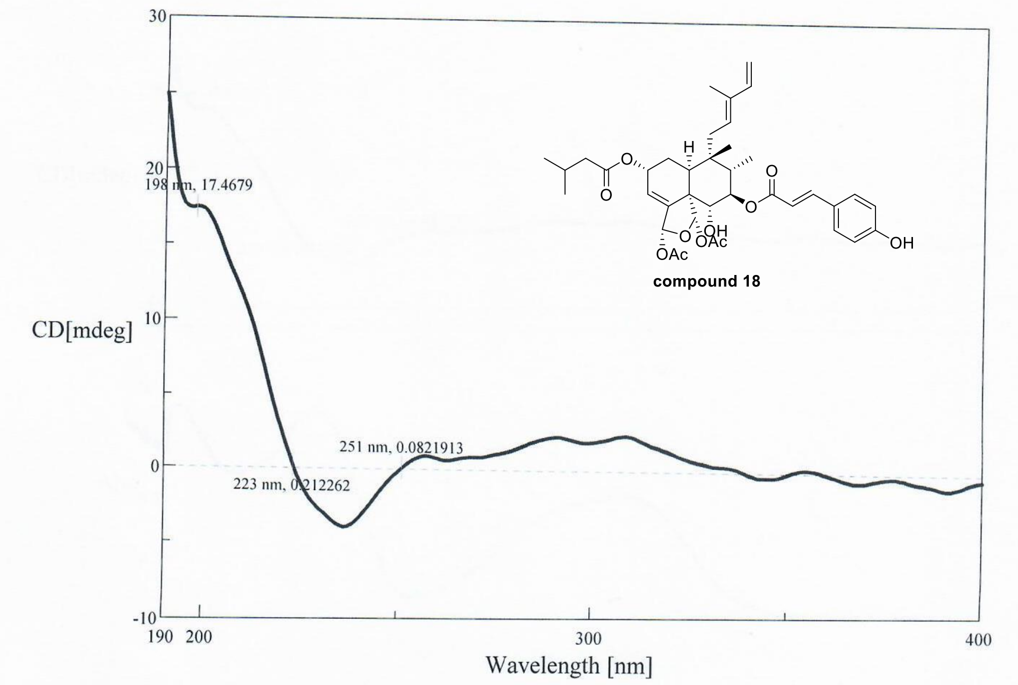

Date	11/29/2022 6:49PM
File name	Y-cg-D-42-B3 200u (process 1)
Model	J-815
Serial No.	A024561168
Band width	1 nm
Response	1 sec
Sensitivity	Standard
Measurement range	400 - 190 nm
Data pitch	1nm
Scanning speed	200 nm/min
Accumulation	2
Temperature	25 C
Sample name	Y-cg-D-42-B3 200u
Operator	Vilailak
Comment	3.8 mg in 3 ml ACN 200u/3800u

Figure S115. CD spectrum of compound **18**

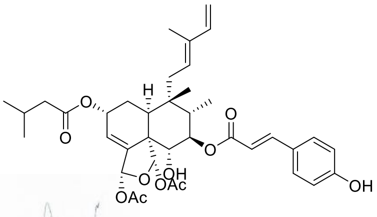

compound 18

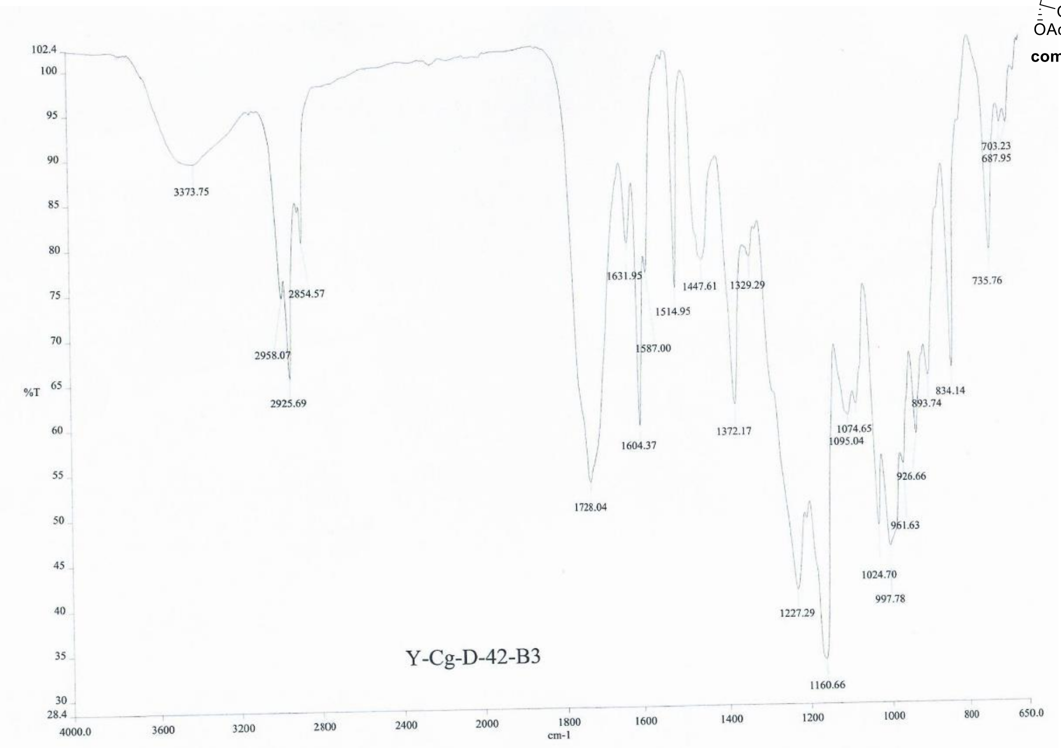

Figure S116. IR spectrum of compound **18**

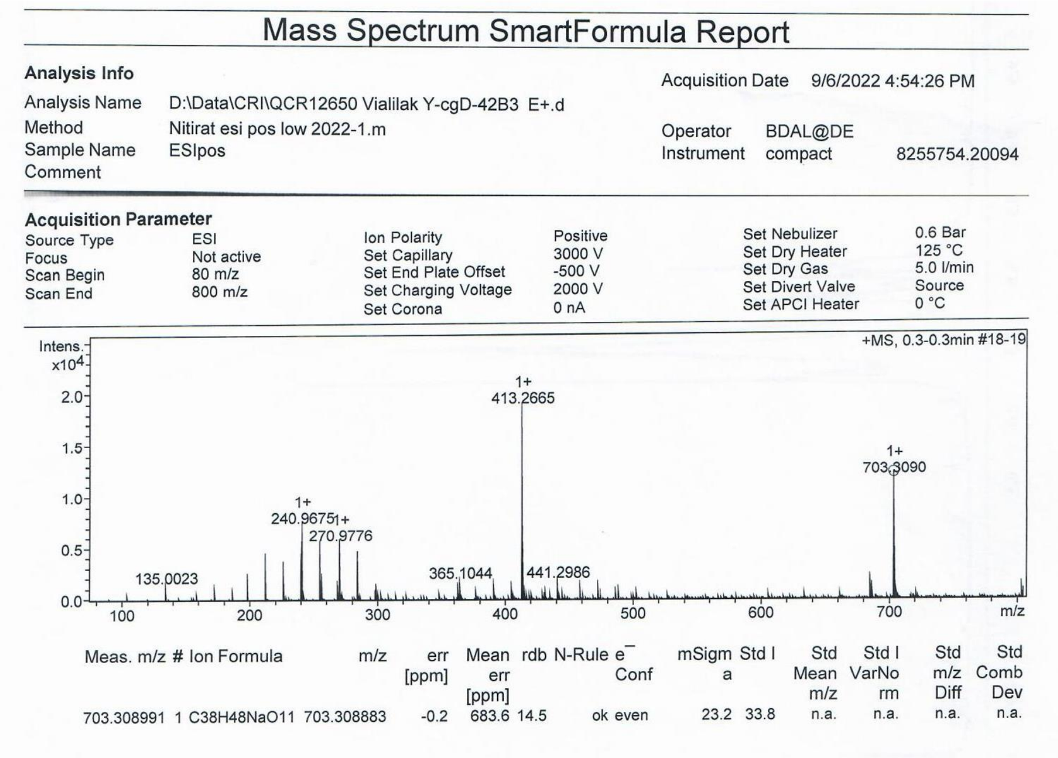

Mass Spectrum SmartFormula Report

Analysis Info

Analysis Name	D:\Data\CRI\QCR12650 Vialilak Y-cgD-42B3 E+.d	Acquisition Date	9/6/2022 4:54:26 PM
Method	Nitirat esi pos low 2022-1.m	Operator	BDAL@DE
Sample Name	ESIpos	Instrument	compact 8255754.20094
Comment			

Acquisition Parameter

Source Type	ESI	Ion Polarity	Positive	Set Nebulizer	0.6 Bar
Focus	Not active	Set Capillary	3000 V	Set Dry Heater	125 °C
Scan Begin	80 m/z	Set End Plate Offset	-500 V	Set Dry Gas	5.0 l/min
Scan End	800 m/z	Set Charging Voltage	2000 V	Set Divert Valve	Source
		Set Corona	0 nA	Set APCI Heater	0 °C

Meas. m/z	#	Ion Formula	m/z	err [ppm]	Mean err [ppm]	rdb	N-Rule	e⁻ Conf	mSigma	Std I	Std Mean m/z	Std I VarNorm	Std m/z Diff	Std Comb Dev
703.308991	1	C38H48NaO11	703.308883	-0.2	683.6	14.5	ok	even	23.2	33.8	n.a.	n.a.	n.a.	n.a.

Figure S117. ESITOFMS spectrum of compound **18**

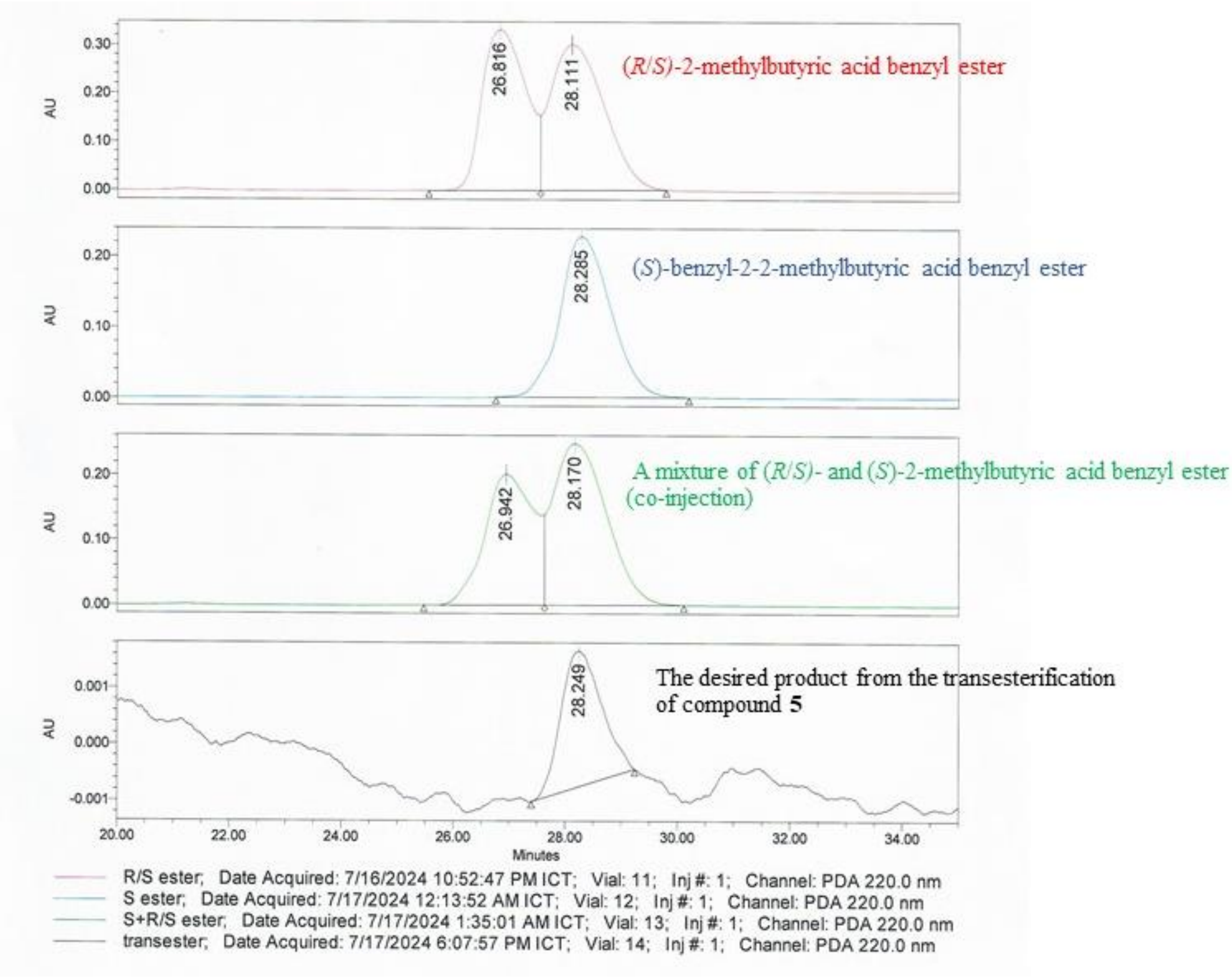

Figure S118. Chiral HPLC analysis of 2-methylbutyric acid benzyl ester derived from transesterification of **5** and synthetic samples

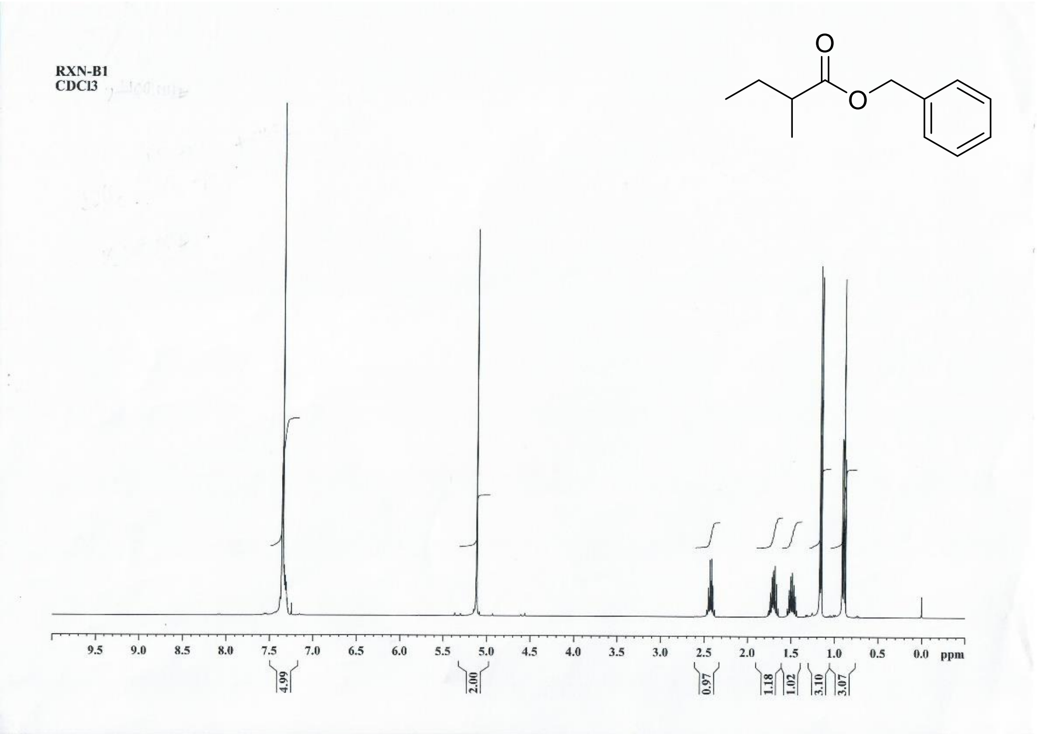

Figure S119. ^{1}H-NMR spectrum (400 MHz, $CDCl_3$) of *R*/*S*-2methylbutyric acid benzyl ester

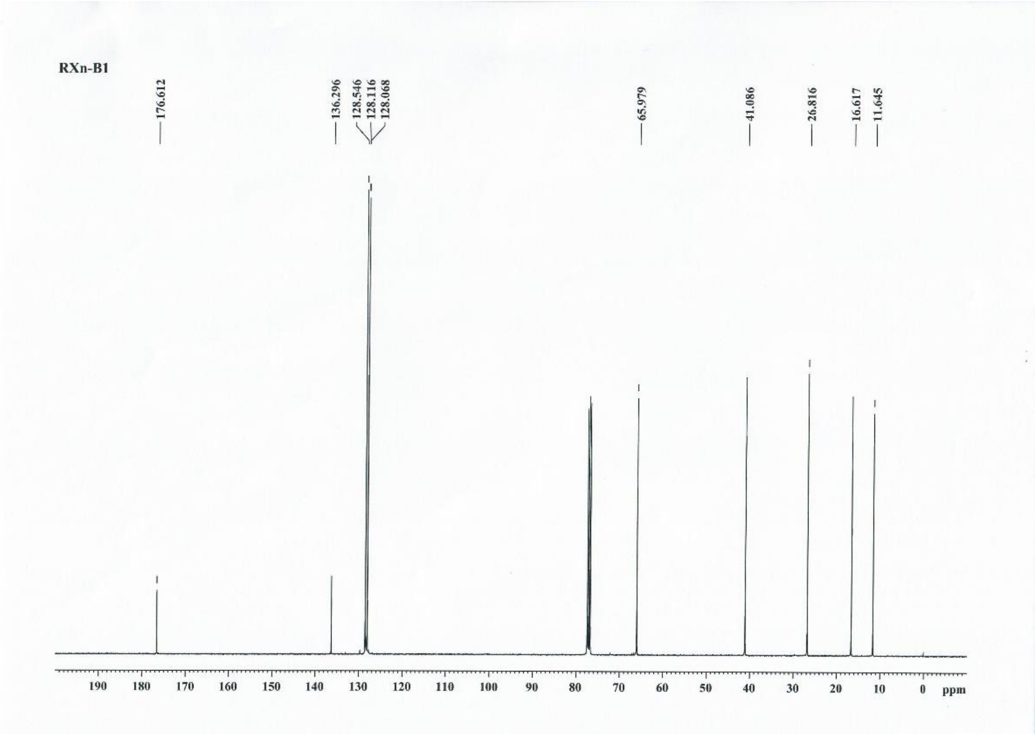

Figure S120. ^{13}C-NMR spectrum (100 MHz, $CDCl_3$) of *R*/*S*-2methylbutyric acid benzyl ester

Table S1. Conformational analysis of Compound **1**

Conformer of **1**	Gibbs Free Energy (Hartree)	Gibbs Free Energy (kcal/mol)	Relative Gibbs Free Energy (kcal/mol)	Population (%)
**1 –1**	-1998.22854	-1253888.408850	3.483880	0.3
**1 –2**	-1998.234092	-1253891.892730	0.000000	99.5
**1 –3**	-1998.22821	-1253888.201775	3.690955	0.2

Table S2. Conformational analysis of Compound **4**

Conformer **4**	Gibbs Free Energy (Hartree)	Gibbs Free Energy (kcal/mol)	Relative Gibbs Free Energy (kcal/mol)	Population (%)
Conformer **4-1**	-1770.436925	-1110949.170438	0.176328	18.6
Conformer **4-2**	-1770.435902	-1110948.528505	0.818260	6.4
Conformer **4-3**	-1770.435314	-1110948.159535	1.187230	3.4
Conformer **4-4**	-1770.437206	-1110949.346765	0.000000	25.0
Conformer **4-5**	-1770.434936	-1110947.922340	1.424425	2.3
Conformer **4-6**	-1770.435868	-1110948.507170	0.839595	6.2
Conformer **4-7**	-1770.435988	-1110948.582470	0.764295	7.0
Conformer **4-8**	-1770.435636	-1110948.361590	0.985175	4.8
Conformer **4-9**	-1770.436347	-1110948.807743	0.539023	10.2
Conformer **4-10**	-1770.435747	-1110948.431243	0.915523	5.4
Conformer **4-11**	-1770.435965	-1110948.568038	0.778728	6.8
Conformer **4-12**	-1770.435427	-1110948.230443	1.116323	3.9

Table S3. Conformational analysis of Compound **13**

Conformer **35-B8**	Gibbs Free Energy (Hartree)	Gibbs Free Energy (kcal/mol)	Relative Gibbs Free Energy (kcal/mol)	Population (%)
Conformer **13-1**	-2189.832118	-1374119.654045	0.000000	73.7
Conformer **13-2**	-2189.829979	-1374118.311823	1.342222	7.8
Conformer **13-3**	-2189.829486	-1374118.002465	1.651580	4.7
Conformer **13-4**	-2189.830520	-1374118.651300	1.002745	13.8

Table S4. Conformational analysis of Compound **14**

Conformer **14**	Gibbs Free Energy (Hartree)	Gibbs Free Energy (kcal/mol)	Relative Gibbs Free Energy (kcal/mol)	Population (%)
Conformer **14-1**	-2303.141967	-1445221.584293	0.796925	18.2
Conformer **14-2**	-2303.140945	-1445220.942988	1.438230	6.2
Conformer **14-3**	-2303.139291	-1445219.905103	2.476115	1.1
Conformer **14-4**	-2303.140144	-1445220.440360	1.940858	2.7
Conformer **14-6**	-2303.143237	-1445222.381218	0.000000	69.0
Conformer **14-7**	-2303.139744	-1445220.189360	2.191858	1.8

Table S5. Conformational analysis of Compound **17**

Conformer **17**	Gibbs Free Energy (Hartree)	Gibbs Free Energy (kcal/mol)	Relative Gibbs Free Energy (kcal/mol)	Population (%)
Conformer **17-1**	-2303.154005	-1445229.138138	1.483410	7.3
Conformer **17-2**	-2303.156369	-1445230.621548	0.000000	87.4
Conformer **17-3**	-2303.15262	-1445228.269050	2.352497	1.7
Conformer **17-4**	-2303.153268	-1445228.675670	1.945877	3.4

Table S6. Coordinates of Compound **1**

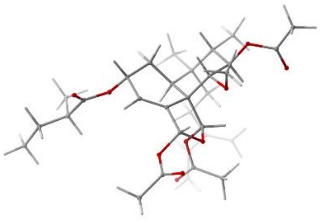

conformer **1-1**

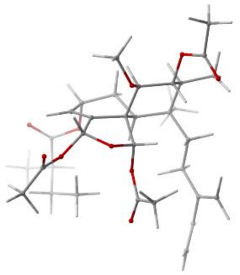

conformer **1-2**

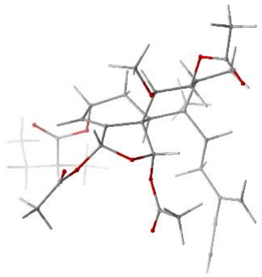

conformer **1-3**

O	4.192887	-1.829921	-2.152928	3.985932	-2.041379	-2.405107	4.411513	-1.703784	-1.756312
C	3.944604	-0.676665	-1.891411	3.692795	-0.870810	-2.332937	4.105111	-0.560831	-1.506623
C	4.962805	0.398098	-1.554304	4.671300	0.269736	-2.152111	5.063165	0.522944	-1.038992
C	6.372445	-0.197927	-1.515447	4.914148	0.492461	-0.643378	6.512984	0.148801	-1.376219
C	7.430771	0.765510	-0.986509	3.673728	0.849562	0.170430	6.845598	0.179461	-2.866243
O	2.692594	-0.184730	-1.854332	2.423081	-0.424972	-2.361436	2.840474	-0.111474	-1.574774
C	1.611089	-1.110453	-2.127104	1.366436	-1.417039	-2.342022	1.814669	-1.072202	-1.930421
H	1.911235	-1.726015	-2.987411	1.568527	-2.138963	-3.146963	2.203156	-1.679311	-2.760712
C	1.399373	-1.970457	-0.915668	1.368717	-2.097931	-1.003475	1.532377	-1.934307	-0.735056
C	0.364343	-1.775500	-0.106887	0.479286	-1.779481	-0.070100	0.434951	-1.760715	-0.007581
C	0.178683	-2.419512	1.239012	0.568591	-2.184128	1.375957	0.164611	-2.402022	1.325164
H	-0.312451	-3.400418	1.230905	0.192923	-3.184367	1.624544	-0.305249	-3.392619	1.287957
O	1.444314	-2.545026	1.867210	1.929825	-2.089152	1.772430	1.383258	-2.499108	2.044510
C	1.561847	-3.508530	2.819338	2.302525	-2.790693	2.875466	1.449999	-3.456501	3.007817
C	2.934223	-3.465711	3.428271	3.742150	-2.509832	3.198345	2.773424	-3.384524	3.714432
O	0.680474	-4.273101	3.110444	1.564905	-3.521933	3.481180	0.564696	-4.237061	3.238069
O	-0.640694	-1.533659	1.960661	-0.211152	-1.246442	2.071642	-0.722249	-1.528933	1.979822
C	-0.846127	-0.348228	1.244920	-0.570778	-0.184875	1.233405	-0.900564	-0.352725	1.241983
O	0.153667	0.602794	1.558761	0.425184	0.821588	1.259166	0.054325	0.621447	1.618757
C	-0.697684	-0.703881	-0.260419	-0.632994	-0.759117	-0.208071	-0.636453	-0.714130	-0.246122
C	-2.010469	-1.323903	-0.799352	-1.988571	-1.472319	-0.436676	-1.892898	-1.365630	-0.874550
H	-1.774201	-1.810995	-1.761477	-1.887341	-2.090279	-1.345600	-1.577172	-1.851327	-1.814374
O	-2.525332	-2.281675	0.097369	-2.319343	-2.297077	0.656893	-2.450208	-2.330815	-0.011833
C	-3.060605	-0.253326	-1.067430	-3.106139	-0.466183	-0.681358	-2.943692	-0.319315	-1.223749
H	-3.368236	0.224221	-0.127310	-3.277828	0.140978	0.217802	-3.327091	0.156250	-0.310970
O	-4.207508	-0.942406	-1.591150	-4.296259	-1.238565	-0.907601	-4.035557	-1.035281	-1.823462
C	-5.368522	-0.824084	-0.908569	-5.334800	-1.040274	-0.064920	-5.245527	-0.936821	-1.228225
O	-5.524774	-0.102084	0.042962	-5.361440	-0.191994	0.789995	-5.485275	-0.213246	-0.295467
C	-2.584725	0.788391	-2.078728	-2.835548	0.421591	-1.895319	-2.418964	0.727253	-2.205487
H	-2.302249	0.235987	-2.993289	-2.687650	-0.256079	-2.755648	-2.060608	0.176226	-3.093939

C	-3.732496	1.726967	-2.461238	-4.061229	1.283428	-2.210703	-3.555976	1.639106	-2.674717
C	-1.291219	1.497112	-1.572193	-1.501155	1.209600	-1.719340	-1.179955	1.466294	-1.613494
C	-0.756060	2.411962	-2.690831	-1.182919	1.946353	-3.035005	-0.584523	2.384545	-2.698340
C	-1.613777	2.388576	-0.346277	-1.648713	2.276637	-0.605025	-1.606775	2.358644	-0.420438
C	-0.769430	4.058922	1.411118	-0.565349	4.189863	0.724538	-0.920423	4.057990	1.378221
C	0.311630	4.983083	1.823606	0.559929	5.147716	0.820357	0.111027	5.006527	1.857113
C	0.801800	5.079097	3.060526	1.234704	5.423547	1.937591	0.514193	5.118917	3.123731
C	-0.214730	0.417649	-1.219364	-0.347058	0.206190	-1.389829	-0.109790	0.412444	-1.175561
H	0.613297	0.927295	-0.708706	0.535898	0.794823	-1.106200	0.667090	0.942983	-0.609019
C	0.404130	-0.251874	-2.464778	0.087372	-0.641166	-2.606161	0.614447	-0.249924	-2.366502
H	4.689504	0.747448	-0.541769	4.195679	1.176753	-2.557880	4.962167	0.499509	0.062287
H	6.350262	-1.103236	-0.889955	5.390566	-0.415461	-0.233353	7.176538	0.841908	-0.833098
H	7.566540	1.636839	-1.645266	3.131643	1.700334	-0.270974	6.193758	-0.506342	-3.428667
H	8.406019	0.263575	-0.903666	3.943600	1.142237	1.194598	7.885337	-0.137357	-3.036246
H	7.162601	1.141859	0.014208	2.965277	0.012804	0.248751	6.735472	1.188802	-3.292276
H	2.175695	-2.696379	-0.669242	2.198388	-2.769466	-0.780050	2.304743	-2.640423	-0.426432
H	3.693858	-3.576983	2.641811	4.370382	-2.737720	2.325785	3.589929	-3.488530	2.986070
H	3.032709	-4.264299	4.170730	4.050949	-3.109709	4.060627	2.831449	-4.175713	4.468968
H	3.092083	-2.485124	3.899629	3.861277	-1.438682	3.416334	2.879968	-2.397751	4.187213
H	-1.841509	0.015406	1.529856	-1.527485	0.195951	1.611776	-1.921161	-0.008569	1.452097
H	-4.199830	2.185255	-1.577275	-4.393231	1.860078	-1.334824	-4.095053	2.091022	-1.829177
H	-4.515152	1.165172	-2.989466	-4.900233	0.643469	-2.516778	-4.286663	1.058205	-3.254248
H	-3.394806	2.529139	-3.131385	-3.864620	1.983626	-3.033926	-3.188127	2.445394	-3.323717
H	0.291799	2.694261	-2.507857	-0.128628	2.259502	-3.071616	0.441763	2.689566	-2.443675
H	-1.339296	3.342765	-2.747647	-1.796768	2.853696	-3.133847	-1.180850	3.302667	-2.804410
H	-0.808617	1.932096	-3.679145	-1.379354	1.320562	-3.917908	-0.556396	1.896931	-3.683869
H	-1.947730	1.767489	0.494533	-1.831720	1.790679	0.361998	-1.983501	1.735641	0.400728
H	-2.477727	3.022455	-0.600240	-2.556022	2.865811	-0.810955	-2.464821	2.971216	-0.738698
H	0.750252	5.593335	1.024450	0.866026	5.620884	-0.120851	0.590173	5.621809	1.085533
H	0.444405	4.429301	3.862476	1.009700	4.911818	2.875971	0.116592	4.465901	3.903844
H	1.601440	5.783675	3.298510	2.056825	6.142290	1.938015	1.281327	5.841170	3.410675
H	0.743069	0.506586	-3.179625	0.279626	0.003941	-3.470903	0.985827	0.511986	-3.061196
H	-0.327751	-0.879930	-2.995509	-0.694765	-1.353268	-2.910326	-0.059559	-0.901772	-2.943046
C	-0.488939	3.300642	0.133276	-0.481777	3.247834	-0.454946	-0.537575	3.298560	0.127918
H	-0.248318	4.045715	-0.644658	-0.385637	3.867024	-1.363538	-0.260676	4.044272	-0.637251
H	0.439171	2.729208	0.297387	0.472067	2.703646	-0.361597	0.389571	2.749000	0.359034
C	-1.893490	3.944536	2.131971	-1.562422	4.199592	1.619923	-2.088958	3.924551	2.020776
H	-2.706544	3.273498	1.847972	-2.403349	3.505255	1.566584	-2.866680	3.235293	1.685906
H	-2.040708	4.549733	3.028542	-1.574380	4.932334	2.429055	-2.309879	4.531509	2.900860
C	-6.419249	-1.722180	-1.500216	-6.433320	-2.028210	-0.343735	-6.232149	-1.858808	-1.889419
H	-6.219750	-2.756232	-1.180239	-6.146221	-3.002917	0.079288	-6.032615	-2.887991	-1.554192
H	-7.407326	-1.420202	-1.136862	-7.359984	-1.691032	0.132818	-7.249635	-1.578436	-1.596879
H	-6.380187	-1.696794	-2.596896	-6.573452	-2.159605	-1.424451	-6.117064	-1.833289	-2.980774
C	-2.795625	-3.539142	-0.476612	-2.623104	-3.629641	0.317230	-2.652594	-3.595780	-0.597230
H	-3.543981	-3.465385	-1.284865	-3.489997	-3.688717	-0.363903	-3.342222	-3.540695	-1.457497
H	-1.877498	-4.004758	-0.882244	-1.760562	-4.132904	-0.159049	-1.698271	-4.043548	-0.933486
H	-3.189325	-4.176905	0.325588	-2.862062	-4.150145	1.253853	-3.089709	-4.238671	0.177930
C	4.824698	1.588573	-2.512626	5.973317	-0.025123	-2.892094	4.657332	1.923785	-1.506749
H	5.090554	1.293418	-3.540707	6.416461	-0.963246	-2.527759	4.600094	1.985028	-2.604069
H	3.796248	1.973213	-2.516420	5.807316	-0.131150	-3.974417	3.677638	2.208710	-1.102023
H	5.492256	2.408069	-2.211965	6.694937	0.789820	-2.733531	5.399869	2.660090	-1.163847
H	6.642559	-0.542255	-2.527822	5.661070	1.299273	-0.556004	6.710638	-0.858818	-0.981432
C	0.257187	1.130871	2.816153	0.687023	1.513178	2.409186	0.058440	1.158383	2.876621

C	-0.862610	0.864844	3.786871	-0.298616	1.399855	3.541855	-1.121009	0.873827	3.768059
H	-0.610612	1.345829	4.737448	0.059393	2.023386	4.367213	-0.946131	1.363769	4.731282
H	-1.800161	1.294542	3.403638	-1.287023	1.759263	3.219894	-2.037848	1.282483	3.317586
H	-1.008726	-0.214203	3.928279	-0.397514	0.355402	3.866522	-1.254654	-0.207466	3.904515
O	1.226109	1.795052	3.059547	1.678282	2.189444	2.438959	0.994211	1.843819	3.182694

Table S7. Coordinates of Compound **4**

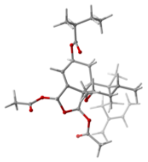

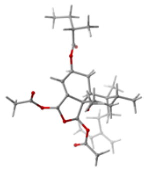

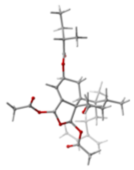

	conformer **4-1**			conformer **4-2**			conformer **4-3**		
C	1.162157	2.007920	0.037057	-0.945767	-2.160770	0.518722	-0.876814	-2.177506	0.489212
C	0.243516	0.906202	-0.605619	-0.108148	-0.942759	-0.009040	-0.111688	-0.927185	-0.071770
C	0.645683	0.936473	2.319095	-1.294906	-0.824897	2.681453	-1.165797	-0.865519	2.675331
C	0.724997	2.239607	1.518580	-0.880924	-2.171509	2.077022	-0.734522	-2.193475	2.042315
C	-1.171235	1.465103	-0.880650	1.408293	-1.186707	0.156818	1.418980	-1.116416	0.015198
C	-2.104736	0.490939	-1.591880	2.264122	-0.127759	-0.521461	2.200114	-0.024255	-0.699813
C	-0.593623	-2.738681	-0.070005	-0.140504	2.833144	0.198204	-0.269204	2.843836	0.164976
O	0.784883	-2.722125	0.158799	-1.497059	2.546271	0.153897	-1.615211	2.508168	0.184262
C	1.302744	-1.416699	0.065972	-1.713114	1.155190	0.026739	-1.787301	1.110831	0.059392
H	-1.291025	0.341682	1.605111	0.602334	0.141813	2.434977	0.681991	0.168383	2.340701
H	-0.298848	2.652914	1.472910	0.18035	-2.333977	2.338666	0.343451	-2.319127	2.250638
C	1.572847	3.263308	2.278578	-1.669947	-3.303979	2.738908	-1.449743	-3.356763	2.733878
H	0.680050	0.641593	-1.584655	-0.289676	-0.862409	-1.094447	-0.351326	-0.848071	-1.145981
O	-3.476515	0.953534	-1.540980	3.617174	-0.187783	-0.024693	3.577596	-0.035595	-0.273353
H	-1.908107	0.520791	-2.675189	2.306645	-0.322680	-1.606258	2.193023	-0.212645	-1.786618
H	-1.100838	-3.159559	0.806401	0.09518	3.446433	1.076863	-0.012728	3.458498	1.036803
O	-0.881429	-3.552625	-1.201965	0.161624	3.597332	-0.978999	-0.053414	3.627613	-1.018296
H	1.806540	-1.294884	-0.905655	-1.841921	0.902879	-1.037498	-1.960394	0.858775	-0.998548
C	-1.959254	-0.929509	-1.153194	1.766125	1.260508	-0.295680	1.664097	1.343823	-0.439444
C	-0.952824	-1.316187	-0.382173	0.549611	1.493493	0.184021	0.466802	1.530166	0.104630
C	2.664888	1.636225	-0.016793	-2.427859	-2.119859	0.070350	-2.379780	-2.188301	0.115101
C	3.252955	1.280542	-1.394110	-2.692113	-1.958855	-1.422844	-2.723707	-2.030146	-1.362066
C	0.119199	-0.419812	0.208458	-0.463793	0.442612	0.611118	-0.485122	0.441113	0.575145
C	-0.275320	-0.088162	1.669113	-0.452791	0.333348	2.157253	-0.391865	0.324267	2.117853
C	0.997446	3.333060	-0.737511	-0.341736	-3.470365	-0.031411	-0.253923	-3.461455	-0.098884
H	-2.649525	-1.664263	-1.574021	2.409695	2.084500	-0.611732	2.260507	2.192332	-0.782013
C	4.763834	1.351426	-1.388646	-4.145796	-2.023552	-1.830773	-4.193117	-2.142355	-1.696730
C	5.520716	0.244948	-1.319320	-5.148177	-2.287436	-0.978502	-5.141504	-2.444668	-0.796771
C	5.421304	2.670024	-1.444677	-4.463422	-1.778632	-3.249576	-4.590337	-1.901803	-3.096070
H	-1.643028	1.766401	0.062900	1.691541	-1.207322	1.220038	1.756706	-1.130677	1.062444
H	-1.104443	2.362261	-1.506546	1.690726	-2.154348	-0.269391	1.713554	-2.071726	-0.429982
C	4.822889	3.853619	-1.619668	-3.581951	-1.530620	-4.223457	-3.768218	-1.619796	-4.111717
C	-1.117986	-4.868713	-0.963161	1.126106	4.530499	-0.872883	0.882487	4.592869	-0.951528
C	-1.298021	-5.604932	-2.261629	1.205547	5.355440	-2.127938	0.870027	5.430284	-2.200788
O	-1.172454	-5.356666	0.134275	1.825769	4.667151	0.101678	1.625571	4.745800	-0.012130
C	-4.114534	0.962394	-0.357965	4.411129	-1.136789	-0.555173	4.380169	-0.952289	-0.848467
O	-3.632540	0.579505	0.683570	4.051056	-1.901692	-1.419152	4.003370	-1.729602	-1.692947
C	-5.501771	1.562331	-0.481170	5.774578	-1.136135	0.110575	5.788622	-0.868849	-0.287846
C	-6.515513	0.695780	0.264881	5.642974	-1.834759	1.472342	5.762671	-1.168225	1.217876
C	-5.465098	2.996267	0.079049	6.82848	-1.798288	-0.777561	6.732111	-1.779417	-1.077890
C	-4.483915	3.922595	-0.634096	7.120891	-1.027749	-2.061624	8.200572	-1.621300	-0.694948
O	2.270867	-1.291969	1.085294	-2.913861	0.884014	0.702623	-2.941715	0.793214	0.793370

O	3.760188	-2.385167	-0.210724	-4.079519	1.367694	-1.175902	-4.218730	1.243965	-1.019953
C	3.488791	-1.826423	0.820842	-4.061927	1.061228	-0.015552	-4.130654	0.932661	0.135896
C	4.434324	-1.565192	1.956158	-5.254767	0.814316	0.860098	-5.267480	0.639108	1.069751
O	-0.341324	-1.284685	2.418843	-0.839707	1.572720	2.710748	-0.793115	1.546393	2.698941
C	-1.402109	-1.306891	3.343317	-0.263775	1.846494	3.962022	-0.163179	1.834133	3.920724
H	0.255561	1.161687	3.325171	-1.174882	-0.878614	3.775612	-0.990234	-0.920182	3.761901
H	1.641274	0.494005	2.457031	-2.355378	-0.610061	2.490614	-2.241215	-0.687341	2.537705
H	2.588368	2.885980	2.473322	-2.75617	-3.160866	2.631222	-2.544306	-3.253942	2.675822
H	1.661860	4.219173	1.742656	-1.413059	-4.289528	2.324799	-1.175537	-4.330575	2.303407
H	1.112473	3.475808	3.255519	-1.452057	-3.332581	3.817462	-1.182021	-3.380855	3.801265
H	3.224637	2.501296	0.368955	-2.880382	-3.069918	0.393579	-2.781816	-3.155246	0.454686
H	2.879277	0.811844	0.672462	-2.960068	-1.330066	0.611284	-2.912343	-1.420460	0.686374
H	2.954074	0.266125	-1.694183	-2.30482	-0.992418	-1.782737	-2.387121	-1.049773	-1.735162
H	2.862408	1.960103	-2.168674	-2.143176	-2.727146	-1.994586	-2.178960	-2.777034	-1.965322
H	0.048589	3.837934	-0.505509	0.573074	-3.766969	0.501580	0.697076	-3.726286	0.385259
H	1.805249	4.032383	-0.478577	-1.058123	-4.299134	0.064415	-0.933719	-4.316488	0.027716
H	1.035684	3.179535	-1.827455	-0.089636	-3.383001	-1.099769	-0.059587	-3.360055	-1.178016
H	6.611753	0.315518	-1.294799	-6.182463	-2.316990	-1.328913	-6.190574	-2.507982	-1.094795
H	5.081752	-0.754449	-1.288023	-4.983502	-2.485557	0.081119	-4.916778	-2.642035	0.251913
H	6.512215	2.646724	-1.346148	-5.528791	-1.810695	-3.500297	-5.665381	-1.968683	-3.292827
H	5.406999	4.774726	-1.662684	-3.915746	-1.361240	-5.248637	-4.159153	-1.457236	-5.117614
H	3.740727	3.950329	-1.731792	-2.505841	-1.480873	-4.043380	-2.686633	-1.534633	-3.986047
H	-2.060614	-5.107133	-2.876415	1.321523	4.697367	-3.000171	0.968806	4.783935	-3.083876
H	-0.352896	-5.578700	-2.823154	0.265865	5.910913	-2.259219	-0.095240	5.950345	-2.281770
H	-1.583956	-6.642396	-2.059432	2.047961	6.051430	-2.057683	1.688070	6.157249	-2.164212
H	-5.752808	1.606828	-1.551966	6.042954	-0.081724	0.289017	6.102644	0.182458	-0.416807
H	-6.579281	-0.312952	-0.169457	4.887779	-1.341359	2.100548	5.059566	-0.503089	1.736761
H	-7.515463	1.151820	0.218127	6.604625	-1.810478	2.005668	6.757956	-1.024105	1.660905
H	-6.224597	0.592722	1.320393	5.352699	-2.889057	1.340039	5.456734	-2.211284	1.400292
H	-6.484780	3.409672	0.010404	7.753796	-1.901965	-0.186984	6.413144	-2.826139	-0.942319
H	-5.218491	2.941281	1.152878	6.489404	-2.817508	-1.023631	6.604811	-1.567189	-2.150596
H	-4.546010	4.943632	-0.229846	7.905469	-1.525990	-2.650013	8.839688	-2.236952	-1.344803
H	-3.443366	3.581589	-0.515887	6.222866	-0.960133	-2.693615	8.529780	-0.574357	-0.798417
H	-4.693842	3.971506	-1.714167	7.465624	-0.004051	-1.842556	8.395095	-1.931085	0.343229
H	5.302414	-2.227402	1.870220	-6.168413	0.883804	0.261135	-6.212401	0.676960	0.518379
H	4.774003	-0.519592	1.884062	-5.179245	-0.180088	1.321888	-5.132267	-0.353192	1.522335
H	3.930429	-1.697779	2.921864	-5.269944	1.558433	1.669386	-5.268609	1.379694	1.882435
H	-1.307745	-0.508609	4.103453	0.84133	1.865978	3.902811	-0.392637	1.085320	4.701177
H	-2.379128	-1.194954	2.838066	-0.61983	2.837972	4.270475	0.935913	1.894506	3.804502
H	-1.365051	-2.278991	3.852456	-0.560761	1.112426	4.733698	-0.538533	2.810112	4.254659

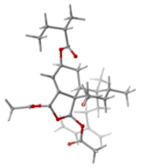

conformer **4-4**

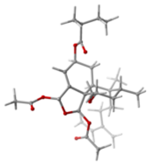

conformer **4-5**

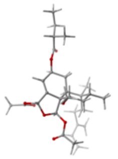

conformer **4-6**

	conformer **4-4**			conformer **4-5**			conformer **4-6**		
C	-0.972616	-2.100229	-0.833272	-0.80111	-2.30576	-0.02114	-0.54613	-2.11251	-0.07827
C	-0.115198	-0.785637	-0.832618	-0.09245	-1.03172	-0.60835	0.12055	-0.71713	-0.32362
C	-0.505124	-2.241662	1.699092	-0.35886	-1.32493	2.31191	-0.85211	-1.37620	2.38181
C	-0.518301	-2.997158	0.362872	-0.25538	-2.56616	1.41855	-0.39122	-2.48696	1.43022
C	1.343262	-1.089196	-1.254681	1.38205	-1.33863	-0.95609	1.66163	-0.82024	-0.25827
C	2.248310	0.136894	-1.333629	2.11984	-0.18359	-1.62537	2.39167	0.46652	-0.61753
C	0.354664	2.281953	1.268350	0.16819	2.65892	0.15028	-0.37008	2.87512	0.61434
O	-0.963054	1.977797	1.550332	-1.17385	2.40370	0.43458	-1.67436	2.41767	0.67060
C	-1.385520	0.826142	0.840807	-1.48472	1.04109	0.24214	-1.76007	1.04248	0.35872
H	1.373832	-1.238152	1.412834	1.41849	-0.35783	1.59144	0.91832	-0.17117	2.24081
H	0.523889	-3.291912	0.147180	0.81934	-2.79483	1.30402	0.69347	-2.62084	1.59349
C	-1.311207	-4.298097	0.513555	-0.87979	-3.77128	2.12686	-1.05378	-3.80896	1.82590
H	-0.531137	-0.100780	-1.589419	-0.59628	-0.77763	-1.55686	-0.13098	-0.39235	-1.34652
O	3.647248	-0.231986	-1.398718	3.54888	-0.41958	-1.64470	3.73673	0.44104	-0.09150
H	2.109557	0.625215	-2.311287	1.88907	-0.17774	-2.70238	2.47220	0.55291	-1.71400
H	0.902484	2.450558	2.204134	0.64420	3.10936	1.02969	-0.13350	3.42448	1.53443
O	0.467766	3.556598	0.607198	0.26648	3.57076	-0.94090	-0.21670	3.88008	-0.40074
H	-1.899677	1.149330	-0.071875	-1.97928	0.91878	-0.73393	-2.06356	0.92764	-0.69117
C	1.975856	1.179059	-0.299932	1.76488	1.16462	-1.08816	1.72293	1.69367	-0.10051
C	0.883449	1.136755	0.449753	0.73897	1.33499	-0.26610	0.48851	1.66138	0.38718
C	-2.492498	-1.826107	-0.723211	-2.34256	-2.17012	0.03619	-2.03541	-2.11571	-0.49996
C	-3.088974	-0.842099	-1.724635	-3.05309	-1.79566	-1.26007	-2.31652	-1.81589	-1.98155
C	-0.110378	-0.005600	0.517036	-0.15285	0.24128	0.29106	-0.35382	0.42212	0.62954
C	0.330322	-0.965747	1.646858	0.34759	-0.11478	1.71299	-0.17172	-0.03858	2.09664
C	-0.743636	-2.858703	-2.157780	-0.48617	-3.52346	-0.91587	0.17008	-3.18733	-0.92524
H	2.611934	2.069076	-0.305067	2.31239	2.02594	-1.47787	2.22000	2.65085	-0.27950
C	-4.578374	-0.621449	-1.598337	-4.56146	-1.75410	-1.17706	-3.77445	-2.02447	-2.31951
C	-5.373884	-1.352904	-0.802776	-5.25697	-2.09740	-0.08215	-4.19168	-3.05489	-3.06622
C	-5.175259	0.460086	-2.403311	-5.29528	-1.30769	-2.37530	-4.71206	-1.02296	-1.77025
H	1.797247	-1.806268	-0.561018	1.93004	-1.61685	-0.04778	1.99355	-1.11411	0.74860
H	1.349808	-1.559008	-2.245138	1.43587	-2.19559	-1.63736	2.01679	-1.59570	-0.94443
C	-4.518300	1.293597	-3.216288	-4.75501	-0.96114	-3.54801	-5.90528	-1.29180	-1.23179
C	-0.131338	3.717590	-0.591909	0.29114	4.89223	-0.63063	-0.49441	3.54563	-1.68081
C	0.001100	5.142434	-1.055019	0.28506	5.71853	-1.88690	-0.37695	4.75002	-2.57300
O	-0.693860	2.843286	-1.204791	0.31448	5.32231	0.49203	-0.79369	2.43719	-2.05090
C	4.243724	-0.740827	-0.306312	4.22756	-0.39458	-0.48469	4.64524	-0.26279	-0.79346
O	3.672678	-0.995675	0.728703	3.73386	-0.15647	0.59379	4.39107	-0.83655	-1.82556
C	5.730997	-0.929133	-0.539917	5.68656	-0.75424	-0.68904	6.00040	-0.23584	-0.10854
C	5.992515	-1.857925	-1.728284	6.57885	0.21992	0.07909	5.89241	-0.90344	1.26997
C	6.414848	0.435692	-0.720196	5.90145	-2.20535	-0.22069	7.06691	-0.86229	-1.01044
C	6.211400	1.382660	0.458941	5.05257	-3.23470	-0.96179	8.48753	-0.71697	-0.47311
O	-2.300712	0.167466	1.682036	-2.40283	0.69798	1.25190	-2.76056	0.51289	1.20144
O	-3.928146	1.589708	1.012248	-4.09470	1.48336	-0.02843	-4.36001	1.61355	0.04533
C	-3.573074	0.656292	1.677972	-3.70770	1.00714	1.00376	-4.04011	0.88920	0.94999

C	-4.432131	-0.155101	2.604210	-4.55067	0.65899	2.19530	-4.96637	0.24019	1.93747
O	0.322709	-0.263588	2.875419	0.23843	1.02197	2.54545	-0.60085	1.00197	2.95106
C	1.341577	-0.673284	3.754254	1.30984	1.15948	3.44709	0.00255	0.98960	4.21773
H	-0.080432	-2.904795	2.470540	0.10616	-1.54911	3.28586	-0.61045	-1.68358	3.41237
H	-1.524787	-1.992977	2.022043	-1.40712	-1.06568	2.51486	-1.94047	-1.24705	2.33470
H	-2.344143	-4.109129	0.844503	-1.93182	-3.58730	2.39400	-2.15194	-3.73529	1.80484
H	-1.353031	-4.874345	-0.422197	-0.83752	-4.68535	1.51702	-0.75544	-4.63883	1.16936
H	-0.839103	-4.938443	1.274299	-0.33887	-3.97495	3.06352	-0.76562	-4.08106	2.85297
H	-2.999789	-2.794630	-0.852718	-2.73245	-3.14608	0.36388	-2.44043	-3.11966	-0.29608
H	-2.736950	-1.491529	0.290824	-2.62381	-1.45394	0.81542	-2.61865	-1.42884	0.12593
H	-2.606290	0.142105	-1.630603	-2.72564	-0.80357	-1.60950	-2.04307	-0.77516	-2.22655
H	-2.881379	-1.168792	-2.758796	-2.77631	-2.49744	-2.06604	-1.70481	-2.46387	-2.62536
H	0.224632	-3.379623	-2.180846	0.53059	-3.91037	-0.75417	1.12240	-3.50427	-0.47605
H	-1.524936	-3.617063	-2.312649	-1.18446	-4.34782	-0.71064	-0.45927	-4.08410	-1.02578
H	-0.778900	-2.177928	-3.022984	-0.58438	-3.27729	-1.98501	0.38808	-2.82863	-1.94245
H	-6.445960	-1.149927	-0.749874	-6.34795	-2.04261	-0.07673	-5.24549	-3.17790	-3.32566
H	-4.997164	-2.169012	-0.185065	-4.77832	-2.44214	0.83503	-3.48608	-3.79318	-3.45588
H	-6.260142	0.569678	-2.302816	-6.38399	-1.26726	-2.26602	-4.36942	0.01781	-1.78578
H	-5.052769	2.068746	-3.768421	-5.38481	-0.64230	-4.38037	-6.54111	-0.48513	-0.86026
H	-3.435416	1.252996	-3.351998	-3.67729	-0.97423	-3.72495	-6.27546	-2.31725	-1.14303
H	-0.579784	5.792608	-0.384786	1.08675	5.38800	-2.56167	-1.15196	5.47984	-2.29763
H	1.050115	5.464255	-1.000382	-0.67013	5.56774	-2.41057	0.59898	5.23379	-2.42890
H	-0.378254	5.231822	-2.078373	0.40821	6.77628	-1.63190	-0.50563	4.44585	-3.61699
H	6.104249	-1.397034	0.383989	5.89751	-0.69533	-1.76776	6.23801	0.83116	0.05121
H	5.506584	-2.835315	-1.589143	6.46289	1.24924	-0.29150	5.10348	-0.43252	1.87166
H	7.073274	-2.030349	-1.842011	7.63599	-0.06481	-0.02787	6.83931	-0.81656	1.82104
H	5.612670	-1.415308	-2.660561	6.31870	0.21289	1.14757	5.65753	-1.97506	1.16382
H	7.491987	0.256539	-0.873812	6.97042	-2.44402	-0.34612	6.82362	-1.92681	-1.16277
H	6.037374	0.898895	-1.646734	5.69218	-2.25343	0.86136	6.99878	-0.39742	-2.00604
H	6.737039	2.334849	0.295283	5.29171	-4.25412	-0.62508	9.21780	-1.11263	-1.19418
H	5.145121	1.611911	0.615322	3.97655	-3.07273	-0.79164	8.73913	0.34059	-0.28991
H	6.591223	0.941745	1.394030	5.22506	-3.18664	-2.04851	8.63244	-1.26280	0.47175
H	-5.436738	0.278079	2.646245	-5.60482	0.84308	1.96431	-5.95697	0.70253	1.87038
H	-4.488897	-1.188797	2.231970	-4.39941	-0.39604	2.46407	-5.04758	-0.82854	1.68573
H	-3.978736	-0.183935	3.604595	-4.23435	1.26964	3.05309	-4.56268	0.32496	2.95493
H	1.232014	-1.731782	4.056869	1.37306	0.30861	4.15139	-0.25922	0.08951	4.80369
H	2.341649	-0.542682	3.301434	2.27540	1.24549	2.91525	1.10599	1.04712	4.14221
H	1.263445	-0.047509	4.653505	1.12953	2.07628	4.02373	-0.36272	1.87269	4.75832

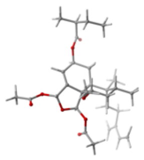

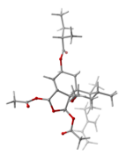

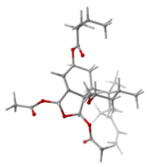

	conformer **4-7**			conformer **4-8**			conformer **4-9**		
C	-0.928311	-2.016844	-0.190090	-0.610512	-2.262197	0.368998	-1.158035	-1.965076	0.027046
C	0.070045	-0.857858	-0.552784	0.099153	-0.979485	-0.184559	-0.221430	-0.890688	-0.626527
C	-1.117707	-0.945879	2.140327	-1.102246	-0.921859	2.508825	-0.654339	-0.844435	2.292014
C	-0.884590	-2.239911	1.351679	-0.562389	-2.231606	1.928291	-0.733592	-2.164832	1.516821
C	1.547162	-1.284071	-0.425726	1.633010	-1.060659	-0.026447	1.203278	-1.445780	-0.844752
C	2.517810	-0.251213	-0.980848	2.363712	0.052444	-0.761251	2.142572	-0.481773	-1.564631
C	0.441142	2.834401	0.109454	-0.306274	2.766797	-0.046708	0.492535	2.773203	-0.258352
O	-0.933334	2.711215	-0.127954	-1.622811	2.332232	-0.258916	-0.913901	2.703735	-0.278072
C	-1.328763	1.360524	-0.236143	-1.738498	0.935172	-0.158502	-1.369440	1.398967	-0.035515
H	0.879527	-0.178337	1.998401	0.698042	0.208343	2.236085	1.274178	-0.243085	1.568314
H	0.142897	-2.579351	1.579031	0.509092	-2.287233	2.194910	0.288656	-2.583122	1.486118
C	-1.826984	-3.327754	1.868391	-1.241633	-3.421389	2.611079	-1.587884	-3.171084	2.292172
H	-0.097135	-0.623211	-1.619176	-0.096572	-0.939728	-1.270058	-0.628889	-0.656780	-1.625307
O	3.845653	-0.509355	-0.479519	3.723387	0.156470	-0.290222	3.518666	-0.917086	-1.455819
H	2.561316	-0.332122	-2.079602	2.407604	-0.178595	-1.838833	1.978983	-0.558040	-2.651248
H	0.606808	3.262336	1.105609	-0.253216	3.343146	0.884504	0.836797	3.219441	0.682991
O	1.009949	3.697656	-0.863537	0.085467	3.596872	-1.126805	0.917829	3.576712	-1.340896
H	-1.597877	1.150227	-1.280813	-1.963299	0.519554	-1.154210	-1.999018	1.093874	-0.881032
C	2.160406	1.147461	-0.605322	1.723169	1.385964	-0.569509	1.956016	0.954780	-1.194050
C	0.980675	1.446821	-0.073641	0.505615	1.507082	-0.052678	0.922013	1.354642	-0.466892
C	-2.347977	-1.614171	-0.689534	-2.071924	-2.366852	-0.132168	-2.649690	-1.556895	-0.052154
C	-3.312408	-2.714605	-1.166243	-2.259075	-2.507368	-1.651597	-3.231035	-1.339179	-1.472719
C	-0.140221	0.470140	0.237007	-0.396859	0.375732	0.410227	-0.136339	0.460687	0.145540
C	-0.145458	0.165603	1.755876	-0.370577	0.298602	1.958778	0.254445	0.176377	1.616389
C	-0.476821	-3.304300	-0.901387	0.134694	-3.522338	-0.121220	-1.013099	-3.305488	-0.722519
H	2.863488	1.941138	-0.866614	2.247888	2.264456	-0.950652	2.634802	1.685951	-1.638871
C	-4.615249	-2.107305	-1.636779	-3.697002	-2.808961	-2.006295	-4.343008	-2.309566	-1.796339
C	-4.858722	-1.921684	-2.942536	-4.079677	-4.011818	-2.453578	-4.168607	-3.321432	-2.659714
C	-5.619291	-1.669911	-0.652403	-4.654443	-1.698125	-1.824023	-5.640249	-2.147269	-1.115701
H	1.820116	-1.460844	0.625869	1.925217	-1.006305	1.033247	1.654690	-1.710945	0.119085
H	1.726448	-2.218645	-0.966244	2.009896	-2.011605	-0.415439	1.158935	-2.365888	-1.439600
C	-5.578337	-1.870554	0.669516	-5.881686	-1.810660	-1.307254	-5.997467	-1.113423	-0.345294
C	1.068879	5.018717	-0.551124	-0.111749	4.934018	-0.976499	1.086124	4.903391	-1.091967
C	1.608223	5.800308	-1.716418	0.253256	5.653653	-2.244328	1.457326	5.620949	-2.359367
O	0.734410	5.477021	0.508800	-0.518892	5.446337	0.031723	0.953240	5.408199	-0.009729
C	4.544849	-1.484035	-1.095072	4.604562	-0.730651	-0.792476	4.125510	-0.868901	-0.256286
O	4.134164	-2.098750	-2.049868	4.310756	-1.569137	-1.610770	3.610793	-0.442879	0.751302
C	5.882730	-1.704636	-0.416639	5.982369	-0.530188	-0.187102	5.526056	-1.448608	-0.339818
C	6.754114	-0.448413	-0.503669	5.920013	-0.781337	1.326458	6.270078	-1.249106	0.976816
C	5.676374	-2.154327	1.038431	7.014303	-1.399389	-0.910392	5.457281	-2.922521	-0.793771
C	4.814778	-3.406117	1.177287	8.453231	-1.128884	-0.481150	4.616593	-3.821755	0.109990
O	-2.484532	1.226979	0.560897	-2.832646	0.664964	0.696509	-2.182641	1.449844	1.128803
O	-3.813819	1.670798	-1.217143	-4.274951	1.369860	-0.891745	-4.029192	1.828289	-0.118977
C	-3.677449	1.502122	-0.034997	-4.068353	0.962276	0.220894	-3.497601	1.705997	0.957151

C	-4.759703	1.568596	1.002280	-5.104491	0.669286	1.266460	-4.196281	1.748943	2.285628
O	-0.385161	1.367173	2.456606	-0.863034	1.512682	2.486356	0.309802	1.396129	2.331010
C	0.160049	1.402570	3.749745	-0.316361	1.863263	3.731438	1.398218	1.471586	3.221290
H	-0.994402	-1.165384	3.213187	-0.973792	-0.943157	3.603267	-0.255276	-1.047865	3.299296
H	-2.145408	-0.580861	2.009007	-2.177839	-0.818471	2.316292	-1.652550	-0.408711	2.432022
H	-2.879486	-3.021913	1.771606	-2.336054	-3.385181	2.498654	-2.603182	-2.787703	2.476782
H	-1.698839	-4.284077	1.342419	-0.890274	-4.386164	2.218376	-1.678211	-4.134657	1.770601
H	-1.642406	-3.511398	2.937950	-1.025678	-3.406701	3.690350	-1.132143	-3.368588	3.274469
H	-2.854332	-1.035203	0.091332	-2.519566	-3.263193	0.325339	-3.229421	-2.356826	0.430666
H	-2.242035	-0.924517	-1.541358	-2.669117	-1.519676	0.228263	-2.824369	-0.670780	0.565202
H	-2.871708	-3.267041	-2.007639	-1.956793	-1.579176	-2.167076	-3.611213	-0.309871	-1.564685
H	-3.502275	-3.452129	-0.370972	-1.619334	-3.310023	-2.044788	-2.450293	-1.444506	-2.240000
H	0.458407	-3.699840	-0.482075	1.046886	-3.717367	0.461112	-0.083619	-3.831521	-0.458945
H	-1.224170	-4.101638	-0.806012	-0.507625	-4.410907	-0.031146	-1.852526	-3.975294	-0.488087
H	-0.319078	-3.130001	-1.978251	0.429070	-3.441046	-1.178442	-1.018210	-3.162306	-1.814399
H	-5.777613	-1.442227	-3.287961	-5.117097	-4.209975	-2.731712	-4.970314	-4.034043	-2.869684
H	-4.143147	-2.236506	-3.705749	-3.360626	-4.826445	-2.572338	-3.222776	-3.465033	-3.187110
H	-6.481880	-1.143095	-1.074180	-4.300239	-0.702455	-2.113633	-6.362083	-2.953693	-1.285623
H	-6.386754	-1.521174	1.314490	-6.531484	-0.936803	-1.222850	-6.991469	-1.076912	0.105435
H	-4.750550	-2.391590	1.155320	-6.267964	-2.770511	-0.952597	-5.339704	-0.260331	-0.161397
H	2.570303	5.379434	-2.040123	1.270278	5.378255	-2.556041	2.336986	5.147064	-2.816763
H	0.910291	5.714789	-2.561756	-0.432915	5.341095	-3.044724	0.628141	5.538845	-3.076760
H	1.725592	6.851003	-1.431583	0.178527	6.734772	-2.087955	1.660475	6.674068	-2.139176
H	6.356038	-2.521324	-0.982849	6.229079	0.535395	-0.342212	6.041614	-0.889422	-1.138838
H	6.912893	-0.141727	-1.548270	5.153666	-0.150571	1.796569	6.358719	-0.181580	1.222215
H	7.739512	-0.639874	-0.053059	6.885515	-0.553915	1.799534	7.282311	-1.674781	0.908147
H	6.285176	0.388741	0.034058	5.679359	-1.836634	1.534278	5.742782	-1.730103	1.812583
H	6.670451	-2.334201	1.480361	6.761054	-2.460346	-0.749654	5.054289	-2.954101	-1.817235
H	5.225441	-1.322486	1.603971	6.913889	-1.231515	-1.993794	6.489053	-3.307160	-0.848047
H	4.727371	-3.709915	2.230855	9.156295	-1.720632	-1.085532	4.612615	-4.854862	-0.267764
H	3.795215	-3.238851	0.793758	8.715424	-0.065923	-0.609961	5.000920	-3.846340	1.140844
H	5.243330	-4.250797	0.615215	8.628548	-1.390319	0.573623	3.568734	-3.481904	0.151511
H	-5.737497	1.623516	0.512745	-5.202499	-0.422952	1.363219	-5.182191	2.211512	2.169083
H	-4.709765	0.690526	1.659446	-4.790750	1.074235	2.237483	-4.323260	0.715368	2.644509
H	-4.595458	2.463301	1.621008	-6.066455	1.092742	0.958657	-3.588504	2.288770	3.022603
H	-0.296233	0.652578	4.421530	-0.560011	1.128512	4.520679	1.354706	2.458880	3.700035
H	1.254716	1.236677	3.732712	0.784721	1.965242	3.676336	1.342463	0.696855	4.009012
H	-0.041383	2.402067	4.156305	-0.749078	2.832373	4.011560	2.361881	1.362309	2.691147

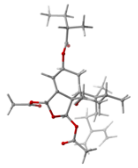

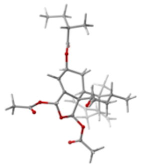

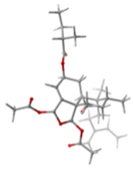

	conformer **4-10**			conformer **4-11**			conformer **4-12**		
C	-0.784389	-1.925946	0.420644	-0.900401	-2.050066	0.658163	-1.060196	-1.958754	0.383439
C	0.042533	-0.676576	-0.042015	-0.127510	-0.820100	0.066741	-0.153448	-0.800326	-0.163662
C	-1.050800	-0.749037	2.695930	-1.605759	-0.507797	2.600228	-1.288389	-0.600547	2.547686
C	-0.684750	-2.050772	1.973074	-1.027414	-1.869024	2.203964	-0.976737	-1.972305	1.940581
C	1.560508	-0.943095	0.077826	1.382216	-0.888841	0.383338	1.345681	-1.149277	-0.032112
C	2.435086	0.169834	-0.481736	2.190450	0.149636	-0.378689	2.260757	-0.151054	-0.724796
C	-0.000822	3.063675	0.335595	-0.496836	2.936367	-0.162037	0.088829	2.968800	0.059587
O	-1.355405	2.777653	0.383443	-1.812761	2.508562	-0.364689	-1.286110	2.778241	0.029698
C	-1.587928	1.391116	0.232404	-1.908223	1.103975	-0.284130	-1.600165	1.408539	-0.096228
H	0.849240	0.212264	2.439652	0.229510	0.586702	2.427922	0.671456	0.222241	2.272645
H	0.378423	-2.255862	2.193581	0.006079	-1.906018	2.594737	0.074499	-2.206749	2.188757
C	-1.482262	-3.213631	2.569313	-1.801277	-2.986141	2.908901	-1.833890	-3.047049	2.614081
H	-0.167250	-0.508747	-1.111803	-0.212022	-0.880908	-1.031159	-0.358317	-0.706574	-1.244495
O	3.775486	0.061821	0.046629	3.503991	0.287324	0.203267	3.615467	-0.308417	-0.256382
H	2.507409	0.070130	-1.577625	2.328358	-0.179640	-1.422214	2.266134	-0.342665	-1.811021
H	0.272594	3.683352	1.198999	-0.469324	3.624775	0.690703	0.376883	3.554108	0.941629
O	0.315547	3.914675	-0.775786	-0.031176	3.620099	-1.319562	0.427996	3.722174	-1.113383
H	-1.812101	1.180620	-0.821141	-1.945428	0.685676	-1.300404	-1.764687	1.166144	-1.158357
C	1.930552	1.536105	-0.166297	1.554930	1.499444	-0.368600	1.867427	1.267656	-0.482589
C	0.701656	1.732105	0.295454	0.295906	1.672844	0.017551	0.681174	1.583655	0.024329
C	-2.268928	-1.845269	-0.009590	-2.314830	-2.218163	0.041295	-2.538275	-1.822992	-0.055097
C	-2.550780	-1.635798	-1.506554	-2.489906	-2.179787	-1.490152	-2.816573	-1.676924	-1.558074
C	-0.295811	0.656972	0.691056	-0.659914	0.586912	0.484810	-0.397079	0.605540	0.465813
C	-0.214050	0.436062	2.221769	-0.801336	0.653386	2.026309	-0.370668	0.492579	2.011661
C	-0.196369	-3.199441	-0.225044	-0.103317	-3.345910	0.388396	-0.551003	-3.309312	-0.164986
H	2.548731	2.384789	-0.470798	2.127207	2.335968	-0.775133	2.561574	2.045840	-0.807533
C	-3.983129	-1.991117	-1.846367	-1.770165	-3.229170	-2.305227	-4.268203	-1.956579	-1.879530
C	-4.928908	-1.055487	-2.008475	-0.806397	-2.891243	-3.175922	-5.097608	-1.014638	-2.348738
C	-4.275076	-3.438396	-1.950904	-2.156507	-4.646045	-2.179555	-4.713595	-3.350392	-1.652198
H	1.850853	-1.088576	1.129231	1.570146	-0.735971	1.457237	1.647776	-1.194019	1.025259
H	1.827204	-1.861737	-0.454289	1.784268	-1.872193	0.121654	1.550362	-2.132785	-0.466082
C	-5.185010	-3.994209	-2.756049	-3.222653	-5.123298	-1.527788	-5.903737	-3.717293	-1.169023
C	0.045147	3.473899	-2.026027	-0.233035	4.962935	-1.358954	1.459183	4.582034	-1.012055
C	0.352067	4.546729	-3.033952	0.222440	5.514912	-2.680960	1.578944	5.414640	-2.258723
O	-0.380138	2.377362	-2.290470	-0.707560	5.600463	-0.456680	2.181377	4.655256	-0.047246
C	4.579298	-0.848798	-0.532615	4.417717	-0.636718	-0.148950	4.331323	-1.309379	-0.804970
O	4.243601	-1.540279	-1.465334	4.196257	-1.531041	-0.931048	3.898635	-2.045301	-1.659663
C	5.921059	-0.913944	0.173926	5.719566	-0.424097	0.601236	5.722158	-1.376853	-0.200174
C	5.740957	-1.719574	1.469364	5.546633	-0.974219	2.025016	5.615795	-1.674797	1.302376
C	6.999091	-1.511406	-0.730892	6.895445	-1.073994	-0.128766	6.588128	-2.381451	-0.964604
C	7.339930	-0.640902	-1.936755	7.215006	-0.428412	-1.473782	8.052125	-2.381321	-0.534812
O	-2.719749	1.095334	1.018912	-3.129473	0.826355	0.361267	-2.806566	1.211347	0.600507
O	-4.052548	1.898807	-0.617306	-4.214573	0.816602	-1.624167	-3.954159	1.930955	-1.206110
C	-3.924370	1.380286	0.460519	-4.235083	0.738749	-0.424857	-3.946048	1.502695	-0.081879

C	-5.038075	0.906946	1.348250	-5.448878	0.504507	0.427724	-5.152610	1.159713	0.740475
O	-0.548696	1.646584	2.868024	-1.334658	1.911415	2.379972	-0.656014	1.756215	2.572349
C	-0.015698	1.777397	4.159439	-0.982782	2.348882	3.666596	-0.036157	1.986560	3.811472
H	-0.881266	-0.893241	3.775248	-1.598015	-0.432262	3.699604	-1.159775	-0.661265	3.640512
H	-2.115510	-0.512551	2.569948	-2.652888	-0.408968	2.284375	-2.333188	-0.312987	2.367706
H	-2.567231	-3.040062	2.501600	-2.873882	-2.954245	2.662485	-2.908832	-2.831543	2.515670
H	-1.258265	-4.170289	2.075902	-1.422071	-3.984572	2.649120	-1.648294	-4.049056	2.201511
H	-1.237904	-3.328459	3.636510	-1.715326	-2.872779	4.000464	-1.607216	-3.086900	3.690499
H	-2.739548	-2.793643	0.294348	-2.709313	-3.176278	0.409008	-3.058644	-2.729850	0.290555
H	-2.794177	-1.066224	0.553504	-2.979595	-1.448631	0.452111	-3.020183	-0.990070	0.468138
H	-2.360264	-0.593468	-1.799668	-3.571863	-2.265672	-1.689383	-2.555422	-0.671469	-1.918280
H	-1.876369	-2.262956	-2.112375	-2.213561	-1.193806	-1.885308	-2.194137	-2.388723	-2.128198
H	0.721924	-3.536458	0.277107	0.733992	-3.466187	1.091920	0.346059	-3.663497	0.362957
H	-0.917851	-4.028497	-0.176186	-0.751168	-4.227659	0.492264	-1.321676	-4.086500	-0.056791
H	0.045877	-3.044049	-1.287328	0.302812	-3.369895	-0.631352	-0.300920	-3.247577	-1.235384
H	-5.970752	-1.332923	-2.187072	-0.289164	-3.645039	-3.774843	-6.129228	-1.257836	-2.614363
H	-4.692122	0.009493	-1.956618	-0.509346	-1.850043	-3.322999	-4.767710	0.017196	-2.487480
H	-3.651661	-4.099446	-1.336290	-1.504386	-5.354011	-2.702536	-3.975879	-4.129252	-1.884008
H	-5.337975	-5.075146	-2.773872	-3.437697	-6.193439	-1.515795	-6.166091	-4.770337	-1.048020
H	-5.796421	-3.389321	-3.431115	-3.925005	-4.474388	-0.999664	-6.651901	-2.975395	-0.876288
H	-0.333311	5.392772	-2.880340	1.260256	5.212915	-2.878907	1.630620	4.761123	-3.140495
H	1.375440	4.919961	-2.890121	-0.401963	5.092766	-3.481618	0.681320	6.040295	-2.366639
H	0.226979	4.144968	-4.044871	0.134912	6.606332	-2.673499	2.471974	6.045095	-2.195374
H	6.190694	0.120059	0.445931	5.876135	0.665225	0.669237	6.150168	-0.364766	-0.313396
H	4.968623	-1.272191	2.111148	4.702152	-0.491794	2.537463	4.971163	-0.939443	1.802137
H	6.684813	-1.748050	2.033495	6.457236	-0.793607	2.614982	6.605879	-1.638463	1.777838
H	5.447749	-2.756742	1.242276	5.365416	-2.060377	2.000709	5.194710	-2.679632	1.469194
H	7.903596	-1.673143	-0.121341	7.777791	-1.017635	0.530042	6.155294	-3.388404	-0.844621
H	6.660409	-2.503169	-1.071417	6.670632	-2.143099	-0.272709	6.518554	-2.155013	-2.039728
H	8.139181	-1.097630	-2.538931	8.083911	-0.910429	-1.945671	8.642353	-3.060704	-1.167171
H	6.463928	-0.511358	-2.589577	6.365678	-0.521050	-2.166965	8.494098	-1.375316	-0.622889
H	7.686139	0.357369	-1.622784	7.448700	0.642377	-1.356693	8.179533	-2.711581	0.507446
H	-5.972755	1.398494	1.057769	-6.344497	0.491177	-0.201862	-6.031147	1.670340	0.331789
H	-5.148602	-0.179696	1.203131	-5.345942	-0.456741	0.953458	-5.310686	0.071449	0.670773
H	-4.805468	1.093109	2.404348	-5.523072	1.292019	1.189994	-4.997237	1.421750	1.794568
H	-0.428314	1.034814	4.866586	-1.390821	1.697933	4.461413	1.066113	1.930127	3.728551
H	1.087400	1.678796	4.154840	0.116325	2.403170	3.789427	-0.316628	2.999354	4.128516
H	-0.281255	2.780618	4.517772	-1.404065	3.354919	3.790112	-0.366568	1.272565	4.588309

Table S8. Coordinates of Compound **13**

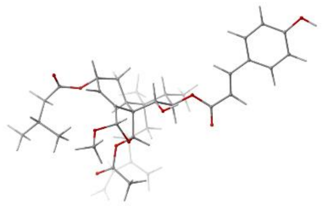

conformer **13-1**

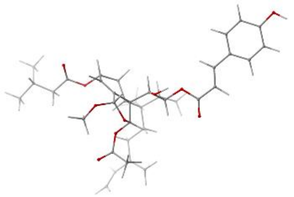

conformer **13-2**

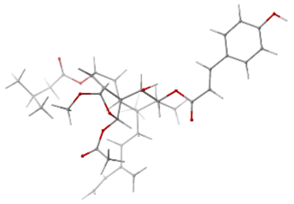

conformer **13-3**

O	3.943849	-4.245030	-1.481889	4.068094	-4.185514	0.150568	3.919276	-4.399029	-1.033087
C	4.152742	-3.080176	-1.725759	4.305923	-3.138314	-0.403708	4.163788	-3.277964	-1.414857
C	6.158167	-2.251329	-0.461247	6.769397	-3.701000	-0.744577	6.537186	-2.649206	-0.770988
C	7.574102	-1.700472	-0.627920	6.561615	-4.752714	-1.835538	5.998663	-1.776593	0.363152
O	3.172803	-2.167596	-1.871447	3.357851	-2.244618	-0.736950	3.252298	-2.287504	-1.427997
C	1.822058	-2.591687	-1.559645	1.992950	-2.562034	-0.353557	1.912034	-2.614780	-0.979223
H	1.626240	-3.526710	-2.104215	1.831757	-3.628572	-0.565605	1.694490	-3.637741	-1.317746
C	1.723366	-2.802959	-0.076703	1.834402	-2.282140	1.110600	1.859576	-2.541153	0.518478
C	1.190024	-1.876437	0.710412	1.199873	-1.197303	1.539780	1.252214	-1.535107	1.138945
C	1.307838	-1.863754	2.208877	1.190515	-0.717696	2.972707	1.274664	-1.285896	2.630371
H	0.475807	-2.371131	2.729215	0.344347	-1.122324	3.543605	0.407570	-1.735002	3.132929
O	2.524452	-2.415335	2.579655	2.317500	-1.041441	3.712502	2.383024	-1.767711	3.306572
O	1.208021	-0.485575	2.559546	0.996246	0.691574	2.856980	1.146337	0.133290	2.735716
C	1.174197	0.322031	1.422581	0.954564	1.100777	1.523915	1.095489	0.737762	1.479902
O	2.483199	0.727191	1.053088	2.241617	1.521691	1.094832	2.394743	1.136822	1.063710
C	0.609151	-0.548245	0.265719	0.557625	-0.136391	0.670229	0.605347	-0.337657	0.470082
C	-0.933442	-0.588624	0.330210	-0.978136	-0.303690	0.666393	-0.935217	-0.443137	0.514138
H	-1.263773	-1.422170	-0.318681	-1.195587	-1.326139	0.302881	-1.215019	-1.387712	0.010282
C	-1.550148	0.690207	-0.222299	-1.645180	0.674041	-0.293325	-1.593022	0.697576	-0.253859
H	-1.285090	1.552602	0.403604	-1.500409	1.709144	0.043536	-1.384104	1.661025	0.229854
O	-2.974177	0.501082	-0.103005	-3.054646	0.389735	-0.196950	-3.009367	0.460695	-0.133766
C	-3.703963	1.520582	0.417460	-3.902366	1.442309	-0.065473	-3.798864	1.513691	0.200705
O	-3.223467	2.560642	0.802954	-3.536786	2.592505	0.001775	-3.376191	2.624356	0.421718
C	-1.166684	0.928506	-1.680678	-1.165320	0.490229	-1.731116	-1.182890	0.719832	-1.724343
H	-1.449534	0.015124	-2.235049	-1.336079	-0.570944	-1.987798	-1.414892	-0.280607	-2.132466
C	-1.972575	2.082079	-2.283539	-2.005104	1.321495	-2.704638	-2.020181	1.727335	-2.516517
C	0.381939	1.060174	-1.809718	0.374740	0.723879	-1.814718	0.361404	0.899010	-1.845549
C	0.748520	1.153744	-3.303183	0.850331	0.387670	-3.241161	0.762401	0.764222	-3.327100
C	0.869274	2.360428	-1.120635	0.704945	2.213841	-1.544219	0.777730	2.313975	-1.369302
C	2.831557	3.921880	-0.567367	2.485024	4.058171	-1.412519	2.664621	4.021837	-1.026763
C	4.235823	4.295184	-0.853118	3.875423	4.463722	-1.720762	4.065841	4.396405	-1.325211
C	5.156227	4.566960	0.073372	4.685290	5.110490	-0.880913	4.939245	4.859654	-0.429626
C	1.063196	-0.200278	-1.179626	1.095187	-0.212193	-0.786542	1.075620	-0.211308	-1.005628
H	2.140432	0.002614	-1.121989	2.149280	0.093156	-0.744927	2.144260	0.038781	-0.970213
C	0.926727	-1.467680	-2.050460	1.100809	-1.693054	-1.223016	0.992421	-1.609779	-1.652083
H	8.193531	-2.354921	-1.261447	5.585483	-5.247354	-1.729556	5.628091	-0.803276	0.005918
H	8.072252	-1.601435	0.348529	7.339998	-5.529477	-1.784021	6.779415	-1.586303	1.115266
H	7.550801	-0.700317	-1.092002	6.613867	-4.292534	-2.837314	5.166329	-2.285193	0.875872
H	2.216318	-3.676453	0.351226	2.343787	-2.940321	1.817640	2.388257	-3.306699	1.091122
H	0.554387	1.193039	1.671870	0.238365	1.930564	1.466381	0.433524	1.608589	1.570457
H	-1.879703	3.004349	-1.692623	-2.029964	2.384499	-2.425944	-1.981424	2.733639	-2.075880
H	-3.039928	1.820286	-2.313603	-3.043704	0.961040	-2.707884	-3.074469	1.415890	-2.525630
H	-1.661321	2.294092	-3.315378	-1.629392	1.237340	-3.733357	-1.690731	1.789372	-3.562555
H	1.816552	0.944567	-3.465198	1.940744	0.243274	-3.274056	1.841825	0.577704	-3.432134

H	0.550013	2.163417	-3.691955	0.607851	1.204425	-3.936855	0.534537	1.687642	-3.879655
H	0.169998	0.447025	-3.916125	0.377351	-0.525725	-3.630770	0.228498	-0.056975	-3.827470
H	0.688801	2.305357	-0.039533	0.444548	2.476611	-0.511042	0.568296	2.427898	-0.298182
H	0.246346	3.195386	-1.478280	0.049369	2.832061	-2.177445	0.133551	3.051494	-1.873379
H	4.523008	4.301219	-1.911880	4.254603	4.161905	-2.704923	4.394997	4.232020	-2.358660
H	4.926121	4.490243	1.138343	4.367887	5.354897	0.135266	4.669833	4.960470	0.624124
H	6.177099	4.835821	-0.205965	5.702457	5.380772	-1.172091	5.960861	5.116921	-0.716808
H	1.218551	-1.253032	-3.084829	1.468720	-1.786999	-2.251100	1.287331	-1.564542	-2.706517
H	-0.110031	-1.836714	-2.080717	0.087763	-2.123360	-1.208558	-0.032675	-2.009522	-1.626417
C	2.330886	2.730543	-1.352202	2.146569	2.637884	-1.805981	2.230149	2.703421	-1.626181
H	2.503187	2.945991	-2.420792	2.385799	2.528753	-2.877912	2.422171	2.757438	-2.711720
H	2.993576	1.884317	-1.107584	2.852600	1.980129	-1.273254	2.917423	1.933574	-1.239106
C	2.095459	4.618108	0.309901	1.624136	4.914835	-0.845674	1.875439	4.820506	-0.294874
H	1.057236	4.365143	0.534072	0.593540	4.640474	-0.611989	0.838865	4.567128	-0.064014
H	2.514565	5.489669	0.816402	1.928676	5.938519	-0.619595	2.247095	5.777161	0.077258
H	6.225333	-3.250749	0.003321	6.697194	-4.205731	0.233177	7.406600	-2.136849	-1.217764
C	3.216312	1.554362	1.850728	2.840087	2.620070	1.643415	3.097901	2.087882	1.744596
C	2.523479	2.212316	3.015349	1.996576	3.529306	2.496853	2.359051	2.913997	2.762953
H	3.264082	2.805216	3.561546	2.631810	4.344424	2.858001	3.066732	3.618232	3.211996
H	1.729680	2.879369	2.647184	1.174516	3.949616	1.898718	1.549870	3.475732	2.273289
H	2.066793	1.461866	3.673879	1.563166	2.975776	3.340702	1.915307	2.269397	3.533681
O	4.368933	1.731914	1.562988	4.000897	2.801331	1.396098	4.261025	2.216606	1.474164
C	2.700732	-2.514170	3.975849	3.532119	-0.438046	3.298358	3.607920	-1.109907	3.028531
H	1.883532	-3.095150	4.442718	3.561965	0.626952	3.578410	3.669283	-0.147211	3.561568
H	3.652640	-3.033547	4.140450	4.333557	-0.969963	3.826027	4.405225	-1.771407	3.390364
H	2.743011	-1.518981	4.448139	3.692250	-0.519116	2.211087	3.747164	-0.924474	1.952838
C	5.316609	-1.349364	0.442795	8.147871	-3.050309	-0.860674	7.004050	-4.009311	-0.250588
H	5.158668	-0.355122	-0.004282	8.251883	-2.515339	-1.819826	6.160782	-4.583802	0.161247
H	4.328491	-1.775498	0.669412	8.322443	-2.323996	-0.051492	7.455838	-4.610750	-1.054890
H	5.826590	-1.189597	1.405341	8.945499	-3.807258	-0.813234	7.757672	-3.881520	0.541894
C	5.513601	-2.448702	-1.849379	5.676218	-2.632481	-0.785028	5.503905	-2.789992	-1.910728
H	5.427937	-1.479787	-2.363461	5.904308	-1.814431	-0.079510	5.362306	-1.821576	-2.409217
H	6.139456	-3.121776	-2.453653	5.610900	-2.157762	-1.777843	5.867324	-3.518626	-2.650977
C	-5.146283	1.220952	0.465367	-5.316154	1.029924	-0.011208	-5.226543	1.154605	0.268223
H	-5.736803	2.018323	0.918747	-6.003390	1.864556	0.134150	-5.865146	1.983545	0.576519
C	-5.689950	0.088990	-0.012550	-5.727201	-0.242539	-0.143241	-5.704118	-0.064565	-0.033511
H	-5.011183	-0.644265	-0.457947	-4.957151	-1.004597	-0.295079	-4.979969	-0.823853	-0.343162
C	-7.105839	-0.282926	-0.011006	-7.104635	-0.736875	-0.111810	-7.100478	-0.502504	-0.000748
C	-8.114384	0.531984	0.525431	-8.217095	0.097012	0.078236	-8.159934	0.333014	0.384622
C	-7.487768	-1.513237	-0.570298	-7.338773	-2.111507	-0.279194	-7.409279	-1.821727	-0.370410
C	-9.443938	0.136347	0.504035	-9.504911	-0.418496	0.099805	-9.468382	-0.127850	0.399556
H	-7.862373	1.495687	0.970454	-8.081239	1.171083	0.212942	-7.965304	1.364817	0.680872
C	-8.813136	-1.921665	-0.598589	-8.620796	-2.640767	-0.259857	-8.712763	-2.295967	-0.360485
H	-6.721796	-2.166520	-0.994817	-6.489651	-2.782537	-0.428779	-6.602486	-2.492879	-0.674266
C	-9.802449	-1.094992	-0.059089	-9.715367	-1.792884	-0.069508	-9.753687	-1.447355	0.026278
H	-10.212890	0.787608	0.928024	-10.356803	0.250110	0.249454	-10.277972	0.540817	0.703888
H	-9.104262	-2.877819	-1.034016	-8.797077	-3.708690	-0.390266	-8.947043	-3.321016	-0.648460
O	-11.080970	-1.533206	-0.108126	-10.946724	-2.351924	-0.057807	-11.008429	-1.951812	0.021288
H	-11.657860	-0.873956	0.294291	-11.606562	-1.662994	0.081316	-11.626016	-1.268969	0.307080
O	-1.358899	-0.819492	1.653575	-1.481349	-0.145347	1.972554	-1.382290	-0.466654	1.849841
H	-2.323021	-0.801540	1.632070	-2.439797	-0.229179	1.905903	-2.345287	-0.503030	1.811951

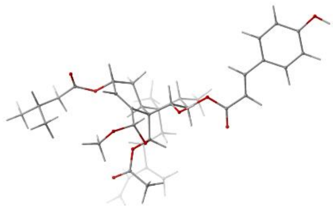

conformer **13-4**

O	3.954478	-4.289719	-0.819973
C	4.182800	-3.173820	-1.224315
C	6.656834	-3.161454	-0.693368
C	8.025957	-2.758893	-1.239329
O	3.234413	-2.234931	-1.383590
C	1.878691	-2.599602	-1.007896
H	1.688687	-3.611345	-1.393637
C	1.780757	-2.572282	0.488231
C	1.202615	-1.557550	1.120597
C	1.266223	-1.326155	2.612562
H	0.425026	-1.790590	3.143882
O	2.404713	-1.808090	3.239502
O	1.127191	0.088753	2.741422
C	1.050029	0.716660	1.498201
O	2.335849	1.156212	1.083748
C	0.567379	-0.345559	0.470680
C	-0.972539	-0.460423	0.512941
H	-1.245381	-1.400132	-0.004186
C	-1.639875	0.684985	-0.238390
H	-1.438576	1.643971	0.257175
O	-3.054349	0.435402	-0.121672
C	-3.852166	1.478142	0.224805
O	-3.437828	2.588077	0.464506
C	-1.229241	0.727665	-1.708253
H	-1.451373	-0.270327	-2.127699
C	-2.076625	1.735000	-2.489910
C	0.313589	0.923789	-1.825841
C	0.715768	0.816416	-3.309238
C	0.713114	2.336106	-1.327756
C	2.572244	4.070770	-0.972846
C	3.963488	4.476206	-1.277711
C	4.836737	4.939133	-0.381809
C	1.039899	-0.192472	-1.002397
H	2.105114	0.070495	-0.960330
C	0.974901	-1.579284	-1.677701
H	8.188793	-3.155042	-2.253991
H	8.833326	-3.138865	-0.594952
H	8.124212	-1.661324	-1.286014
H	2.291813	-3.355584	1.051764
H	0.368771	1.569651	1.612434
H	-2.047324	2.737392	-2.039770
H	-3.127836	1.413347	-2.501076
H	-1.748998	1.810426	-3.535606
H	1.797594	0.646555	-3.417412
H	0.475064	1.744828	-3.847762
H	0.192739	-0.004451	-3.821578

H	0.506333	2.429589	-0.254118
H	0.056796	3.072663	-1.817356
H	4.285168	4.337405	-2.317294
H	4.575637	5.013767	0.676168
H	5.850445	5.221168	-0.673949
H	1.292291	-1.509516	-2.724411
H	-0.049773	-1.981399	-1.682654
C	2.158490	2.750984	-1.583834
H	2.347204	2.818753	-2.669165
H	2.859015	1.988734	-1.205446
C	1.773838	4.846331	-0.226316
H	0.743834	4.571577	0.009454
H	2.130981	5.805198	0.154217
H	6.589683	-4.261907	-0.676671
C	3.006524	2.123194	1.776476
C	2.243464	2.905377	2.812023
H	2.928744	3.626786	3.268440
H	1.412027	3.446719	2.336890
H	1.827532	2.233404	3.574767
O	4.162403	2.300487	1.503971
C	3.627950	-1.188374	2.877891
H	3.710671	-0.183216	3.320950
H	4.424818	-1.824872	3.282494
H	3.749608	-1.102862	1.786134
C	6.454190	-2.649650	0.733732
H	6.474072	-1.546914	0.764195
H	5.490708	-2.986219	1.145339
H	7.248105	-3.019601	1.400507
C	5.544970	-2.656498	-1.619966
H	5.519752	-1.556725	-1.650006
H	5.728058	-3.005248	-2.650812
C	-5.277860	1.109043	0.279345
H	-5.923306	1.928673	0.598103
C	-5.745995	-0.107679	-0.046069
H	-5.015020	-0.856976	-0.363954
C	-7.139848	-0.554503	-0.029717
C	-8.207292	0.269528	0.358347
C	-7.437818	-1.870476	-0.419321
C	-9.513008	-0.199256	0.357014
H	-8.021259	1.298611	0.669230
C	-8.738443	-2.352589	-0.425658
H	-6.624669	-2.532665	-0.725877
C	-9.787423	-1.515401	-0.035781
H	-10.328971	0.460592	0.663567
H	-8.964325	-3.375094	-0.729012
O	-11.039042	-2.027197	-0.057265
H	-11.662923	-1.351839	0.232607
O	-1.414061	-0.506838	1.849829
H	-2.376938	-0.549250	1.816086

Table S9. Coordinates of Compound **14**

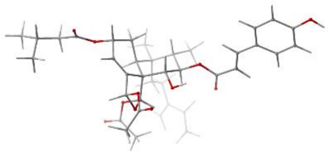

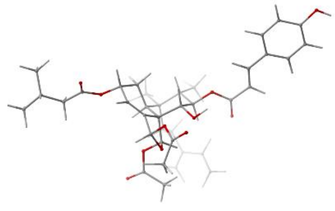

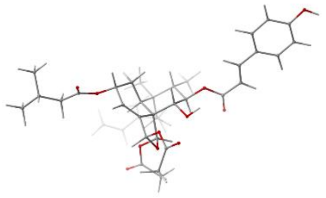

	conformer **14-1**			conformer **14-2**			conformer **14-3**		
O	4.676838	-3.707940	-1.360238	4.777414	-3.744837	-0.566461	4.239936	-3.162056	-2.372772
C	4.846387	-2.554855	-1.680445	4.945842	-2.640170	-1.027250	4.496870	-2.003083	-2.144984
C	7.385501	-2.762622	-1.695076	7.427544	-3.032511	-1.462486	6.948903	-2.255317	-2.798190
C	8.636767	-2.215941	-2.382429	7.252276	-4.003669	-2.631134	6.718625	-2.382396	-4.304973
O	3.853369	-1.649516	-1.734501	3.942485	-1.773561	-1.253718	3.564760	-1.088112	-1.826576
C	2.521765	-2.116700	-1.399943	2.605577	-2.212985	-0.903986	2.190573	-1.546808	-1.751990
H	2.382768	-3.095063	-1.882345	2.498121	-3.251295	-1.249785	2.015296	-2.206730	-2.613951
C	2.413554	-2.249964	0.089670	2.440496	-2.139809	0.584575	2.000017	-2.301313	-0.470634
C	1.705307	-1.390113	0.812703	1.693708	-1.200378	1.153556	1.309494	-1.783433	0.538331
C	1.720743	-1.336476	2.314610	1.647712	-0.939674	2.632879	1.240586	-2.383735	1.914990
H	2.707769	-1.596148	2.728861	2.618615	-1.126637	3.118306	2.199879	-2.833666	2.216443
O	0.743833	-2.213739	2.827118	0.657203	-1.750977	3.222399	0.223443	-3.354951	1.962183
O	1.427659	0.007425	2.627405	1.332132	0.430614	2.744542	0.954950	-1.293953	2.767809
C	1.330413	0.792851	1.465701	1.273981	1.045943	1.481836	0.981783	-0.080209	2.065517
O	2.589600	1.372781	1.191276	2.538498	1.598287	1.176589	2.284030	0.475866	2.147354
C	0.975543	-0.161222	0.293115	0.972564	-0.066383	0.441153	0.677310	-0.401892	0.578453
C	-0.560647	-0.373205	0.251913	-0.559171	-0.303580	0.373218	-0.857460	-0.464879	0.376925
H	-0.749954	-1.318367	-0.290097	-0.718872	-1.318999	-0.034529	-1.046441	-1.019937	-0.560053
C	-1.281760	0.747241	-0.495699	-1.260902	0.688248	-0.553391	-1.468716	0.926541	0.237098
H	-1.233986	1.671856	0.094034	-1.246903	1.687449	-0.099055	-1.406056	1.448600	1.201187
O	-2.663189	0.338708	-0.521515	-2.635510	0.257445	-0.575056	-2.866260	0.693686	-0.020295
C	-3.596874	1.249537	-0.159302	-3.593479	1.194081	-0.381664	-3.766373	1.338338	0.763214
O	-3.340145	2.392166	0.149556	-3.362600	2.372489	-0.223774	-3.459898	2.098258	1.652707
C	-0.764495	0.979796	-1.911984	-0.689635	0.727611	-1.967480	-0.868193	1.776107	-0.879247
H	-0.796295	0.005232	-2.430416	-0.689887	-0.310532	-2.343988	-1.007822	1.214105	-1.820544
C	-1.702496	1.901185	-2.698664	-1.605718	1.513430	-2.911384	-1.637459	3.091736	-1.018219
C	0.742811	1.373728	-1.844122	0.809216	1.151571	-1.897551	0.666619	1.931046	-0.673291
C	1.309235	1.546805	-3.262448	1.431325	1.135078	-3.302989	1.276232	2.610670	-1.910148
C	1.016282	2.680624	-1.055800	1.037589	2.559129	-1.288774	1.009043	2.763352	0.600425
C	0.387467	5.065699	-0.601218	0.368379	4.974122	-1.190634	2.461362	4.805685	-0.014907
C	-0.766129	4.783733	0.286354	-0.814993	4.801179	-0.314385	3.676212	4.137658	0.503299
C	-0.873306	5.136316	1.571971	-0.974464	5.327749	0.904602	4.746868	3.828458	-0.233606
C	1.504826	0.206352	-1.123772	1.553120	0.107788	-0.992586	1.308298	0.505797	-0.516878
H	2.545822	0.533966	-0.991878	2.585179	0.465576	-0.868804	2.359780	0.660892	-0.234329
C	1.557268	-1.088707	-1.962708	1.650480	-1.290827	-1.639207	1.329152	-0.299064	-1.835056
H	8.536624	-2.232478	-3.479172	6.308743	-4.561545	-2.546468	5.734944	-2.823181	-4.521153
H	9.523500	-2.810819	-2.115724	8.074427	-4.735189	-2.658756	7.484065	-3.026058	-4.764976
H	8.830161	-1.173530	-2.078017	7.253738	-3.462302	-3.592845	6.774370	-1.394468	-4.793675
H	2.992716	-3.043203	0.566825	3.009443	-2.849339	1.189076	2.503945	-3.265399	-0.374316
H	0.567193	1.552935	1.663532	0.498273	1.816561	1.543995	0.240732	0.576969	2.532861
H	-1.953244	2.814693	-2.143723	-1.888108	2.491900	-2.501292	-1.670438	3.641828	-0.066234
H	-2.649221	1.379607	-2.900449	-2.538126	0.953149	-3.071980	-2.676532	2.894408	-1.317668

H	-1.272908	2.183957	-3.669323	-1.141732	1.663257	-3.895716	-1.194429	3.740880	-1.785545
H	2.410179	1.578465	-3.236173	2.529979	1.185321	-3.238175	2.375804	2.563600	-1.879944
H	0.964561	2.484610	-3.718092	1.098091	1.996286	-3.897257	1.002485	3.670020	-1.965855
H	1.005257	0.734110	-3.937892	1.162018	0.232946	-3.871075	0.930985	2.143397	-2.843290
H	2.092951	2.720573	-0.834243	2.104822	2.646887	-1.037241	1.969357	2.394761	0.977024
H	0.522118	2.625146	-0.077575	0.508093	2.630807	-0.330261	0.274204	2.538987	1.388603
H	-1.585970	4.199779	-0.149900	-1.611886	4.149855	-0.694095	3.666525	3.837237	1.557866
H	-0.077442	5.680301	2.089291	-0.204550	5.950698	1.369473	4.784140	4.049983	-1.304236
H	-1.770395	4.889790	2.143260	-1.890139	5.149441	1.471763	5.611648	3.334342	0.213590
H	1.882407	-0.870492	-2.986195	2.010036	-1.212794	-2.671420	1.739200	0.308752	-2.648026
H	0.567432	-1.563167	-2.036634	0.669739	-1.787669	-1.679497	0.316428	-0.600149	-2.142338
H	7.213409	-3.795449	-2.040885	7.408361	-3.617314	-0.527800	6.871840	-3.262397	-2.355644
C	3.023510	2.486611	1.850751	2.937787	2.798421	1.690370	2.684212	1.180993	3.240202
C	2.080565	3.143372	2.824263	1.950403	3.574283	2.521819	1.743690	1.269291	4.415676
H	2.618158	3.962384	3.313062	2.461093	4.461636	2.909751	2.289177	1.719228	5.251519
H	1.208635	3.553536	2.290605	1.097912	3.892408	1.901115	0.886431	1.913669	4.165858
H	1.717094	2.426510	3.573341	1.562119	2.966490	3.350696	1.354492	0.281335	4.694846
O	4.123679	2.890449	1.595533	4.045577	3.175104	1.426716	3.762085	1.710690	3.202908
C	7.560068	-2.794362	-0.175758	8.764139	-2.295937	-1.553126	8.338215	-1.692218	-2.498234
H	7.720084	-1.777451	0.221308	8.813744	-1.680191	-2.467071	8.447506	-0.673660	-2.907491
H	6.676495	-3.224828	0.317221	8.918392	-1.628603	-0.690781	8.529345	-1.640429	-1.414876
H	8.431872	-3.404914	0.105184	9.603628	-3.007008	-1.584640	9.122401	-2.318534	-2.950023
C	6.160404	-1.938935	-2.096320	6.275986	-2.028465	-1.394042	5.874963	-1.386992	-2.141400
H	6.209478	-0.916556	-1.687656	6.475534	-1.255703	-0.631370	6.119988	-1.197475	-1.081745
H	6.113992	-1.823344	-3.193362	6.158954	-1.482651	-2.344738	5.814053	-0.392633	-2.613429
C	-4.965460	0.705297	-0.181162	-4.953865	0.629128	-0.385492	-5.160842	1.014640	0.412942
H	-5.722320	1.429805	0.122298	-5.731315	1.375235	-0.215986	-5.891984	1.512902	1.051065
C	-5.261331	-0.554960	-0.541794	-5.219110	-0.673356	-0.583078	-5.506414	0.194908	-0.593933
H	-4.428593	-1.201213	-0.834361	-4.366536	-1.338358	-0.749304	-4.695981	-0.250440	-1.178310
C	-6.586345	-1.174328	-0.592378	-6.533114	-1.317268	-0.604908	-6.860148	-0.176297	-1.009336
C	-7.765963	-0.496493	-0.248657	-7.734940	-0.620283	-0.407617	-8.017153	0.295794	-0.371081
C	-6.696901	-2.513104	-1.002213	-6.609168	-2.701138	-0.832261	-7.023560	-1.047959	-2.098273
C	-9.000090	-1.126894	-0.311955	-8.957346	-1.275604	-0.436575	-9.280034	-0.084887	-0.801107
H	-7.726333	0.544193	0.076349	-7.722288	0.455503	-0.226664	-7.936444	0.973915	0.479736
C	-7.924078	-3.155957	-1.070266	-7.824246	-3.369292	-0.863939	-8.279949	-1.436949	-2.539130
H	-5.793319	-3.063969	-1.273640	-5.687788	-3.267360	-0.987317	-6.138604	-1.430920	-2.611901
C	-9.087170	-2.462782	-0.723934	-9.009864	-2.656507	-0.665331	-9.419704	-0.955059	-1.889694
H	-9.906085	-0.579378	-0.038923	-9.881184	-0.712516	-0.279687	-10.167642	0.295052	-0.288263
H	-8.004275	-4.195715	-1.387997	-7.877676	-4.443889	-1.039753	-8.401128	-2.114580	-3.384520
O	-10.261408	-3.128641	-0.805065	-10.171212	-3.348255	-0.704505	-10.624066	-1.360444	-2.353028
H	-10.977774	-2.542093	-0.536502	-10.905866	-2.743668	-0.549244	-11.319880	-0.961553	-1.818198
O	-1.059355	-0.448917	1.564182	-1.108522	-0.197894	1.663112	-1.449006	-1.125895	1.467486
H	-1.968779	-0.760574	1.510315	-2.010117	-0.533246	1.622207	-2.369586	-1.292012	1.238776
C	0.569444	-2.370173	4.169144	0.426714	-1.721663	4.564705	-0.001913	-4.087693	3.089067
O	-0.342412	-3.055351	4.540801	-0.495245	-2.360449	4.990185	-0.933425	-4.842948	3.090599
C	1.549738	-1.695707	5.097645	1.361835	-0.912201	5.429690	0.948340	-3.920127	4.250032
H	2.566577	-2.084028	4.933116	2.387693	-1.305618	5.361405	1.964230	-4.234423	3.965372
H	1.571387	-0.612505	4.921336	1.382056	0.136547	5.106295	0.996924	-2.870344	4.567070
H	1.245095	-1.910461	6.126972	1.016258	-0.987080	6.465748	0.594560	-4.551890	5.071085
C	1.161215	6.149255	-0.460429	1.127259	6.077053	-1.173237	2.533351	5.834409	-0.868911
H	2.020834	6.316664	-1.113254	2.008933	6.164692	-1.812095	1.629183	6.302540	-1.267275
H	0.952065	6.907737	0.297124	0.883775	6.929720	-0.535588	3.494404	6.248461	-1.180725
C	0.626590	4.033285	-1.680265	0.656448	3.806383	-2.107614	1.136303	4.295656	0.518770

H	-0.293315	3.947229	-2.275208	-0.240650	3.625209	-2.715797	1.047611	4.668838	1.555168
H	1.411690	4.384143	-2.365431	1.461901	4.071223	-2.807663	0.309988	4.760429	-0.036837

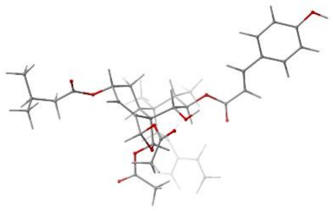

conformer **14-4**

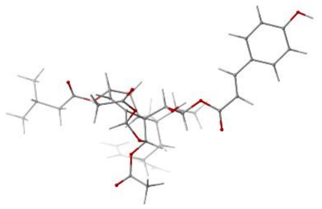

conformer **14-5**

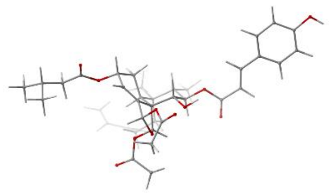

conformer **14-6**

O	4.815814	-3.599395	-1.015223	4.318217	-2.935179	-2.194991	4.188247	-2.481309	-3.109445
C	4.963171	-2.465366	-1.408321	4.528506	-1.767471	-1.962072	4.434486	-1.390913	-2.649827
C	7.527515	-2.656564	-1.695582	7.028541	-1.972726	-2.434502	6.957406	-1.715160	-2.799662
C	7.818766	-3.070769	-0.250770	6.919817	-2.209235	-3.941824	8.262394	-0.946169	-3.006119
O	3.940580	-1.600547	-1.544215	3.556092	-0.885635	-1.673406	3.497188	-0.579872	-2.128890
C	2.620339	-2.083539	-1.189520	2.193974	-1.384850	-1.657734	2.127279	-1.056895	-2.152336
H	2.517126	-3.098143	-1.600690	2.067511	-2.028587	-2.540369	1.951313	-1.512589	-3.137505
C	2.497424	-2.111166	0.304729	1.979586	-2.173400	-0.400915	1.951180	-2.074932	-1.066372
C	1.756821	-1.221228	0.955413	1.232072	-1.708885	0.595004	1.268267	-1.796590	0.038021
C	1.752617	-1.057281	2.449447	1.153535	-2.340844	1.962263	1.230235	-2.685182	1.249268
H	2.740550	-1.260347	2.892172	2.117852	-2.770713	2.267699	2.192197	-3.194875	1.416538
O	0.791497	-1.919215	3.015100	0.127249	-3.304324	2.082086	0.203892	-3.638248	1.110771
O	1.422472	0.298146	2.659088	0.826898	-1.285319	2.826688	0.981401	-1.807958	2.327964
C	1.319401	0.992841	1.441588	0.833841	-0.055330	2.154959	0.974399	-0.468809	1.905787
O	2.567723	1.581306	1.136624	2.116222	0.535789	2.269576	2.270578	0.078213	2.064897
C	1.000888	-0.053783	0.339745	0.570718	-0.339986	0.651650	0.632397	-0.461137	0.393071
C	-0.528929	-0.308818	0.300962	-0.955958	-0.413370	0.411601	-0.907144	-0.486241	0.225609
H	-0.687648	-1.297178	-0.169015	-1.117810	-0.921845	-0.557672	-1.125277	-0.834091	-0.801062
C	-1.271212	0.731643	-0.536361	-1.573090	0.979292	0.318010	-1.517891	0.902663	0.393099
H	-1.256153	1.698718	-0.017226	-1.504073	1.488716	1.288641	-1.416607	1.230101	1.436599
O	-2.640316	0.283131	-0.545584	-2.971496	0.747110	0.062701	-2.924070	0.718979	0.143235
C	-3.603236	1.190374	-0.259009	-3.870824	1.411649	0.831379	-3.796551	1.217655	1.054026
O	-3.382484	2.359446	-0.032298	-3.562108	2.186399	1.706774	-3.459095	1.808502	2.053743
C	-0.743645	0.872454	-1.960904	-0.970123	1.835062	-0.789901	-0.947221	1.946068	-0.560277
H	-0.742754	-0.138522	-2.405364	-1.077593	1.262090	-1.728657	-1.087336	1.555941	-1.584390
C	-1.696920	1.705951	-2.823478	-1.757242	3.135634	-0.971518	-1.733775	3.256816	-0.473332
C	0.751427	1.312159	-1.907598	0.561656	2.016644	-0.558447	0.590612	2.080273	-0.344464
C	1.329810	1.397388	-3.329172	1.157263	2.728102	-1.788226	1.159784	3.021363	-1.423148
C	0.980757	2.680173	-1.214485	0.813135	2.907129	0.686202	0.884805	2.705874	1.045148
C	0.282183	5.073068	-0.939670	2.861731	4.438516	0.183892	2.862793	4.382536	0.810534
C	-0.874797	4.823199	-0.046933	4.073200	4.114856	-0.601625	4.155029	4.323220	0.093149
C	-1.008733	5.263982	1.208606	4.416007	4.662031	-1.771734	4.459410	5.019910	-1.005552
C	1.535770	0.222236	-1.095792	1.223541	0.606874	-0.399433	1.245847	0.665961	-0.488831
H	2.566325	0.586631	-0.979156	2.262431	0.763963	-0.077922	2.299828	0.755768	-0.191966
C	1.632001	-1.129716	-1.835200	1.301214	-0.161244	-1.737295	1.254295	0.167577	-1.950934
H	7.868284	-2.192602	0.412921	5.967581	-2.693572	-4.200352	8.203939	-0.275464	-3.877793
H	8.785559	-3.593439	-0.186620	7.735548	-2.858171	-4.295736	9.104267	-1.636461	-3.167483
H	7.038000	-3.746144	0.127804	6.988444	-1.255300	-4.492527	8.500700	-0.329870	-2.122852
H	3.092999	-2.850368	0.844521	2.527127	-3.114274	-0.312932	2.462088	-3.032536	-1.186585
H	0.536424	1.746800	1.575630	0.064645	0.565458	2.628560	0.239635	0.054039	2.528503
H	-1.975519	2.653224	-2.343474	-1.848938	3.696018	-0.030228	-1.796849	3.631465	0.558415
H	-2.628359	1.146088	-2.990849	-2.776276	2.913236	-1.318678	-2.763265	3.101946	-0.826195
H	-1.265396	1.923526	-3.809964	-1.290124	3.786356	-1.722998	-1.285690	4.037337	-1.102808

H	2.429306	1.457860	-3.294585	2.248814	2.606678	-1.835108	2.254219	2.932674	-1.496858
H	0.968225	2.291768	-3.853861	0.961715	3.807998	-1.748269	0.942758	4.069752	-1.180975
H	1.052692	0.532066	-3.948418	0.731060	2.347412	-2.728383	0.732333	2.814195	-2.415209
H	2.053441	2.765486	-0.986428	0.400257	2.419372	1.580355	0.520810	2.040332	1.839373
H	0.476996	2.682689	-0.239649	0.223994	3.829373	0.568597	0.277977	3.618042	1.142187
H	-1.672432	4.186230	-0.448759	4.718339	3.338559	-0.173401	4.891081	3.618055	0.497777
H	-0.235353	5.866317	1.694421	3.777862	5.399154	-2.267108	3.733229	5.692687	-1.470460
H	-1.906167	5.033311	1.785939	5.341741	4.379678	-2.277081	5.439550	4.931271	-1.478713
H	1.959842	-0.980010	-2.870051	1.710809	0.479375	-2.525131	1.640858	0.944896	-2.618328
H	0.657038	-1.637507	-1.879401	0.305366	-0.484389	-2.075484	0.239212	-0.075663	-2.299166
H	8.350617	-1.996992	-2.023171	6.949059	-2.950029	-1.929642	6.742842	-2.300247	-3.709342
C	2.967347	2.752027	1.714005	2.566179	1.019429	3.464162	2.770641	0.365139	3.300918
C	1.995527	3.460285	2.620707	1.593649	1.081684	4.613403	1.838911	0.268807	4.482245
H	2.506987	4.327894	3.050065	2.123738	1.475253	5.486479	2.416523	0.482194	5.387305
H	1.122058	3.805873	2.045470	0.752742	1.748775	4.368842	1.028783	1.008779	4.389175
H	1.638020	2.796131	3.419619	1.181752	0.086591	4.828968	1.380798	-0.727044	4.547511
O	4.062262	3.160017	1.443164	3.705386	1.393715	3.519050	3.916893	0.712769	3.372751
C	7.501637	-3.862438	-2.638296	8.372866	-1.341448	-2.071409	7.070846	-2.689044	-1.625585
H	6.706433	-4.565950	-2.352645	8.481954	-0.349346	-2.541231	7.270472	-2.147287	-0.685333
H	7.328354	-3.549814	-3.680736	8.478675	-1.210472	-0.983075	6.146650	-3.271722	-1.501602
H	8.462578	-4.398672	-2.605829	9.208194	-1.967995	-2.419153	7.896565	-3.399399	-1.784732
C	6.260043	-1.800849	-1.810163	5.881943	-1.100235	-1.921945	5.795716	-0.739749	-2.602098
H	6.346922	-0.890042	-1.194529	6.048165	-0.807776	-0.870762	5.885359	-0.192181	-1.649759
H	6.124948	-1.441207	-2.843939	5.815509	-0.153471	-2.483895	5.797398	0.030310	-3.393240
C	-4.955397	0.606093	-0.253211	-5.265891	1.085511	0.485486	-5.202731	0.962364	0.693860
H	-5.736447	1.328544	-0.012605	-5.997387	1.592730	1.116292	-5.911884	1.340911	1.431290
C	-5.210060	-0.686162	-0.519831	-5.614009	0.247418	-0.505144	-5.582438	0.333609	-0.431184
H	-4.355006	-1.327246	-0.753601	-4.806530	-0.225387	-1.071876	-4.791912	-0.001934	-1.108822
C	-6.515648	-1.347130	-0.536320	-6.972113	-0.118306	-0.912322	-6.949823	0.042368	-0.865311
C	-7.719418	-0.679140	-0.264570	-8.116597	0.544619	-0.444531	-8.085073	0.393430	-0.119066
C	-6.581158	-2.717726	-0.835894	-7.152886	-1.181349	-1.812053	-7.149837	-0.621740	-2.086484
C	-8.933671	-1.349519	-0.290998	-9.386183	0.155398	-0.847331	-9.362069	0.095420	-0.572109
H	-7.714689	0.385583	-0.026701	-8.018967	1.390099	0.238208	-7.976020	0.909159	0.836100
C	-7.788007	-3.400744	-0.865682	-8.416196	-1.584365	-2.219039	-8.420698	-0.925943	-2.551869
H	-5.658024	-3.261434	-1.049871	-6.277277	-1.709251	-2.197061	-6.282418	-0.907408	-2.686148
C	-8.975730	-2.716855	-0.592088	-9.544313	-0.916018	-1.735078	-9.538310	-0.567165	-1.793421
H	-9.859208	-0.808974	-0.075719	-10.263886	0.690386	-0.474947	-10.232169	0.378025	0.026455
H	-7.833278	-4.465046	-1.097481	-8.551889	-2.413928	-2.913295	-8.570286	-1.442395	-3.500261
O	-10.128786	-3.422271	-0.632403	-10.755659	-1.340830	-2.161781	-10.757941	-0.883569	-2.285306
H	-10.865835	-2.837479	-0.422332	-11.442488	-0.803965	-1.750278	-11.435527	-0.586365	-1.667358
O	-1.040095	-0.297531	1.610593	-1.557912	-1.136576	1.451825	-1.468081	-1.361522	1.171552
H	-1.937940	-0.643266	1.574174	-2.498157	-1.195639	1.249757	-2.400234	-1.462165	0.951235
C	0.604532	-1.980196	4.363150	0.363689	-4.641981	2.069787	-0.000645	-4.598715	2.055720
O	-0.296090	-2.657150	4.775108	-0.590093	-5.387851	2.087629	-0.940821	-5.330423	1.918249
C	1.559146	-1.217275	5.248970	1.793464	-5.138826	2.061481	0.981999	-4.695886	3.197623
H	2.584339	-1.600033	5.128867	2.354302	-4.770671	1.191416	1.986415	-4.944972	2.822251
H	1.565755	-0.150988	4.988559	2.326624	-4.819399	2.969710	1.051048	-3.742150	3.736624
H	1.243250	-1.356473	6.287851	1.759224	-6.232809	2.031760	0.642984	-5.491050	3.869356
C	1.025123	6.184630	-0.868085	2.355163	5.678682	0.212345	2.227446	5.545214	1.005753
H	1.888325	6.328724	-1.521749	1.462371	5.912631	0.798627	1.270023	5.593923	1.531041
H	0.786010	6.989065	-0.169040	2.825390	6.502022	-0.329029	2.656099	6.489171	0.663549
C	0.562912	3.973177	-1.939159	2.262718	3.313837	1.014793	2.348056	3.060507	1.360826
H	-0.346484	3.819489	-2.536520	2.925809	2.439303	0.988643	3.013072	2.247263	1.040408

H	1.347089	4.295888	-2.639065	2.268929	3.642368	2.066477	2.458796	3.096129	2.457036

Table S10. Coordinates of Compound **17**

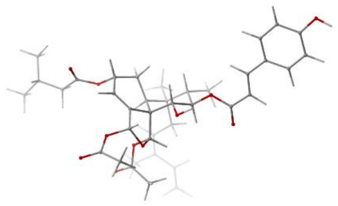
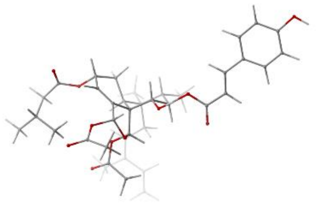
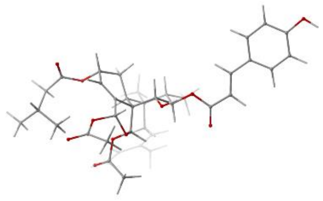

	conformer **17-1**			conformer **17-2**			conformer **17-3**		
O	4.086742	-4.028408	-0.796886	3.860400	-3.643173	-2.584052	3.950625	-2.838517	-3.344135
C	4.294171	-2.910154	-1.204371	4.064123	-2.452801	-2.557507	4.142423	-1.704914	-2.973348
C	6.759867	-3.362527	-1.666352	6.115132	-1.993331	-1.193153	6.147935	-1.606831	-1.471012
C	6.550359	-4.266299	-2.882455	7.534093	-1.429554	-1.252078	7.565738	-1.050798	-1.345545
O	3.319478	-1.998787	-1.387054	3.074790	-1.539158	-2.465871	3.143864	-0.861005	-2.639443
C	1.980633	-2.390357	-0.996597	1.741183	-2.044484	-2.217237	1.810353	-1.419548	-2.566269
H	1.824862	-3.424441	-1.336159	1.547747	-2.862548	-2.925650	1.625031	-1.985851	-3.490202
C	1.877310	-2.312772	0.499462	1.694680	-2.543049	-0.800106	1.753526	-2.322626	-1.366245
C	1.216965	-1.324371	1.089597	1.171814	-1.797259	0.165053	1.221519	-1.900719	-0.226138
C	1.217706	-1.028060	2.563824	1.323040	-2.050837	1.640137	1.356784	-2.588195	1.104207
H	0.384244	-1.510982	3.092881	0.518217	-2.670421	2.059903	0.552026	-3.308715	1.305889
O	2.452197	-1.413110	3.116760	2.583050	-2.636600	1.870766	2.619523	-3.208723	1.168014
O	1.017466	0.369708	2.650884	1.227643	-0.773656	2.238764	1.244744	-1.552399	2.060603
C	0.987517	0.950109	1.373719	1.162962	0.229914	1.260821	1.184106	-0.300263	1.434874
O	2.284941	1.348976	0.988804	2.463371	0.662927	0.920110	2.484116	0.234776	1.266505
C	0.536864	-0.163144	0.390910	0.550311	-0.424494	-0.004429	0.598995	-0.540209	0.018150
C	-0.996109	-0.346021	0.416626	-0.986284	-0.527389	0.103712	-0.938600	-0.665035	0.062079
H	-1.212076	-1.315409	-0.071431	-1.313847	-1.237714	-0.679257	-1.252231	-1.095134	-0.907803
C	-1.711488	0.734153	-0.383327	-1.667669	0.804217	-0.179055	-1.612677	0.692643	0.199056
H	-1.580654	1.715886	0.090731	-1.420668	1.538329	0.599266	-1.382792	1.136179	1.177055
O	-3.110771	0.403565	-0.290347	-3.078386	0.532605	-0.071257	-3.026042	0.412020	0.187020
C	-3.976228	1.407569	0.007974	-3.833550	1.399538	0.652736	-3.792288	0.990219	1.148425
O	-3.631015	2.545510	0.224904	-3.381094	2.362669	1.226149	-3.348035	1.696006	2.023476
C	-1.269435	0.761634	-1.845418	-1.335389	1.338197	-1.571233	-1.255850	1.648374	-0.939449
H	-1.433115	-0.255147	-2.245348	-1.611931	0.544781	-2.288815	-1.535443	1.138338	-1.879497
C	-2.145788	1.709768	-2.668099	-2.188369	2.563668	-1.909175	-2.097361	2.923611	-0.851209
C	0.263380	1.031480	-1.932978	0.203480	1.543054	-1.710569	0.284406	1.866103	-0.999907
C	0.713873	0.925519	-3.403235	0.530039	1.952550	-3.160198	0.647645	2.681349	-2.251425
C	0.569173	2.476907	-1.462806	0.665860	2.703461	-0.791808	0.832385	2.628146	0.255790
C	2.488377	4.130951	-1.271577	2.735090	4.061878	-0.213916	1.834283	4.974588	0.301480
C	1.591944	5.208376	-0.823028	1.974941	4.949749	0.680236	3.228560	4.514757	0.423955
C	1.981242	6.417876	-0.399742	2.502011	5.895362	1.468648	4.301681	5.314364	0.461030
C	1.027097	-0.030936	-1.075115	0.941133	0.207352	-1.366463	1.003114	0.473619	-1.084576
H	2.078408	0.281507	-1.016858	2.014084	0.430149	-1.300962	2.076592	0.664009	-0.952106
C	1.037616	-1.436262	-1.710859	0.807612	-0.871184	-2.461491	0.876477	-0.225279	-2.454676
H	5.588860	-4.796223	-2.822726	8.126165	-1.898858	-2.053732	8.181486	-1.304656	-2.222843
H	7.347706	-5.022340	-2.950598	8.058432	-1.596756	-0.299091	8.064288	-1.454937	-0.451533
H	6.567722	-3.676878	-3.815478	7.514718	-0.342064	-1.434895	7.547121	0.047712	-1.249093
H	2.436818	-3.041216	1.088346	2.221155	-3.470222	-0.569296	2.278530	-3.277253	-1.421089
H	0.302752	1.804005	1.436803	0.565116	1.042671	1.689197	0.571907	0.344129	2.076379
H	-2.173301	2.722320	-2.240752	-2.097548	3.351911	-1.148138	-2.017923	3.392748	0.140082
H	-3.180533	1.339334	-2.693713	-3.249700	2.281893	-1.960929	-3.158804	2.689903	-1.016727
H	-1.798874	1.776815	-3.708060	-1.916263	2.985666	-2.886024	-1.801236	3.656646	-1.613238

H	1.805666	0.813563	-3.479750	1.600096	1.824349	-3.381135	1.737905	2.679041	-2.409226
H	0.442839	1.832432	-3.962541	0.289812	3.011632	-3.331686	0.345250	3.730739	-2.148049
H	0.251802	0.069300	-3.916332	-0.037199	1.361012	-3.893811	0.164850	2.284810	-3.155942
H	0.213386	2.632249	-0.433149	0.398657	2.498119	0.255874	1.867672	2.307922	0.397725
H	-0.046702	3.155030	-2.075007	0.069608	3.589986	-1.061352	0.285960	2.301159	1.156235
H	0.519111	4.992590	-0.834211	0.888996	4.815447	0.700130	3.404373	3.439550	0.508808
H	3.033366	6.708358	-0.356788	3.576453	6.088548	1.506720	4.230187	6.402402	0.393154
H	1.249293	7.161579	-0.079097	1.865784	6.511086	2.107193	5.302350	4.891219	0.565147
H	1.368166	-1.383133	-2.754531	1.065593	-0.454195	-3.441823	1.148557	0.465120	-3.260579
H	0.033357	-1.887032	-1.718268	-0.221144	-1.255900	-2.537989	-0.151380	-0.564557	-2.655702
H	6.722449	-3.992696	-0.762357	6.178737	-3.081577	-1.019767	6.211377	-2.703821	-1.575819
C	2.894934	2.434081	1.550147	3.200080	1.443995	1.760532	3.175617	0.797909	2.295247
C	2.051224	3.360117	2.386545	2.503561	2.054016	2.948327	2.554082	0.769571	3.666943
H	2.703074	4.143723	2.785786	3.247211	2.608654	3.529214	3.298022	1.127939	4.385475
H	1.276419	3.829676	1.760790	1.725438	2.754830	2.608604	1.680683	1.439592	3.696107
H	1.561270	2.820444	3.208504	2.033853	1.281752	3.572840	2.217128	-0.242160	3.927580
O	4.058645	2.605009	1.317023	4.355009	1.626668	1.489561	4.235826	1.303370	2.043408
C	8.120095	-2.667120	-1.723056	5.316048	-1.366602	-0.050280	5.315697	-1.287818	-0.228323
H	8.189232	-2.007312	-2.604537	5.202542	-0.278166	-0.178470	5.200286	-0.201152	-0.084388
H	8.296736	-2.050769	-0.827867	4.309834	-1.798002	0.045684	4.310474	-1.729427	-0.271185
H	8.935425	-3.403213	-1.793080	5.822659	-1.537418	0.911516	5.800678	-1.695994	0.671359
C	5.642916	-2.325619	-1.542478	5.420859	-1.802198	-2.558468	5.492133	-1.073780	-2.763114
H	5.866495	-1.603694	-0.737834	5.320368	-0.730393	-2.785110	5.388143	0.020097	-2.709954
H	5.544265	-1.725317	-2.462022	6.019858	-2.281261	-3.347150	6.119431	-1.333939	-3.628361
C	-5.378801	0.958279	0.042232	-5.261008	1.035517	0.665918	-5.221448	0.660032	1.011015
H	-6.077224	1.747861	0.322889	-5.870053	1.701065	1.279156	-5.840274	1.085892	1.801996
C	-5.769667	-0.292086	-0.257517	-5.772078	-0.001178	-0.019771	-5.723096	-0.077083	0.005494
H	-4.990865	-1.007083	-0.538699	-5.075931	-0.599808	-0.614677	-5.017692	-0.447809	-0.744039
C	-7.135726	-0.817287	-0.256627	-7.171037	-0.428064	-0.069561	-7.123131	-0.444168	-0.211360
C	-8.255866	-0.045807	0.088869	-8.197015	0.213682	0.640944	-8.160260	-0.067286	0.655563
C	-7.350570	-2.157682	-0.616976	-7.517709	-1.533528	-0.863557	-7.459325	-1.211096	-1.338946
C	-9.532601	-0.588079	0.074714	-9.509611	-0.228152	0.561672	-9.473542	-0.438909	0.407347
H	-8.134749	0.999673	0.376190	-7.972465	1.075624	1.270928	-7.943920	0.527652	1.544111
C	-8.621387	-2.713068	-0.635686	-8.825784	-1.986094	-0.952427	-8.767950	-1.589658	-1.599758
H	-6.495209	-2.779969	-0.890489	-6.737565	-2.050912	-1.426914	-6.670355	-1.517480	-2.029863
C	-9.723947	-1.927379	-0.288460	-9.833029	-1.332569	-0.236988	-9.786409	-1.203489	-0.723910
H	-10.390734	0.031632	0.347959	-10.292722	0.286983	1.124225	-10.265491	-0.134136	1.096593
H	-8.782913	-3.754056	-0.916401	-9.089760	-2.844502	-1.570469	-9.023770	-2.184673	-2.476717
O	-10.943492	-2.510171	-0.320627	-11.093322	-1.808065	-0.353155	-11.046860	-1.594865	-1.017315
H	-11.611093	-1.864529	-0.062034	-11.686126	-1.271971	0.185922	-11.647910	-1.264815	-0.339661
O	-1.460720	-0.372421	1.748010	-1.352414	-1.018989	1.373734	-1.325598	-1.525868	1.110418
H	-2.418902	-0.470519	1.696609	-2.316616	-1.044958	1.384016	-2.289581	-1.554489	1.094157
C	2.639154	-1.410959	4.470461	2.941231	-3.065391	3.117690	2.961504	-3.993983	2.232853
O	3.717786	-1.723389	4.888339	4.062782	-3.457034	3.274432	4.090600	-4.389898	2.296748
C	1.471984	-1.029196	5.349475	1.894283	-3.036088	4.204734	1.888494	-4.327694	3.240990
H	1.033181	-0.071596	5.039601	1.456741	-2.034112	4.307255	1.417309	-3.418379	3.637672
H	0.684189	-1.795849	5.290549	1.079397	-3.737335	3.968386	1.101742	-4.938137	2.771949
H	1.834007	-0.969984	6.381059	2.371304	-3.342876	5.141169	2.352576	-4.900819	4.050154
C	2.011275	2.906378	-1.561042	2.129644	3.057255	-0.873601	0.786695	4.129552	0.224662
H	2.741417	2.148168	-1.857274	2.764944	2.421157	-1.496184	-0.205500	4.583991	0.150603
C	3.961583	4.426216	-1.356617	4.218502	4.283751	-0.337408	1.599139	6.463364	0.267542
H	4.516536	3.553634	-1.725049	4.662555	3.580608	-1.054108	0.528195	6.695776	0.190138
H	4.159466	5.278553	-2.026535	4.441785	5.311032	-0.667768	1.991760	6.948537	1.175694

H	4.366512	4.673824	-0.363048	4.717987	4.121675	0.630463	2.112177	6.925123	-0.591785

Table S11. Aromatase inhibition data of compounds at a concentration of 12.5 µM from the CH_2Cl_2 extract of *C. grewiifolia*

**Compound**	% Inhibition
**3**	15%
**5**	18%
**7**	0%
**9**	31%
**11**	25%
**12**	0%
**19**	17%
**20**	0%
Letrozole[a]	IC_{50} = 1.43 ± 0.3 nM

[a]Letrozole is used as a positive control.
